# Boosting peptide half-life: enabling efficient generation of Fc–peptide conjugates

**DOI:** 10.1039/d6sc02646j

**Published:** 2026-05-29

**Authors:** Mahri Park, Friederike M. Dannheim, Monika A. Papworth, Richard Kay, Elpida Tsonou, Daniel Trajkovski, Stephen J. Walsh, Daniel Hovdal, Thomas Wharton, Anne-Chloe Nassoy, Jeremy S. Parker, David R. Spring

**Affiliations:** a Yusuf Hamied Department of Chemistry, University of Cambridge Cambridge UK spring@ch.cam.ac.uk; b Oncology R&D, AstraZeneca UK; c Institute of Metabolic Science, Department of Clinical Biochemistry, University of Cambridge UK; d BioPharmaceuticals R&D, AstraZeneca UK; e Department of Surgery, University of Cambridge UK; f Preclinical and Translational PK & PKPD Specialist (DMPK), AstraZeneca Gothenburg Sweden; g Early Chemical Development, Pharmaceutical Development, R&D, AstraZeneca Macclesfield UK

## Abstract

Therapeutic peptides constitute a valuable class of drug candidates due to their ability to modulate targets which are often considered ‘undruggable’ by traditional small molecule drugs. Nevertheless, many peptides suffer from extremely short plasma half-life which limit their therapeutic potential. The fusion of therapeutic peptides to the Fc region of IgG antibodies has emerged as a highly established and effective approach for prolonging the systemic half-life of peptides. These Fc-fusion compounds are produced using recombinant techniques, expressed as a single linear polypeptide chain. However, such recombinant approaches limit incorporation of non-proteinogenic features such as peptides with non-natural amino acids, unnatural cyclic peptides, or pharmaceutical oligonucleotides. These features can increase stability, improve biological activity, and offer unique chemical properties. Therefore, the development of novel methods for the generation of Fc-fusion proteins which allow incorporation of these non-proteinogenic components is desirable. This work reports a semi-synthetic strategy for generating Fc–peptide conjugates through the design, synthesis, and bioconjugation of functionalised disulfide re-bridging linkers. Using this approach, an Fc–peptide conjugate was generated displaying retained biological activity *in vitro* and prolonged circulation *in vivo*. This methodology allows the simple and efficient generation of Fc–peptide conjugates.

## Abstract 2

Herein, we report a semi-synthetic strategy for generating Fc–peptide conjugates *via* disulfide re-bridging linker bioconjugation that retained biological activity *in vitro* and exhibited prolonged circulation *in vivo*.

## Graphical abstract

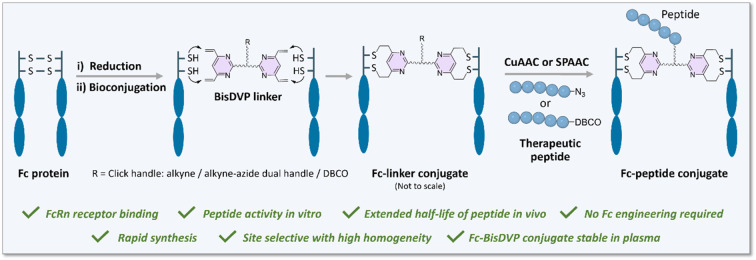

## Introduction

Peptides represent an extensive pool of valuable drug candidates.^1^ Compared to traditional small molecule drugs, these molecules possess certain advantages such as high specificity and low toxicity resulting from exceptionally strong binding to their targets.^2^ This strong binding is a result of the large chemical space covered by side-chain variations of native amino acids.^2,3^ These qualities allow lower dosing and minimise off-target side effects resulting in an improved drug safety profile. In addition, the size and topology of these biotherapeutics allows them to interact with many sites which small molecule drugs are unable to target. Small molecules can efficiently bind to pockets that naturally accept small ligands, however disrupting disease-associated protein–protein interactions (PPIs) – which often occur through flat surfaces rather than distinct binding pockets – continues to be a significant challenge.^3^ In contrast, biotherapeutics such as peptides and small protein-based structures are able to inhibit PPIs that small molecules cannot.^4,5^ Despite these benefits, the therapeutic application of these biomolecules is limited by their rapid clearance and short half-life in circulation which arise from a combination of factors, including proteolytic degradation, rapid metabolism, and a high susceptibility to renal clearance.^6,7^ A common half-life extension strategy involves the fusion of a biotherapeutic to the Fc portion of an IgG antibody, resulting in what is known as an Fc-fusion compound.^8,9^ This approach is one of the most effective techniques for the half-life extension of peptide and protein biotherapeutics. IgG antibodies exhibit an extraordinarily long half-life of approximately 21 days.^10^ This long circulatory half-life is due to their large size and ability to bind the neonatal Fc receptor (FcRn) which allows the antibody to be rescued from lysosomal degradation.^11,12^ IgG binds to the FcRn *via* the Fc domain, therefore biomolecules of interest can be joined to an Fc protein as a valuable half-life extension mechanism. Fc fusions and conjugates have an extended circulation half-life due to this interaction with the FcRn receptor and additionally due to the increased hydrodynamic diameter of the protein species compared to free drug which slows renal filtration. There are approximately 13 Fc fusion therapeutics approved in the European Union and the United States and a further estimated 37 therapeutic fusion products in the clinic.^13–25^ Despite the successes of Fc fusion proteins, an outstanding problem in this field is the lack of efficient methods that enable the incorporation of non-proteinogenic payloads. Fc–peptide fusions are fully recombinant molecules, coded in DNA and expressed as a single, continuous polypeptide chain. In contrast, Fc–peptide conjugates are bioconjugates in which the Fc domain and peptide are produced separately and subsequently joined through chemical coupling after production. Notably, recombinant production of Fc-fusion compounds prevents the incorporation of many cargoes such as unnatural peptides, cyclic peptides, oligonucleotides, nucleic acids, lipids, PEGs and small molecule payloads such as those found in ADCs. In addition to these limits, the overall recombinant production of Fc-fusion compounds can be time consuming and require laborious case-by-case optimisation.

Recent efforts have resulted in several novel semi-synthetic methods for the generation of Fc-conjugates as an alternative to recombinant expression, which allow the incorporation of non-proteogenic components.^26–30^ However, these strategies typically require extensive engineering or alteration of the Fc protein to achieve functionalisation. Disulfide re-bridging reagents have emerged as an attractive technique for the effective functionalisation of antibodies. Notably, in 2022, Novo Nordisk reported the development of insulin–Fc conjugates using trifunctional bishalo-acetamide linkers.^31^ These linkers enabled acylation of a lysine residue on insulin and re-bridging of a reduced interchain disulfide bond within the Fc protein. To limit insulin receptor-mediated clearance, conjugates with only one insulin per Fc domain were produced by engineering an Fc protein with a single hinge-region disulfide bond, preventing half-Fc formation. Because the Fc hinge region is sterically shielded, flexible peptide spacers of varying lengths were inserted between insulin and Fc which influenced conjugate size and receptor affinity.^31^ Overall, the strategy generated a potent insulin–Fc conjugate with extended half-life. However, it relies on engineered Fc protein and was developed specifically for insulin, therefore may be less applicable to other peptides, especially those containing lysine residues as part of the active sequence.

In 2023, Thoreau *et al.* applied bis-dibromopyridazinedione (bis-diBrPD) chemistry to generate Fc-conjugates.^32^ A trifunctional linker, consisting of two phenyl azide-containing pyridazinedione moieties connected by a tetrazine unit, was used to re-bridge two reduced Fc hinge disulfides. Fc fragments obtained by papain digestion were reduced, purified, and conjugated with good efficiency and homogeneity. The resulting Fc conjugates displayed two phenyl-N_3_ handles and one Me–Tz handle, which enabled copper-free click chemistry with payloads such as rhodamine, fluorescein, or biotin. The approach provided rapid conjugation with low linker equivalents and avoided the need for recombinant proteins, though its application to therapeutic peptides and half-life extension has not yet been evaluated.

The same bis-diBrPD strategy was also employed to generate bispecific antibodies.^33^ By re-bridging Fc disulfides and attaching two distinct Fab fragments *via* click chemistry, IgG-like bispecifics were produced. A T cell-engager created with this method was able to effectively kill HER2+ve cells in co-culture assays. This represents one of the first demonstrations of site-selective chemical modification of a non-engineered Fc fragment. However, their work did not extend to the formation of Fc–peptide conjugates, nor did it evaluate the pharmacokinetics of the resulting constructs. This highlights the need for efficient chemical strategies to generate Fc–peptide conjugates with demonstrated biological efficacy and extended circulation half-life.

**Fig. 1:**
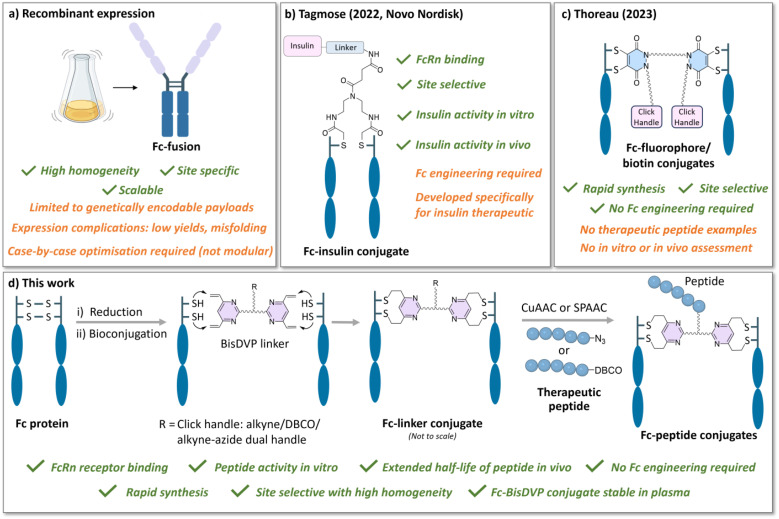
Examples of Fc-fusion and Fc-conjugate generation: recombinant expression to generate Fc-fusion (a), generation of Fc–insulin conjugate^31^ (b), generation of Fc–fluorophore/biotin conjugates^32^ (c) and the general principle for the formation of Fc–peptide conjugates *via* the re-bridging of two disulfide bonds of Fc protein with a bis-divinylpyrimidine trifunctional linker, introducing a click handle allowing the attachment of a therapeutic peptide (d). Note the illustrations shown of Fc–linker conjugates throughout are not to scale.

Herein, we present a disulfide re-bridging strategy employing a dual-bridging linker capable of reacting with the four cysteine residues of a reduced IgG1 Fc fragment, effectively restoring all interchain disulfide bonds (Fig. 1). Incorporation of this reagent into a two-step re-bridging-click workflow permits the efficient synthesis of Fc conjugates that display both preserved biological efficacy and substantially prolonged circulation times, thereby providing a non-recombinant route to long-acting Fc-based therapeutics.

## Results and discussion

**Fig. 2:**
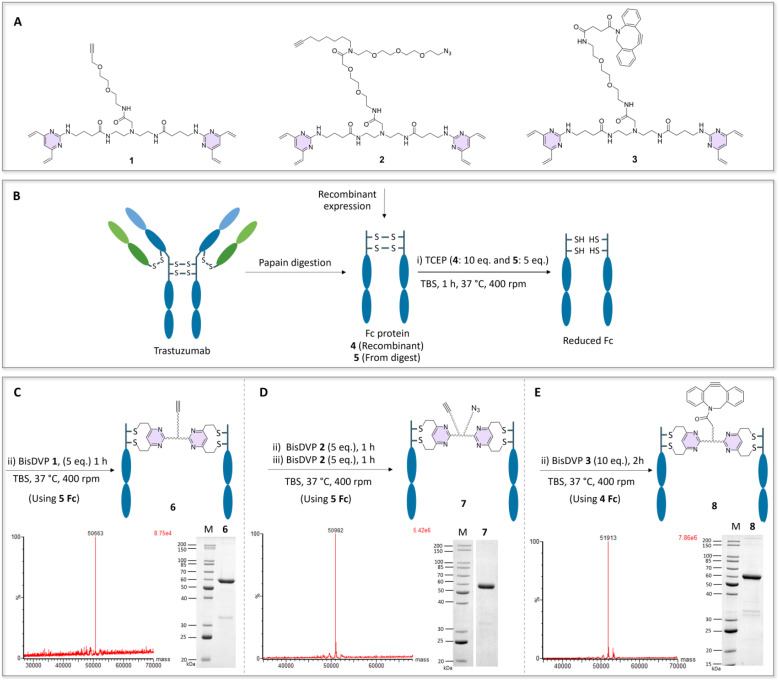
(A) Structures of BisDVP linkers 1, 2 and 3. (B) Reaction scheme of enzymatic Fc production from native mAb and expression of recombinant Fc protein, followed by TCEP reduction of Fc (C) reaction of reduced Fc 5 (from enzymatic digestion) with BisDVP 1, to form Fc-conjugate 6. LC-MS and SDS-PAGE analysis of Fc-conjugate 6. (D) Reaction of reduced Fc 5 (from enzymatic digestion) with BisDVP 2, to form Fc-conjugate 7. LC-MS and SDS-PAGE analysis of Fc-conjugate 7. (E) Reaction of reduced Fc 4 (from recombinant expression) with BisDVP 3, to form Fc-conjugate 8. LC-MS and SDS-PAGE analysis of Fc-conjugate 8. All SDS-PAGE using 12% polyacrylamide gel under reducing conditions. Lane: M = molecular weight marker.

**Fig. 3:**
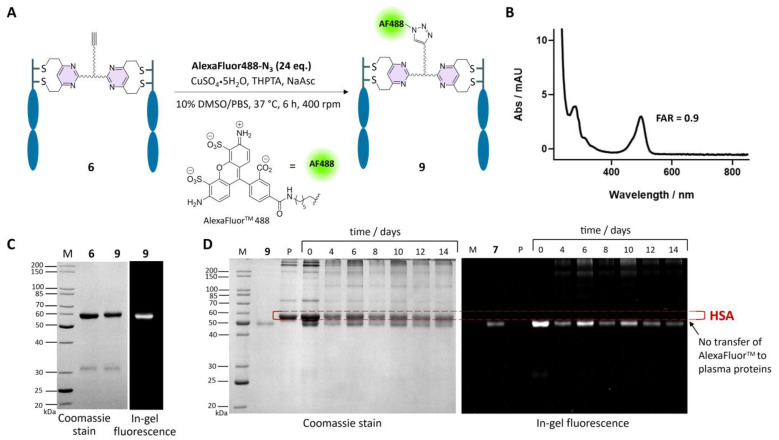
(A) CuAAC reaction between 6 and Alexa Fluor™ 488 azide to form conjugate 9. (B) UV-visible absorbance of 9. (C) SDS-PAGE analysis of 6 and 9 under reducing conditions. (D) Stability analysis of Fc-conjugate 9 in human plasma by SDS-PAGE under non-reducing conditions; M = molecular weight marker, P = human plasma, days of incubation are depicted above the representative lane. Left gel is after Coomassie staining, right gel is in-gel fluorescence measured before staining (see SI for maleimide control).

In an IgG1 Fc protein, reduction of the two interchain disulfide bonds exposes four reactive cysteine residues, providing suitable sites for selective re-bridging. To exploit this, the Spring Group developed a bis-divinylpyrimidine (BisDVP) linker containing two cysteine-reactive moieties, originally designed to restore disulfide connectivity in full IgG antibodies and prevent “half-antibody” formation.^34^ This linker was therefore ideal for Fc re-bridging, incorporating a functional alkyne handle for copper-catalysed azide–alkyne cycloaddition (CuAAC) to enable site-specific drug attachment. The spacing between the two DVP units was previously optimised using IgG1 crystal structure data, which indicated an interchain disulfide distance of approximately 20 Å, ensuring structural compatibility.^34^ A series of BisDVP linker variants bearing different click handles were synthesised to facilitate Fc conjugate formation (Fig. 2A, see SI for full synthetic details). These were synthesised using traditional solution-based chemical synthesis but could be synthesised by solid-phase synthesis, following an approach analogous to our recent TetraDVP work.^35^ BisDVP thus acts as a dual-bridging reagent with a functional handle, enabling covalent restoration of disulfide bonds while introducing a point of further modification. Fc protein was obtained either by recombinant expression in mammalian cells (to produce Fc protein 4) or by papain digestion of the full-length IgG1 antibody trastuzumab^36^ (to produce Fc protein 5) (Fig. 2B, see SI). Both Fc proteins 4 and 5 share an identical amino acid sequence, with the sole exception that Fc 4 contains four additional residues N-terminal to the hinge region. In Fc 5, these residues were removed by enzymatic digestion. This structural difference may account for the modest variation in conditions required for their conjugation. Enzymatic digestion of antibodies provides a rapid and accessible route to Fc fragments using readily available, off-the-shelf antibodies (which are themselves recombinantly expressed but typically obtained commercially). However, digestion is unsuitable for large-scale production, where recombinant expression provides a more practical and scalable solution. As unmodified, non-engineered, IgG1 Fc is relatively easy to express and requires minimal optimisation, both approaches were considered valuable and employed for Fc generation. The interchain disulfide bonds of the IgG1 Fc protein were selectively reduced using tris(2-carboxyethyl)phosphine hydrochloride (TCEP) for 1 hour, followed by incubation with BisDVPs 1, 2 or 3 at 37 °C for 1–2 hours (Fig. 2C–E). The reaction mixtures were then passed through a desalting column and subjected to buffer exchange/ultrafiltration, yielding Fc–BisDVP conjugates 6, 7, and 8 (Fig. 2C–E). Characterisation by liquid chromatography-mass spectrometry (LC-MS) and reducing SDS-PAGE confirmed efficient disulfide re-bridging, with each conjugate incorporating a single linker per Fc protein, exhibiting high purity and homogeneity. SDS-PAGE analysis demonstrated minimal formation of “half-Fc” species, indicating that the predominant Fc-conjugate product resulted from successful re-bridging of the interchain disulfide bonds, thereby restoring covalent linkage between the two Fc heavy chains (Fig. 2C–E). Importantly, non-covalent interactions between the two heavy chains preserved their association throughout reduction, enabling complete re-bridging without formation of dissociated heavy chains. The resulting Fc conjugates contained an accessible click handle for subsequent functionalisation. Following optimisation of Fc bioconjugation, control experiments were performed to confirm the chemoselectivity and connectivity of the Fc–BisDVP conjugate. To assess chemoselectivity, non-reduced Fc (lacking free thiols) was incubated with BisDVP 1 at 37 °C for 2 h. LC-MS analysis of the reaction (for both digested and recombinant Fc) showed only unmodified Fc protein, confirming that BisDVP reacts exclusively with free cysteine residues (see SI). To verify the connectivity, Fc proteins modified with 1 were treated with excess *N*-(*tert*-butoxycarbonyl)-l-cysteine methyl ester to quench any unreacted vinyl groups indicative of incomplete re-bridging. LC-MS analysis detected only the expected mass adducts and no addition of cysteine (see SI). These results demonstrate that BisDVP achieves site-selective and complete disulfide re-bridging in both Fc protein formats. With respect to the regioselectivity of the Fc–linker conjugation – the four thiols of the Fc can react with the four vinyl groups of the BisDVP linker in multiple orientations (see SI) but this variability is not therapeutically relevant as the resulting regioisomers are expected to have equivalent properties. For simplicity, the Fc–BisDVP conjugates will be illustrated as a single isomer throughout. To evaluate the final step of Fc-conjugate synthesis, the alkyne-containing Fc–BisDVP conjugate was reacted with Alexa Fluor™ 488 Azide *via* copper-catalysed azide–alkyne cycloaddition (CuAAC) in PBS with CuSO_4_·5H_2_O, THPTA, and sodium ascorbate for 6 hours, using 24 equivalents of fluorophore (Fig. 3A). Excess reagents were removed by sequential desalting with two Zeba™ Spin Columns (7K MWCO). SDS-PAGE with in-gel fluorescence confirmed attachment of Alexa Fluor 488 (AF488) to the Fc fragment, and UV-vis analysis indicated near-complete conversion with a fluorophore-to-antibody ratio of 0.9, demonstrating the efficiency of the CuAAC reaction (Fig. 3C). To assess the stability of Fc conjugates in human plasma, we compared the performance of BisDVP linkages with conventional maleimide conjugates. Alexa Fluor 488 was introduced as a fluorescent reporter to allow qualitative analysis of payload transfer by SDS-PAGE. The resulting Fc–BisDVP–AF488 (9) and Fc–maleimide–AF488 (S21) conjugates were incubated in human plasma at 37 °C for up to 14 days, with aliquots collected at defined intervals and analysed by in-gel fluorescence (Fig. 3, see SI). As expected, the Fc–maleimide–AF488 conjugate S21 exhibited instability, with fluorescence transfer to human serum albumin (HSA, ∼67 kDa) detectable after four days (see SI). This observation is consistent with the known susceptibility of thiosuccinimide linkages to retro-Michael deconjugation, leading to payload release and interception by the free cysteine residue on HSA.^37^ In marked contrast, the Fc–BisDVP–AF488 conjugate 9 showed no detectable transfer of fluorescence to plasma proteins throughout the 14 day incubation (Fig. 3D). The stability of the BisDVP conjugate is attributed to the formation of a robust thioether linkage and the incorporation of four covalent attachments between the Fc protein and linker-payload, providing enhanced resistance to exchange reactions. These data demonstrate that BisDVP linkages offer significantly greater plasma stability compared with maleimides. This property is particularly advantageous for the development of Fc conjugates, where premature release of therapeutic payloads could reduce efficacy and half-life.

**Fig. 4:**
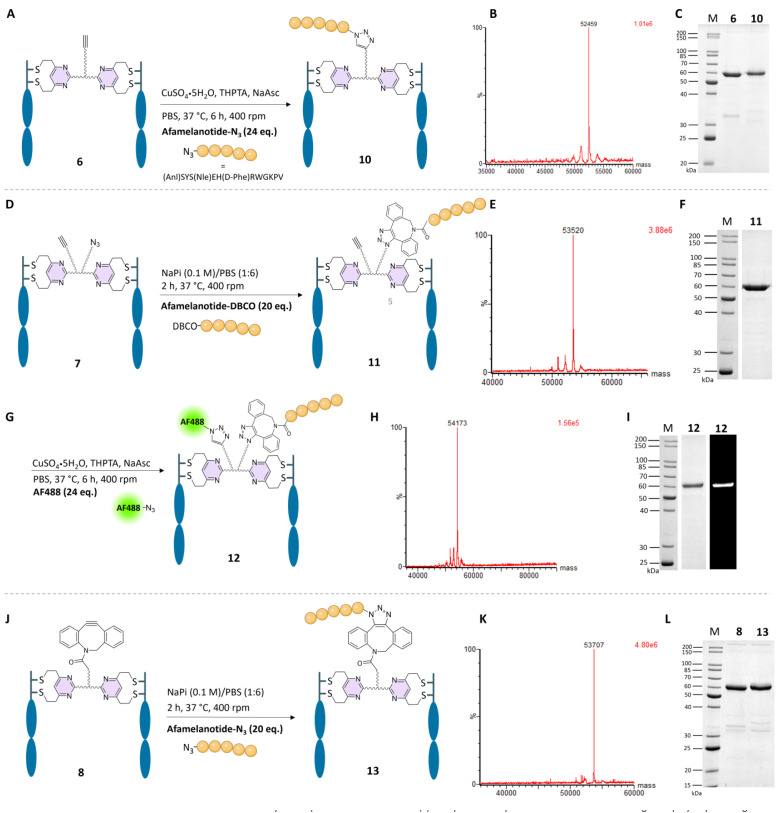
(A) CuAAC of 6 to form Fc–afamelanotide 10. (B) Analysis of 10 by LC-MS, deconvoluted MS; expected 52 449 Da and observed and 52 453 Da. Peaks < 30% not annotated for clarity – full spectrum available in SI. (C) Analysis of 10 by SDS-PAGE. (D) CuAAC of 7 to form 11. (E) Analysis of 11 by LC-MS, deconvoluted MS; expected 53 505 Da and observed 53 520 Da. (F) Analysis of 11 by SDS-PAGE. (G) CuAAC of 11 with AF488-azide to form 12. (H) Analysis of 12 by LC-MS, deconvoluted MS; expected 54 178 Da and observed 54 173 Da. (I) Analysis of 12 by SDS-PAGE with in-gel fluorescence of AF488 12. (J) SPAAC of 8 to form Fc–afamelanotide 13. (K) Analysis of 13 by LC-MS, deconvoluted MS; expected 53 709 Da and observed 53 707 Da. Peaks < 10% not annotated for clarity – full spectrum available in SI. (L) Analysis of 13 by SDS-PAGE. All SDS-PAGE using 12% polyacrylamide gel under reducing conditions. Lane: M = molecular weight marker. Afamelanotide-N_3_ sequence: XSYS(Nle)EH(D-Phe)RWGKPV where X is azido-lysine.

With an efficient strategy for generating Fc-conjugates established, we next aimed to produce an Fc–peptide conjugate. Afamelanotide, a synthetic α-melanocyte-stimulating hormone analogue and FDA-approved treatment for erythropoietic protoporphyria, was selected as the model peptide (Fig. 4).^38^ Despite its clinical efficacy, afamelanotide has a short plasma half-life (∼30 min), necessitating administration *via* subcutaneous implant, which can cause adverse local reactions and limit patient compliance.^39^ To enable site-specific attachment to the Fc–BisDVP–alkyne conjugate *via* CuAAC, afamelanotide was modified to include an azide handle by introducing the unnatural amino acid azido-l-lysine residue at the N-terminus. The active site responsible for stimulating the melanocortin 1 receptor is the ‘His-d-Phe-Arg-Trp’ sequence,^40,41^ thus addition of azido-lysine at the N-terminus should in theory not affect the therapeutic activity of the peptide. Afamelanotide-azide was synthesised by solid-phase peptide synthesis (SPPS) (see SI). Fc–BisDVP conjugate 6 was reacted with 24 equivalents of peptide under CuAAC conditions for 6 hours (Fig. 4A). After desalting and ultrafiltration, LC-MS (Fig. 4B) and SDS-PAGE (Fig. 4C) analysis showed that the desired Fc–afamelanotide conjugate 10 was formed in excellent conversion, homogeneity and purity. Next, afamelanotide-azide was reacted with a BisDBCO linker to form afamelanotide–DBCO (see SI). The afamelanotide–DBCO was then reacted with BisDVP conjugate 7 in a strain-promoted azide–alkyne click (SPAAC) reaction (Fig. 4D). LC-MS and SDS-PAGE analysis showed that the desired product 11 was formed in good purity (Fig. 4E and F). This Fc–afamelanotide conjugate 11 was then further reacted in a CuAAC reaction with Alexa Fluor™ 488 Azide (Fig. 4G). LC-MS and SDS-PAGE analysis (including in-gel fluorescence imaging) confirmed that the desired conjugate 12 was formed in excellent purity (Fig. 4H and I). This result highlights the ability of our system to orthogonally append two different payloads, allowing facile access to dual payload conjugates. Finally, Fc–BisDVP conjugate 8 was reacted with afamelanotide-azide in a SPAAC reaction (Fig. 4J). After desalting and ultrafiltration, LC-MS analysis revealed presence of the desired Fc–afamelanotide conjugate 13 with excellent conversion and purity (Fig. 4K). SDS-PAGE analysis also supports this observation with the presence of one main band on the gel (Fig. 4L). For completion, the SPAAC reaction was also carried out on Fc–BisDVP–DBCO obtained using Fc protein that was obtained by enzymatic digestion rather than recombinant expression (see SI). LC-MS analysis revealed formation of the desired Fc–afamelanotide conjugate in equally good conversion and purity as the recombinant form with SDS-PAGE supporting this observation (see SI, Fig S9).

Having demonstrated successful Fc–peptide conjugation using afamelanotide, we next applied this strategy to the glucagon-like peptide-1 (GLP-1) receptor agonist exenatide which is used for the treatment of type two diabetes.^42,43^ Exenatide was chosen due to the availability of GLP-1 assays to enable the biological evaluation of the Fc–peptide conjugate and validate this method. Previous work by Novo Nordisk on the development of an Fc–insulin conjugate highlighted the importance of the spacer length between the Fc protein and attachment site of the therapeutic.^31^ Novo Nordisk's work showed that an optimal activity for the Fc–insulin conjugate was observed when using a flexible PEG-like (GQEP)_19_ amino acid spacer (∼3.5 kDa), conversely when no spacer was incorporated, they saw a complete loss of activity. Based on these findings, we incorporated a similar design principle, introducing an azido-lysine residue into the exenatide sequence for CuAAC conjugation and two PEG12 linkers in between this residue and the exenatide sequence to provide sufficient separation between the Fc and the peptide's receptor-binding domain, minimising steric hindrance.

**Fig. 5:**
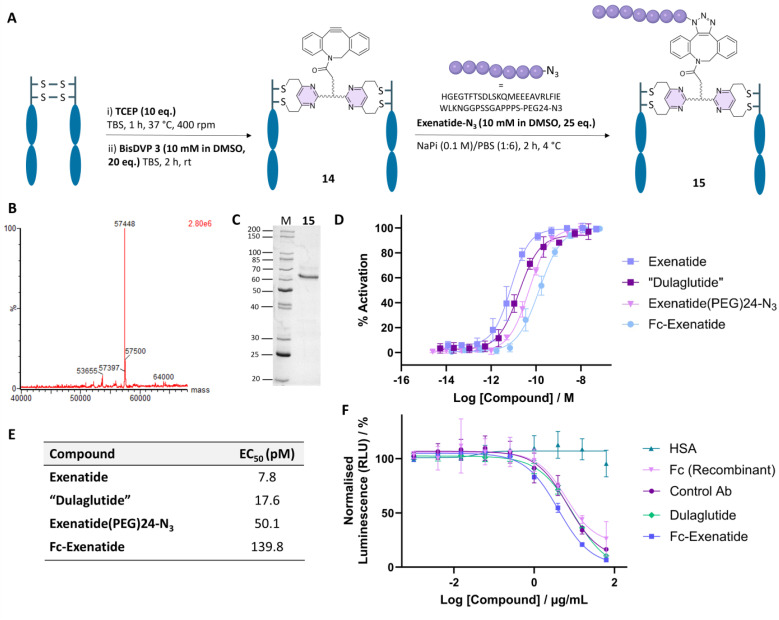
(A) Fc–exenatide formed *via* room temperature bioconjugation to give 14 and 4 °C SPAAC to give 15. (B) Deconvoluted MS of 15; expected 57 440 Da and observed 57 448 Da. (C) Analysis of Fc–exenatide 15 on 12% polyacrylamide gel under reducing conditions. Lanes: M = molecular weight ladder, 15 = Fc–exenatide 15. (D) % activation of GLP1R agonists determined by cAMP-based potency assay. (E) EC_50_ values for GLP1R agonists determined by cAMP-based potency assay. EC_50_ values (pM) were calculated using log(agonist) *vs.* response curve fit with variable slope (four parameters) in GraphPad Prism. (F) Graph of normalised luminescence representing FcRn binding of each species.

The SPAAC reaction was carried out between Fc–BisDVP–DBCO and exenatide-PEG24-azide however a small impurity was observed by LC-MS which was thought to be due to instability of the DBCO moiety on the Fc–BisDVP–DBCO. Due to the large size of the exenatide-PEG24-azide we believed that the SPAAC reaction proceeded more slowly than for the afamelanotide-azide and that this longer SPAAC reaction at 37 °C may promote degradation of the DBCO. It was also thought that the development of a one-pot reaction may accelerate the production of the conjugates. To this end, the reaction conditions were optimised to circumvent formation of the impurity – presumed to arise from degradation of the DBCO linker – and to enhance overall process efficiency. Fc protein was first reduced with 10 equivalents of TCEP, shaking at 37 °C for 1 hour as per standard conditions. After which the reduced Fc solution was cooled to room temperature. To the reduced Fc solution was added 20 equivalents of BisDVP 3. This was then allowed to react at room temperature with no shaking for 2 hours (Fig. 5A). The reaction mixture was then taken forward for a SPAAC reaction without purification. A SPAAC reaction was carried out with 25 equivalents of exenatide-azide, at 4 °C for 2 hours. LC-MS analysis revealed good conversion to the Fc–exenatide conjugate 15 (Fig. 5B) and pleasingly, no impurity was detected by LC-MS. This proved successful formation of Fc–exenatide species in a one-pot reaction and good conversion to the desired conjugate using a large therapeutic peptide moiety (∼5.5 kDa). A minor impurity was observed corresponding to over addition of linker, specifically an Fc protein with two BisDVP linkers attached and functionalised with two exenatide peptides (64 000 Da) (Fig. 5B). LC-MS analysis showed a significant amount of excess exenatide (∼5.5 kDa) remained in solution with the Fc–exenatide conjugate (57 kDa), after ultrafiltration with a molecular weight cut-off (MWCO) of 10 kDa (see SI). Therefore, protein A purification was employed, exploiting its specific binding to the Fc region to separate the conjugate from unbound exenatide. Following washing, elution, neutralisation, and buffer exchange into PBS, LC-MS analysis confirmed removal of the peptide impurity and yielded a Fc–exenatide conjugate in good purity (see SI, Fig. S1 and S2).

### Biological evaluation

#### cAMP potency assay

The biological activity of Fc–exenatide was assessed using a cyclic AMP (cAMP) assay,^44^ employing HTRF® technology.^45^ This method utilises Förster resonance energy transfer (FRET) to quantitatively monitor intracellular cAMP levels, serving as a surrogate marker for GLP-1 receptor-mediated GPCR activation by exenatide and its conjugates. In this assay, endogenous cAMP production competes with a fluorescently labelled tracer for antibody binding, resulting in an inverse correlation between fluorescence signal and agonist potency. This robust and widely applied method enabled comparative evaluation of exenatide, exenatide-PEG24-azide, Fc–exenatide, and a dulaglutide analogue (a clinically relevant GLP-1 receptor agonist and Fc fusion compound). As expected, exenatide exhibited the highest potency (EC50 = 7.8 pM) (Fig. 5D and E). Incorporation of the PEG24 spacer reduced potency (50.1 pM), likely due to conformational flexibility hindering receptor binding. Conjugation to Fc resulted in a further reduction (139.8 pM), consistent with steric effects; however, Fc–exenatide remained efficacious and only 18-fold less potent than exenatide. Importantly, its potency was comparable to approved GLP-1 therapeutics, within an order of magnitude of the dulaglutide-like analogue.^46,47^ Peptide conjugation to large biomolecules, including Fc domains, often results in reduced biological activity. In this work, we propose that the incorporation of a spacer, with an informed design, between the peptide and the Fc moiety enabled preservation of the pharmacological activity of the peptide. Greater retention of activity between the conjugate and the parent peptide may be achieved by incorporating a cleavable linker, enabling release of the peptide in its active form and reducing steric hindrance from the attached Fc protein. Overall, these data demonstrate that Fc-conjugation retains clinically relevant activity, supporting this approach as a viable strategy for extending half-life while maintaining therapeutic efficacy.

#### FcRn binding immunoassay

Having established the biological activity of Fc–exenatide, we next evaluated its interaction with the neonatal Fc receptor (FcRn), a key determinant of the prolonged half-life of Fc-based therapeutics. Although the Fc region was modified using the BisDVP linker, the conjugation site lies within the hinge region and is spatially distant from the FcRn binding interface (CH2–CH3 domain), suggesting minimal risk of interference. FcRn binding was quantified using the Lumit® FcRn Binding Immunoassay (Promega), a sensitive bioluminescent competition assay based on NanoBiT® technology.^48,49^ In this format, Fc-containing analytes compete with a labelled IgG tracer for receptor binding, producing a concentration-dependent reduction in luminescence. As expected, human serum albumin (HSA), which engages FcRn at an alternative site, showed no competitive binding, confirming assay specificity (Fig. 5F). Notably, Fc–exenatide displayed FcRn binding comparable to, and in some cases exceeding, that of control IgG, unmodified Fc, and a dulaglutide analogue (Fig. 5F). These results demonstrate that Fc–exenatide fully retains FcRn binding functionality despite chemical modification, thereby supporting its potential to benefit from FcRn-mediated recycling and achieve extended systemic half-life.

#### *In vivo* assessment

**Fig. 6:**
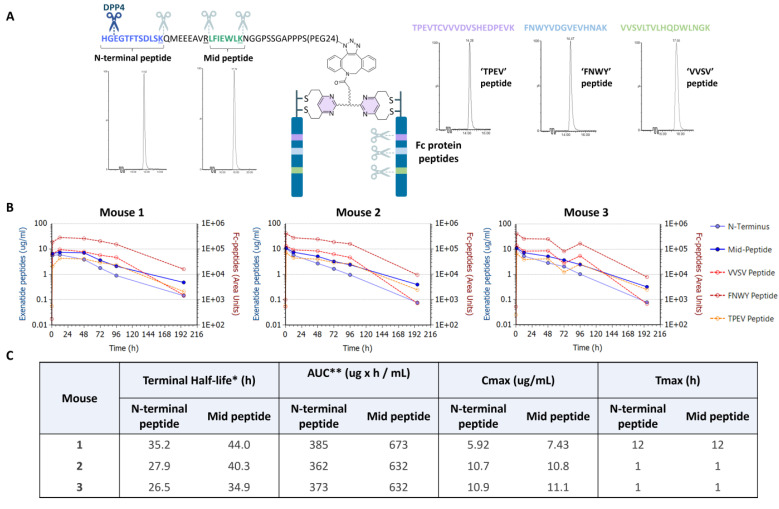
(A) Generation of peptides and their mass spectra from tryptic digest of Fc–exenatide 15 and cleavage of N-terminal peptide of exenatide in Fc–exenatide 15 by DPP4. (B) The PK profiles of each species after exclusion of 120 h timepoint in blood plasma measured from 1 hour to 196 hours. (C) Peptide exposure using a non-compartment approach (linear up-log down trapezoidal method) following iv bolus injections. *Last 3 datapoints used in calculation. **Sum of back-extrapolated (*t*_1_ to *t*_0_) and extrapolated area (*t*_last_ to infinity) is <10%.

The pharmacokinetics of the Fc–exenatide conjugate were evaluated *in vivo* to determine its circulation half-life and demonstrate the potential of this platform for peptide half-life extension. The study was conducted using C57BL/6J female mice, with three treatment groups receiving a single intravenous dose *via* tail vein injection of exenatide (3 mice), Fc–exenatide (3 mice), or PBS (2 mice) as a vehicle control. Both exenatide and Fc–exenatide were administered at 25 nmol kg^−1^, calculated based on body weight at dosing. Blood samples were collected at multiple timepoints over eight days, and plasma was stored at −80 °C prior to analysis. Exenatide was detectable only at the 1 hour timepoint (see SI), consistent with its known short half-life in mice (∼1.7–1.9 h).^50,51^ In contrast, Fc–exenatide was detected up to 196 hours (Fig. 6). Extraction from the mouse plasma was carried out using anti-Fc antibody immobilised on beads, and then tryptic digestion to generate peptides for LC-MS/MS peptide mapping analysis. The Fc protein backbone generates 3 peptides which can be detected. Exenatide generates 2 peptides to be detected, one from the middle of the sequence (mid-peptide) and one from the N-terminus (N-terminal peptide) (Fig. 6). LC-MS/MS analysis confirmed the presence of both the mid-sequence and N-terminal exenatide peptides, indicating retention of the intact, active molecule. However, evidence of N-terminal cleavage by dipeptidyl peptidase-4 (DPP4) was observed, consistent with known GLP-1 analogue metabolism (see SI).^52^ The calculated half-life of intact Fc–exenatide was 27–35 hours, with a calculated half-life of the mid peptide being 35–44 hours, meaning the half-life could be further improved if the peptide incorporated a non-natural/stabilised amino acid at the N-terminal to enable DPP4-resistance. Comparison with dulaglutide provides useful context for these data. While dulaglutide exhibits a ∼4–5 day half-life in humans, preclinical studies report shorter half-lives in rodents (∼1.5–2 days),^53^ reflecting known species differences in FcRn-mediated recycling. In this context, the half-life observed for Fc–exenatide (∼27–44 h) is consistent with expected behaviour for Fc-containing constructs in rodent models. This suggests that the pharmacokinetic profile is not indicative of instability arising from the disulfide re-bridging strategy. Importantly, a clear and significant extension in half-life relative to native exenatide is achieved, supporting the effectiveness of the conjugation approach and its potential for further optimisation. These results provide the first *in vivo* proof-of-concept for the Fc–BisDVP–peptide conjugation strategy. These findings support the use of this approach for generating long-acting peptide therapeutics.

## Conclusions

In summary, we have established an approach that allows for precise, site-selective modification of Fc proteins, enabling the creation of highly uniform Fc-conjugates without relying on recombinant methods. This strategy is based on reducing and re-bridging Fc proteins using a single bridging linker, which supports efficient site-specific bioconjugation and yields Fc-conjugates of high stability and purity. Additionally, we incorporated an azido-l-lysine residue into the peptide sequence, which facilitated covalent attachment to Fc–linker conjugates through click chemistry with either therapeutic peptides and/or fluorophores. The resulting Fc-conjugates remained stable in human plasma. Biological activity studies of exenatide and Fc–exenatide showed that potency of the peptide was retained, attributed to the long linker length between the peptide and Fc domain. Moreover, pharmacokinetic evaluation *in vivo* revealed that the Fc–exenatide conjugate exhibited a significantly extended circulation half-life compared with the exenatide peptide alone.

We anticipate that this platform will be broadly applicable to a diverse range of Fc proteins and therapeutic agents, minimising the need for extensive case-specific optimisation and offering strong potential to streamline the development and scale-up of Fc conjugates for pharmaceutical applications.

## Ethics approval

All animal experiments were conducted under the authority of the UK Home Office in compliance with the Animals (Scientific Procedures) Act 1986 (ASPA), under project license number PP5753595. The study was carried out at the approved establishment in the University of Cambridge and adhered to the 3Rs principle (Replacement, Reduction, Refinement). All procedures were performed in accordance with institutional guidelines for the care and use of laboratory animals (Animal Welfare & Ethical Review Body).

## Author contributions

M. P., E. T., M. A. P., R. K., and D. T. were involved in investigation. D. R. S., J. S. P., A.-C. N., M. A. P., F. M. D., and S. J. W. were involved in supervision. F. M. D. was involved in early conceptualisation. D. H. was involved in *in vivo* data analysis. The manuscript was written through contributions of all authors. All authors have given approval to the final version of the manuscript.

## Conflicts of interest

J. S. P., A.-C. N., M. A. P., E. T., D. H., and F. M. D. are employees of AstraZeneca and may own stock or options of AstraZeneca.

## Supplementary Material

SC-017-D6SC02646J-s001

## Data Availability

The data supporting this article have been included as part of the supplementary information (SI). Supplementary information is available. See DOI: https://doi.org/10.1039/d6sc02646j.
