# Supplementary material for: Boosting peptide half-life: enabling efficient generation of Fc–peptide conjugates
Source: Chem Sci. 2026 May 29;17(27):13454–64. doi: 10.1039/d6sc02646j (PMC13244299; doi:10.1039/d6sc02646j)
Supplement: SC-017-D6SC02646J-s001 [file SC-017-D6SC02646J-s001.pdf]

Supplementary Information (SI) for Chemical Science.
This journal is © The Royal Society of Chemistry 2026

Boosting Peptide Half-Life: Enabling Efficient Generation of Fc-Peptide Conjugates

Mahri Park*[a], Friederike M. Dannheim[a], Monika A. Papworth[b], Richard Kay[c], Elpida Tsonou[d], Daniel Trajkovski[e], Stephen Walsh[a], Daniel Hovdal[f], Thomas Wharton,[a] Anne-Chloe Nassoy[b], Jeremy S. Parker[g], David Spring*[a]

[a]Department of Chemistry, University of Cambridge, Cambridge, UK [b]Oncology R&D, AstraZeneca, UK [c]Institute of Metabolic Science, Department of Clinical Biochemistry, University of Cambridge UK [d]BioPharmaceuticals R&D, AstraZeneca, UK [e]Department of Surgery, University of Cambridge, UK [f]Preclinical and translational PK & PKPD specialist (DMPK), AstraZeneca, Gothenburg, Sweden [g]Early Chemical Development, Pharmaceutical Development, R&D, AstraZeneca, Macclesfield, UK.

Supplementary Information

Contents

Chemistry 3
- Chemistry Experimental General Information 3
- Chemical Synthesis 5
- Peptide Synthesis 20

Bioconjugation 22
- Peptide Conjugation General Information 22
- Protein A purification of Fc-Exenatide to remove Exenatide 24
- Fc Production *via* Trastuzumab Digestion 26
- Fc Production *via* Recombinant Expression 28
- Bioconjugation Experimental 30
- Selectivity Control Experiments 40

Biological Studies 45

In Vivo Study 48

Mass Spectrometry Traces 67

NMR Spectra 87

HPLC Traces 106

Chemistry

Chemistry Experimental General Information

All solvents and reagents were used as received unless otherwise stated. Ethyl acetate, methanol, dichloromethane, acetonitrile and toluene were distilled from calcium hydride. Diethyl ether was distilled from a mixture of lithium aluminium hydride and calcium hydride. Petroleum ether (PE) refers to the fraction between 40 – 60 °C upon distillation. Tetrahydrofuran was dried using Na wire and distilled from a mixture of lithium aluminium hydride and calcium hydride with triphenylmethane as indicator.

Non-aqueous reactions were conducted under a stream of dry nitrogen using oven-dried glassware. Temperatures of 0 °C were maintained using an ice-water bath. Room temperature (rt) refers to ambient temperature.

Yields refer to spectroscopically and chromatographically pure compounds unless otherwise stated. Reactions were monitored by thin layer chromatography (TLC) or liquid chromatography mass spectroscopy (LC-MS). TLC was performed using glass plates precoated with Merck silica gel 60 F254 and visualized by quenching of UV fluorescence (λmax = 254 nm) or by staining with potassium permanganate. Retention factors (Rf) are quoted to 0.01. LC-MS was carried out using a Waters ACQUITY H-Class UPLC with an ESCi Multi-Mode Ionisation Waters SQ Detector 2 spectrometer using MassLynx 4.2 software; ESI refers to the electrospray ionisation technique; LC system: solvent A: 2 mM NH_4OAc in H_2 O/MeCN (95:5); solvent B: MeCN; solvent C: 2% formic acid; column: ACQUITY UPLC® CSH C18 (2.1 mm × 50 mm, 1.7 µm, 130 Å) at 40 °C; gradient: 5 – 95 % B with constant 5 % C over 1 min at flow rate of 0.6 mL/min; detector: PDA eλ Detector 220 – 800 nm, interval 1.2 nm.

Flash column chromatography was carried out using slurry-packed Merck 9385 Kieselgel 60 SiO_2 (230-400 mesh) or Combiflash Rf200 automated chromatography system with Redisep® reverse-phase C18-silica flash columns (20-40 µm).

Analytical high performance liquid chromatography (HPLC) was performed on Agilent 1260 Infinity machine, using a Supelcosil™ ABZ+PLUS column (150 mm × 4.6 mm, 3 µm) with a linear gradient system (solvent A: 0.05% (v/v) TFA in H_2O;solvent B: 0.05% (v/v) TFA in MeCN) over 20 min at a flow rate of 1 mL/min, and UV detection (λmax = 220 – 254 nm).

Infrared (IR) spectra were recorded neat on a Perkin-Elmer Spectrum One spectrometer with internal referencing. Selected absorption maxima (νmax) are reported in wavenumbers (cm-1) with peak intensity reported as follows: w = weak; m = medium; s = strong.

1H and 13C nuclear magnetic resonance (NMR) were recorded using an internal deuterium lock on Bruker DPX-400 (400 MHz, 101 MHz), Bruker Avance 400 QNP (400 MHz, 101 MHz) and Bruker Avance 500 Cryo Ultrashield (500 MHz, 126 MHz). In 1H NMR, chemical shifts (δH) are reported in parts per million (ppm), to the nearest 0.01 ppm and are referenced to the residual non-deuterated solvent peak ($CDCl_3$: 7.26, CD_3OD: 3.31). Coupling constants (J) are reported in Hertz (Hz) to the nearest 0.1 Hz. Data are reported as follows: chemical shift, multiplicity (s = singlet; d = doublet; t = triplet; q = quartet; quint = quintet; m = multiplet; or as a combination of these, e.g. dd, dt etc.), integration and coupling constant(s). In 13C NMR, chemical shifts (δC) are quoted in

ppm, to the nearest 0.1 ppm, and are referenced to the residual non-deuterated solvent peak ($CDCl_3$: 77.16, CD_3OD: 49.00). Conformational isomers (rotamers) are produced by restricted rotation around a bond.

High resolution mass spectrometry (HRMS) measurements were recorded with a Micromass Q-TOF mass spectrometer or a Waters LCT Premier Time of Flight mass spectrometer. Mass values are reported within the error limits of ±5 ppm mass units. ESI refers to the electrospray ionisation technique.

Chemical Synthesis

Scheme S1. Synthesis of BisDVP 1.

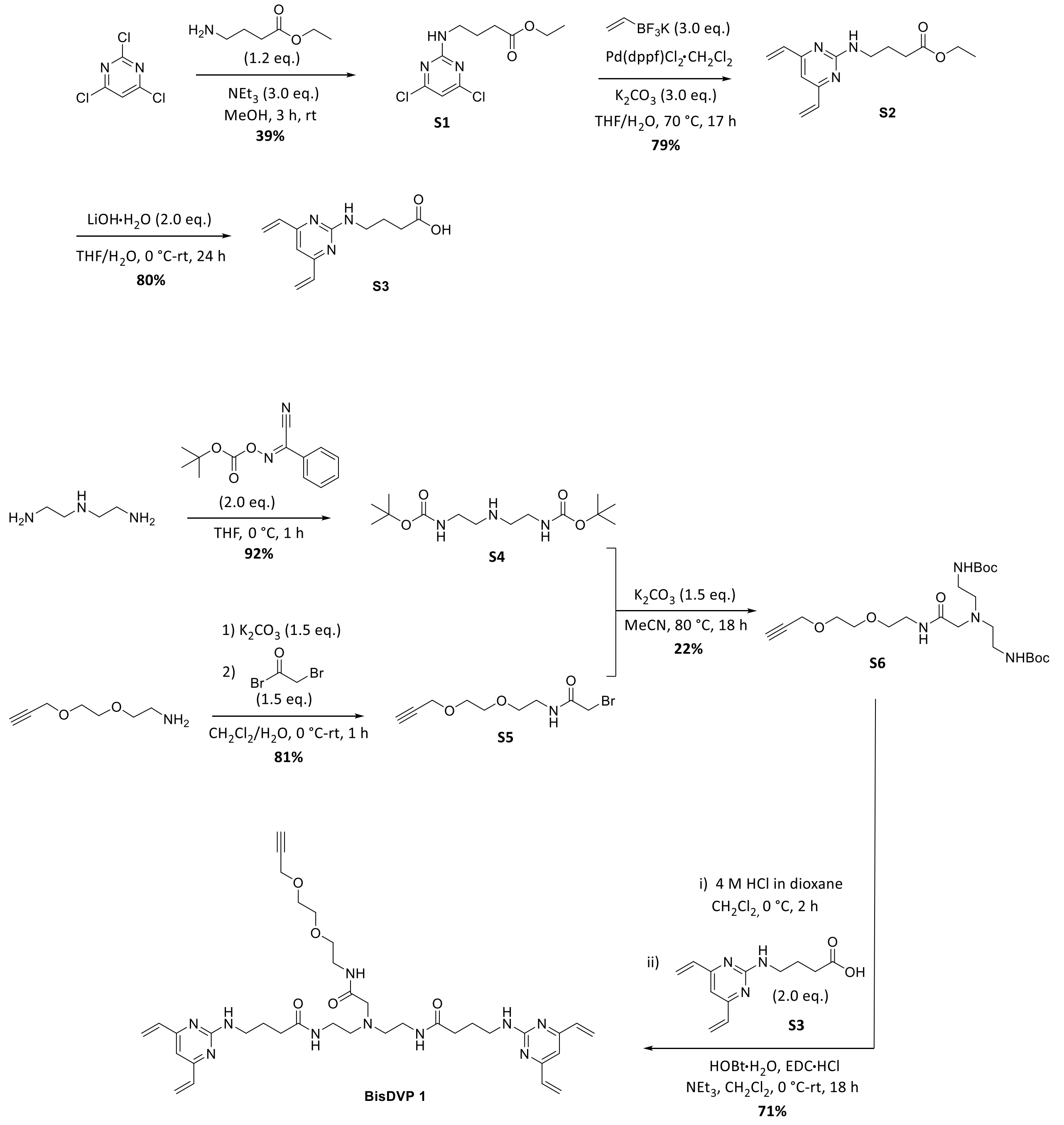

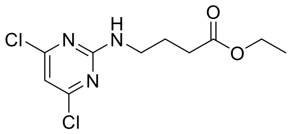

Ethyl 4-((4,6-dichloropyrimidin-2-yl)amino)butanoate (S1) To a solution of ethyl 4-aminobutyrate hydrochloride (3.5 g, 20.9 mmol) and triethylamine (7.24 mL, 52.2 mmol) in methanol (174 mL) was added dropwise 2,4,6-trichloropyrimidine (2.0 mL, 17.4 mmol). After addition, the reaction mixture was kept stirring at room temperature for 3 h before being concentrated *in vacuo*. The residue was purified by column chromatography (0-20% EtOAc/PE) to provide the title compound as a white crystalline solid (1.89 g, 6.80 mmol, 39%).

δ_H (400 MHz, CD_3OD) 6.65 (s, 1H), 4.12 (q, 2H, *J* = 7.1 Hz), 3.40 (t, 2H, *J* = 6.8 Hz), 2.38 (t, 2H, *J* = 7.3 Hz), 1.89 (qn, 2H, *J* = 7.0 Hz), 1.24 (t, 3H, *J* = 7.1 Hz); **δ_C** (101 MHz, CD_3OD) 175.0, 163.4, 163.1/162.8, 108.8, 61.6, 41.5, 32.3, 25.5, 14.5; **LRMS** (ESI) *m/z* found $[M+H]^+$ 278.1, $C_{10}H_{14}{}^{35}Cl_2N_3O_2^+$ required 278.0.

These data are consistent with those previously reported.[1,2]

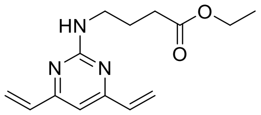

Ethyl 4-((4,6-divinylpyrimidin-2-yl)amino)butanoate (S2) A mixture of ethyl 4-((4,6-dichloropyrimidin-2-yl)amino)butanoate S**1** (1.89 g, 6.79 mmol), potassium vinyltrifluoroborate (2.73 g, 20.4 mmol), $PdCl_2(dppf)\cdot CH_2Cl_2$ (833 mg, 1.02 mmol) and K_2CO_3 (2.82 g, 20.4 mmol) in THF (81 mL) and H_2O (8.1 mL) was heated to 70 °C under nitrogen for 17 h. The reaction mixture was cooled to ambient temperature, filtered through Celite®, and concentrated *in vacuo*. The residue was purified by column chromatography (0-20% EtOAc/PE) to yield the product as a yellow oil (1.39 g, 5.32 mmol, 79%).

δ_H (400 MHz, $CDCl_3$) 6.57 (dd, 2H, *J* = 10.6, 17.3 Hz), 6.53 (s, 1H), 6.37 (d, 2H, *J* = 17.3 Hz), 5.57 (d, 2H, *J* = 10.6 Hz), 5.34 (s, 1H), 4.12 (q, 2H, *J* = 7.1 Hz), 3.54 (q, 2H, *J* = 6.5 Hz), 2.41 (t, 2H, *J* = 7.3 Hz), 1.96 (qn, 2H, *J* = 7.1 Hz), 1.24 (t, 3H, *J* = 7.1 Hz); **δ_C** (101 MHz, $CDCl_3$) 173.4, 163.6, 162.4, 135.7, 121.6, 105.7, 60.4, 40.7, 31.7, 25.1, 14.2; **LRMS** (ESI) *m/z* found $[M+H]^+$ 262.9, $C_{14}H_{18}N_3O_3^+$ required 262.3.

These data are consistent with those previously reported.[1]

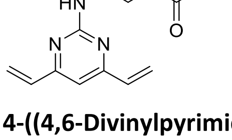

4-((4,6-Divinylpyrimidin-2-yl)amino)butanoic acid (S3) To a solution of ethyl 4-((4,6-divinylpyrimidin-2-yl)amino)butanoate **S2** (550 mg, 2.10 mmol) in THF (20 mL) and H_2O (20 mL) at 0 °C was added $LiOH\cdot H_2O$ (177 mg, 4.20 mmol). The mixture was stirred at room temperature for 24 h, and then diluted with H_2O (30 mL) and

washed with Et_2O (30 mL). The aqueous layer was adjusted to pH 4 using 1 M HCl and then extracted with CH_2Cl_2 (8 x 30mL). The organic layer was dried over Na_2SO_4, filtered, and concentrated *in vacuo* to yield the product as a yellow solid (390 mg, 1.67 mmol, 80%).

δ_H (400 MHz, CD_3OD) 6.70 (s, 1H), 6.61 (dd, 2H, *J* = 10.7, 17.4 Hz), 6.37 (d, 2H, *J* = 17.4 Hz), 5.57 (d, 2H, *J* = 10.7 Hz), 3.48 (t, 2H, *J* = 6.8 Hz), 2.38 (t, 2H, *J* = 7.3 Hz), 1.92 (qn, 2H, *J* = 7.1 Hz); **δ_C** (101 MHz, CD_3OD) 177.4, 165.3, 164.0, 137.0, 122.1, 105.7, 41.5, 32.4, 26.1; **LRMS** (ESI) *m/z* found $[M+H]^+$ 234.0, $C_{12}H_{14}N_3O_2^+$ required 234.1.

These data are consistent with those previously reported.[1]

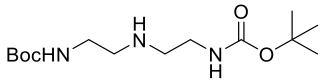

Di-*tert*-butyl (azanediylbis(ethane-2,1-diyl))dicarbamate (S4) A solution of diethylenetriamine (0.48 mL, 4.2 mmol) and triethylamine (1.8 mL, 12.6 mmol) in THF (25 mL) was stirred for 20 minutes at 0 °C under nitrogen. To this solution was added dropwise a solution of Boc-ON (2.1 g, 8.30 mmol) in THF (25 mL). The reaction was stirred at 0 °C under nitrogen for 2 h and then concentrated *in vacuo*. The crude yellow oil was dissolved in CH_2Cl_2 and sequentially washed with 10% aq. NaOH, water and brine. The organic phase was dried over Na_2SO_4 and concentrated *in vacuo*. Purification by column chromatography (10% MeOH/CH_2Cl_2) yielded the product as a colourless gum (1.17 g, 3.86 mmol, 92% yield).

δ_H (400 MHz, $CDCl_3$) 5.08 (br s, 2H) 3.20-3.15 (m, 4H), 2.69 (t, 4H, J = 5.6 Hz), 1.40 (s, 18H); **δ_C** (101 MHz, CD_3OD) 156.2, 79.2, 48.8, 40.2, 28.4; **LRMS** (ESI) *m/z* found $[M+H]^+$ 304.5, $C_{14}H_{30}N_3O_4^+$ required 304.2.

These data are consistent with those previously reported.[2]

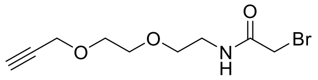

2-Bromo-*N*-(2-(2-(prop-2-yn-1-yloxy)ethoxy)ethyl)acetamide (S5) To a solution of 2-(2-(prop-2-yn-1-yloxy)ethoxy)ethan-1-amine (794 mg, 5.55 mmol) in CH_2Cl_2 (11 mL) was added a solution of K_2CO_3 (1.15 g, 8.32 mmol) in H_2O (18 mL) and cooled to 0 °C. A solution of bromoacetyl bromide in CH_2Cl_2 (10 mL) was added dropwise over 30 min. The reaction was stirred at 0 °C for 30 min followed by a further 30 min at rt. Upon completion the reaction was diluted with H_2O (10 mL) and the organic layer was washed with H_2O (3 x 20 mL). The aqueous layer was extracted with CH_2Cl_2 (3 x 20 mL). The organic layers were combined, dried with Na_2SO_4 and concentrated *in vacuo* to afford the title compound as a colourless oil (1.19 g, 4.50 mmol, 81%).

R_f 0.58 (SiO_2, 10% MeOH/CH_2Cl_2); **ν_{max}** (neat/cm^{-1}) 3286 (m, N-H), 2873 (m, C-H), 2114 (w, C≡C), 1654 (s, C=O), 1086 (s, C-O); **δ_H** (400 MHz, $CDCl_3$) 6.90 (br s, 1H), 4.22 (d, 2H, *J* = 1.8 Hz), 3.87 (s, 2H), 3.72-3.66 (m, 4H) 3.60 (t,

2H, J = 5.0 Hz), 3.50 (q, 2H, J = 5.2 Hz), 2.45 (t, 1H, J = 2.0); **δ_C** (101 MHz, $CDCl_3$) 165.6, 79.4, 74.8, 70.1, 69.3, 69.0, 58.5, 39.9, 29.1; **HRMS** (ESI) *m/z* found $[M+H]^+$ 264.0231, $C_9H_{15}O_3BrN^+$ required 264.0235.

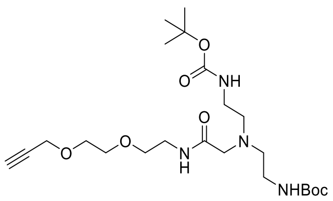

bis-*N*-Boc-amine intermediate (PEG2-alkyne) (S6) A solution of di-tert-butyl (azanediylbis(ethane-2,1-diyl))dicarbamate **S4** (70 mg 0.231 mmol), 2-bromo-N-(2-(2-(prop-2-yn-1-yloxy)ethoxy)ethyl)acetamide **S5** (61 mg, 0.231 mmol) and K_2CO_3 (48 mg, 0.347 mmol) in MeCN (6 mL) was heated under reflux at 80 °C for 18 h. Upon completion, the reaction mixture was concentrated *in vacuo*. The residue was purified by column chromatography (0-10% MeOH/CH_2Cl_2) to afford the title compound as a colourless oil (25 mg, 0.051 mmol, 22% yield).

R_f 0.51 (SiO_2, 10% MeOH/CH_2Cl_2); **ν_{max}** (neat/cm^{-1}) 3338 (m, N-H), 2933 (m, C-H), 2114 (w, C≡C), 1690 (s, C=O), 1166 (s, C-O); **δ_H** (400 MHz, $CDCl_3$) 7.46 (br s, 1H), 5.68 (br s, 2H), 4.08 (d, 2H, J = 2.2 Hz), 3.62-3.57 (m, 4H), 3.53 (t, 2H, J = 5.2 Hz), 3.39 (q, 2H, J = 5.5 Hz), 3.13-3.09 (m, 4H), 3.02 (s, 2H), 2.51 (t, 4H, J = 5.3 Hz), 2.40 (t, 1H, J = 2.3 Hz), 1.37 (s, 18H); **δ_C** (400 MHz, $CDCl_3$) 171.1, 156.6, 79.2, 79.1, 74.9, 69.7, 69.4, 69.0, 58.6, 58.2, 55.0, 38.9, 38.4, 28.5; **HRMS** (ESI) *m/z* found $[M+H]^+$ 487.3135, $C_{23}H_{43}O_7N_4^+$ required 487.3132.

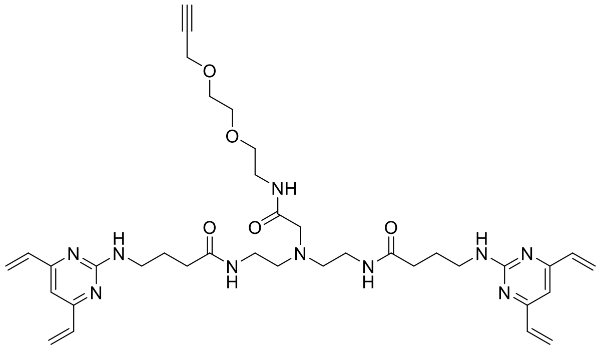

***bis*-Divinylpyrimidine Alkyne Linker (BisDVP 1)** To a solution of **S6** (200 mg, 0.410 mmol) in CH_2Cl_2 (1.4 mL) at 0 °C was added HCl (4M in dioxane, 3.4 mL). The reaction was stirred under nitrogen for 2 h and then concentrated *in vacuo* to yield the desired amine hydrochloride salt as a white solid. The amine hydrochloride salt was re-dissolved in CH_2Cl_2 (6 mL) and cooled to 0 °C. A solution of 4-((4,6-divinylpyrimidin-2-yl)amino)butanoic acid **S3** (191 mg, 0.820 mmol), triethylamine (571 µL, 4.10 mmol), EDC•HCl (314 mg, 1.64 mmol) and HOBt monohydrate (222 mg, 1.64 mmol) in CH_2Cl_2 (14 mL) at 0 °C was added. The reaction was stirred under nitrogen for 18 h and then diluted with CH_2Cl_2 (30 mL), washed with brine (30 mL), dried over Na_2SO_4, and concentrated *in vacuo*. Purification by column chromatography (0-10% MeOH/CH_2Cl_2) yielded the product as a yellow oil (209 mg, 0.290 mmol, 71% yield).

R_f 0.42 (SiO_2, 10% MeOH/CH_2Cl_2); **ν_{max}** (neat/cm^{-1}) 3293 (m, N-H), 2929 (m, C-H), 2112 (w, C≡C), 1638 (s, C=O), 1538 (s, C=C); 1085 (s, C-O); **δ_H** (400 MHz, $CDCl_3$) 7.19 (t, 2H, *J* = 4.9 Hz), 6.56 (dd, 4H, *J* = 10.6, 17.3 Hz), 6.50 (s, 2H), 6.33 (d, 4H, *J* = 17.3 Hz), 6.12 (t, 2H, *J* = 5.4 Hz), 5.54 (d, 4H, *J* = 10.6 Hz) 4.12 (d, 2H, *J* = 1.6 Hz), 3.66-3.64 (m, 2H), 3.58-3.56 (m, 2H), 3.52 (q, 4H, *J* = 6.0 Hz), 3.46 (t, 2H, J = 4.5 Hz), 3.34-3.25 (m, 6H, H11), 3.00 (s, 2H), 2.55 (t, 4H, *J* = 4.6 Hz), 2.47 (s, 1H), 2.34 (t, 4H, *J* = 7.5 Hz), 1.95 (qn, 4H, *J* = 6.8 Hz); **δ_C** (101 MHz, $CDCl_3$) 174.1, 170.7, 163.7, 162.7, 135.9, 121.4, 105.2, 78.8, 75.4, 69.9, 69.8, 69.0, 58.4, 57.7, 54.2, 40.8, 39.2, 37.0, 33.6, 25.9; **HRMS** (ESI) *m/z* found $[M+H]^+$ 717.4208, $C_{37}H_{53}O_5N_{10}^+$ required 717.4200.

Scheme S2. Synthesis of BisDVP 2.

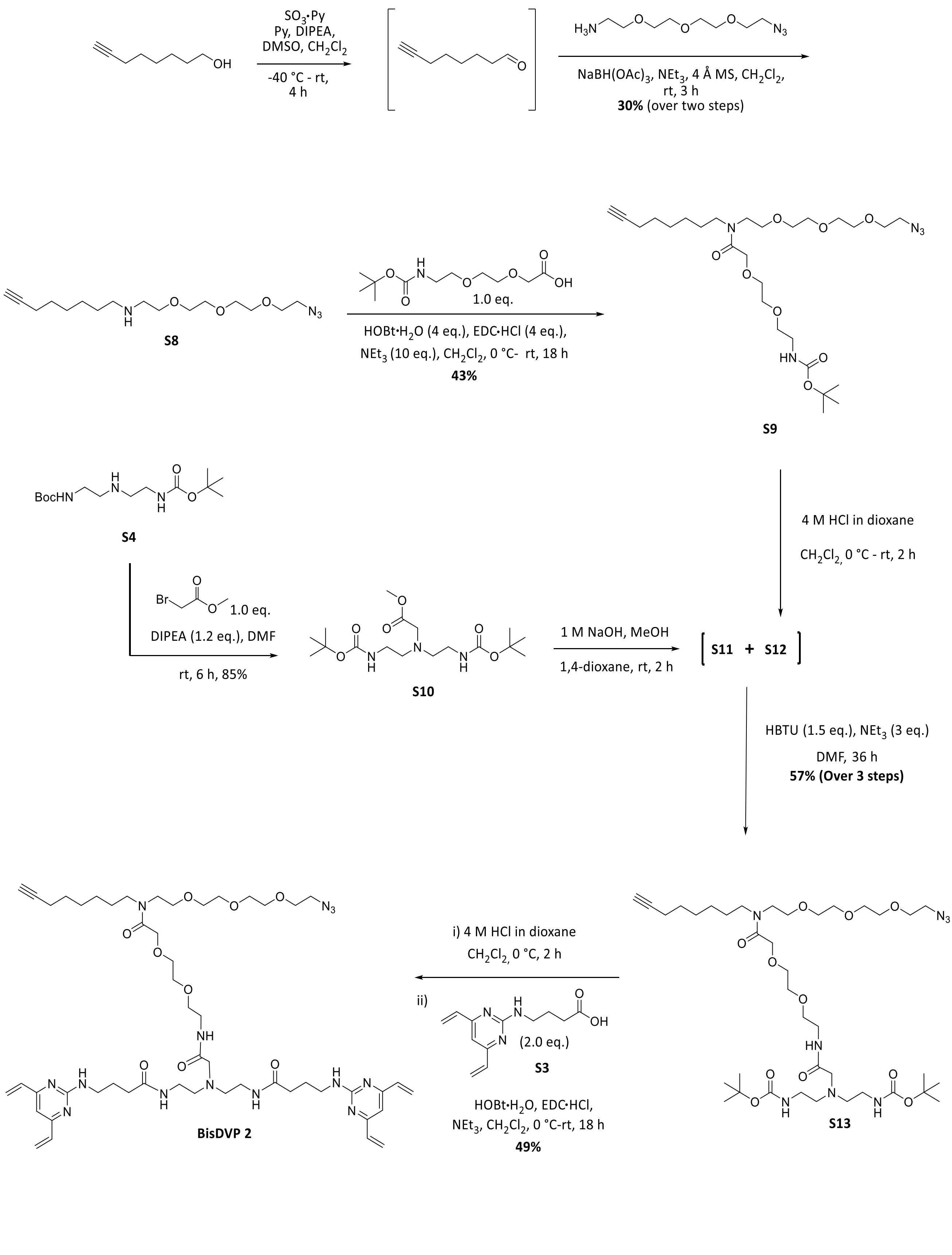

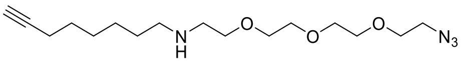

N-(2-(2-(2-(2-Azidoethoxy)ethoxy)ethoxy)ethyl)oct-7-yn-1-amine (S8) A solution of sulfur trioxide pyridine complex (756 mg, 4.75 mmol) and pyridine (0.38 mL, 4.75 mmol) in CH_2Cl_2 (1.55 mL) was stirred at rt for 5 min under nitrogen. To this solution was added DIPEA (0.83 mL, 4.75 mmol) and DMSO (1.13 mL, 15.85 mmol) and stirred at rt for 5 min under nitrogen. The mixture was cooled to –40 °C and to this was added oct-7-yn-1-ol (200 mg, 1.58 mmol). The reaction was stirred for 2 h under nitrogen warming slowly to –20 °C. Upon completion, the reaction was acidified to pH 3 with 1 M HCl and diluted with CH_2Cl_2 (10 mL). The layers were separated, and the aqueous phase was extracted with further CH_2Cl_2 (2 × 20 mL). The combined organic fractions were washed with brine (2 × 50 mL), dried ($MgSO_4$) and concentrated *in vacuo*. The crude oil was immediately redissolved in MeOH (1.24 mL). A separate solution of 2-(2-(2-(2-azidoethoxy)ethoxy)ethoxy)ethan-1-amine (517 mg, 2.37 mmol) and 4 Å molecular sieves in CH_2Cl_2 (12 mL) was stirred at rt for 5 min under nitrogen. To this solution was added the aldehyde solution, followed by $NaBH(OAc)_3$ (1.01 g, 4.75 mmol) and the reaction mixture stirred at rt for 3 h. Upon completion, the reaction mixture was filtered, diluted with $NaHCO_3$ (sat. aq., 15 mL) and extracted with CH_2Cl_2 (3 × 25 mL). The combined organic layers were washed with brine (50 mL), dried ($MgSO_4$) and concentrated *in vacuo*. The crude residue was purified by flash column chromatography (0-10% MeOH/CH_2Cl_2) to yield the title compound (149 mg, 0.456 mmol, 30% over two steps) as a colourless oil.

R_f 0.32 (SiO_2, 10% MeOH/CH_2Cl_2); **ν_{max}** (neat/cm^{-1}) 3297 (m, N-H), 2865 (m, C-H), 2102 (s, C≡C), 1111 (s, C-O); **δ_H** (400 MHz, $CDCl_3$) 3.81 (t, 2H, J = 5.1 Hz), 3.68-3.65 (m, 10H), 3.40 (t, 2H, J = 5.0 Hz), 3.06 (t, 2H, J = 5.1 Hz), 2.89 (t, 2H, J = 5.1 Hz), 2.16 (td, 2H, J = 10.3, 2.6 Hz), 1.93 (t, 1H, J = 2.6 Hz), 1.76 (qn, 2H, J = 7.6 Hz), 1.54-1.34 (m, 7H); **δ_C** (101 MHz, $CDCl_3$) 84.5, 70.7, 70.6, 70.5, 70.4, 70.1, 68.5, 67.4, 50.8, 48.6, 47.7, 28.4, 28.3, 27.2, 26.5, 18.4; **HRMS** (ESI) m/z found $[M+H]^+$ 327.2408, $C_{16}H_{31}N_4O_3^+$ required 327.2396.

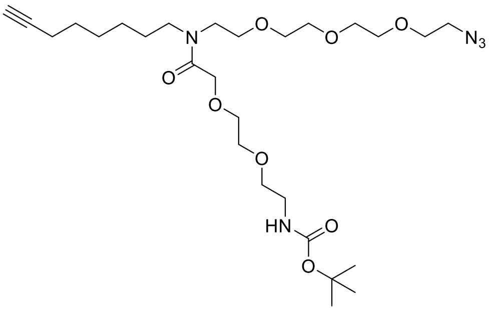

***tert*-Butyl (20-azido-9-(oct-7-yn-1-yl)-8-oxo-3,6,12,15,18-pentaoxa-9-azaicosyl)carbamate (S9)** Compound **S8** (91.6 mg, 0.28 mmol) was dissolved in CH_2Cl_2 (4 mL) and cooled to 0 °C. A solution of 2,2-Dimethyl-4-oxo-3,8,11-trioxa-5-azatridecan-13-oic acid (73.7 mg, 0.28 mmol), triethylamine (0.39 mL, 2.80 mmol), EDC•HCl (215 mg, 1.12 mmol) and HOBt monohydrate (171 mg, 1.12 mmol) in CH_2Cl_2 (9.5 mL) at 0 °C was added. The reaction was stirred under nitrogen for 18 h and then diluted with CH_2Cl_2 (30 mL), washed with brine (30 mL), dried over Na_2SO_4, and concentrated *in vacuo*. Purification by column chromatography (0-5% MeOH/CH_2Cl_2) yielded the product as a colourless oil (102.7 mg, 3.25 mmol, 64% yield).

R_f 0.38 (SiO_2, 5% MeOH/CH_2Cl_2); **ν_{max}** (neat/cm^{-1}) 3304 (m, N-H), 2865 (m, C-H), 2102 (s, C≡C), 1644 (s, C=O), 1105 (s, C-O); **δ_H** (500 MHz, $CDCl_3$) 5.12-5.09 (m, 1H), 4.25-4.19 (m, 2H), 3.69-3.55 (m, 16H), 3.52-3.42 (m, 4H), 3.36 (t, 2H, *J* = 4.9 Hz), 3.34-3.24 (m, 4H), 2.18-2.13 (m, 2H), 1.94-1.91 (m, 1H), 1.57-1.48 (m, 4H), 1.43-1.36 (m, 11H), 1.30-1.24 (m, 2H); **δ_C** (125.7 MHz, $CDCl_3$) 169.4/169.0, 156.0, 85.5/84.3, 79.1, 70.7-70.0, 69.7, 69.2/69.1, 68.5/68.2, 50.6, 48.1/46.0, 46.6/45.7, 40.4, 28.7, 28.4, 28.3, 27.3, 26.4, 18.3 Conformational isomers (rotamers) present; **HRMS** (ESI) m/z found $[M+H]^+$ 572.3661, $C_{27}H_{50}N_5O_8^+$ required 572.3659.

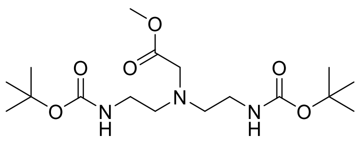

Methyl bis(2-((tert-butoxycarbonyl)amino)ethyl)glycinate (S10) To a solution of **S4** (451 mg, 1.49 mmol) in DMF (3 mL) was added a solution of DIPEA (0.31 mL, 1.79 mmol) and methyl bromoacetate (0.21 mL, 2.24 mmol) in DMF (3 mL). The reaction was stirred at rt under nitrogen for 6 h and then concentrated under a stream of nitrogen. Purification by column chromatography (0-50% EtOAc/ petroleum ether) yielded the product as a colourless oil (473.1 mg, 1.26 mmol, 85% yield).

δ_H (400 MHz, $CDCl_3$) 5.17 (br s, 2H), 3.66 (s, 3H), 3.34 (s, 2H), 3.11 (q, 4H, J = 5.5 Hz), 2.68 (t, 4H, J = 5.9 Hz), 1.40 (s, 18H); **δ_C** (101 MHz, $CDCl_3$) 172.2, 156.2, 79.2, 55.0, 54.2, 51.7, 38.7, 28.5. **LRMS** (ESI) *m/z* found $[M+H]^+$ 376.3, $C_{17}H_{33}N_3O_6^+$ required 376.5.

These data are consistent with those previously reported.[2]

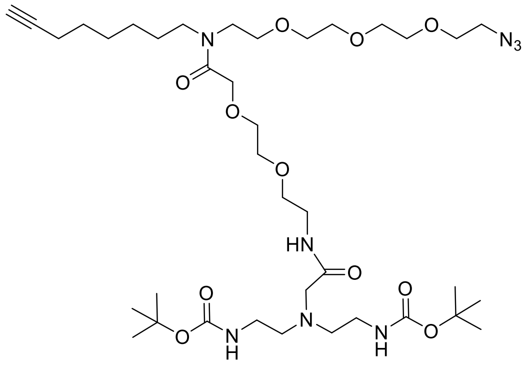

tert-butyl (20-azido-9-(oct-7-yn-1-yl)-8-oxo-3,6,12,15,18-pentaoxa-9-azaicosyl)carbamate (S13) *Step 1*: To a solution of methyl bis(2-((tert-butoxycarbonyl)amino)ethyl)glycinate **S10** (124 mg, 0.33 mmol) in MeOH (1.3 mL) and 1,4-dioxane (1.3 mL) was added aqueous NaOH (1 M, 0.67 mL). The reaction mixture was stirred at rt for 4 h and then concentrated *in vacuo*. The resulting residue was re-dissolved in MeOH, filtered, and concentrated again. The residue was then dissolved in DMF (1 mL). *Step 2*: To a solution of **S9** (188 mg, 0.330 mmol) in CH_2Cl_2 (1.13 mL) at 0 °C was added HCl (4M in dioxane, 2.74 mL). The reaction was stirred under nitrogen for 2 h and then concentrated *in vacuo* to yield the desired amine hydrochloride salt as a colourless oil. The amine

hydrochloride salt was re-dissolved in DMF (1 mL) followed by addition of the acid generated in step 1, triethylamine (0.14 mL, 0.99 mmol), HBTU (188 mg, 0.495 mmol). After stirring at rt for 36 h, the reaction mixture was diluted with EtOAc and washed with brine, 1 M HCl, brine, saturated $NaHCO_3$ and water. The organic phase was dried over Na_2SO_4 and concentrated *in vacuo*. Purification by column chromatography (0-10% MeOH/EtOAc) yielded the product as a colourless oil (153 mg, 0.188 mmol, 57%).

R_f 0.24 (SiO_2, 10% MeOH/EtOAc); **ν_{max}** (neat/cm^{-1}) 3317 (m, N-H), 2866 (m, C-H), 2104 (s, C≡C), 1657 (s, C=O), 1105 (s, C-O); **δ_H** (400 MHz, $CDCl_3$) 5.77 (s, 2H), 4.26-4.19 (m, 2H), 3.70-3.57 (m, 18H), 3.54-3.39 (m, 6H), 3.37-3.24 (m, 2H), 3.21-3.17 (m, 4H), 3.14-3.10 (m, 2H), 2.65-2.58 (m, 4H), 2.23-2.17 (m, 2H), 1.98-1.95 (m, 1H), 1.62-1.50 (m, 4H), 1.43-1.36 (m, 20H), 1.34-1.27 (m, 2H); **δ_C** (101 MHz, $CDCl_3$) 171.1, 169.2, 156.6, 85.5/84.3, 70.8-70.1, 79.1, 69.9, 69.2/69.1, 68.5/68.3, 58.7, 55.2, 50.7, 48.1/46.1, 46.6/45.8, 39.0, 38.5, 28.7, 28.5, 28.3, 27.4, 26.5/26.4, 18.3; Conformational isomers (rotamers) present; **HRMS** (ESI) m/z found $[M+H]^+$ 815.5248, $C_{38}H_{71}N_8O_{11}^+$ required 815.5242.

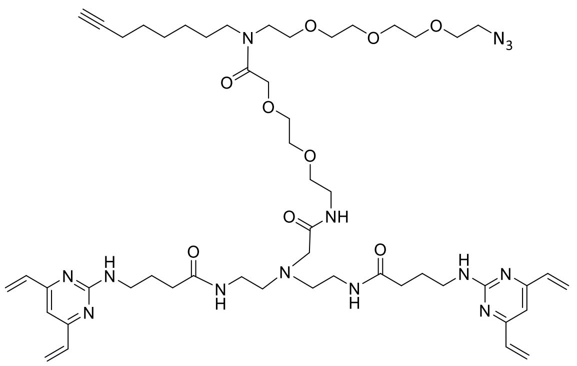

Bis-divinylpyrimidine Dual-functional alkyne-azide Linker (BisDVP 2) To a solution of **S13** (131.1 mg, 0.16 mmol) in CH_2Cl_2 (0.5 mL) at 0 °C was added HCl (4M in dioxane, 1.30 mL). The reaction was stirred under nitrogen for 2 h and then concentrated *in vacuo* to yield the desired amine hydrochloride salt as a colourless oil. The amine hydrochloride salt was re-dissolved in CH_2Cl_2 (2 mL) and cooled to 0 °C. A solution of 4-((4,6-divinylpyrimidin-2-yl)amino)butanoic acid **S3** (74.6 mg, 0.32 mmol), triethylamine (220 µL, 1.60 mmol), EDC•HCl (122.7 mg, 0.64 mmol) and HOBt monohydrate (98.0 mg, 0.640 mmol) in CH_2Cl_2 (5.6 mL) at 0 °C was added. The reaction was stirred under nitrogen for 18 h and then diluted with CH_2Cl_2 (30 mL), washed with brine (30 mL), dried over Na_2SO_4, and concentrated *in vacuo*. Purification by column chromatography (0-10% MeOH/CH_2Cl_2) yielded the product as a colourless oil (81.43 mg, 0.078 mmol, 49% yield).

R_f 0.30 (SiO_2, 5% MeOH/CH_2Cl_2); **ν_{max}** (neat/cm^{-1}) 3293 (m, N-H), 2861 (m, C-H), 2104 (s, C≡C), 1638 (s, C=O), 1101 (s, C-O); **δ_H** (500 MHz, $CDCl_3$) 7.48-7.44 (m, 2H) 6.53 (dd, 4H, *J* = 10.7, 17.2 Hz), 6.48 (s, 2H), 6.30 (d, 4H, *J* = 16.9 Hz), 6.04-6.01 (m, 2H), 5.51 (d, 4H, *J* = 10.6 Hz), 4.22-4.14 (m, 2H), 3.63-3.54 (m, 18H), 3.48-3.47 (m, 6H), 3.36-3.34 (m, 2H), 3.30-3.25 (m, 8H), 3.03-3.02 (m, 2H), 2.56-2.53 (m, 4H), 2.35-2.32 (m, 4H), 2.16-2.14 (m, 2H), 1.94-1.91 (m, 6H), 1.53-1.45 (m, 4H), 1.40-1.35 (m, 2H), 1.25-1.23 (m, 2H); **δ_C** (125.7 MHz, $CDCl_3$) 174.1, 171.1, 169.1/168.5, 163.6, 162.7, 136.0, 121.3, 105.2, 85.5/84.2, 70.7-69.9, 69.1, 68.8/68.6, 68.4/68.4, 58.0, 54.6, 50.6,

47.9/46.0, 46.5/45.8, 40.8, 39.2, 37.2, 33.5, 29.7, 28.6-28.2, 27.4, 26.4/26.3, 25.8, 18.3; Conformational isomers (rotamers) present; **HRMS** (ESI) m/z found $[M+H]^+$ 1045.6315, $C_{52}H_{81}N_{14}O_9^+$ required 1045.6311.

Scheme S3. Synthesis of BisDVP 3.

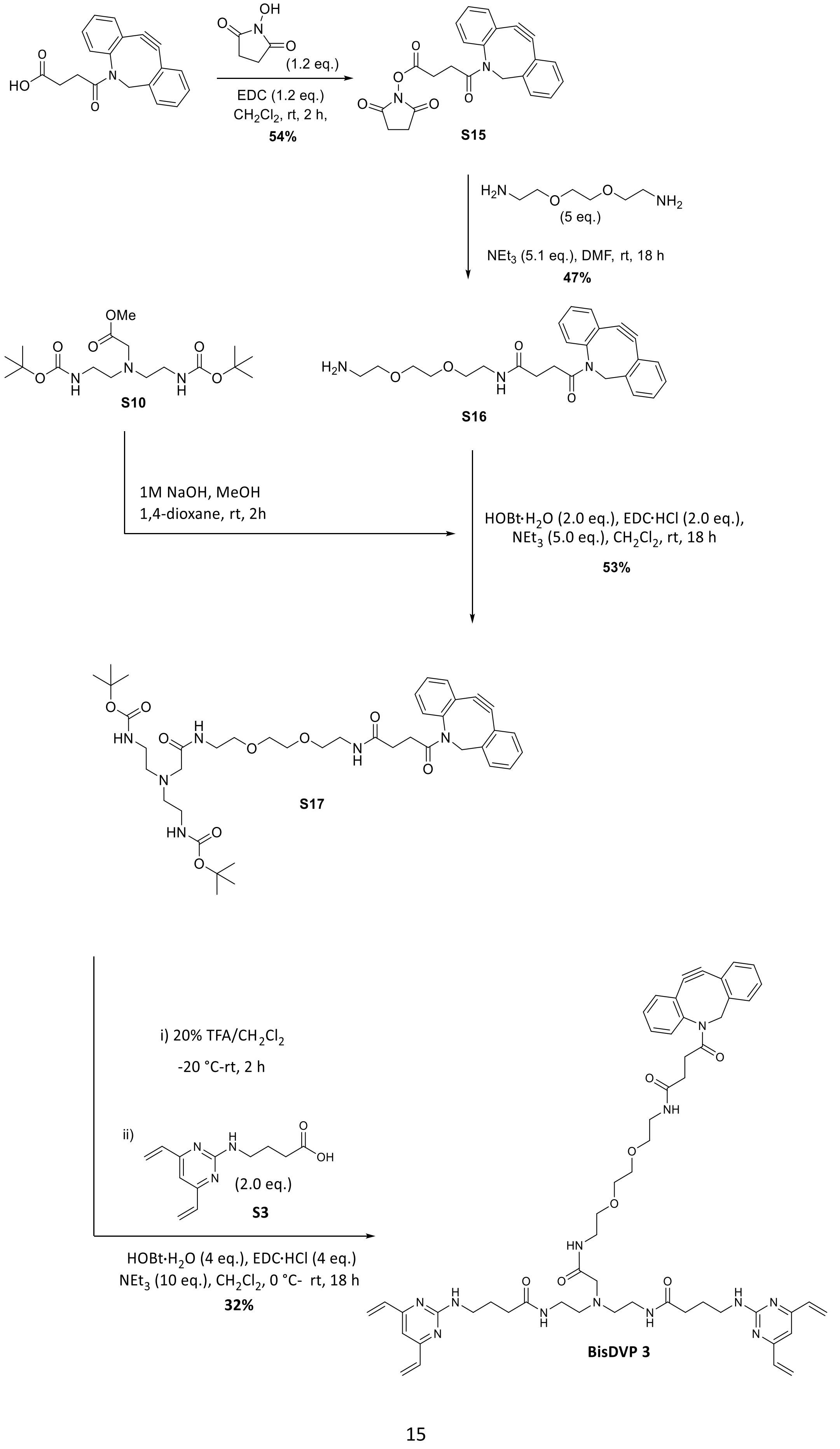

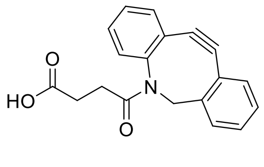

DBCO-COOH Synthesised by Dr Tim Schober using an OPRD protocol.[3]

$δ_H$ (400 MHz, $(CD_3)_2SO$) 11.97 (br s), 7.67-7.61 (2H, m, H9), 7.53 – 7.44 (3H, m), 7.41 – 7.33 (3H, m), 5.04 (1H, d, J = 14.0 Hz), 3.63 (1H, d, *J* = 14.0 Hz), 2.60 (dt, *J* = 16.6, 7.1), 2.34-2.16 (2H, m), 1.78 (dt, *J* = 16.7, 6.5, 1H); **$δ_C$** (101 MHz, $(CD_3)_2SO$) 174.0, 171.2, 151.9, 148.9, 132.8, 130.1, 129.4, 128.7, 128.5, 128.1, 127.3, 125.6, 123.0, 122.0, 114.7, 108.5, 55.4, 29.7, 29.4; **LRMS** (ESI) *m/z* found $[M+H]^+$ 306.1, $C_{19}H_{10}NO_3^+$ required 306.3.

These data are consistent with those previously reported.[4]

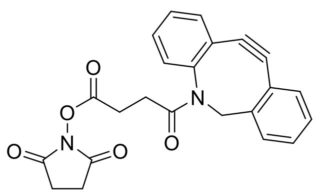

DBCO-NHS (S15) To a solution of **DBCO-COOH** (100 mg, 0.33 mmol) in CH_2Cl_2 (1.5 mL) was added NHS (45.2 mg, 0.393 mmol) and EDC (61 mg, 0.393 mmol). After stirring at room temperature for 2 h, the reaction mixture was filtered, concentrated *in vacuo* and purified by column chromatography on silica gel (2% MeOH/CH_2Cl_2) and concentrated to dryness to provide the product (71.5 mg, 0.178 mmol, 54%) as a white solid.

Rf = 0.6 (10% MeOH/CH_2Cl_2). **$δ_H$** (400 MHz, $CDCl_3$) 7.70 (d, 1H, *J* = 7.6 Hz), 7.44-7.37 (m, 5H), 7.33-7.31 (m, 1H), 7.28-7.27 (m, 1H), 5.19 (d, 1H, *J* = 14.0 Hz), 3.70 (d, 1H, *J* = 13.9 Hz), 3.00-2.95 (m, 1H), 2.85-2.79 (m, 5H), 2.67-2.63 (m, 1H), 2.11-2.07 (m, 1H); **$δ_C$** (101 MHz, $CDCl_3$) 170.3, 168.9 (2C), 168.3, 151.0, 147.8, 132.3, 129.1, 128.7, 128.4, 128.4, 127.8, 127.2, 125.6, 123.0, 122.7. 115.0, 107.6, 55.6, 29.2, 26.4, 25.5 (2C).

These data are consistent with those previously reported.[5]

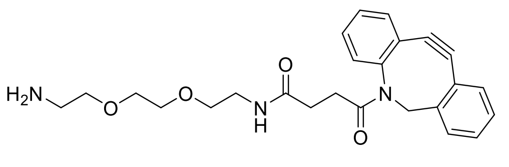

Amino-PEG2-DBCO (S16) To a solution of 2,2'-(ethane-1,2-diylbis(oxy))bis(ethan-1-amine) (0.13 mL, 0.889 mmol) in DMF (6 mL) was added DBCO-NHS **S15** (71.5 mg, 0.178 mmol) in DMF (2 mL) dropwise. To this solution was added triethylamine (0.13 mL, 0.907 mmol) in DMF (2 mL) dropwise, and the reaction was stirred at rt for 18 h. The reaction mixture was purified by HPLC (10-60% MeCN/H_2O over 20 min) and lyophilisation yielded the product as a white solid (36.5 mg, 0.084 mmol, 47% yield).

N_{max} (neat/cm^{-1}) 3280 (m, N-H), 2874 (m, C-H), 2115 (m, C≡C), 1638 (s, C=O), 1562 (s, C=C); 1094 (s, C-O), 1036 (m, C-N); **δ_H** (400 MHz, $CDCl_3$) 7.77-7.75 (m, 1H), 7.70-7.68 (m, 1H), 7.64-7.62 (m, 1H), 7.53-7.48 (m, 3H), 7.41-7.29 (m, 3H), 5.04 (d, 1H, *J* = 14.0 Hz), 3.63 (d, 1H, *J* = 13.9 Hz), 3.60-3.57 (m, 4H), 3.52-3.43 (m, 4H), 3.13-3.08 (m, 2H), 2.99-2.94 (m, 2H), 2.62-2.55 (m, 1H), 2.28-2.20 (m, 1H), 2.05-1.98 (m, 1H), 1.81-1.74 (m, 1H); **δ_C** (101 MHz, $(CD_3)_2SO$) 171.7, 171.5, 152.1, 148.9, 132.9, 130.1, 129.4, 128.6, 128.5, 128.1, 127.3, 125.6, 123.0, 121.9, 114.7, 108.6, 70.1, 69.8, 69.4, 67.1, 55.4, 39.1, 38.9, 30.8, 30.1; **HRMS** (ESI) m/z found $[M+H]^+$ 436.2233, $C_{25}H_{30}N_3O_4^+$ required 436.2231.

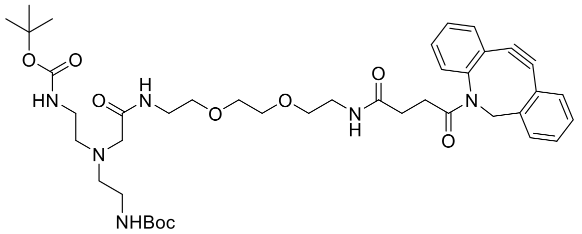

Boc-Amino-PEG2-DBCO (S17)

Step 1: To a solution of methyl bis(2-((tert-butoxycarbonyl)amino)ethyl)glycinate **S10** (38.3 mg, 0.102 mmol) in MeOH (0.41 mL) and 1,4-dioxane (0.41 mL) was added aqueous NaOH (1 M, 0.21 mL). The reaction mixture was stirred at rt for 2 h and then concentrated *in vacuo*. The resulting residue was re-dissolved in MeOH, filtered, and concentrated again. The residue was then dissolved in CH_2Cl_2 (1 mL). *Step 2:* To a solution of Amino-PEG2-DBCO **S16** in CH_2Cl_2 (1 mL) was added at 0 °C the acid generated in step 1, triethylamine (72 µL, 0.51 mmol), EDC•HCl (39.1 mg, 0.204 mmol) and HOBt monohydrate (27.6 mg, 0.204 mmol) in CH_2Cl_2 (1 mL). The reaction was stirred under nitrogen for 5 h and concentrated *in vacuo*. The reaction mixture was purified *via* flash column chromatography (5% MeOH/ CH_2Cl_2) to give the product as a yellow oil (42 mg, 0.054 mmol, 53% yield).

R_f 0.68 (SiO_2, 10% MeOH/CH_2Cl_2); **ν_{max}** (neat/cm^{-1}) 3321 (m, N-H), 2872 (m, C-H), 2110 (m, C≡C), 1657 (s, C=O), 1525 (s, C=C), 1167 (s, C-O); **δ_H** (400 MHz, $CDCl_3$) 7.64 (d, 1H, *J* = 7.5 Hz), 7.51-7.49 (m, 2H), 7.38-7.21 (m, 6H), 6.33 (br s, 1H), 5.62 (br s, 2H), 5.12 (d, 1H, *J* = 13.9 Hz), 3.64 (d, 1H, *J* = 13.8 Hz), 3.59-3.54 (m, 6H), 3.44-3.40 (m, 4H), 3.31-3.27 (m, 2H), 3.15-3.11 (m, 4H), 3.07 (s, 2H), 2.82-2.74 (m, 1H), 2.46-2.39 (m, 1H), 2.55 (t, 4H, *J* = 5.4 Hz), 2.16 (dt, 1H, *J* = 15.1, 6.3 Hz), 1.92 (dt, 1H, *J* = 16.5, 6.3 Hz), 1.42 (s, 18H); **δ_C** (101 MHz, $(CD_3)_2SO$) 172.3, 172.2, 171.3, 156.5, 151.4, 148.1, 132.2, 129.4, 128.6, 128.1, 128.1, 127.7, 127.0, 125.5, 123.2, 122.5, 114.7, 107.9, 79.3, 70.2, 69.9, 69.8, 69.5, 58.8, 55.5, 55.2, 39.0, 38.9, 38.6, 31.1, 30.1, 28.5; **HRMS** (ESI) m/z found $[M+H]^+$ 779.4369, $C_{41}H_{59}N_6O_9^+$ required 779.4338.

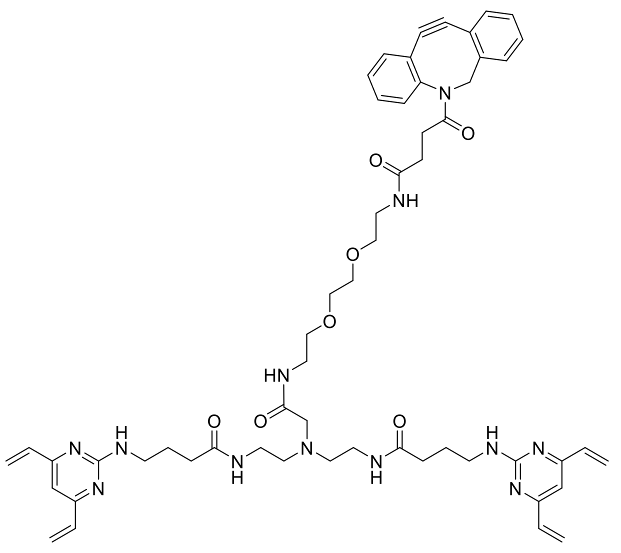

Bis-divinylpyrimidine- DBCO (BisDVP 3) To a solution of **S17** (167 mg, 0.214 mmol) in CH_2Cl_2 (0.5 mL) at -20 °C was added 20% TFA/ CH_2Cl_2 (18.3 mL) dropwise. The reaction was stirred under nitrogen for 2.5 h slowly allowing to reach rt. Upon completion the reaction was diluted with toluene. Residual TFA was removed by azeotroping with toluene and the solution was concentrated *in vacuo* to yield the desired TFA salt. The amine TFA salt was re-dissolved in CH_2Cl_2 (4 mL) and cooled to 0 °C. A solution of 4-((4,6-divinylpyrimidin-2-yl)amino)butanoic acid S**3** (99.9 mg, 0.428 mmol), triethylamine (300 µL, 2.14 mmol), EDC•HCl (164.1 mg, 0.856 mmol) and HOBt monohydrate (131 mg, 0.856 mmol) in CH_2Cl_2 (6 mL) at 0 °C was added. The reaction was stirred under nitrogen for 18 h and then diluted with CH_2Cl_2 (30 mL), washed with brine (30 mL), dried over Na_2SO_4, and concentrated *in vacuo*. Purification by column chromatography (0-10% MeOH/CH_2Cl_2) yielded the product as a yellow oil (69.1 mg, 0.0685 mmol, 32% yield).

$\mathbf{R_f}$ 0.40 (SiO_2, 10% MeOH/CH_2Cl_2); $\mathbf{\nu_{max}}$ (neat/cm^{-1}) 3302 (m, N-H), 2872 (m, C-H), 2115 (m, C≡C), 1638 (s, C=O), 1541 (s, C=C); $\mathbf{\delta_H}$ (700 MHz, $CDCl_3$) 7.66 (1H, d, *J* = 7.5 Hz), 7.52-7.50 (1H, m), 7.42-7.41 (1H, m), 7.40-7.39 (2H, m), 7.37-7.36 (1H, s), 7.35-7.33 (1H, m), 7.30 (1H, t, *J* = 7.5 Hz), 7.23 (1H, d, J = 7.5 Hz), 7.17 (2H, t, *J* = 4.9 Hz), 6.57 (dd, 4H, *J* = 10.7, 17.4 Hz), 6.53 (2H, s), 6.34 (4H, d, *J* = 17.1 Hz), 5.97 (2H, t, *J* = 5.8 Hz), 5.55 (4H, d, *J* = 10.7 Hz), 5.15 (1H, d, *J* = 14.0 Hz), 3.69 (1H, d, *J* = 14.0 Hz), 3.54-3.51 (m, 8H), 3.48-3.47 (m, 2H), 3.44-3.37 (m, 2H), 3.34-3.27 (m, 8H), 3.05 (2H, s), 2.83-2.78 (m, 1H), 2.56 (4H, t, *J* = 5.4 Hz), 2.44-2.40 (m, 1H), 2.33 (4H, t, *J* = 7.5 Hz), 2.18 (1H, dt, *J* = 15.3, 6.1 Hz), 1.97-1.93 (m, 5H); $\mathbf{\delta_C}$ (176.1 MHz, $CDCl_3$) 174.0, 172.4, 171.2, 163.7, 162.7, 151.3, 148.1, 135.9, 132.2, 129.3, 128.7, 128.3, 128.1, 127.8, 127.1, 125.5, 123.2, 122.5, 121.4, 114.6, 107.9, 105.2, 70.0, 70.0, 69.9, 69.7, 58.2, 55.6, 54.6, 40.7, 39.1, 38.9, 37.3, 33.6, 31.1, 30.2, 25.8 (Note, peak at 53.4 = CH_2Cl_2, and peak at 50.8 = MeOH); **HPLC** (10-60%, Abs: 254 mAU, MeCN/H_2O over 20 min) retention time 11.438 min; **HPLC** (40-60% MeCN/H_2O over 20 min) retention time 2.376 min; **HRMS** (ESI) m/z found $[M+H]^+$ 1009.5436, $C_{55}H_{69}N_{12}O_7^+$ required 1009.5412.

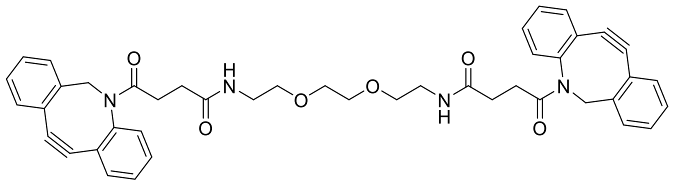

DBCO-PEG2-DBCO (S18) A solution of DBCO-acid (350 mg, 1.15 mmol), triethylamine (0.97 mL, 6.88 mmol), EDC•HCl (660 mg, 3.44 mmol) and HOBt monohydrate (465 mg, 3.44 mmol) was stirred in CH_2Cl_2 (15 mL) at 0 °C. This solution was then added dropwise to a solution of diamine (80 uL, 0.573 mmol) in CH_2Cl_2 (5 mL) at 0 °C for 12 h. The reaction was concentrated *in vacuo*. Purification by reverse phase flash column chromatography (10-60% solvent B in solvent A. Solvent A: H_2O. Solvent B: MeCN) and lyophilisation yielded the product as a light brown solid (261 mg, 0.361 mmol, 63% yield).

ν_{max} (neat/cm^{-1}) 3316 (m, N-H), 2870 (m, C-H), 2126 (m, C≡C), 1642 (s, C=O), 1545 (s, C=C); 1094 (s, C-O); **δ_H** (400 MHz, $CDCl_3$) 7.67-7.64 (m, 2H), 7.54-7.50 (m, 2H), 7.44-7.38 (m, 6H), 7.34-7.21 (m, 6H), 6.37-6.28 (m, 2H), 5.14 (d, 2H, *J* = 13.9 Hz), 3.66 (dd, 2H, *J* = 13.8, 2.7 Hz), 3.56-3.55 (m, 4H), 3.52-3.42 (m, 4H), 3.37-3.27 (m, 4H), 2.84-2.76 (m, 2H), 2.46-2.34 (m, 2H), 2.21-2.03 (m, 2H), 1.99-1.86 (m, 2H); **δ_C** (176.1 MHz, $CDCl_3$) 172.4, 172.3, 151.4, 148.1, 132.2, 129.4, 128.6, 128.1, 128.1, 127.7, 127.0, 125.5, 123.2, 122.5, 114.7, 107.9, 70.2, 69.8, 55.5, 39.2, 31.1, 30.2; **HRMS** (ESI) m/z found $[M+H]^+$ 723.3185, $C_{44}H_{43}N_4O_6^+$ required 723.3177.

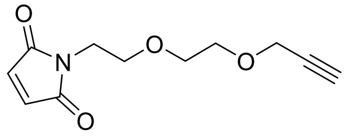

1-(2-(2-(prop-2-yn-1-yloxy)ethoxy)ethyl)-1H-pyrrole-2,5-dione (S19)

A solution of amine (125 mg, 0.87 mmol) and maleic anhydride (85.6 mg, 0.87 mmol) was heated under reflux in AcOH (4.6 mL) for 18 h. The resulting solution was concentrated *in vacuo* and purified *via* flash column chromatography (50% EtOAc/PE) to give the desired product as a light-yellow oil (94 mg, 0.418 mmol, 48% yield).

R_f 0.47 (SiO_2, 50% EtOAc/PE); **ν_{max}** (neat/cm^{-1}) 2865 (m, C-H), 2120 (m, C≡C), 1694 (s, C=O), 1107 (s, C-O), 1031 (m, C-N); **δ_H** (400 MHz, $CDCl_3$) 6.70 (s, 2H), 4.16 (d, 2H, J = 2.3 Hz), 3.75 – 3.72 (m, 2H), 3.66 - 3.64 (m, 6H), 2.42 (t, 1H, J = 2.3 Hz); **δ_C** (101 MHz, $CDCl_3$) 170.8, 134.3, 79.8, 74.6, 70.0, 69.2, 68.0, 58.6, 37.2; **HRMS** (ESI) m/z found $[M+H]^+$ 224.0915, $C_{11}H_{14}NO_4^+$ required 224.0923.

Peptide Synthesis

Automated SPPS was performed using a CEM Liberty Blue Automated Microwave Peptide Synthesiser using MHBA Rink Amide resin (0.308 mmol/g, 100-200 mesh). All peptide couplings were performed with Fmoc-protected amino acids (5 equiv.), Oxyma pure (10 equiv.) and DIC (5 equiv.) in DMF. All amino acids were coupled with 25 W power at 75 °C over 15 min. Fmoc deprotection was performed with a solution of 20% piperidine in DMF, with 45 W power at 75 °C over 3 min.

N-terminal acetyl capping was performed on the N-terminus of the peptide after the final deprotection. The capping was achieved by adding Ac_2O (5.0 equiv.) and DIPEA (10.0 equiv.) in CH_2Cl_2 to the resin and was shaken at room temperature overnight. Peptide was cleaved from the resin by treatment with a TFA cleavage cocktail consisting of TFA (91%), H_2O (3%), EDT (3%), and thioanisole (3%) and the mixture was agitated at room temperature for 3 h. The resin was filtered, and the cleavage mixture removed under a stream of nitrogen until only a few millilitres remained. The peptide was then precipitated by addition of the cleavage mixture to cold diethyl ether. The precipitated crude peptide was collected, dissolved, and purified by preparative HPLC using a gradient of 20-60% B. The purified peptide was lyophilised, the mass analysed by LCMS, and the purity determined by analytical HPLC.

Afamelanotide-N_3

Ac-X-Ser-Tyr-Ser-Nle-Glu-His-D-Phe-Arg-Trp-Gly-Lys-Pro-Val-NH_2 (X = Azido-lysine).

White solid (213 mg, 43% yield, 95% purity). **HPLC** (5-95% MeCN/H_2O over 20 min) retention time 7.459 min; **HPLC** (20-60% MeCN/H_2O over 20 min) retention time 10.047 min; **LRMS** (ESI) *m/z* found $[M+H]^+$ 1982.4, required 1982.3.

Exenatide-PEG24-N_3 (≥95% purity) was purchased from GL Biochem, China.

HGEGTFTSDLSKQMEEEAVRLFIEWLKNGGPSSGAPPPS-PEG24-N_3

Exenatide used for PK *in vivo* study was purchased from Bachem.

HGEGTFTSDLSKQMEEEAVRLFIEWLKNGGPSSGAPPPS

"Dulaglutide" used for PK *in vivo* study is a recombinant fusion protein, produced by a recombinant expression in CHO cells, which has 100% sequence homology to Dulaglutide.

HGEGTFTSDVSSYLEEQAAKEFIAWLVKGGGGGGGSGGGGSGGGGSAESKYGPPCPPCPAPEAAGGPSVFLFPPKPKDTLMIS
RTPEVTCVVVDVSQEDPEVQFNWYVDGVEVHNAKTKPREEQFNSTYRVVSVLTVLHQDWLNGKEYKCKVSNKGLPSSIEKTIS

KAKGQPREPQVYTLPPSQEEMTKNQVSLTCLVKGFYPSDIAVEWESNGQPENNYKTTPPVLDSDGSFFLYSRLTVDKSRWQEG
NVFSCSVMHEALHNHYTQKSLSLSLG

Afamelanotide-N_3 and BisDBCO (S18) SPAAC to form Afamelanotide-DBCO. To Afamelanotide-N_3 (8.5 mg), in PBS (20 mL) and NaPi (0.1 M) (4 mL) with DMSO (2 mL) (8% DMSO), was added BisDBCO linker **S18** (5 eq., 17 mg). The reaction was stirred at room temperature for 5 h and monitored by HPLC. The reaction mixture was then purified by preparative HPLC using a gradient of 10-60% B (Solvent A: 0.05% (v/v) TFA in H_2O; Solvent B: 0.05% (v/v) TFA in CH_3CN). The purified peptide conjugate was lyophilised (57% yield, 90% purity). **HPLC** (5-95% MeCN/H_2O over 20 min) retention time 7.544 min.

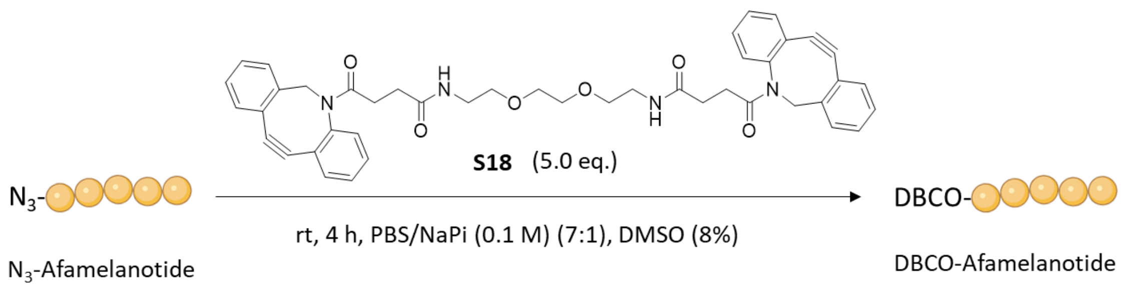

Scheme S4: Reaction of Afamelanotide-azide with BisDBCO **S18** to form Afamelanotide-DBCO.

Bioconjugation

Peptide Conjugation General Information

SDS-PAGE

Non-reducing Tris-Glycine SDS-PAGE with 12% acrylamide with 4% stacking gel was performed as standard. Broad range molecular weight marker (10-200 kDa, New England BioLabs) was run in all gels. Samples (10 µL at approximately 10 µM) were prepared with reducing or non-reducing loading dye (10 µL, reducing dye contains β-mercaptoethanol) and heated to 90 °C for 5 min prior to loading. Gels were run at constant voltage (200 V) for 65 min in 1 × Laemmli running buffer (LRB). All gels were stained with Coomassie dye and imaged on a Syngene gel imaging system. Gels containing fluorescently labelled samples were imaged for in-gel fluorescence prior to Coomassie staining.

UV-vis Spectroscopy

UV-vis spectroscopy was used to determine protein concentrations and fluorophore to antibody ratios (FAR) using a NanoDrop One spectrophotometer. Sample buffer was used as a blank for baseline correction. The concentration of antibody species in solution was determined using UV-vis
spectroscopy. The Beer-Lambert Law states that: $A = \varepsilon c l$ where A = absorbance, ε = extinction coefficient, c = concentration and l = path length. In most cases, the path length is set to 1 cm to simplify the equation. The following extinction coefficients were used: ε280 = 215380 M^{-1} cm^{-1} for trastuzumab IgG; ε280 = 71570 M^{-1} cm^{-1} for Trastuzumab digest/recombinant Fc protein; ε280 = 5500 M^{-1} cm^{-1} for Exenatide; (extinction coefficients for IgG, Fc and Exenatide were determined theoretically from amino acid sequence data); ε280 = 2540 M^{-1} cm^{-1} for divinylpyrimidine residue;[159] ε495 = 71000 M^{-1} cm^{-1} for Alexa Fluor® 488; Correction factors for the absorption at 280 nm of Alexa Fluor® 488 was 0.11, obtained from the manufacturer. Extinction coefficients for synthesised Fc-peptide conjugates were determined from the sum of the extinction coefficients of the protein and peptide parts e.g. Fc-Exenatide (71570 + 5500 = 77070 M^{-1} cm^{-1}).

Protein LCMS

Protein LC–MS was performed on a Xevo G2-S TOF mass spectrometer coupled to an Acquity UPLC system using an Acquity UPLC BEH300 C4 column (1.7 µm, 2.1 × 50 mm). H2O with 0.1% formic acid (solvent A) and 95% MeCN and 5% water with 0.1% formic acid (solvent B), were used as the mobile phase at a flow rate of 0.2 mL/min. The gradient was programmed as follows: 95% A for 0.93 min, then a gradient to 100% B over 4.28 min, then 100% B for 1.04 minutes, then a gradient to 95% A over 1.04 min. The electrospray source was operated with a capillary voltage of 2.0 kV and a cone voltage of 190 V. Nitrogen was used as the desolvation gas at a total flow of 850 L/h. Total mass spectra were reconstructed from the ion series using the MaxEnt 1 algorithm preinstalled on MassLynx 4.2 software according to the manufacturer's instructions. Trastuzumab/ Fc protein samples were deglycosylated with PNGase F (New England Biolabs) prior to LCMS analysis, unless otherwise specified. Only

the region of the total ion chromatograph (TIC) between 3.00-3.90 min was analysed. Peaks outside of this range did not contain proteinogenic signals and were excluded.

Size-exclusion Chromatography (SEC)

Analytical size-exclusion chromatography (SEC) was performed on an Agilent 1260 Infinity using a Tosoh Bioscience TSKgel G3000SWXL column (7.8 mm × ID 30 cm L). Proteins were eluted with NaPi pH 7 (50 mM NaPi, 100 mM NaCl, 0.02% NaN3) at a flow rate of 0.6 mL/min.

Preparative SEC was performed on an AKTA pure chromatography system using a Superdex^{TM} 200 Increase 10/300 GL column. Samples were injected at a concentration of approximately 1 mg/mL and eluted with TBS pH 8 (25 mM Tris HCl, 200 mM NaCl, 0.5 mM EDTA) at a flow rate of 0.5 mL/min.

Protein A Purification

Cytiva Protein A HP SpinTrap™ columns were used following the antibody purification protocol. Firstly, the Fc protein sample was buffer exchanged into binding buffer (20 mM sodium phosphate, pH 7.0). The Protein A HP SpinTrap™ column (Cytiva) was equilibrated with binding buffer. The sample was then applied to the column and incubated at room temperature with end-over-end mixing for 4 minutes. Unbound species were eluted from the column and the column was washed with binding buffer two times. The bound Fc fraction was then eluted with two washes of elution buffer (0.1 M glycine-HCl, pH 2.7) which was neutralised with neutralising buffer 7% (V/V) (1 M Tris-HCl, pH 9.0). The eluted Fc solutions were immediately buffer exchanged into PBS buffer.

UV-Vis FAR Calculation

Sample buffer was used as a baseline for analysis. FAR was calculated using the following formula;

$$FAR = \frac{Abs_{495}/\varepsilon_{495}}{(Abs_{280\_corrected} - 0.11 \times Abs_{495})/\varepsilon_{280}}$$

$$Abs_{280\_corrected} = Abs_{280} - (0.61 \times Abs_{298}) + (0.1 \times Abs_{280})$$

where;

Abs_{495} = 0.49

Abs_{280} = 0.67

$Abs_{280\_corrected}$ = 0.60

ε_{280} = 71,570 M^{-1} cm^{-1} for trastuzumab Fc

ε_{495} = 71,000 M^{-1} cm^{-1} for AlexaFluor488

A correction factor of 0.11 for AlexaFluor488 absorption at 280 nm and a corrected absorbance for trastuzumab at 280 nm was used to account for DVP absorbance at 280 nm.

Protein A purification of Fc-Exenatide to remove Exenatide

LC-MS analysis confirmed that protein A purification removed the Exenatide impurity in the wash step and afforded clean Fc-Exenatide conjugate.

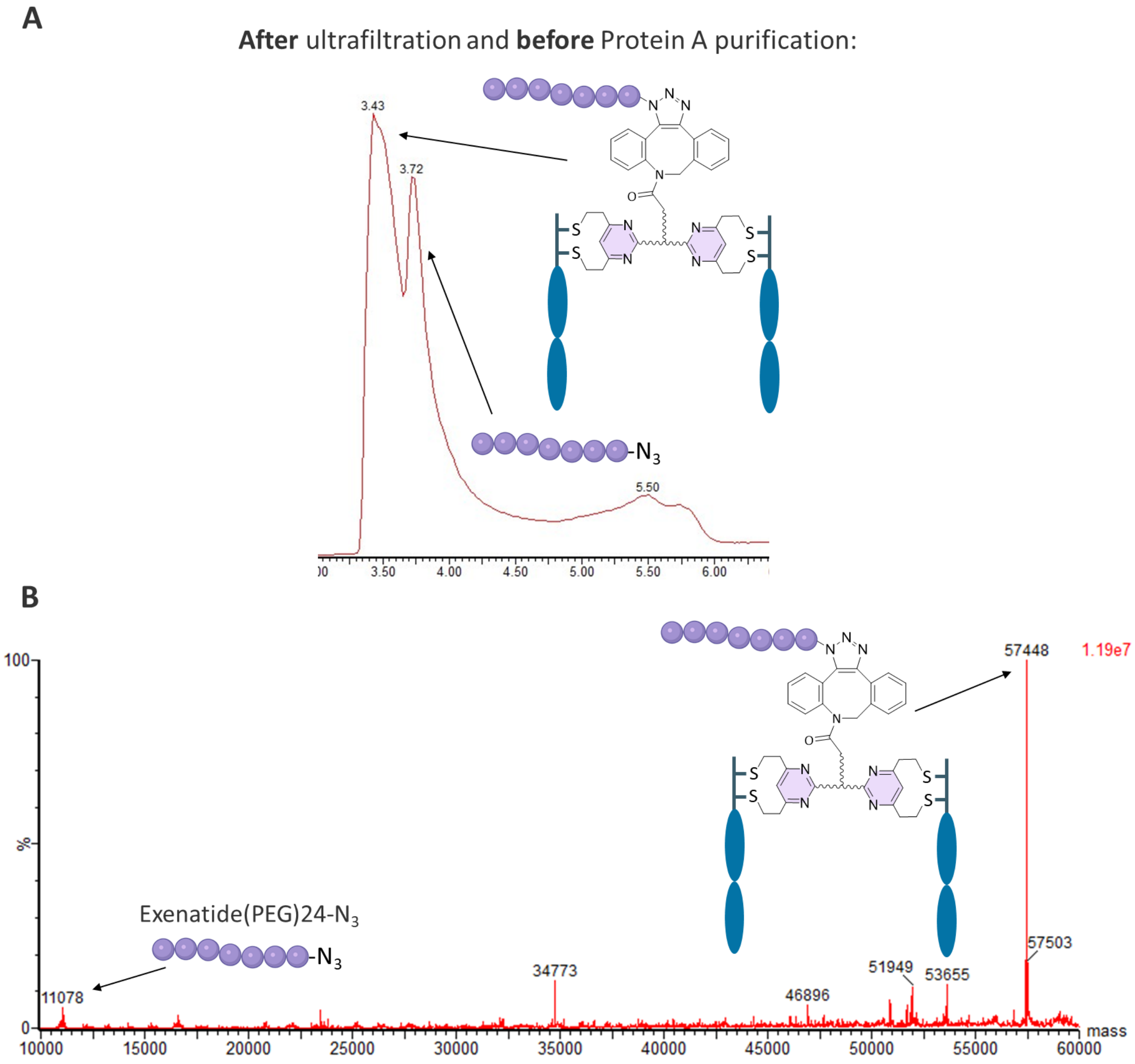

Figure S1: Analysis of Fc-Exenatide protein A purification LC-MS. A) LC-MS Chromatogram of Fc-Exenatide *before* protein A purification. B) Analysis of Fc-Exenatide *before* protein A purification by LC-MS, deconvoluted MS; expected 57440 Da and observed 57448 Da, and Exenatide(PEG)24-azide expected 5540 Da and observed 11078 Da (2M+H).

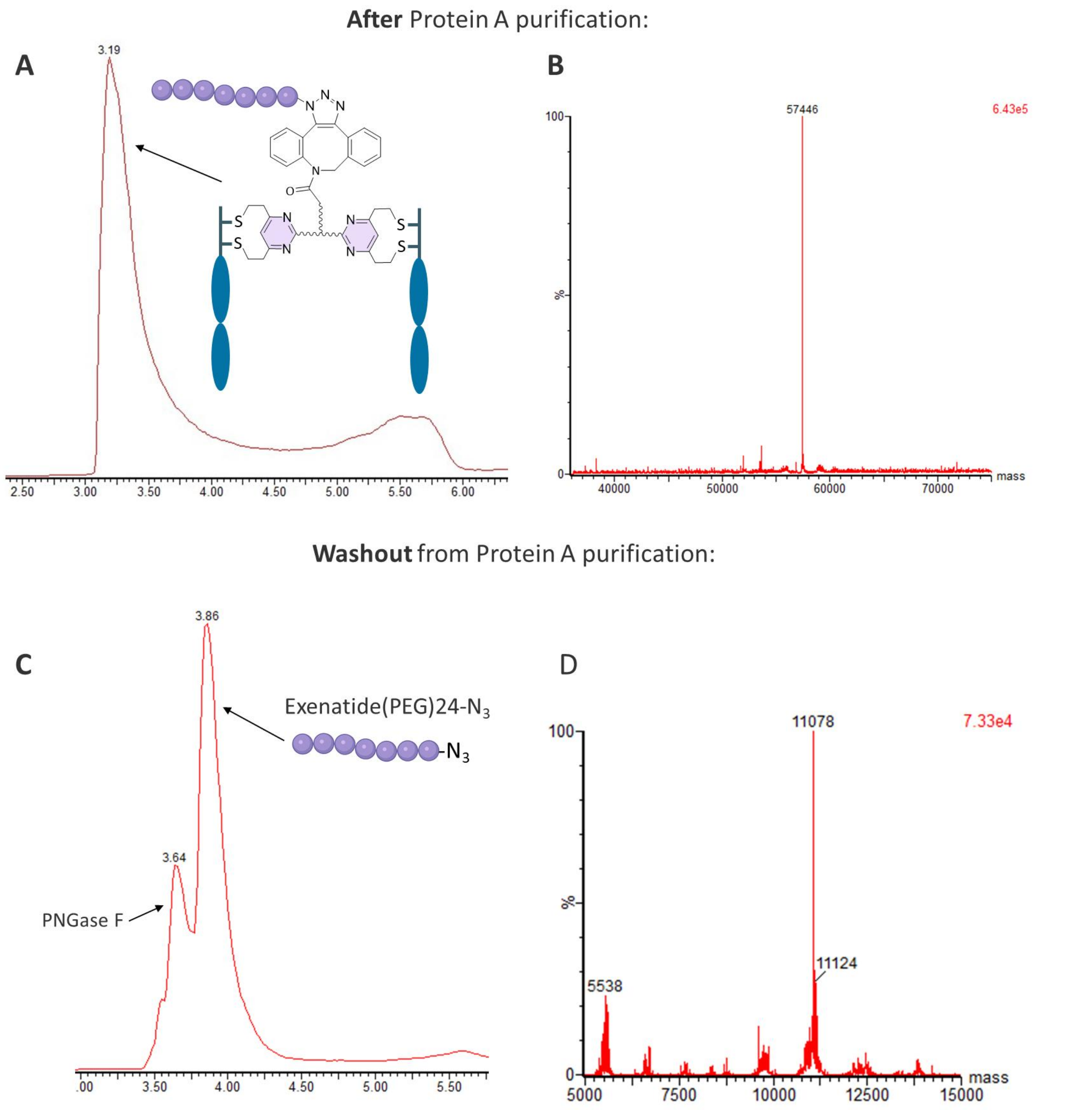

Figure S2: Analysis of Fc-Exenatide protein A purification LC-MS. A) LC-MS Chromatogram of Fc-Exenatide *after* protein A purification. B) Analysis of Fc-Exenatide *after* protein A purification by LC-MS, deconvoluted MS; expected 57440 Da and observed 57446 Da. C) LC-MS Chromatogram of washout from protein A purification. D) Analysis of washout from protein A purification by LC-MS, deconvoluted MS; expected 5540 Da and observed 5538 Da and 11079 Da (2M+H).

Fc Production *via* Trastuzumab Digestion

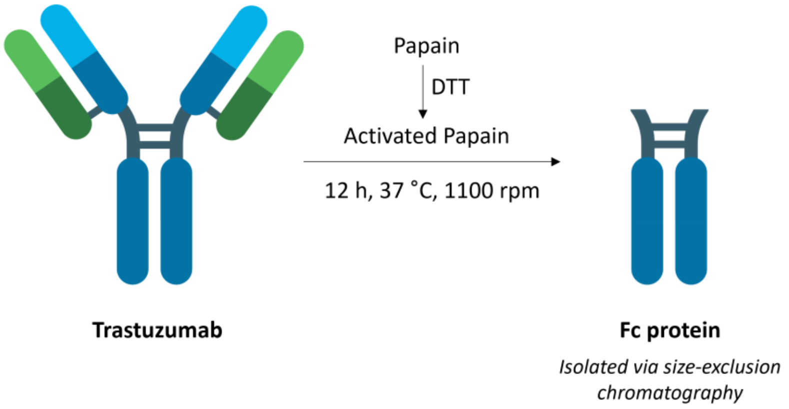

Trastuzumab Fc was prepared using the Pierce™ Fab Preparation Kit (ThermoFisher). Immobilised papain (0.5 mL, 0.25 mg/mL) was activated with 10 mM DTT (in Digestion buffer: 50 mM phosphate, 1 mM EDTA, 150 mM NaCl, pH 6.8) whilst shaking (1100 rpm) for 1 h at 37 °C. The resin was washed with Digestion buffer (without DTT) four times. Trastuzumab (0.5 mL, 1-4 mg) in Digestion buffer was added and the mixture was incubated for 12 h at 37 °C whilst shaking (1100 rpm). The digest was isolated from the papain and the papain was washed with binding buffer (20 mM sodium phosphate, pH 7.0) four times. The collection and washes were combined and concentrated by diafiltration, and buffer exchanged into SEC TBS buffer (25 mM Tris, *200 mM NaCl*, 0.5 mM EDTA, pH 8). The trastuzumab Fab and Fc were separated using size-exclusion chromatography. The isolated Fc protein was buffer exchanged into TBS buffer (25 mM Tris, *25 mM NaCl*, 0.5 mM EDTA, pH 8) and stored at 4 °C until use.

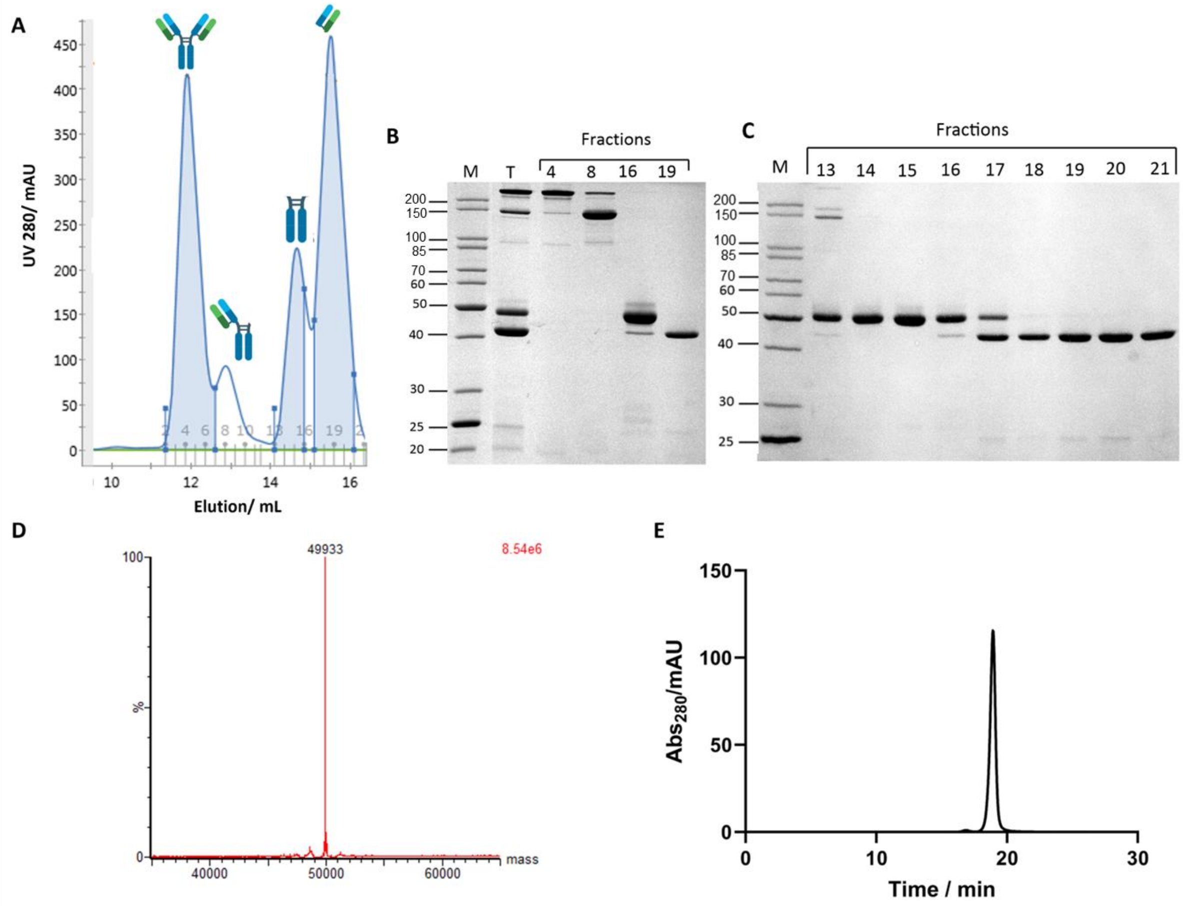

F

TCPPCPAPELLGGPSVFLFPPKPKDTLMISRTPEVTCVVVDVSHEDPEVKFNWYVDGVEVHNAKT
KPREEQYNSTYRVVSVLTVLHQDWLNGKEYKCKVSNKALPAPIEKTISKAKGQPREPQVYTLPPS
REEMTKNQVSLTCLVKGFYPSDIAVEWESNGQPENNYKTTPPVLDSDGSFFLYSKLTVDKSRWQ
QGNVFSCSVMHEALHNHYTQKSLSLSPG

Figure S3: A) SEC chromatogram for the digestion of trastuzumab. B) SDS-PAGE analysis (12% separating gel, non-reducing conditions) of the SEC fractions for the purification of trastuzumab digestion. Lane M) molecular weight marker, lane T) shows the trastuzumab digest mixture, lanes 4-19) SEC fractions. C) SDS-PAGE analysis (12% separating gel, non-reducing conditions) of the SEC fractions of a trastuzumab digest. Lane M) molecular weight marker, lanes 13-21) SEC fractions. Proteins were visualised by Coomassie blue stain. D) Deconvoluted mass spectrum of deglycosylated Fc protein, expected mass: 49923 Da; found: 49933 Da. E) Analytical SEC chromatogram of Fc protein. F) Full amino acid sequence of Fc protein heavy chain.

Fc Production *via* Recombinant Expression

A 500 mL culture of suspension CHO G22 cells[6] were transfected, using a polyethylenimine (PEI MAX, Polysciences) -based method, with a plasmid DNA containing a gene encoding Fc portion of human IgG1 with N-terminal signal leader (facilitating secretion to the medium) under the control of a mammalian promoter. The cells were then cultured at 37C and on day 1 fed using AstraZeneca proprietary expression-medium feed and then cultured at 34C and fed again on day 3. After 6 days, the cells were harvested by centrifugation and the culture supernatant containing recombinant proteins was collected and filtered. The Fc-containing supernatant was purified using protein A-based column (MabSelect SuRe, Cytiva) using an AKTAxpress system The purified Fc protein was eluted from the resin using low pH (pH 2.7) 0.1 M glycine and peak fractions combined, neutralised with 10% vol 1 M Tris (pH 9) then buffer exchanged to PBS pH 7.4 using a PD-10 column and protein concentration was measured at A280 using NanoDrop. The final Fc protein was then analysed by SDS-PAGE and LC-MS. The Fc protein was aliquoted in 100 µL samples at a concentration of ~2 mg/mL then snap frozen on liquid nitrogen and stored at -80 °C.

DKTHTCPPCPAPELLGGPSVFLFPPKPKDTLMISRTPEVTCVVVDVSHEDPEVKFNWYVDGVEVH
NAKTKPREEQYNSTYRVVSVLTVLHQDWLNGKEYKCKVSNKALPAPIEKTISKAKGQPREPQVYT
LPPSREEMTKNQVSLTCLVKGFYPSDIAVEWESNGQPENNYKTTPPVLDSDGSFFLYSKLTVDKS
RWQQGNVFSCSVMHEALHNHYTQKSLSLSPGK

Figure S4: Full amino acid sequence of Fc protein heavy chain derived from human IgG1 (identical to the Fc sequence contained in Trastuzumab), C-terminal lysine (K) is cleaved during expression and not present in final protein.

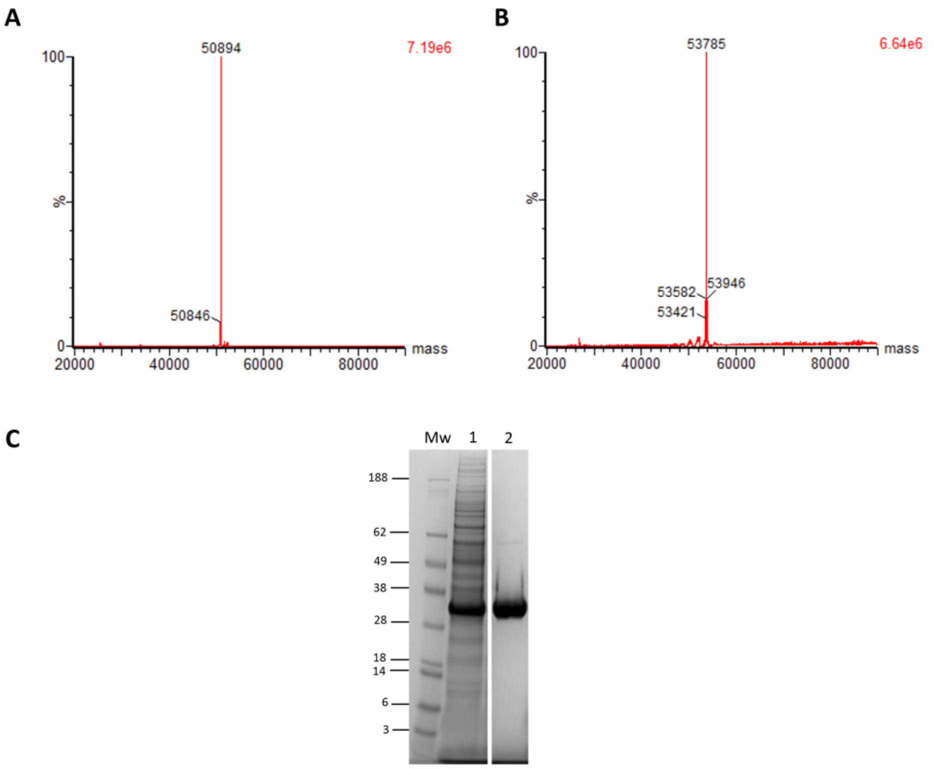

Figure S5: A) Deconvoluted mass spectrum of deglycosylated recombinant Fc protein, expected mass: 50891 Da; found: 50894 Da; B) Deconvoluted mass spectrum of recombinant Fc protein with glycosylation, major species found: 53785 Da. C) SDS-PAGE analysis under reducing conditions. Lanes: Mw) pre-stained molecular weight standard SeeBlueTM (Invitrogen), 1) culture supernatant containing Fc protein before purification, and 2) Fc protein after MAbSelect SuRe purification. Proteins were visualised by Coomassie blue stain.

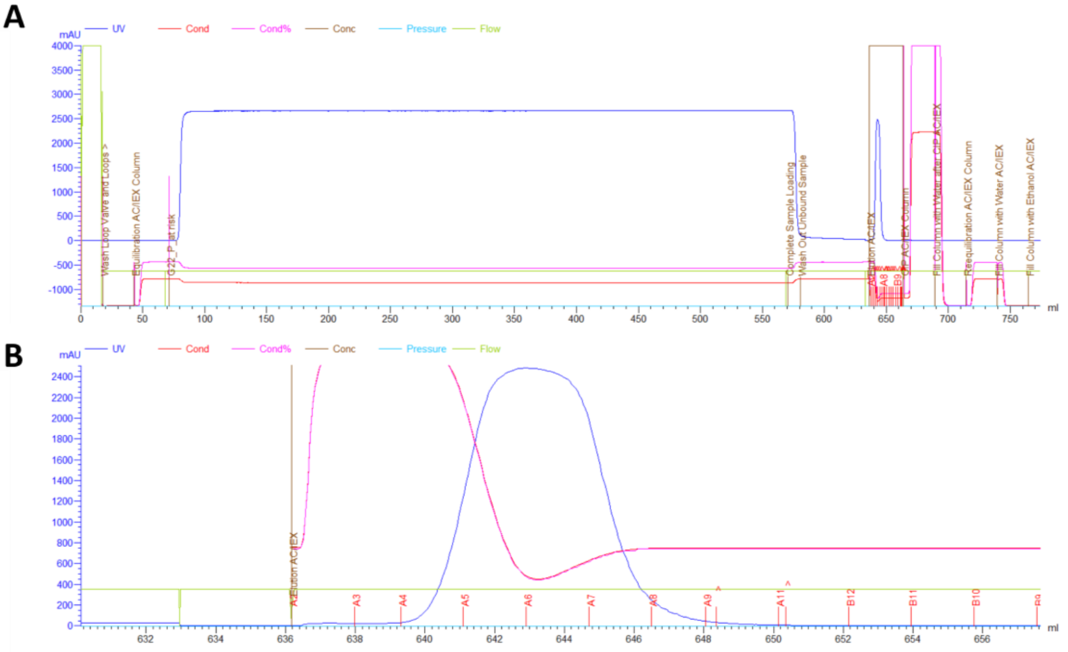

Figure S6: MAbSelect SuRe affinity chromatography purification trace (UV) of Fc protein: A) full trace; B) close-up of protein elution peak.

Bioconjugation Experimental

General Protocol

To a solution of Fc protein (obtained via recombinant expression or enzymatic digestion) (1.0 mg/mL) in TBS (25 mM Tris, 25 mM NaCl, 0.5 mM EDTA, pH 8) was added TCEP (5-10 eq. as 1 mM solution in H_2O). The mixture was vortexed and incubated at 37 °C for 1 h with shaking at 400 rpm. A solution of BisDVP **1** or **2** (10 mM in DMSO, 5-10 eq. added in one shot or sequentially as 5 eq. 1 h, 5 eq. 1h) was added with additional DMSO to reach 10% DMSO (v/v) and the reaction mixture incubated at 37 °C for 1 h with shaking at 400 rpm (note for BisDVP **3** reaction mixture should be incubated at rt). The excess reagents were removed by use of a Zeba™ Spin Desalting Column (40K MWCO, 0.5 mL), followed by repeated diafiltration into PBS with an Amicon-Ultra centrifugal filter (10K MWCO, Merck Millipore). Samples were stored at 4 °C until analysis.

Fc Conjugate (6); digest Fc

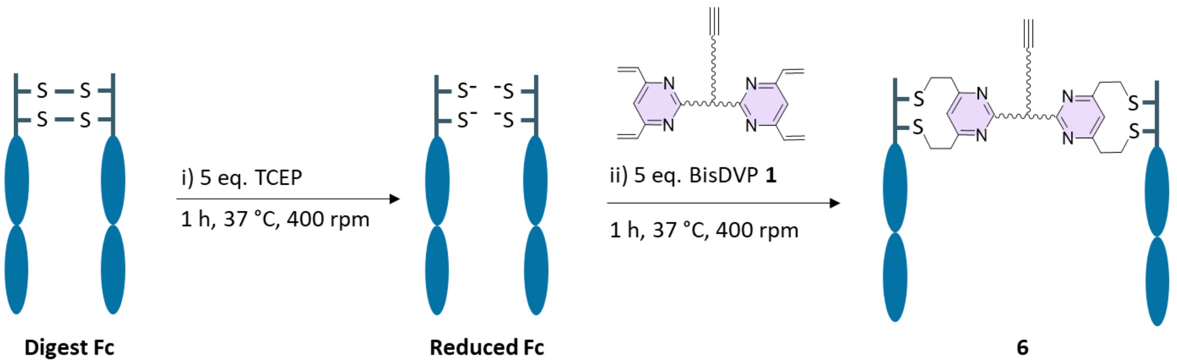

To a solution of IgG1 Fc protein (300 µL, 20 µM, 1.0 mg/mL) in TBS (25 mM Tris, 25 mM NaCl, 0.5 mM EDTA, pH 8) was added TCEP (5 eq. as 1 mM solution in H_2O). The mixture was vortexed and incubated at 37 °C for 1 h with shaking at 400 rpm. A solution of BisDVP **1** (10 mM in DMSO, 5 eq.) was added with additional DMSO to reach 10% DMSO (v/v) and the reaction mixture incubated at 37 °C for 1 h with shaking at 400 rpm. The excess reagents were removed by use of a Zeba™ Spin Desalting Column (40K MWCO, 0.5 mL), followed by repeated diafiltration into PBS with an Amicon-Ultra centrifugal filter (10K MWCO, Merck Millipore). Samples were stored at 4 °C until analysis.

Fc Conjugate (7); digest Fc

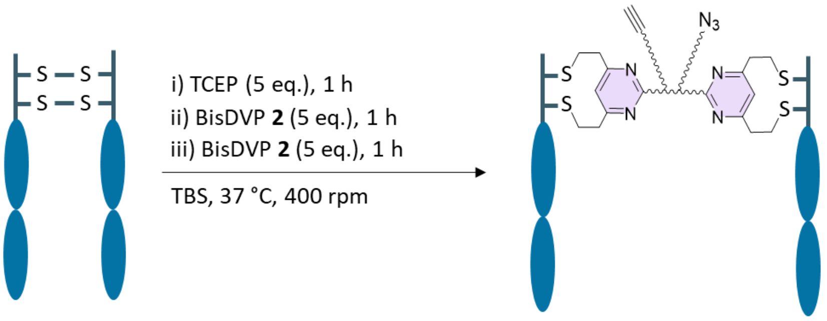

Digest Fc 7

To a solution of IgG1 Fc protein (120 µL, 19.5 µM, 0.99 mg/mL) in TBS (25 mM Tris, 25 mM NaCl, 0.5 mM EDTA, pH 8) was added TCEP (5 eq. as 1 mM solution in H_2O). The mixture was vortexed and incubated at 37 °C for 1 h with shaking at 400 rpm. A solution of BisDVP **2** (10 mM in DMSO, 5 eq.) was added with additional DMSO to reach 10% DMSO (v/v) and the reaction mixture incubated at 37 °C for 1 h with shaking at 400 rpm. Another portion of BisDVP **2** (10 mM in DMSO, 5 eq.) was added, and the reaction mixture incubated at 37 °C for 1 h with shaking at 400 rpm. The excess reagents were removed by use of a Zeba™ Spin Desalting Column (40K MWCO, 0.5 mL), followed by repeated diafiltration into PBS with an Amicon-Ultra centrifugal filter (10K MWCO, Merck Millipore). Samples were stored at 4 °C until analysis.

Fc Conjugate (8); recombinant Fc

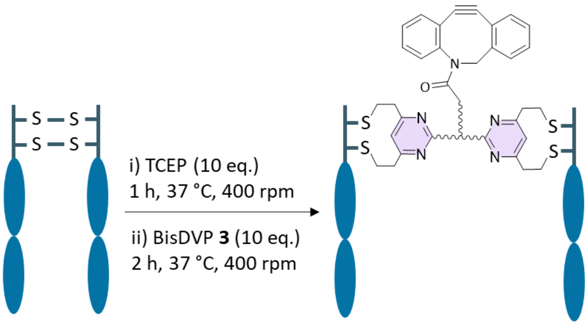

Recombinant Fc 8

To a solution of IgG1 Fc protein (94 µL, 20.8 µM, 1.00 mg/mL) in TBS (25 mM Tris, 25 mM NaCl, 0.5 mM EDTA, pH 8) was added TCEP (10 eq. as 1 mM solution in H_2O). The mixture was vortexed and incubated at 37 °C for 1 h with shaking at 400 rpm. A solution of **BisDVP 3** (10 mM in DMSO, 10 eq.) was added with additional DMSO to reach 10% DMSO (v/v) and the reaction mixture incubated at 37 °C for 2 h with shaking at 400 rpm The excess reagents were removed by use of a Zeba™ Spin Desalting Column (40K MWCO, 0.5 mL), followed by repeated diafiltration into PBS with an Amicon-Ultra centrifugal filter (10K MWCO, Merck Millipore). Samples were stored at 4 °C until analysis.

Fc-BisDVP-AlexaFluor488 (9)

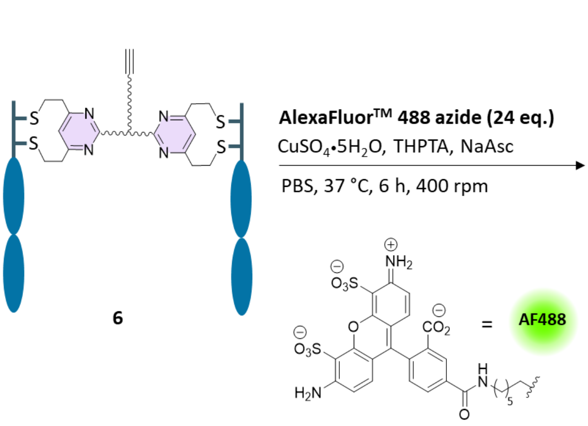

To a solution of Fc-BisDVP conjugate **6** (75 µL, 19.4 µM, 0.98 mg/mL) in PBS was added THPTA (20 mM in H_2O, 200 equiv.), $CuSO_4{\cdot}5H_2O$ (5 mM in H_2O, 40 equiv.), sodium ascorbate (50 mM in H_2O, 300 equiv.), and AlexaFluor488 Azide (5 mM in DMSO, 24 equiv.) and the reaction mixture was vortexed and incubated at 37 °C for 6 h with shaking at 400 rpm. The excess reagents were removed by filtration through two Zeba™ Spin Desalting Columns (40K MWCO, 0.5 mL), followed by repeated diafiltration into PBS with an Amicon-Ultra centrifugal filter (10K MWCO, Merck Millipore). Samples were stored at 4 °C until analysis.

Fc-BisDVP-Afamelanotide (10)

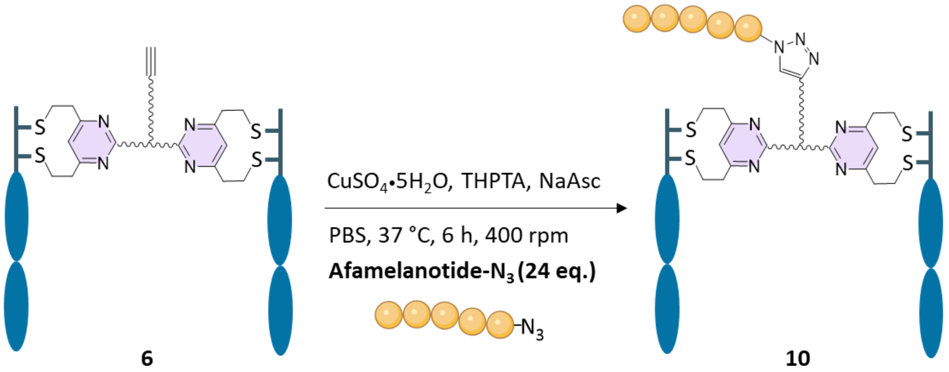

To a solution of Fc-BisDVP conjugate **6** (55 µL, 18.6 µM, 0.94 mg/mL) in PBS was added THPTA (200 equiv.), $CuSO_4{\cdot}5H_2O$ (40 equiv.), sodium ascorbate (300 equiv.), and Afamelanotide-azide (5 mM in DMSO, 24 equiv.) with additional DMSO to reach 10% DMSO (v/v) and the reaction mixture was vortexed and incubated at 37 °C for 6 h with shaking at 400 rpm. The excess reagents were removed by filtration through two Zeba™ Spin Desalting Columns (40K MWCO, 0.5 mL), followed by repeated diafiltration into PBS with an Amicon-Ultra centrifugal filter (10K MWCO, Merck Millipore). Samples were stored at 4 °C until analysis.

Fc-BisDVP Afamelanotide-AF488 (12)

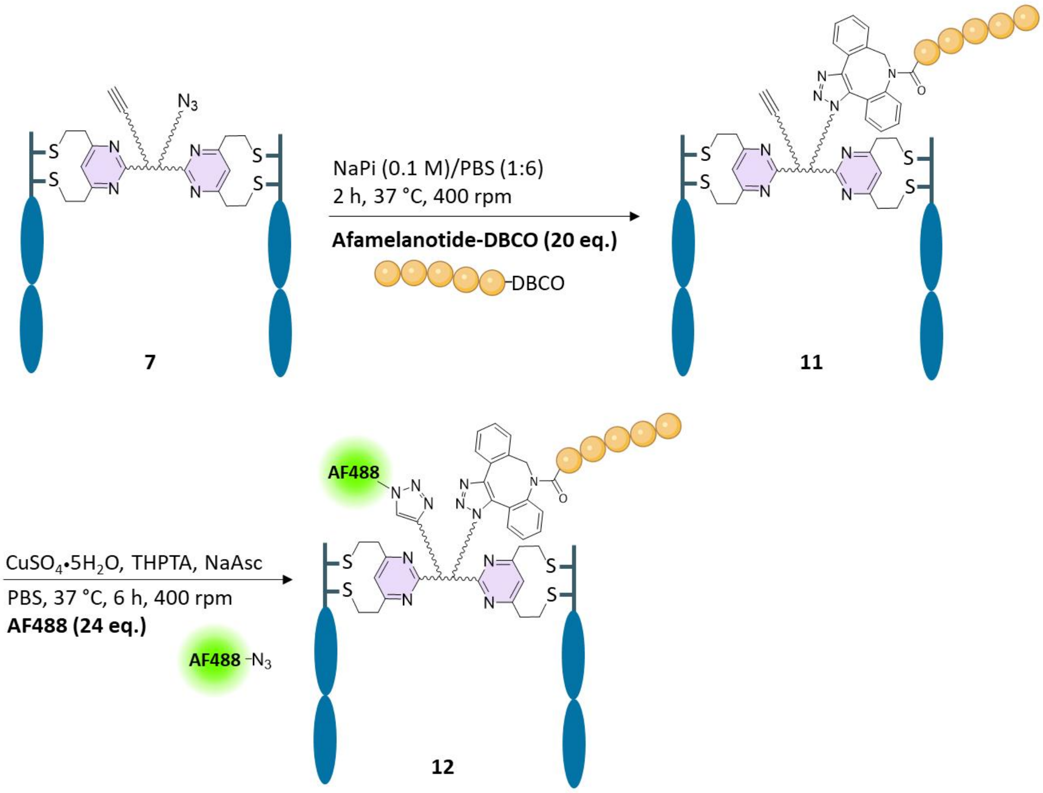

SPAAC: To a solution of Fc-BisDVP conjugate **7** (107 µL, 7.6 µM, 0.4 mg/mL) in PBS was added Afamelanotide-DBCO (5 mM in DMSO, 10 equiv.) and 0.1 M NaPi pH 7, to reach 15% (v/v). The reaction mixture was vortexed and incubated at 37 °C for 24 h with shaking at 400 rpm. The excess reagents were removed by filtration through Zeba™ Spin Desalting Columns (40K MWCO, 0.5 mL), followed by repeated diafiltration into PBS with an Amicon-Ultra centrifugal filter (10K MWCO, Merck Millipore). Samples were stored at 4 °C until analysis.

CuAAC: To a solution of Fc-BisDVP-Afamelanotide **11** (50 µL, 4.5 µM, 0.23 mg/mL) in PBS was added THPTA (200 equiv.), $CuSO_4{\cdot}5H_2O$ (40 equiv.), sodium ascorbate (300 equiv.), and AlexaFluor488 Azide (5 mM in DMSO, 24 equiv.) with additional DMSO to reach 5% DMSO (v/v) and the reaction mixture was vortexed and incubated at 37 °C for 6 h with shaking at 400 rpm. The excess reagents were removed by filtration through two Zeba™ Spin Desalting Columns (40K MWCO, 0.5 mL), followed by repeated diafiltration into PBS with an Amicon-Ultra centrifugal filter (10K MWCO, Merck Millipore). Samples were stored at 4 °C until analysis.

Fc-BisDVP-DBCO-Afamelanotide (13)

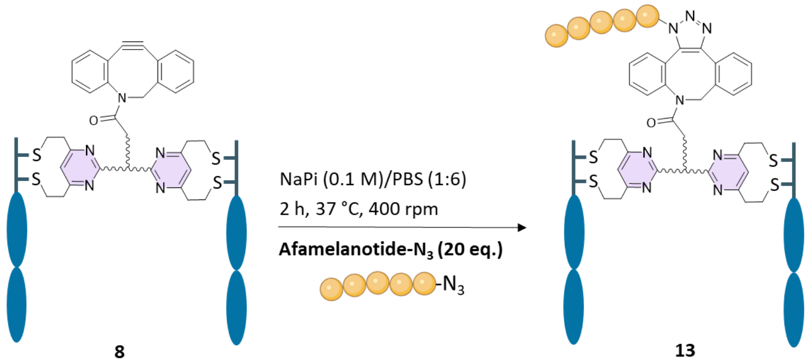

To a solution of Fc-BisDVP conjugate **8** (70 µL, 14.7 µM, 0.74 mg/mL) in PBS was added Afamelanotide-azide (10 mM in DMSO, 20 equiv.) and 0.1 M NaPi pH 7, to reach 15% (v/v). The reaction mixture was vortexed and incubated at 37 °C for 2 h with shaking at 400 rpm. The excess reagents were removed by filtration through Zeba™ Spin Desalting Columns (40K MWCO, 0.5 mL), followed by repeated diafiltration into PBS with an Amicon-Ultra centrifugal filter (10K MWCO, Merck Millipore). Samples were stored at 4 °C until analysis.

Fc BisDVP DBCO-Exenatide (15)

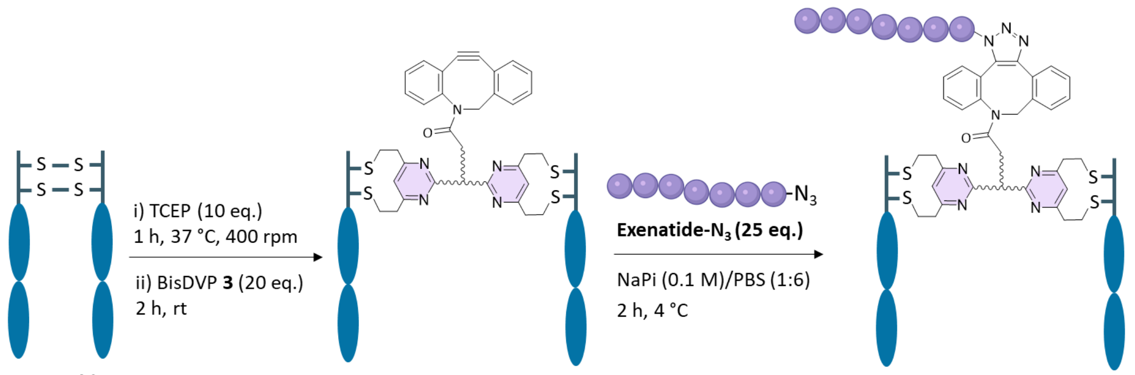

To a solution of IgG1 Fc protein (100 µL, 18 µM, 0.91 mg/mL) in TBS (25 mM Tris, 25 mM NaCl, 0.5 mM EDTA, pH 8) was added TCEP (10 eq. as 1 mM solution in H_2O). The mixture was vortexed and incubated at 37 °C for 1 h with shaking at 400 rpm. The reaction was then removed from heat and cooled to rt. A solution of **BisDVP 3** (10 mM in DMSO, 20 eq.) was added with additional DMSO to reach 10% DMSO (v/v) and the reaction mixture left at rt for 2 h. To this solution was added Exenatide-azide (10 mM in DMSO, 25 equiv.) and 0.1 M NaPi pH 7, to reach 15% (v/v). The reaction mixture was vortexed and left to react at 4 °C for 12 h. The excess reagents were removed by filtration through Zeba™ Spin Desalting Columns (40K MWCO, 0.5 mL), followed by repeated diafiltration into PBS with an Amicon-Ultra centrifugal filter (10K MWCO, Merck Millipore). Samples were stored at 4 °C until analysis.

Fc Maleimide-AF488 (S21)

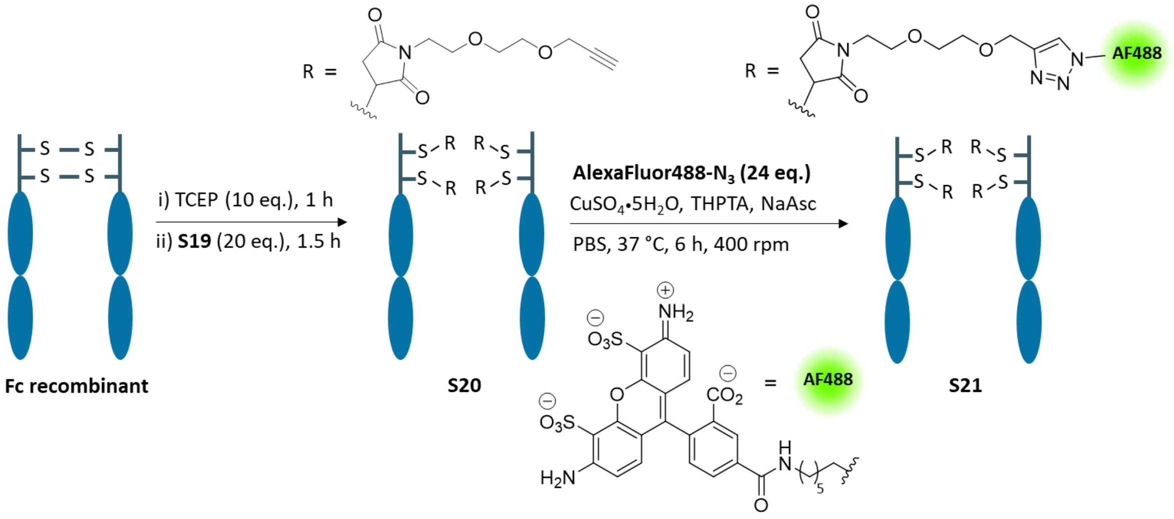

Conjugation: To a solution of IgG1 Fc protein (300 µL, 20.1 µM, 1.02 mg/mL) in TBS (25 mM Tris, 25 mM NaCl, 0.5 mM EDTA, pH 8) was added TCEP (10 eq. as 1 mM solution in H_2O). The mixture was vortexed and incubated at 37 °C for 1 h with shaking at 400 rpm. A solution of maleimide **S19** (10 mM in DMSO, 20 eq.) was added with additional DMSO to reach 10% DMSO (v/v) and the reaction mixture incubated at 37 °C for 1.5 h with shaking at 400 rpm. The excess reagents were removed by use of a Zeba™ Spin Desalting Column (40K MWCO, 0.5 mL), followed by repeated diafiltration into PBS with an Amicon-Ultra centrifugal filter (10K MWCO, Merck Millipore) to form **S20**. Samples were stored at 4 °C until analysis.

CuAAC: To a solution of Fc-maleimide conjugate **S20** (75 µL, 17.3 µM, 0.88 mg/mL) in PBS was added THPTA (20 mM in H_2O, 340 equiv.), $CuSO_4{\cdot}5H_2O$ (5 mM in H_2O, 68 equiv.), sodium ascorbate (50 mM in H_2O, 510 equiv.), and AlexaFluor488 Azide (5 mM in DMSO, 24 equiv.) with additional DMSO to reach 10% DMSO (v/v) and the reaction mixture was vortexed and incubated at 37 °C for 6 h with shaking at 400 rpm. The excess reagents were removed by filtration through two sequential Zeba™ Spin Desalting Columns (40K MWCO, 0.5 mL), followed by repeated diafiltration into PBS with an Amicon-Ultra centrifugal filter (10K MWCO, Merck Millipore) to form **S21**. Samples were stored at 4 °C until analysis.

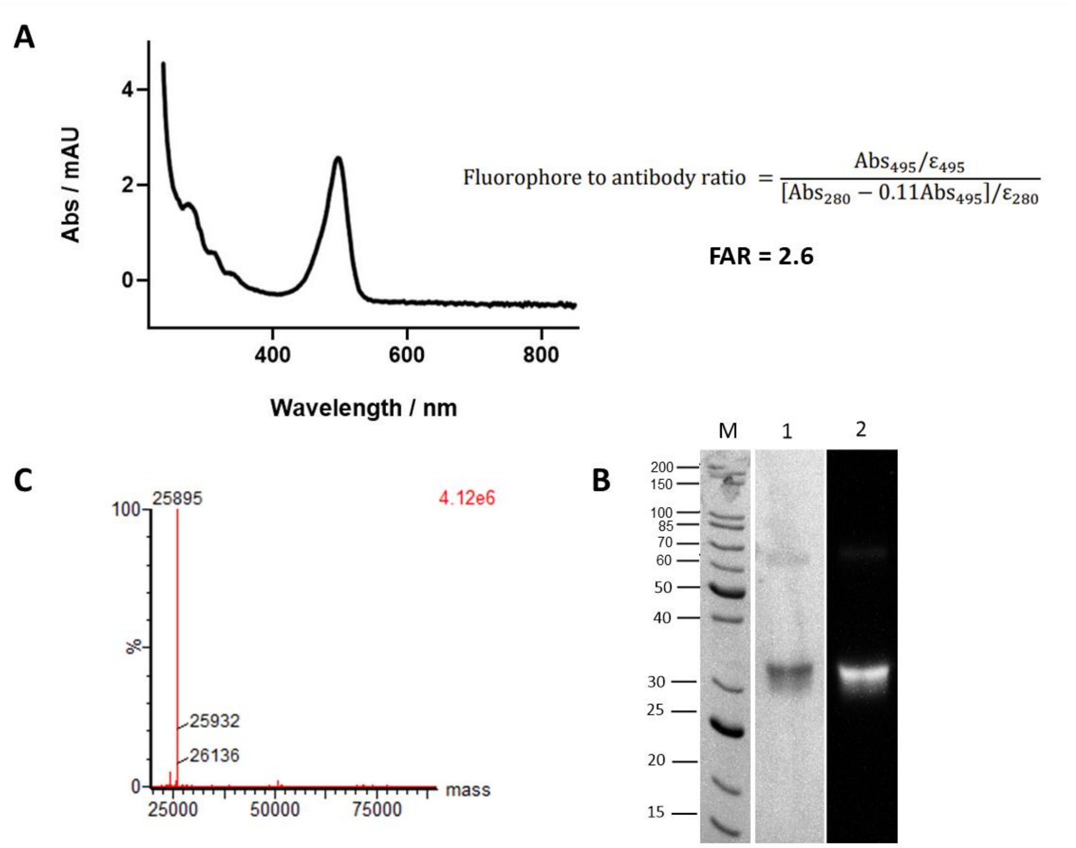

Figure S7: Analysis of Fc-maleimide-AF488 **S21** by UV-vis spectroscopy, LC-MS and SDS-PAGE. A) UV-vis spectrum. B) Analysis by SDS-PAGE on 12% polyacrylamide gel under reducing conditions. Lanes: M =molecular weight marker, 1 = **S21** (Coomassie staining), 2 = **S21** (in-gel fluorescence). C) Analysis by LC-MS, deconvoluted MS; expected 25892 Da and observed and 25895 Da.

Fc Conjugate (S22); recombinant Fc

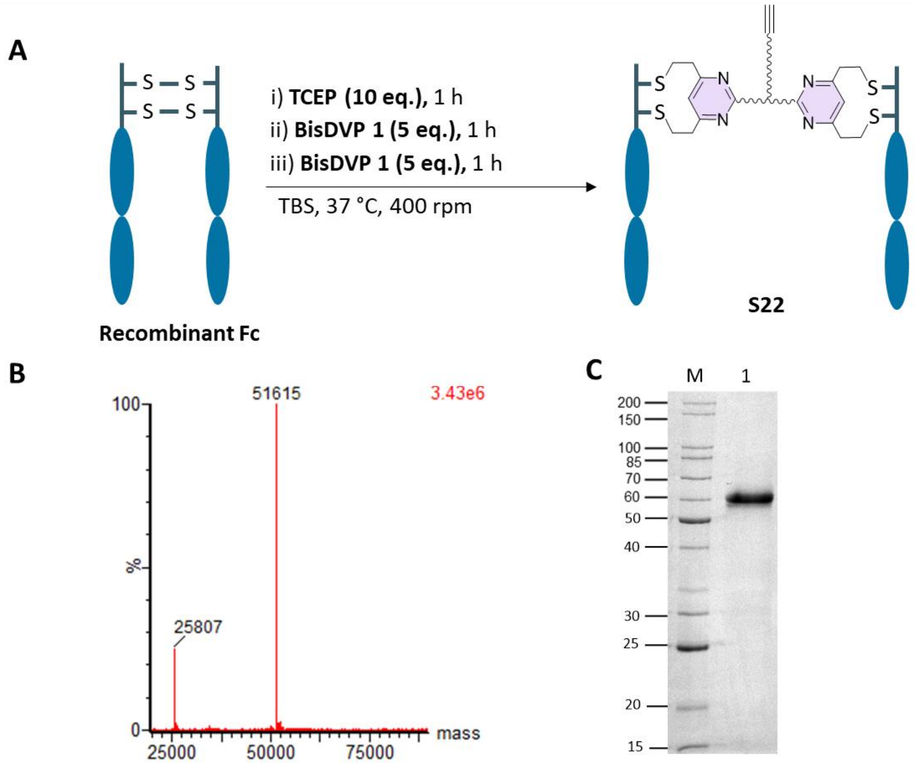

Figure S8: A) Bioconjugation of recombinant Fc **4** with BisDVP **1** to form **S22**. B) Deconvoluted mass spectrum of deglycosylated **S22**, expected mass: 51608 Da; found: 51615 Da. C) SDS-PAGE analysis (12% separating gel) of **S22** under reducing conditions. Lanes M) molecular weight ladder and 1) Fc-BisDVP-alkyne conjugate **S22**. Proteins were visualised by Coomassie blue stain.

To a solution of IgG1 Fc protein (300 µL, 20 µM, 1.0 mg/mL) in TBS (25 mM Tris, 25 mM NaCl, 0.5 mM EDTA, pH 8) was added TCEP (10 eq. as 1 mM solution in H_2O). The mixture was vortexed and incubated at 37 °C for 1 h with shaking at 400 rpm. A solution of BisDVP **1** (10 mM in DMSO, 5 eq.) was added with additional DMSO to reach 10% DMSO (v/v) and the reaction mixture incubated at 37 °C for 1 h with shaking at 400 rpm. Another portion of BisDVP **1** (10 mM in DMSO, 5 eq.) was added, and the reaction mixture incubated at 37 °C for 1 h with shaking at 400 rpm. The excess reagents were removed by use of a Zeba™ Spin Desalting Column (40K MWCO, 0.5 mL), followed by repeated diafiltration into PBS with an Amicon-Ultra centrifugal filter (10K MWCO, Merck Millipore). Samples were stored at 4 °C until analysis.

Fc Conjugate (S23); recombinant Fc

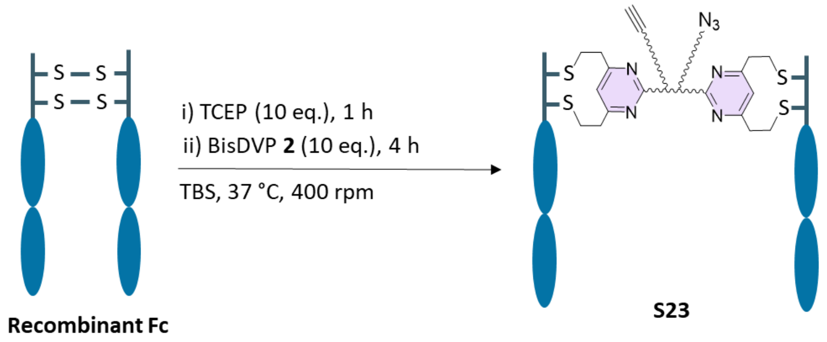

To a solution of IgG1 Fc protein (150 µL, 19.1 µM, 0.97 mg/mL) in TBS (25 mM Tris, 25 mM NaCl, 0.5 mM EDTA, pH 8) was added TCEP (10 eq. as 1 mM solution in H_2O). The mixture was vortexed and incubated at 37 °C for 1 h with shaking at 400 rpm. A solution of BisDVP **2** (10 mM in DMSO, 10 eq.) was added with additional DMSO to reach 10% DMSO (v/v) and the reaction mixture incubated at 37 °C for 4 h with shaking at 400 rpm. The excess reagents were removed by use of a Zeba™ Spin Desalting Column (40K MWCO, 0.5 mL), followed by repeated diafiltration into PBS with an Amicon-Ultra centrifugal filter (10K MWCO, Merck Millipore). Samples were stored at 4 °C until analysis.

Fc-BisDVP-DBCO-Afamelanotide (S25); digest Fc

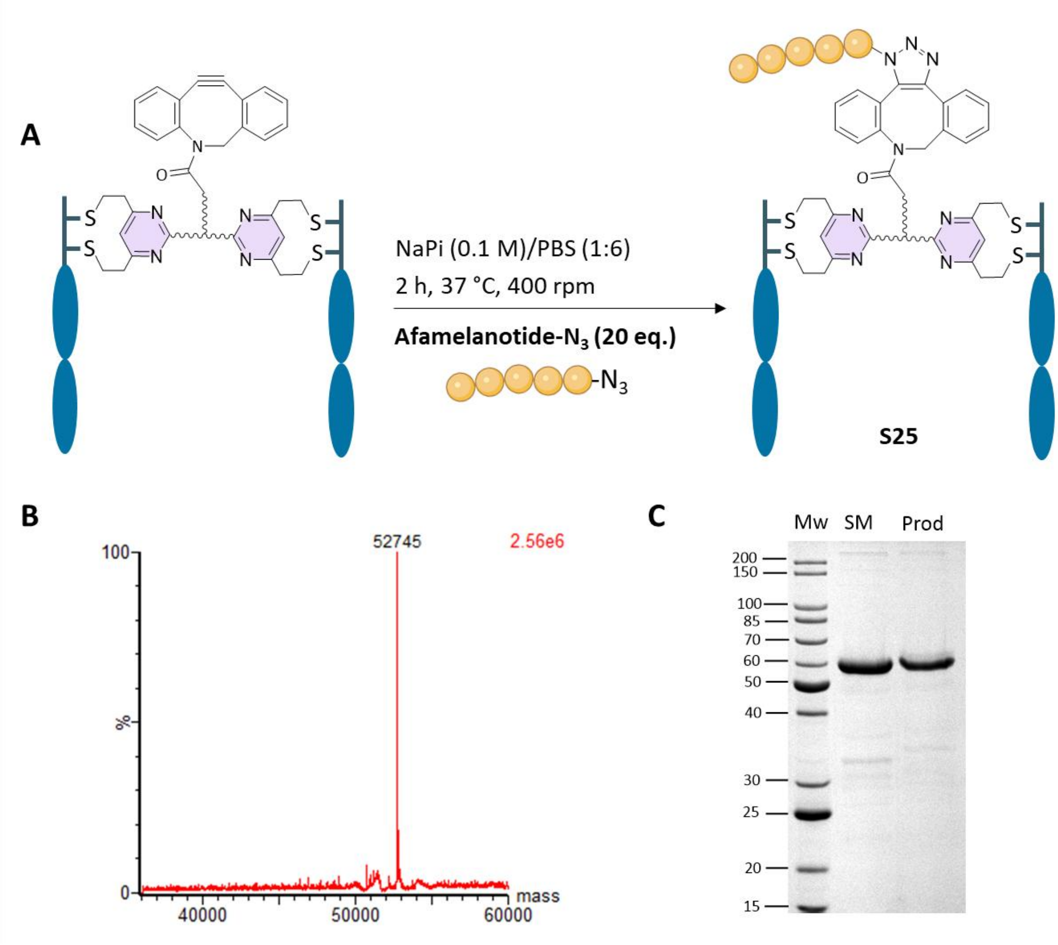

Figure S9: Analysis of Fc-Afamelanotide (digest Fc) **S25** by LC-MS and SDS-PAGE. (A) SPAAC of Fc-BisDVP-DBCO to form Fc-Afamelanotide **S25**. (B) Analysis of **S25** by LC-MS, deconvoluted MS; expected 52750 Da and observed 52745 Da. Peaks < 10% not annotated for clarity - full annotated spectrum available in section: mass spectrometry traces. (C) Analysis of **S25** by SDS-PAGE on 12% polyacrylamide gel. Lanes: Mw = molecular weight marker, SM = Fc-BisDVP-DBCO, Prod = Fc-Afamelanotide **S25.**

To a solution of Fc-BisDVP conjugate (70 µL, 14.7 µM, 0.74 mg/mL) in PBS was added Afamelanotide-azide (10 mM in DMSO, 20 equiv.) and 0.1 M NaPi pH 7, to reach 15% (v/v). The reaction mixture was vortexed and incubated at 37 °C for 2 h with shaking at 400 rpm. The excess reagents were removed by filtration through Zeba™ Spin Desalting Columns (40K MWCO, 0.5 mL), followed by repeated diafiltration into PBS with an Amicon-Ultra centrifugal filter (10K MWCO, Merck Millipore). Samples were stored at 4 °C until analysis.

Selectivity Control Experiments

SDS-PAGE analysis under reducing conditions demonstrated minimal formation of "half-Fc" species, indicating that the predominant Fc-conjugate product resulted from successful rebridging of the interchain disulfide bonds within the hinge region, thereby restoring covalent linkage between the two Fc heavy chains. The extent of this interchain rebridging was subsequently evaluated in greater detail.

Extent of Re-bridging

The connectivity of the Fc-BisDVP conjugate was studied to ensure that full re-bridging was occurring. The masses of the desired fully re-bridged product and an isomer in which only one of the reduced disulfides is re-bridged (Figure S6A) are the same, but the latter contains two reactive vinyl groups. To studiy whether this latter isomer was being formed, a vinyl quenching experiment was run. Fc protein (digest and recombinant) was modified with BisDVP-alkyne **1** and 100 equivalents of *N*-(tert-Butoxycarbonyl)-L-cysteine methyl ester were added to the purified reaction mixture to quench any unreacted vinyl groups which may be present in the conjugate. LC-MS analysis of the products revealed only the masses of the desired fully re-bridged conjugate, suggesting the partially re-bridged isomer is not formed.

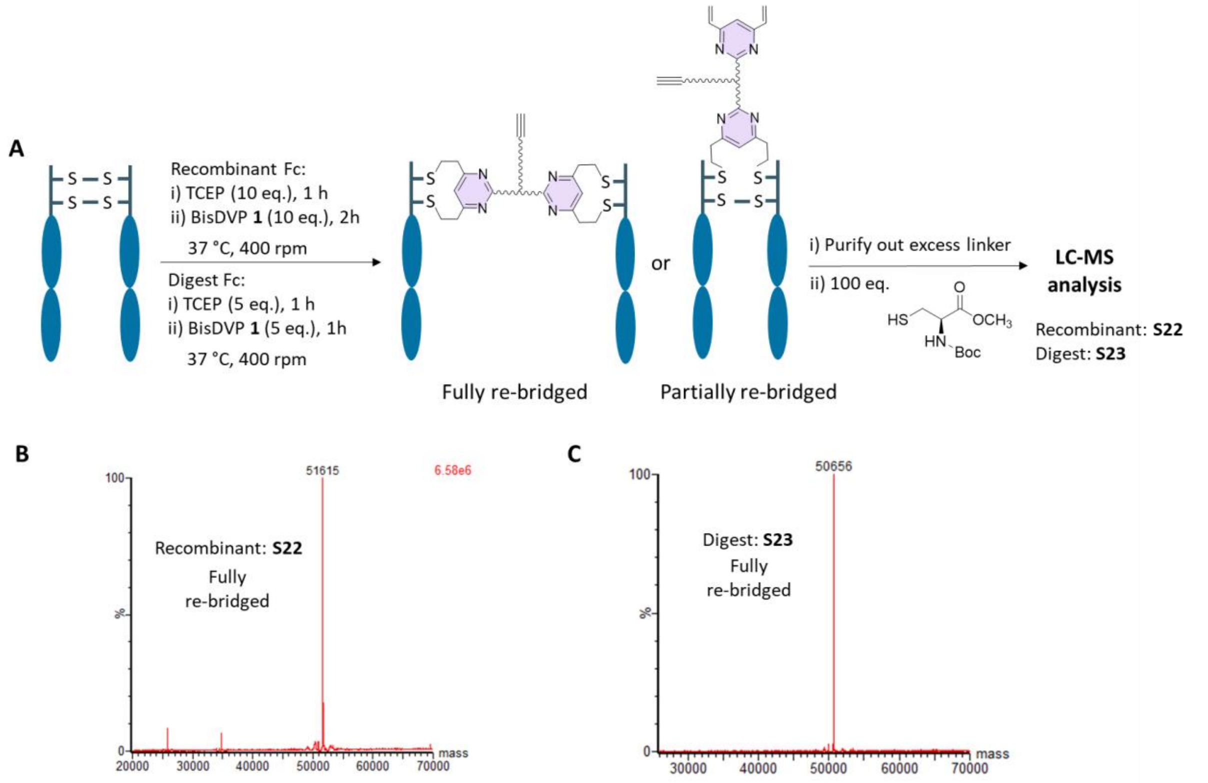

Figure S10: A) Bioconjugation quenched with N-(tert-Butoxycarbonyl)-L-cysteine methyl ester. B) Mass spectrum of the deglycosylated reaction of recombinant Fc-BisDVP (**S22**) with N-(tert-Butoxycarbonyl)-L-cysteine methyl ester, expected mass: 51606 Da, found: 51615 Da. C) Mass spectrum of the deglycosylated reaction of digest Fc-BisDVP (**S23**) with N-(tert-Butoxycarbonyl)-L-cysteine methyl ester, expected mass: 50649 Da, found: 50656 Da.

Regioselectivity

Since there are four thiols free for reaction with the four vinyl groups of the BisDVP linker, it is possible that the linker may conjugate in different regioselectivities (Figure S7). From a therapeutic standpoint, the regioselectivity is not a concern since the different regioisomers should not possess different pysiological properties. When the Fc protein is reduced, the heavy chains are held in place by non-covalent interactions, and thus the linker fits with the structure of the Fc protein as opposed to the linker dictating the structure of the Fc protein.

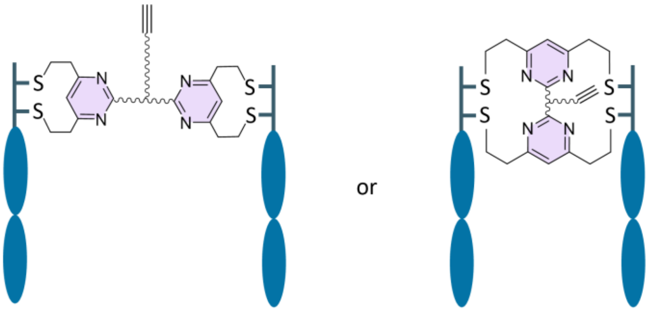

Figure S11: Illustration of two possible geometries of Fc-BisDVP conjugate.

An experiment to assess the regioselectivity of the Fc-BisDVP conjugation was carried out which involved bioconjugation of the Fc protein with a mono DVP reagent **S3**. This demonstrated it is equally likely for the DVP to re-bridge intrachain or interchain as SDS-PAGE analysis showed both a band corresponding to two Fc heavy chains individually re-bridged with DVP (~25 Da), and Fc protein where the DVP had re-bridged between the two heavy chains covalently binding them together (~51 Da) (Figure S8).

The two disulfide bonds of the Fc protein were reduced using 10 equivalents of TCEP and, after 1 hour, 20 equivalents of DVP **S3** were added to the reaction mixture. LC-MS analysis was carried out to ensure that addition of two linkers and no over addition or under addition had occurred. SDS-PAGE analysis of Fc protein under reducing conditions shows the presence of individual Fc heavy chains at ~26.5 kDa (Lane 1, Figure S8C). Analysis of the reaction mixture under reducing conditions revealed a band at ~55 kDa corresponding to Fc-DVP conjugate with interchain re-bridging (Lane 2, Figure S8C) and also a band at ~26.5 kDa, which may correspond to individual unmodified Fc heavy chains or Fc heavy chains where the DVP has undergone intrachain re-bridging. SDS-PAGE analysis of Fc protein under non-reducing conditions shows a band at ~50 kDa as expected (Lane 3, Figure C). Finally, analysis of the reaction mixture under non-reducing conditions showed a band at ~50 kDa, which may be unmodified Fc protein and/or Fc-DVP employing interchain rebridging to covalently bind the two heavy chains. Additionally, a band at ~25 kDa was observed under non-reducing conditions, which likely corresponds to “half-Fc” species of 42 (Lane 4, Figure S8C). This can be concluded since under non-reducing conditions anything held together by disulfides or covalent bonds will remain bound together, whereas the half-Fc is only held together via non-covalent interactions, which upon heating at 90 °C for 5 mins denature and break the interactions to give the species as the individually modified heavy chains. Analysis under reducing and non-reducing conditions was required since unmodified Fc protein was also present. Since the protein samples were

loaded at the same concentration, comparison between the two runs can be made. The band for each re-bridged form appeared to be similar intensities and thus similar in concentration suggesting that there is a similar proportion of each species present. Therefore, it could be assumed that for the Fc-BisDVP conjugates there is roughly an even mixture of the two different regioisomers.

To a solution of IgG1 Fc protein (80 µL, 20.7 µM, 1.05 mg/mL) in TBS (25 mM Tris, 25 mM NaCl, 0.5 mM EDTA, pH 8) was added TCEP (10 eq. as 1 mM solution in H_2O). The mixture was vortexed and incubated at 37 °C for 1 h with shaking at 400 rpm. A solution of **S3** (10 mM in DMSO, 10 eq.) was added with additional DMSO to reach 10% DMSO (v/v) and the reaction mixture incubated at 37 °C for 2 h with shaking at 400 rpm. The excess reagents were removed by use of a Zeba™ Spin Desalting Column (40K MWCO, 0.5 mL), and N-(tert-Butoxycarbonyl)-L-cysteine methyl ester (100 eq., as a 40 mM solution in DMSO) was added and allowed to react at rt for 30 minutes. Samples were stored at 4 °C until analysis.

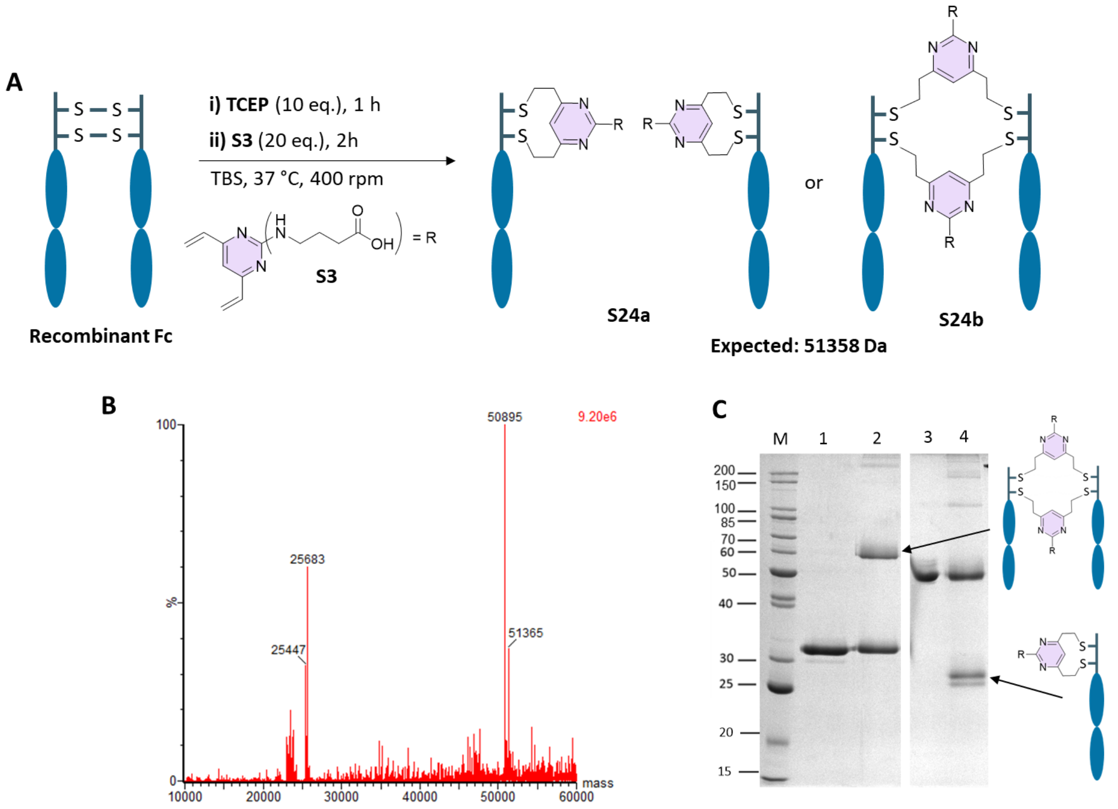

Figure S12: A) Reaction of Fc protein (recombinant) with DVP **S3** to form S24a/S24b. B) Deconvoluted mass spectrum of deglycosylated bioconjugation product, expected mass: 51358 Da; found: 51365 Da (**S24a**/**S24b**) and 50895 Da (unmodified Fc). C) SDS-PAGE analysis (12% separating gel). Lanes M) molecular weight ladder and 1) Fc-protein (reducing), 2) Fc-DVP (**S24a**/**S24b**) (reducing), 3) Fc protein (non-reducing), 4) Fc-DVP (**S24a**/**S24b**) (non-reducing). Proteins were visualised by Coomassie blue stain.

Chemoselectivity

Unreduced Fc contains no free thiols, therefore any reaction between the linker and unreduced Fc would imply modification of amino acid residues other than cysteine. LC-MS analysis of the reaction mixtures for both Fc protein types showed only presence of unmodified Fc protein, indicating that no reaction had taken place, and thus validating the chemoselectivity of the BisDVP linker for free thiols.

Recombinant Fc: To a solution of IgG1 Fc protein (100 µL, 20.7 µM, 1.05 mg/mL) in TBS (25 mM Tris, 25 mM NaCl, 0.5 mM EDTA, pH 8) was added **BisDVP 1** (10 mM in DMSO, 10 eq.) was added with additional DMSO to reach 10% DMSO (v/v) and the reaction mixture incubated at 37 °C for 2 h with shaking at 400 rpm. The excess reagents were removed by use of a Zeba™ Spin Desalting Column (40K MWCO, 0.5 mL), followed by repeated diafiltration into PBS with an Amicon-Ultra centrifugal filter (10K MWCO, Merck Millipore). Samples were stored at 4 °C until analysis. No reaction was observed.

Digest Fc: To a solution of IgG1 Fc protein (14.9 µL, 19 µM, 0.96 mg/mL) in TBS (25 mM Tris, 25 mM NaCl, 0.5 mM EDTA, pH 8) was added **BisDVP 1** (10 mM in DMSO, 5 eq.) was added with additional DMSO to reach 10% DMSO (v/v) and the reaction mixture incubated at 37 °C for 2 h with shaking at 400 rpm. The excess reagents were removed by use of a Zeba™ Spin Desalting Column (40K MWCO, 0.5 mL), followed by repeated diafiltration into PBS with an Amicon-Ultra centrifugal filter (10K MWCO, Merck Millipore). Samples were stored at 4 °C until analysis. No reaction was observed.

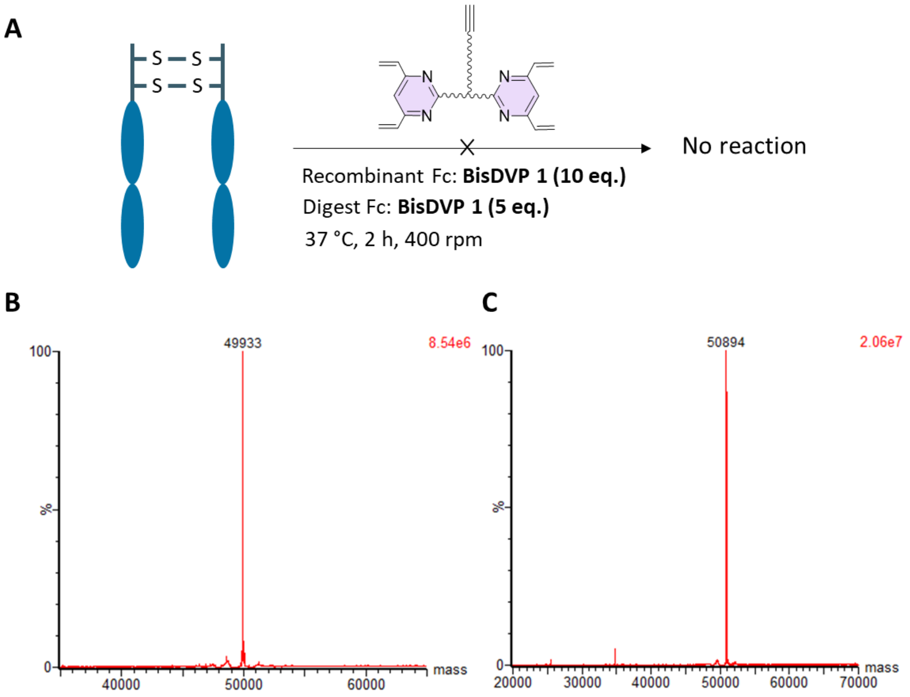

Figure S13: A) Treatment of unreduced trastuzumab Fc with **BisDVP 1**. B) Deconvoluted mass spectrum of deglycosylated reaction mixture of non-reduced digest Fc and 5 equivalents of **BisDVP 1**, 2 h, 37 °C, expected

mass: 49923 Da; found: 49932 Da. C) Deconvoluted mass spectrum of deglycosylated reaction mixture of non-reduced recombinant Fc and 10 equivalents of **BisDVP 1**, 2 h, 37 °C, expected mass: 50891 Da; found: 50894 Da.

Biological Studies

Human Plasma Stability Study

To a solution of Fc-AlexaFluor™ 488 conjugate **9** (BisDVP conjugated) or **S21** (maleimide conjugated) (20 µL,) in PBS were added 2.22 µL of reconstituted human plasma (Sigma). The mixture was incubated at 37 °C for 14 days. Aliquots of 2.2 µL were removed after 0, 4, 6, 8, 10, 12 and 14 days, flash frozen and stored at -80 °C until analysis. SDS-PAGE was followed by in-gel fluorescence imaging and Coomassie brilliant blue staining and imaging.

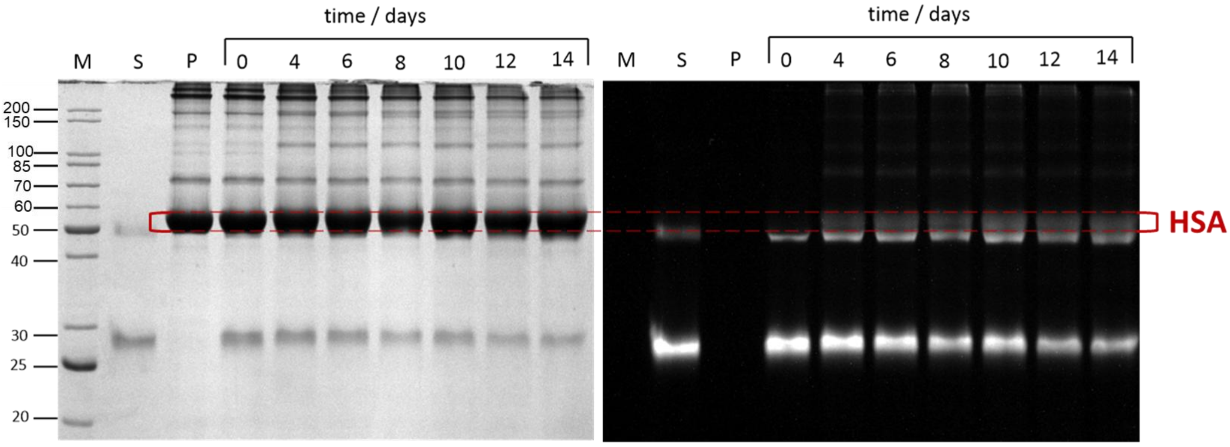

Figure S14: Stability analysis of Fc-maleimide-AF488 **S21** in human plasma by SDS-PAGE, non-reducing conditions. Lanes: M = molecular weight marker, S = Fc-maleimide-AF488 **S21**, P = human plasma, days of incubation are depicted above the representative lane. Left gel is after Coomassie staining, right gel is in-gel fluorescence measured before staining. Transfer of AlexaFluor™ 488 to human serum albumin (66.5 kDa, indicated by the red box) was observed after 4-days into the incubation period.

Human FcRn Binding Assay

The Promega Lumit™ FcRn Binding Immunoassay was carried out following the technical manual. **Preparing Tracer-LgBiT:** To a reagent reservoir was added 1.9 mL of FcRn assay buffer. To this was added 28.5 µL of Tracer-LgBit. The contents of the reservoir were mixed by pipetting. **Preparing hFcRn-SmBiT:** To a second reservoir was added 3.7 mL of FcRn assay buffer. To this was added 4.625 µL of hFcRn-Biotin and 4.625 µL of Streptavidin-SmBiT. The contents of the reservoir were mixed by pipetting. **Assay run:** First, 12.5 µL of Tracer-LgBiT was pipetted into all wells of a white 96-well plate. Next, 12.5 µL of control antibody or sample (of each concentration) was added to each well. To each well was then added 25 µL of hFcRn-Sm-BiT solution. The plate was then covered with a plate seal and mixed gently on a plate shaker (300-400 rpm) for 30-60 minutes at room temperature. To a reservoir 1.825 mL of FcRn assay buffer was added. To this was then added 36.5 µL of Lumit FcRn detection substrate A to create the Lumit FcRn detection reagent. The contents of the reservoir were mixed by pipetting. Next, 12.5 µL of Lumit FcRn detection reagent was added to each well. The plate was incubated at

room temperature for 3-5 minutes and then read using a Luminometer. Data was then presented as a graph of normalised luminescence representing FcRn binding of each species.

cAMP Based Potency Assay

Assay for the activation (EC50) of CHO cells overexpressing the glucagon like peptide 1 receptor (GLP-1R) through measurement of cAMP accumulation, general protocol.

Materials:

Reagent	Source	Cat No
GLP-1 (7-36) amide (0774) (09WI)	Bachem	#H-6795
DMSO	Sigma	D8418
Albumin from Bovine Serum	Sigma	# A9418
Albumin from Mouse Serum	Equitech-Bio	# MSA62-1202
IBMX- 3-Isobutyl-1-methylxanthine	Sigma	# I7018
ECHO 384 well PP dilution plates	Labcyte Inc.	#P-05525
opaque u-bottomed 96 well dilution plates	Greiner	#650201
black shallow-well u-bottom 384-well HTRF Assay Plates	Corning	#4514
cAMP dynamic 2 HTRF kit	CisBio	# 62AM4PEC
HEPES	Sigma	#H3375
HBSS	Sigma	#H826-500ML

Assay buffer: HBSS + 0.1% BSA + 25 mM HEPES pH 7.4. + 0.1% IBMX

Sample dilutions:

Top dilutions prepared in column 1, 5 and 9 of 96 well plates peptides pre-diluted 1:100 in DMSO or 0.1% BSA assay buffer. Columns 2-4, 6-8 and 10-12 were filled with 198 µl of DMSO or assay buffer. All samples then were serially diluted 1:100, 3 times, in DMSO or 0.1% BSA assay buffer. At least 35 µl of all these dilutions were transferred to a 384-well Echo source plate as follow (interleaved). 35 µl 0.1% BSA assay buffer was added to columns 20-23 in each Echo plate. Cell line controls were placed in column 24 A to F. Echo protocol to fire assay buffer to backfill wells with lower volume of compound (to have the same volume in every well).

Cell Culture Cryopreserved:

One vial of each cell line was thawed for <5 minutes at 37ºC in water bath. Half a vial (0.5 mL) of cells was transferred in a falcon tube with 4ml of serum free assay buffer. 50 µl cell stock was diluted 1 in 2 into Trypan blue before reading on Celleca cell counter (viability and viable cell density) using 1:2. The cells were centrifuged for 5 minutes at 1200 rpm. Supernatant was discarded, tubes flicked to resuspend the cells.
Cells resuspended in serum-free assay buffer at 2x the required concentration.

cAMP HTRF 2-step protocol: Completed as per manufacturers' guidelines:

cAMP assay kits brought up to RT for > 30 min prior to use.
HTRF reagent (cryptate and d2) made up 1/20 dilution in Lysis buffer.
Manually add 2.5uL/well of d2 reagent to wells E23-P24 of Assay Plates.
Manually add 2.5uL /well of Lysis Buffer to wells A23-D24 of Assay Plates / cAMP standard curve plate.
Combi-Drop dispenser used to add 2.5uL /well anti-cAMP d2 reagent to Columns 1-22 of Assay Plates.
Combi-Drop dispenser used to add 2.5uL /well anti-cAMP cryptate reagent to all wells of 384 Assay Plates.
Assay plates incubated at Room Temperature for 1 hour.

Read plates on Envision L and measured FRET signal using the parameters detailed below.
Excitation: 320nm. Emission:620 nm & 665nm

Data analysis: Control wells used to normalise for differences in Max values by different cell lines. In data analysis, NSB (Min) wells (Cryptate only, no d2 therefore no FRET) are used to subtract background from all wells as part of the %DeltaF calculation.

Table 14: Sample plan for cAMP assay.

Sample	Plate Dilution	Stock Concentration (M)	Top Concentration
GLP1 (Reference)	1 in 100 (2 ul + 198)	2.64E-04	2.64E-08
Exenatide	1 in 100 (2 ul + 198)	4.83E-04	4.83E-08
'Dulaglutide'	1 in 100 (2 ul + 198)	2.16E-04	2.16E-08
Fc- Exenatide	1:10 (8 ul + 72)	6.96E-05	6.96E-08
Exenatide-PEG24-N_3	1 in 100 (2 ul + 198)	1.00E-04	1.00E-08

In Vivo Study

All animal experiments were conducted under the authority of the UK Home Office in compliance with the Animals (Scientific Procedures) Act 1986 (ASPA), under project license number PP5753595. The study was carried out at the approved establishment in the University of Cambridge and adhered to the 3Rs principle (Replacement, Reduction, Refinement). All procedures were performed in accordance with institutional guidelines for the care and use of laboratory animals (Animal Welfare & Ethical Review Body).

PK Study

Female C57BL/6J mice (6-8 weeks old) were purchased from Charles River. The animals were verified as pathogen free and in excellent health. The mice were acclimatised to their environment for 10 days prior to the study.

Eight female C57BL/6 mice (632, Charles River Laboratories, UK), aged 56-62 days, were used in this study. The animals were housed in a temperature- and humidity-controlled environment (21 ± 2°C, 55 ± 10% humidity) with a 12-hour light/dark cycle and provided with ad libitum access to standard rodent chow and water.

Initially, blood was sampled from a superficial vessel to provide a baseline measurement. Two days later, Exenatide, Fc-Exenatide or PBS was dosed at 25 nmol/kg *via* intravenous administration. Blood was then sampled from a superficial vessel at 1, 12, 48, 72, 96, 120, 196 hours. Body weight was also measured prior to dosing and up to 120 hours post treatment. Samples underwent centrifugation to obtain the plasma fraction which was removed, flash frozen and stored at -80 °C in lithium heparin collection tubes until analysis.

Table S1: *In vivo* study details, of dose given to each mouse, strain type and sex. All mice were of wildtype genotype. Both Exenatide and Fc-Exenatide were dosed at 25 nmol/kg.

Animal Barcode	Exenatide (0.0982 mg/mL) Dose	Fc-Exenatide (0.947 mg/mL) Dose	PBS (vehicle) Dose	Mouse Earmark	Strain	Sex	Age
M01221636			34 uL	NM245	C57BL/6J	Female	8w 1d
M01221637		32 uL		BL245	C57BL/6J	Female	8w 1d
M01221638	23 uL			TL245	C57BL/6J	Female	8w 1d
M01221639			34 uL	BR345	C57BL/6J	Female	8w 1d
M01221640	22 uL			NM246	C57BL/6J	Female	8w 1d
M01221641		34 uL		BL246	C57BL/6J	Female	8w 1d
M01221642	22 uL			TL246	C57BL/6J	Female	8w 1d
M01221643		32 uL		BR246	C57BL/6J	Female	8w 1d

Method outline for analysis of in-vivo stability

LC-MS peptide mapping was the chosen method of analysis for the plasma samples. Calibration standards were prepared in mouse plasma, and extracted using an immunodepletion method. Anti- human Fc antibody (Jackson Immunoresearch) was coupled to magnetic beads, which was used to extract the Fc-Exenatide molecule from mouse plasma. Fc-Exenatide extracts were then washed, reduced, alkylated and digested with trypsin to generate proteotypic peptides. After digestion, a target peptide sequence is used for quantification with LC-MS on a triple quadrupole mass spectrometer. Peptides selected for LC-MS/MS analysis included the N-terminal sequence of exenatide, a peptide in the centre of exenatide, three different peptides from the human heavy chain backbone as well as a peptide from the goat anti-human antibody to use as an internal standard.

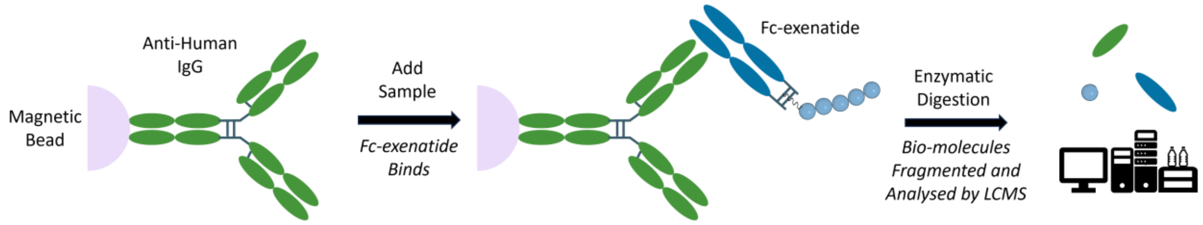

Figure S15: General method for analysing the *in vivo* samples.

Calibration standards are prepared in mouse plasma. Samples and calibration standards are incubated with magnetic beads that have anti human IgG antibody conjugated to them. All calibration standards and samples are prepared in duplicate. The beads are washed to remove any unbound material. The beads are reduced, alkylated and digested with trypsin. After digestion, a target peptide sequence is used for quantification with LCMS. A separate peptide sequence from the target antibody is used as a control. The target peptide and control peptide are compared to give ratio of intact target antibody

The immunoaffinity based extraction of the Fc-Exenatide molecule from mouse plasma followed by tryptic digestion and LC-MS/MS analysis allowed the detection of the drug at 25 ng/mL in mouse plasma (400 pM). This approach enables the determination of the concentration of both Exenatide and Fc derived peptides in extracted mouse plasma, demonstrating if the molecule is intact.

Both the Fc-Exenatide and Exenatide were dosed at 25 nmol/kg, with the dose calculated using the bodyweight of the mouse on the day of administration. Blood samples were taken from each mouse at several timepoints over 8 days. All desired samples were collected, except the 24-hour timepoint due to insufficient bleeding at the vein for most of the mice.

Table S2: Weight of mice throughout study.

	Weight (grams)						
**Animal Barcode**	**Baseline**	**24hrs**	**48hrs**	**72hrs**	**96hrs**	**120hrs**	**Dosed with**
M01221636	19.9	19.3	19.2	18.9	18.7	18.4	PBS
M01221637	20.9	19.3	18.7	18.5	18.1	17.9	Fc-Ex
M01221638	21.2	20.3	19.8	20.1	19.9	19.5	Ex
M01221639	20.5	21.1	20.1	19.8	20.1	20.5	PBS
M01221640	20.4	19.5	19.5	20.1	19.5	19.4	Ex
M01221641	22.8	20.9	19.7	19.3	19.2	19.1	Fc-Ex
M01221642	20.8	19.3	19.3	19.1	19.3	19.2	Ex
M01221643	20.8	19.8	19.3	19.9	19.1	18.8	Fc-Ex

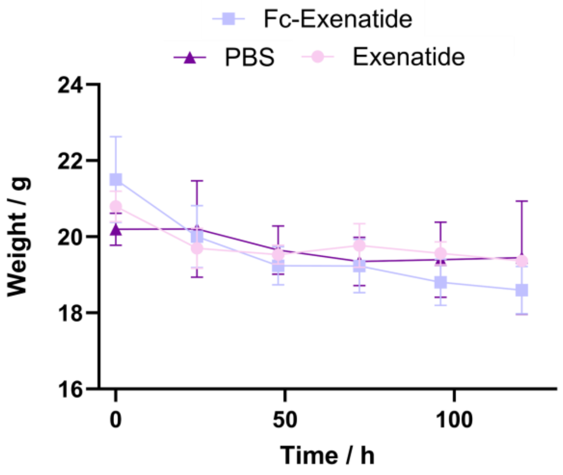

Figure S16: Mouse body weight from baseline (0 hours) up to 120 hours post-treatment with **Fc-Exenatide**, **Exenatide** or **PBS**.

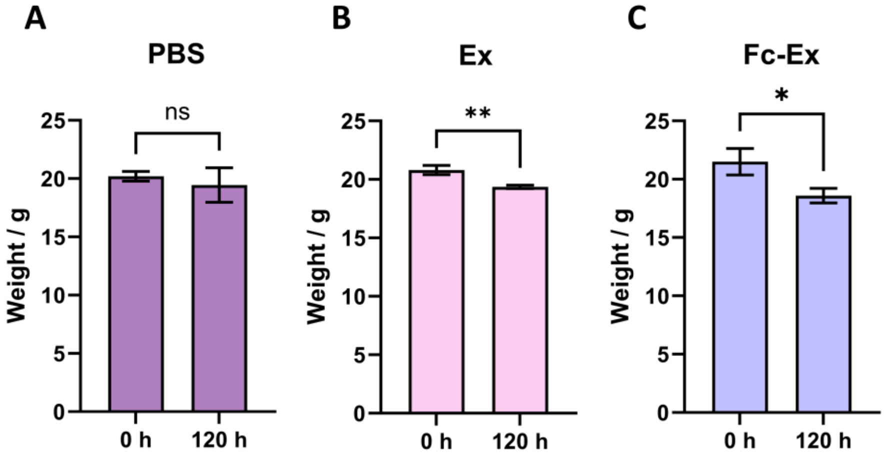

Figure S17: Comparison between mice weights at 0 hour and 120 hours, for mice dosed with A) PBS, B) Exenatide (Ex), or C) Fc-Exenatide (Fc-Ex). Data was analysed by an unpaired t-test (90% confidence) whereby * = $p < 0.1$, ** = $p < 0.05$, ns = non-significant, $p > 0.1$. Weight data shown is the mean of the weight of a minimum of 2 mice and error bars represent standard error of the mean.

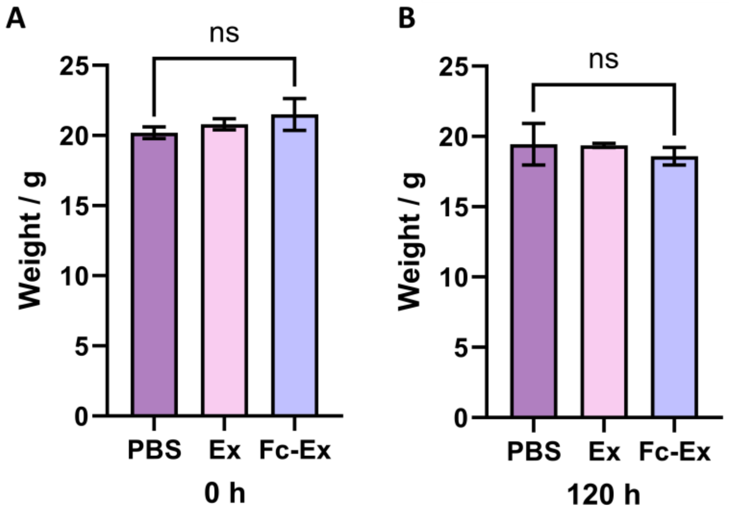

Figure S18: For mice dosed with either PBS, Exenatide (Ex), or Fc-Exenatide (Fc-Ex): A) comparison between mice weight at 0 hours and B) comparison between mice weights at 120 hours. Data was analysed by an ordinary one-way ANOVA whereby ns = non-significant, $p > 0.05$. Weight data shown is the mean of the weight of a minimum of 2 mice and error bars represent standard error of the mean.

Blood Plasma Sample Analysis of Exenatide

Table S3: Concentration of Exenatide detected in blood plasma sample in three different mice over 196 h time course.

Animal Barcode	Time	Exenetide conc (ng/mL)
M01221640	0	Not detected
M01221640	1	140
M01221640	12	Not detected
M01221640	48	Not detected
M01221640	72	Not detected
M01221640	96	Not detected
M01221640	120	Not detected
M01221640	196	Not detected
M01221638	0	Not detected
M01221638	1	76
M01221638	12	Not detected
M01221638	48	Not detected
M01221638	72	Not detected
M01221638	96	Not detected
M01221638	120	Not detected
M01221638	196	Not detected
M01221642	0	Not detected
M01221642	1	876
M01221642	12	Not detected
M01221642	48	Not detected
M01221642	72	Not detected
M01221642	96	Not detected
M01221642	120	Not detected
M01221642	196	Not detected

Table S4: Calibration lines generated for Exenatide concentration analysis.

Standard	Conc. (ng/mL)	Calibration line 1	Calibration line 2
1	10	11	9
2	25	23	26
3	50	58	50
4	100	100	103
5	500	427	560
6	1000	877	957
7	5000	5832	4333
8	10000	10931	9560

Detection of Exenatide in the mouse blood plasma was assessed at each time point by LC-MS/MS analysis. Generation of a calibration line determined the concentration of the Exenatide present. Exenatide was only detected at the first time point of 1 hour, after which no Exenatide was detected. This observation is unsurprising due to the short half-life of Exenatide in mice. It was thought that the large error bar (Figure SC) was due to the

suspected small amount of Exenatide in the blood plasma at 1 h making it difficult to carry out an accurate measurement.

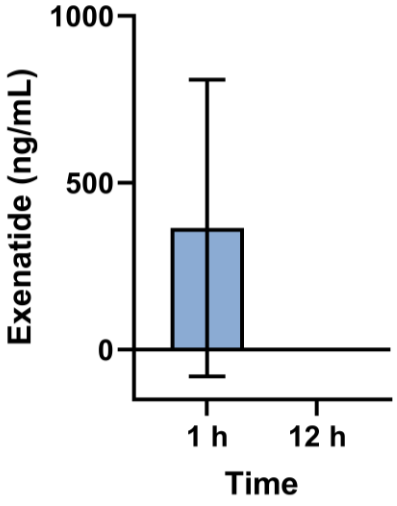

Figure S19: The concentration of Exenatide detected in mouse blood plasma 1 hour and 12 hours post dosing.

Blood Plasma Sample Analysis of Fc-Exenatide

Extraction from the mouse plasma was carried out using anti-Fc antibody immobilised on beads, and then tryptic digestion to generate peptides for LC-MS/MS analysis. The Fc protein backbone generates 3 peptides which can be detected. Exenatide generates 2 peptides to be detected, one from the middle of the sequence (mid-peptide) and one from the N-terminus (N-terminal peptide) (Figure SV).

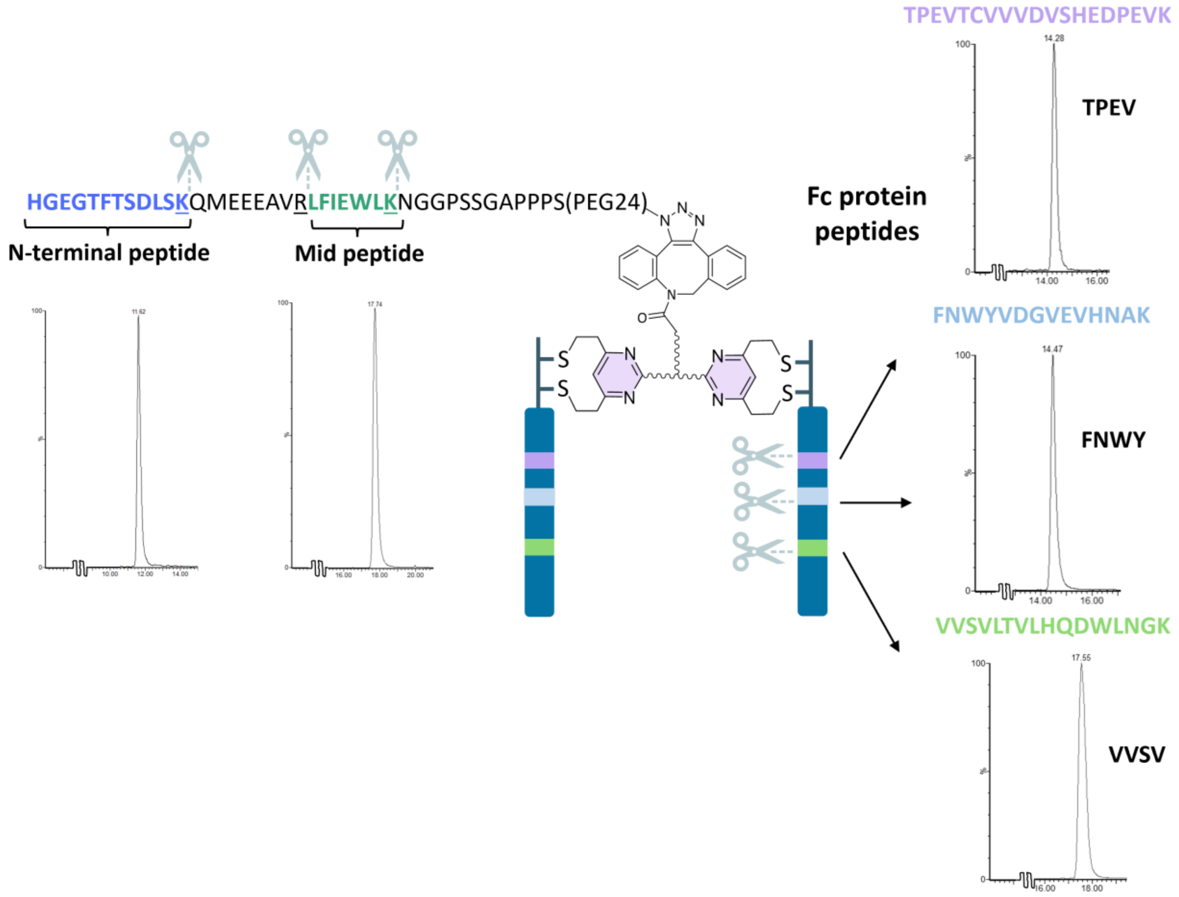

Figure S20: LC-MS/MS analysis of mouse plasma spiked with 5 µg/mL (80 nM) of Fc-exenatide. The sample was extracted using the anti-Fc beads, reduced, alkylated and tryptically digested to generate peptides from the Exenatide peptide and the Fc protein for LC-MS/MS analysis.

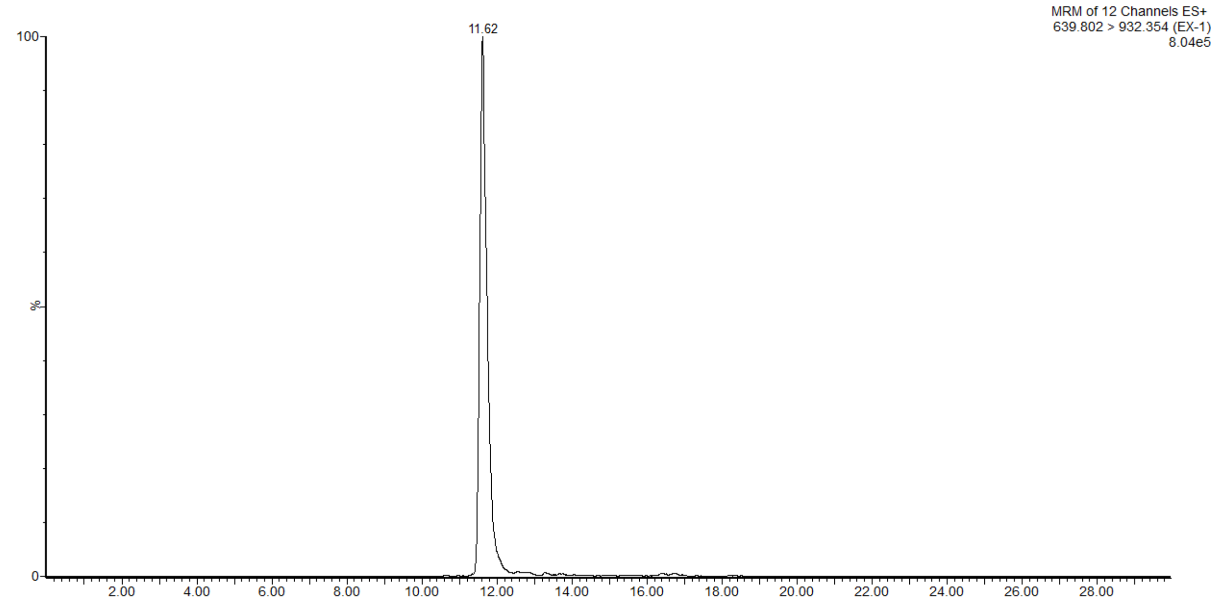

Figure S21: Extracted ion chromatogram of Exenatide N-terminal peptide: HGEGTFTSDLSK. From an LC-MS/MS analysis of mouse plasma spiked with 5 µg/mL (80 nM) of Fc-exenatide. The sample was extracted using the anti-Fc beads, reduced, alkylated and tryptically digested to generate peptides from the Exenatide peptide and the Fc protein for LC-MS/MS analysis.

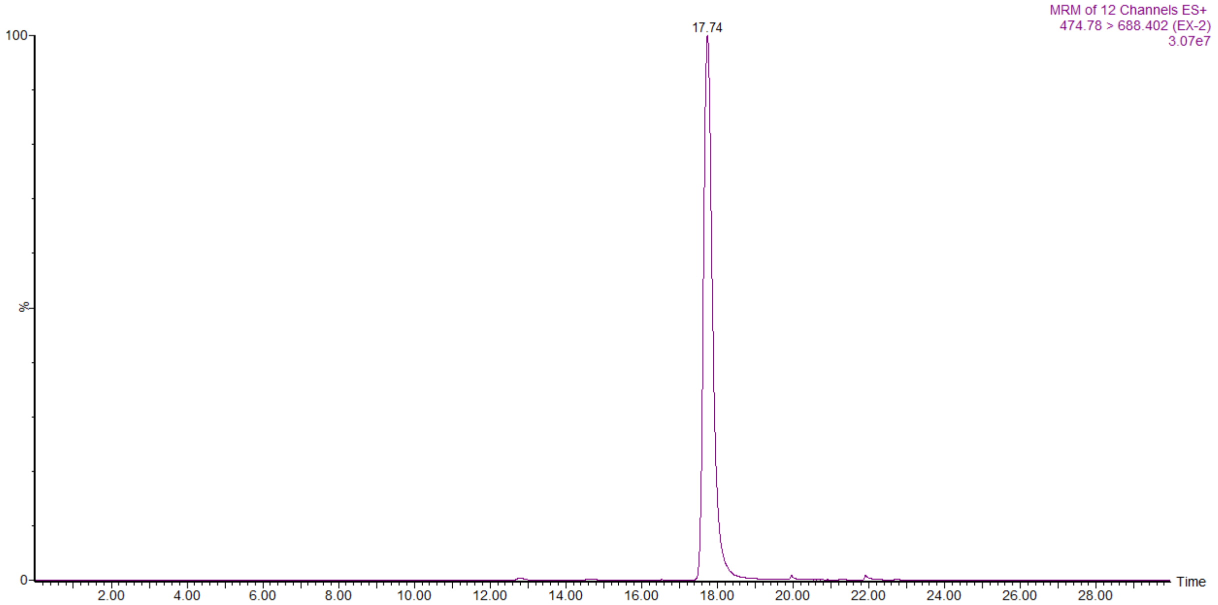

Figure S22: Extracted ion chromatogram of Exenatide mid peptide: LFIEWLK. From LC-MS/MS analysis of mouse plasma spiked with 5 µg/mL (80 nM) of Fc-exenatide. The sample was extracted using the anti-Fc beads, reduced, alkylated and tryptically digested to generate peptides from the Exenatide peptide and the Fc protein for LC-MS/MS analysis.

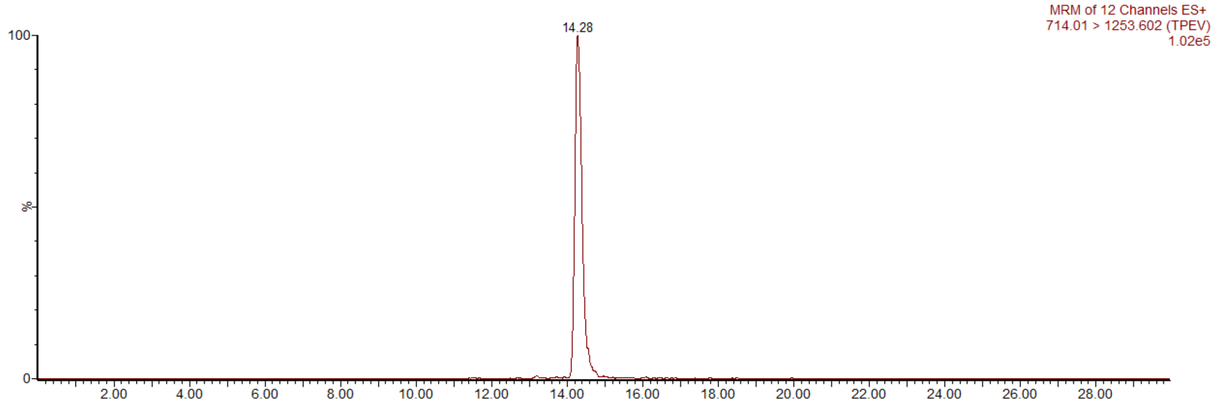

Figure S23: Extracted ion chromatogram of Fc protein back bone peptide: TPEVTCVVVDVSHEDPEVK. From LC-MS/MS analysis of mouse plasma spiked with 5 µg/mL (80 nM) of Fc-exenatide. The sample was extracted using the anti-Fc beads, reduced, alkylated and tryptically digested to generate peptides from the Exenatide peptide and the Fc protein for LC-MS/MS analysis.

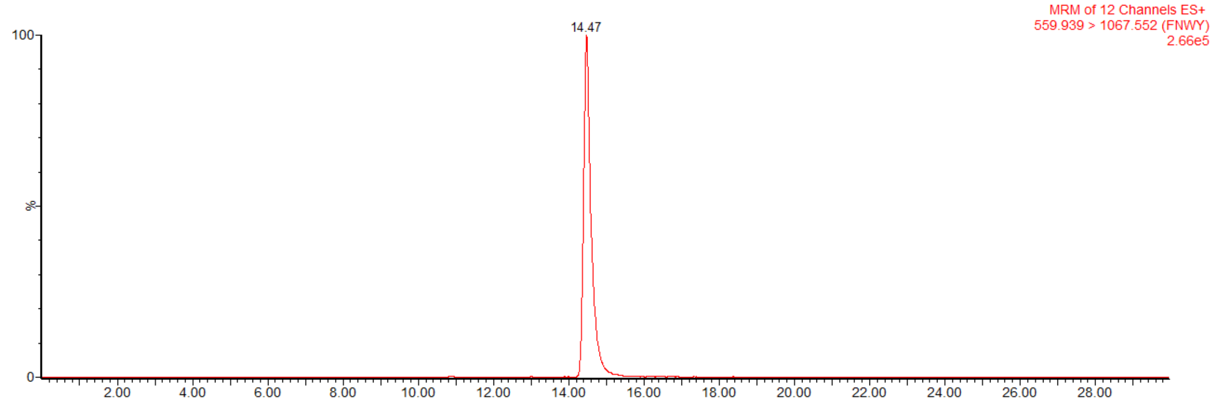

Figure S24: Extracted ion chromatogram of Fc protein back bone peptide: FNWYVDGVEVHNAK. From LC-MS/MS analysis of mouse plasma spiked with 5 µg/mL (80 nM) of Fc-exenatide. The sample was extracted using the anti-Fc beads, reduced, alkylated and tryptically digested to generate peptides from the Exenatide peptide and the Fc protein for LC-MS/MS analysis.

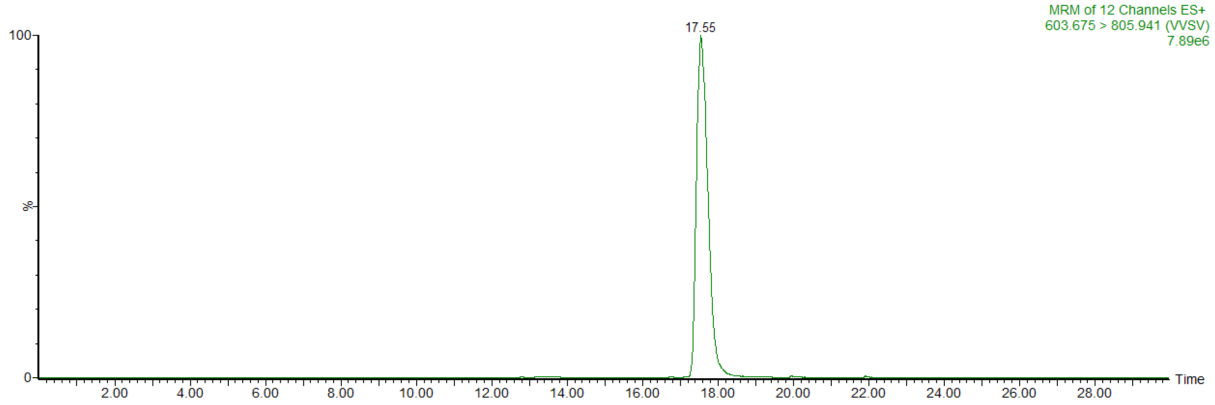

Figure S25: Extracted ion chromatogram of Fc protein back bone peptide: VVSVLTVLHQDWLNGK. From LC-MS/MS analysis of mouse plasma spiked with 5 µg/mL (80 nM) of Fc-exenatide. The sample was extracted using

the anti-Fc beads, reduced, alkylated and tryptically digested to generate peptides from the Exenatide peptide and the Fc protein for LC-MS/MS analysis.

The N-terminal peptide sequence is essential for Exenatide-receptor binding, therefore, if the N-terminal peptide is not intact the molecule is no longer active. Presence of the N-terminal peptide in addition to the middle-sequence peptide indicates that fully intact Exenatide is present.

Peptide peaks and calibration lines: N-terminus, mid peptide, Fc backbone

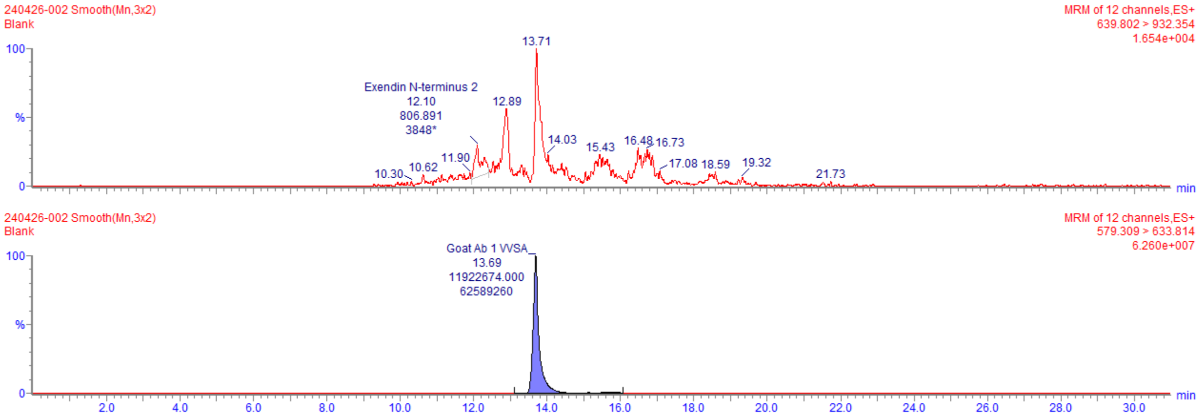

Figure S28: Blank plasma N-terminus

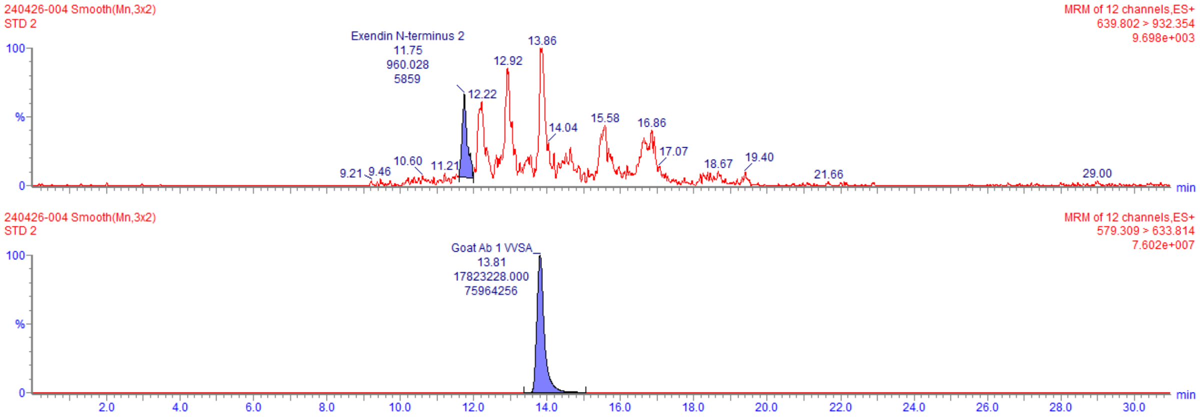

Figure S29: N-terminus 25 ng/mL

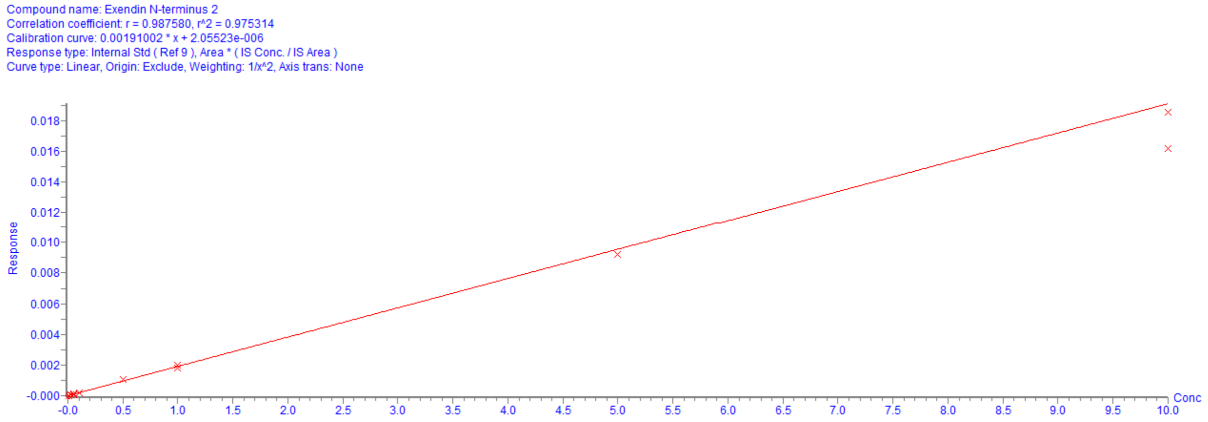

Figure S30: N-terminus calibration line

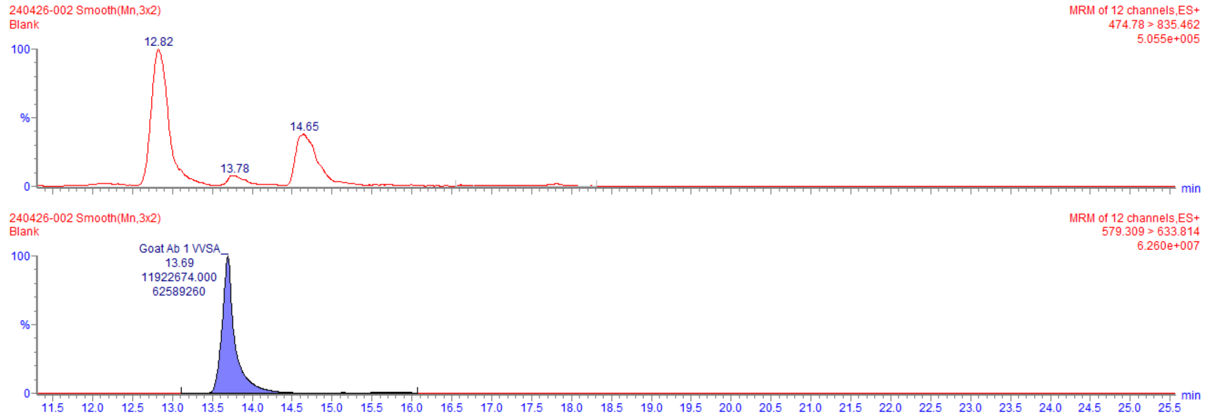

Figure S31: Blank plasma mid peptide

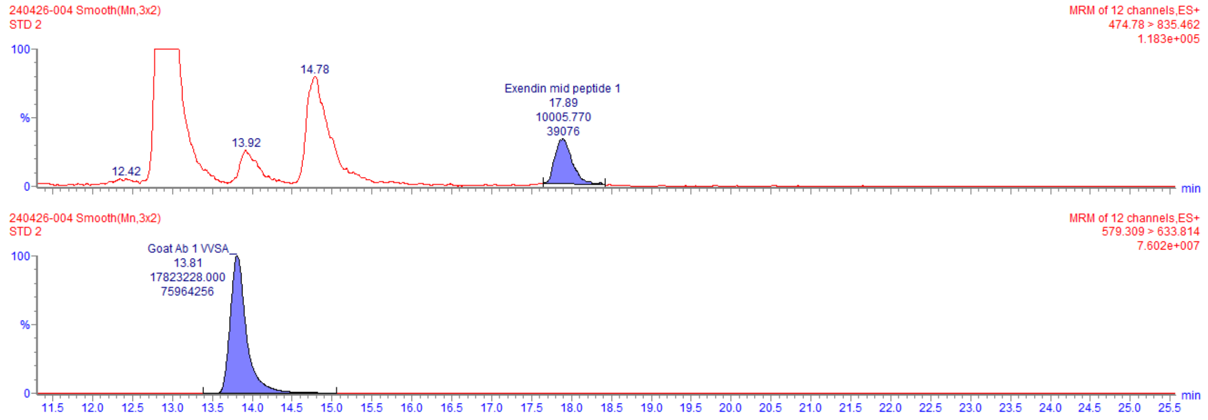

Figure S32: Mid peptide 25 ng/mL

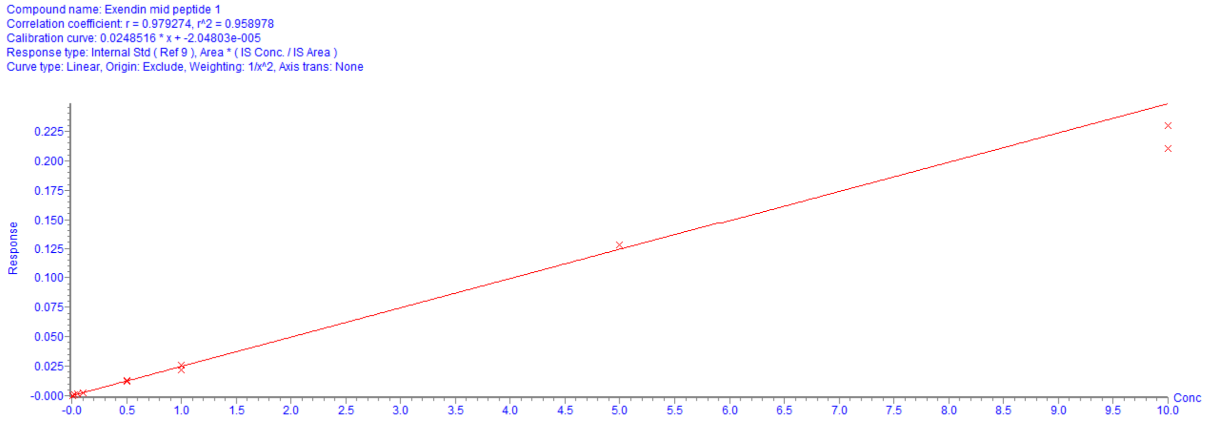

Figure S33: Mid peptide calibration line

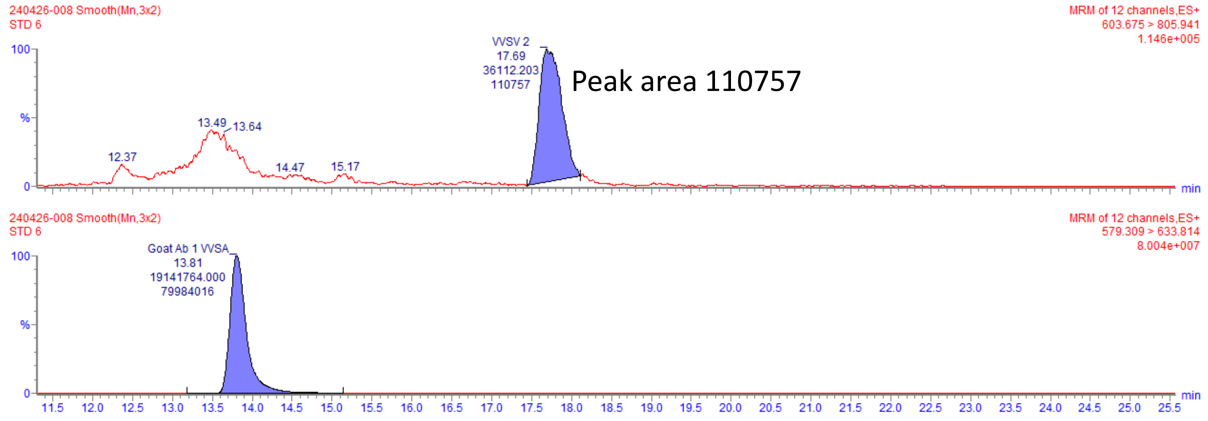

Figure S34: VSVV peptide 1 ug/mL

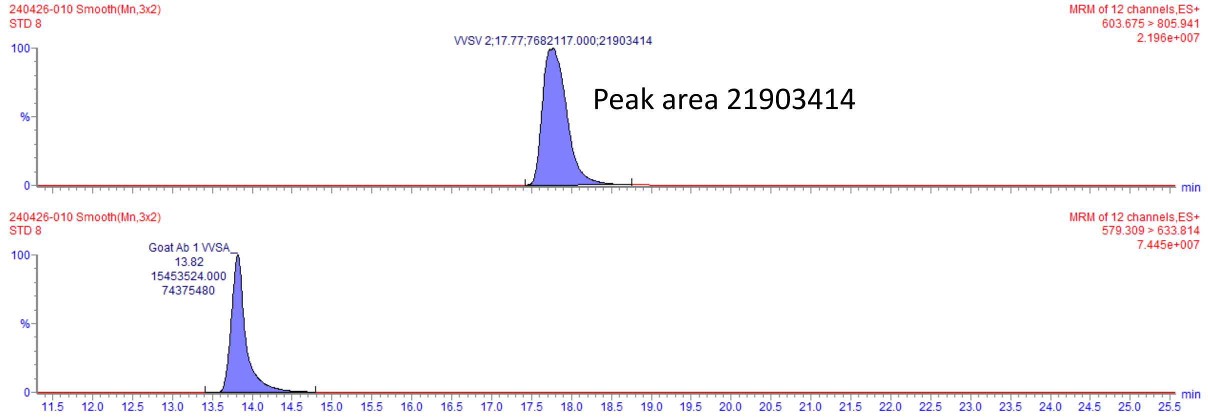

Figure S35: VSVV peptide 1 ug/mL?

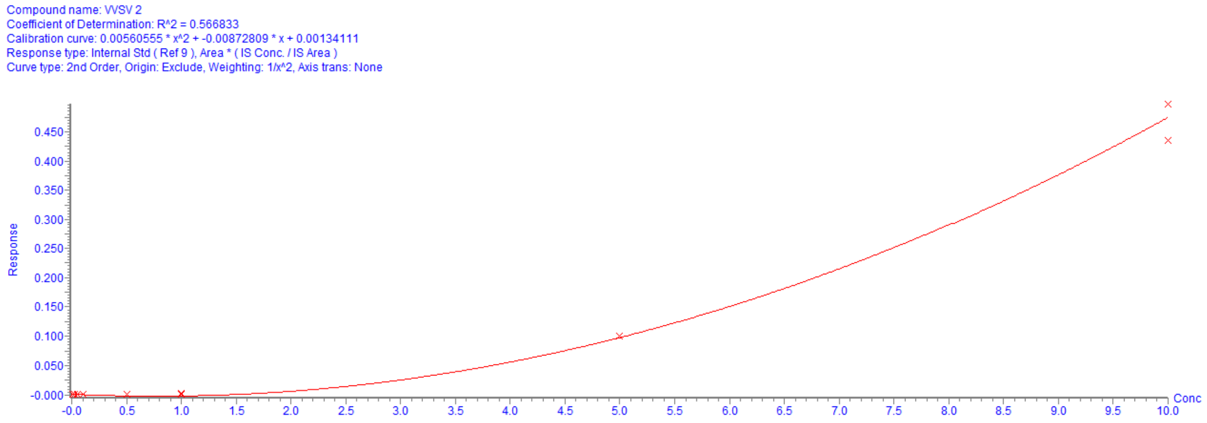

Figure S36: VSVV peptide calibration line (10ug/mL area: 21903414) and (1 ug/mL area: 110757) Note: All Fc peptide peak areas non-linear. All N-terminal peptides were linear.

Sample Analysis

Exenatide peptides:

Table S5: Concentration of N-terminal peptide detected in three different mice over 196 hours.

	Concentration (µg/mL)		
	N-terminus Peptide		
Time (h)	Mouse '637	Mouse '641	Mouse '643
0	0	0	0
1	10	10.2	11.2
12	6	5.8	6.1
48	3.8	2.7	2.6
72	1.7	1.7	2.2
96	1.1	1	0.9
120			
196	0.9	0.6	0.6

Table S6: Concentration of Mid peptide detected in three different mice over 196 hours.

	Concentration (µg/mL)		
	Mid peptide		
Time (h)	Mouse '637	Mouse '641	Mouse '643
0	0	0	0
1	9.9	10.5	11.5
12	7.7	7.5	8.3
48	7	5	4.9
72	3.5	3.2	3.6
96	2.6	2.2	2.1
120			
196	3.9	3.3	2.7

Fc protein backbone peptides:

Table S7: Concentration of VVSV Fc backbone peptide detected in three different mice over 196 hours.

	Peptide PEAK AREA only		
	VVSV Peptide		
Time (h)	Mouse '637	Mouse '641	Mouse '643
0	62	532	14
1	63699	130742	138773
12	95232	92800	84160
48	77647	82981	86229
72	56747	62176	27574
96	46491	46081	54443
120			61
196	1528	726	680

Table S8: Concentration of FNWY Fc backbone peptide detected in three different mice over 196 hours.

	Peptide PEAK AREA only		
	FNWY Peptide		
Time (h)	Mouse '637	Mouse '641	Mouse '643
0	176	1008	524
1	187165	409058	417652
12	281918	276296	261787
48	258958	241767	253080
72	203553	187690	82314
96	152155	159782	168295
120		1901	1746
196	16030	9559	7969

Table S9: Concentration of TPEV Fc backbone peptide detected in three different mice over 196 hours.

	Peptide PEAK AREA only		
	TPEV Peptide		
Time (h)	Mouse '637	Mouse '641	Mouse '643
0	561	572	249
1	20884	70605	67294
12	42851	44209	38850
48	40517	39529	41663
72	29165	28974	12292
96	23338	25814	25363
120		32	121
196	2121	2462	2486

Note: unknown issue with 120 hour analysis resulted in missing data points for 120 hour samples.

Calibration lines were generated using the peak area ratio of specific peptides to the goat antibody peptide. This was successful for the Exenetide derived peptides, however the calibration samples for the Fc protein backbone peptides unexplainably failed to generate a linear fit. Therefore, concentrations of Fc derived peptides in mouse samples could not be assigned based on the calibration standards and only peak area values have been reported for Fc peptides, therefore these are not directly comparable. Since the Exenatide peptide concentrations were successfully obtained, half-lives can be generated for these peptides and thus a conclusion of whether a significant extension in half-life was achieved can be determined.

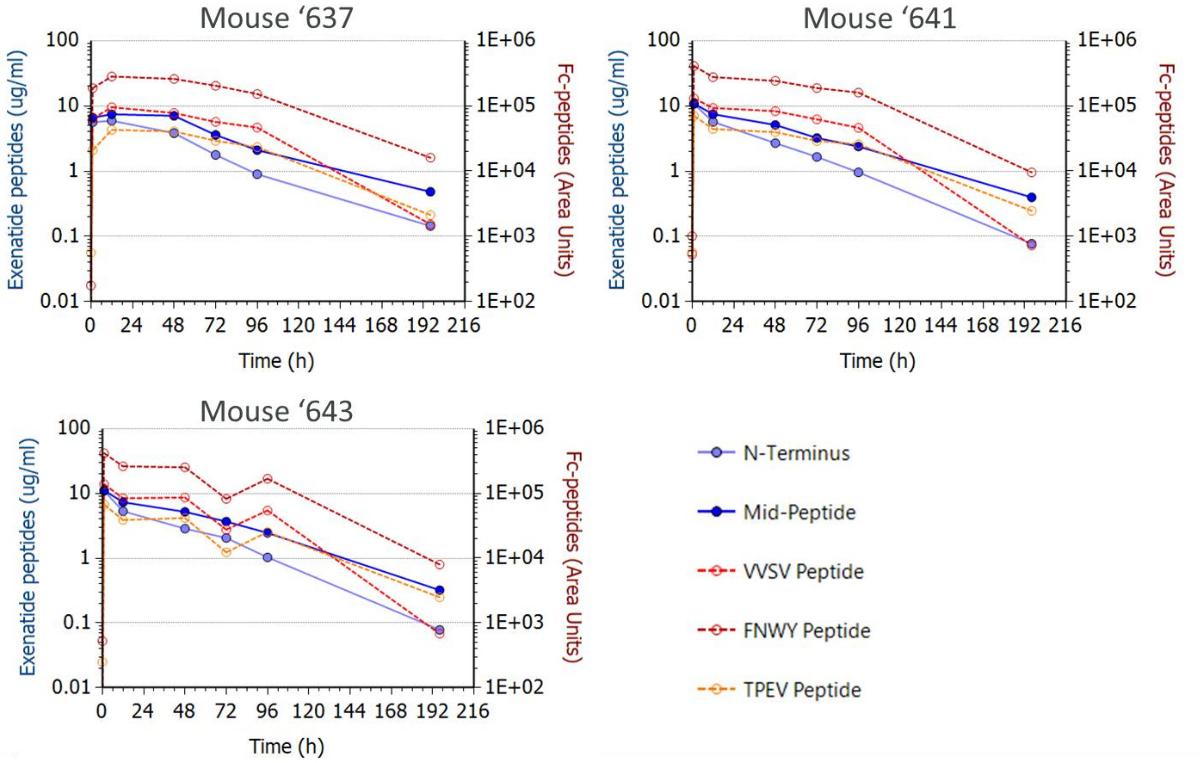

Figure S37: The PK profiles of each species after exclusion of 120 h timepoint in blood plasma measured from 1 hour to 196 hours.

Detection of the Fc-Exenatide conjugate was achieved in all the samples except the 120-hour samples where analysis failed for all species. It is possible that the sample collected at the 120-hour timepoint was too little for extraction.

LC-MS/MS analysis of the samples revealed that a higher concentration of mid peptide vs. N-terminal peptide was present, thus indicating that cleavage of the N-terminal peptide had occurred. It was likely that cleavage of the final two N-terminal amino acids of Exenatide occurred as it is typical of GLP1 peptide analogues to be cleaved at this position by the dipeptidyl peptidase-4 (DPP4) enzyme. Further analysis searching for degradation species confirmed that cleavage had occurred between glycine and glutamic acid as predicted (Figure SX).

LC-MS/MS Analysis of Mouse ‘637 samples to understand cleavage of Exenatide peptide

>sp|Q9R0Q5|FCEX_Human PYY Fc-Exenatide OS=Homo sapiens GN=MPARK PE=3 SV=1

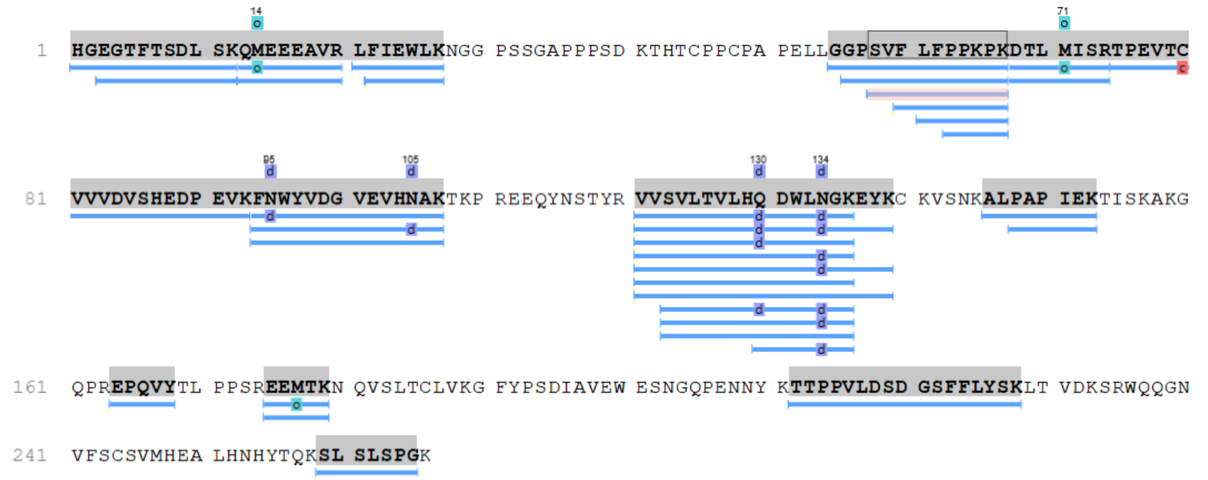

Time	HGEGTFTSDLSK	EGTFTSDLSK	LFIEWLK	FIEWLK	VVSVLTVLHQDWLNGK
0					
1	1.27E+06	1.44E+04	2.15E+06		2.59E+05
12	5.66E+05	9.74E+04	1.26E+06	1.56E+04	2.49E+05
48	5.58E+05	2.78E+05	1.93E+06	1.91E+04	2.11E+05
72	2.45E+05	1.73E+05	1.02E+06	1.06E+04	1.59E+05
96	1.28E+05	9.84E+04	7.29E+05		2.21E+05
196	7.41E+03	1.61E+04	1.12E+05		1.90E+04

Cleavage   Cleavage (v low level)

HGEGTFTSDLSKQMEEEAVRLFIEWLKNGGPSSGAPPPS......DKTHTCPPCPAPELLGGPSVFLFPPKPK
DTLMISRTPEVTCVVVDVSHEDPEVKFNWYVDGVEVHNAKTKPREEQYNSTYRVVSVLTVLHQDWLNGK
EYKCKVSNKALPAPIEKTISKAKGQPREPQVYTLPPSREEMTKNQVSLTCLVKGFYPSDIAVEWESNGQPE
NNYKTTPPVLDSDGSFFLYSKLTVDKSRWQQGNVFSCSVMHEALHNHYTQKSLSLSPGK

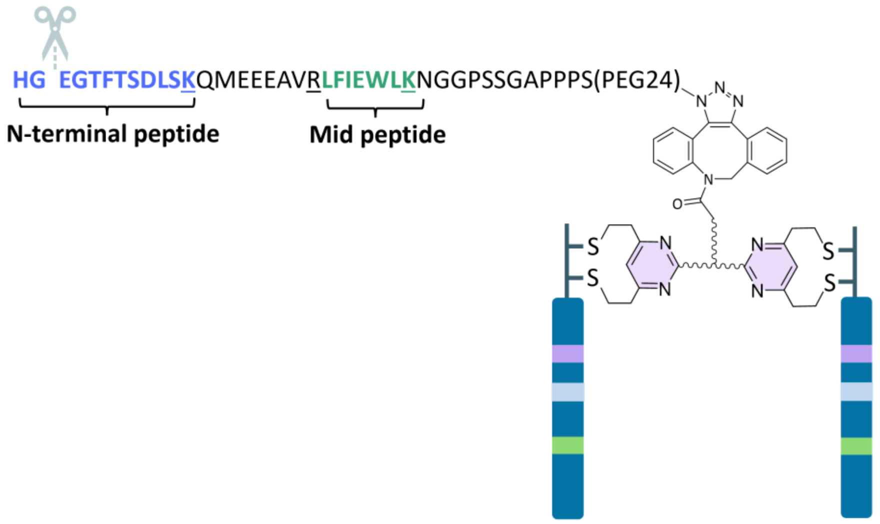

Figure S38: Cleavage of N-terminal peptide of Exenatide in Fc-Exenatide.

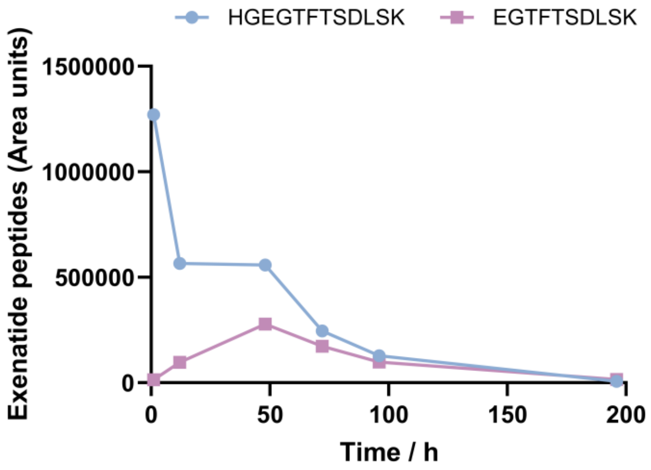

Figure S39: Fc-Exenatide degradation analysis. Result of LC-MS/Ms analysis of animal BL245 samples 0, 1, 12, 48, 72, 96 and 196 hour samples.

Table S10: Peptide exposure using a non-compartment approach (linear up-log down trapezoidal method) following iv bolus injections. Note: Mouse '637 (BL245) is simplified to Mouse 1; Mouse '641 is simplified to Mouse 2 (BL246); and Mouse '643 (BR246) is simplified to Mouse 3. *Last 3 datapoints used in calculation. **Sum of back-extrapolated (t1 to t0) and extrapolated area (tlast to infinity) is <10%.

Mouse	Terminal Half-life* (h)		AUC** (ug x h / mL)		Cmax (ug/mL)		Tmax (h)	
	N-terminal peptide	Mid peptide	N-terminal peptide	Mid peptide	N-terminal peptide	Mid peptide	N-terminal peptide	Mid peptide
**1**	35.2	44.0	385	673	5.92	7.43	12	12
**2**	27.9	40.3	362	632	10.7	10.8	1	1
**3**	26.5	34.9	373	632	10.9	11.1	1	1

Mass Spectrometry Traces

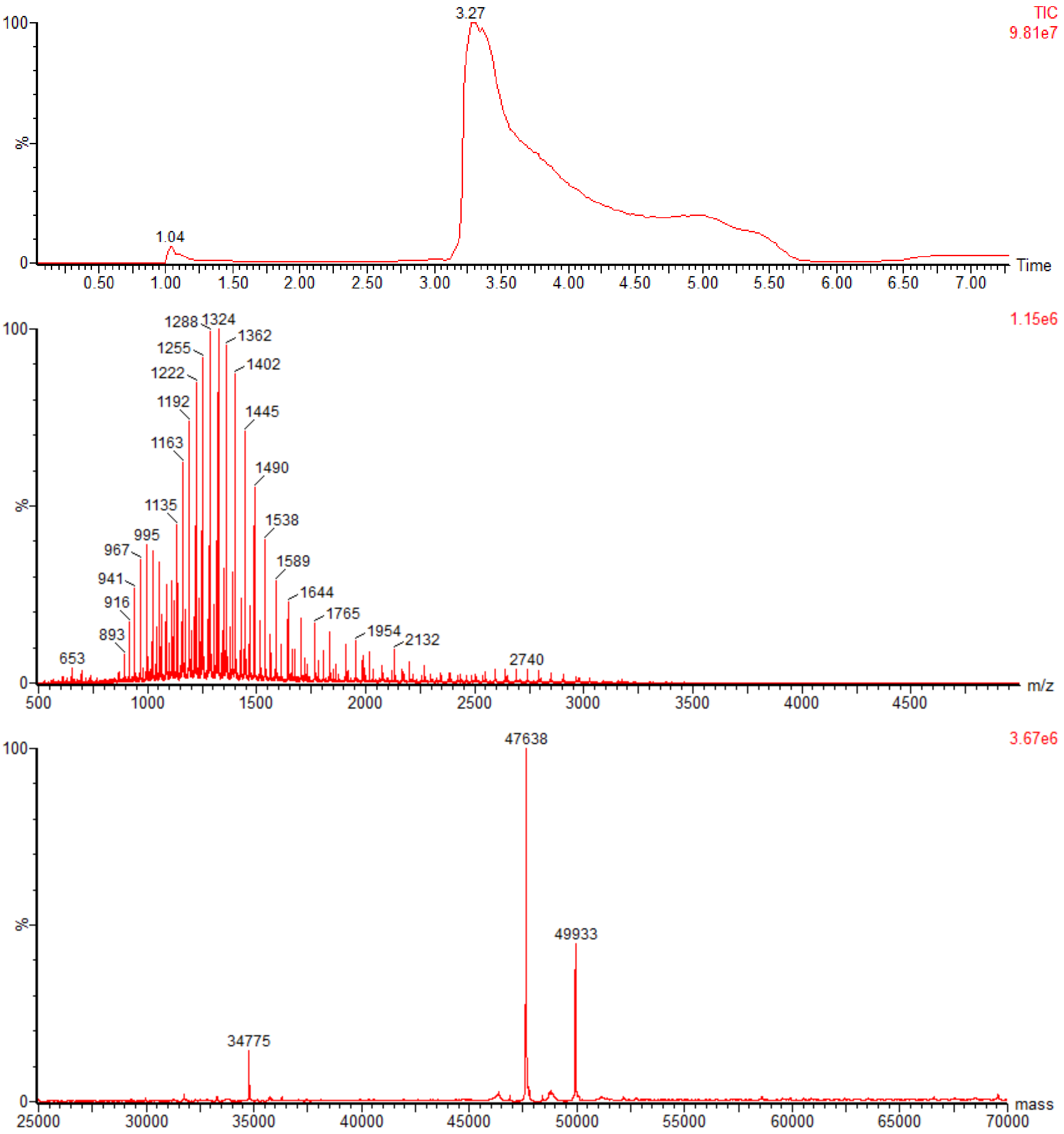

Figure S40: TIC trace (top), non-deconvoluted (middle) and deconvoluted (bottom) MS of the Trastuzumab digestion at 12 hours.

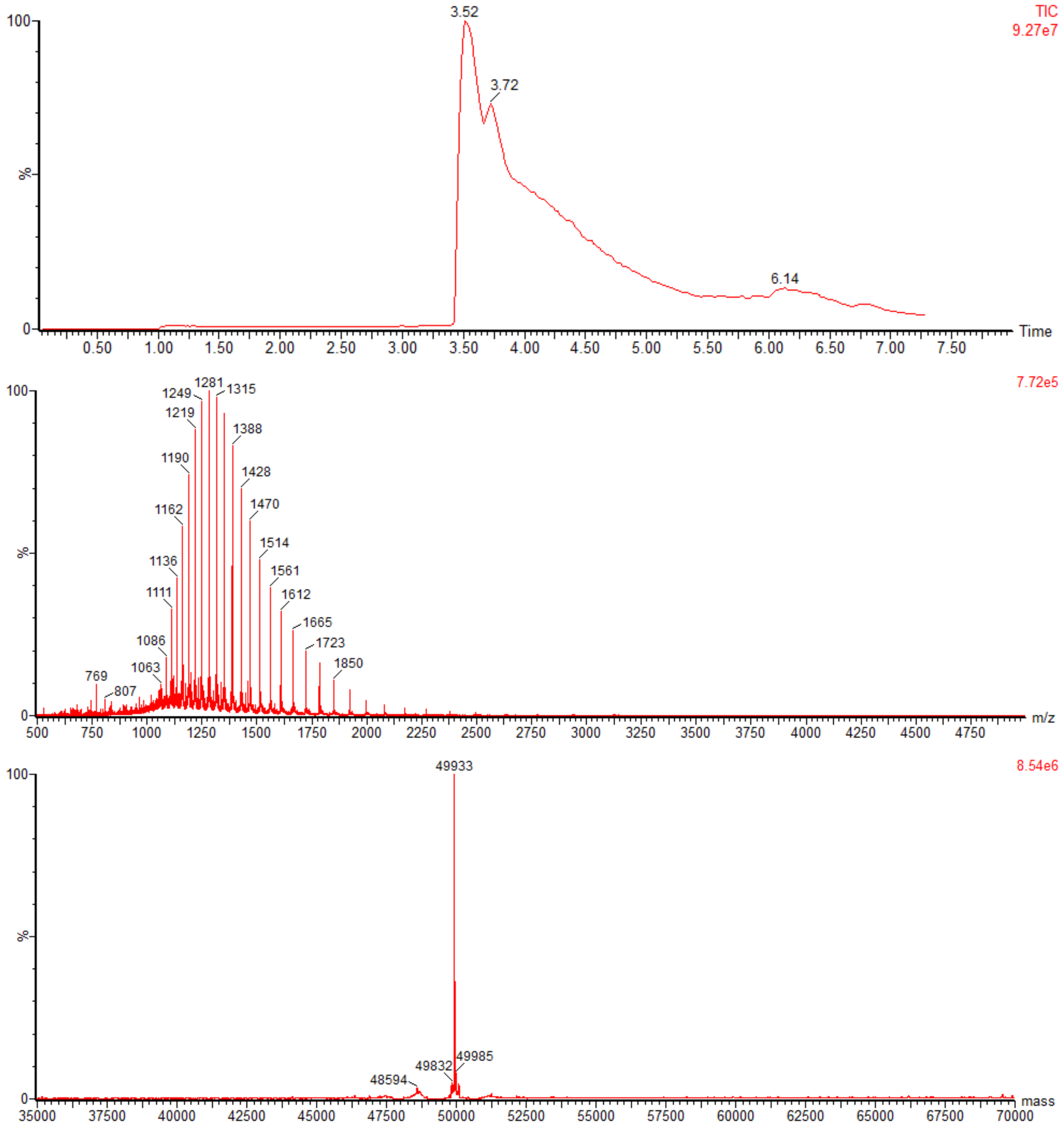

Figure S41: TIC trace (top), non-deconvoluted (middle) and deconvoluted (bottom) MS of purified Fc protein from digestion, expected mass: 49923 Da; found: 49933 Da.

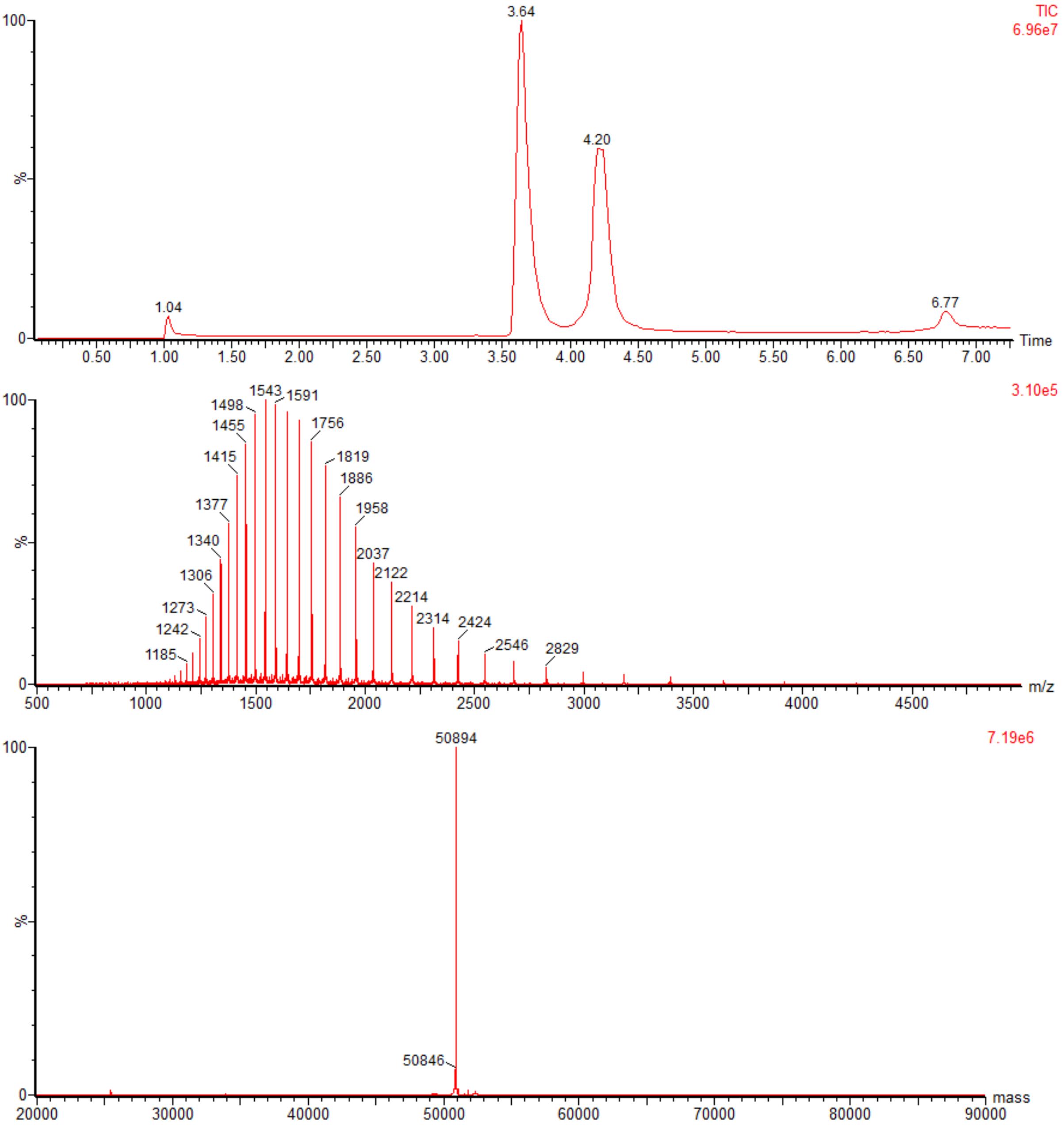

Figure S42: TIC trace (top), non-deconvoluted (middle) and deconvoluted (bottom) MS of recombinant Fc protein, expected mass: 50891 Da; found: 50894 Da.

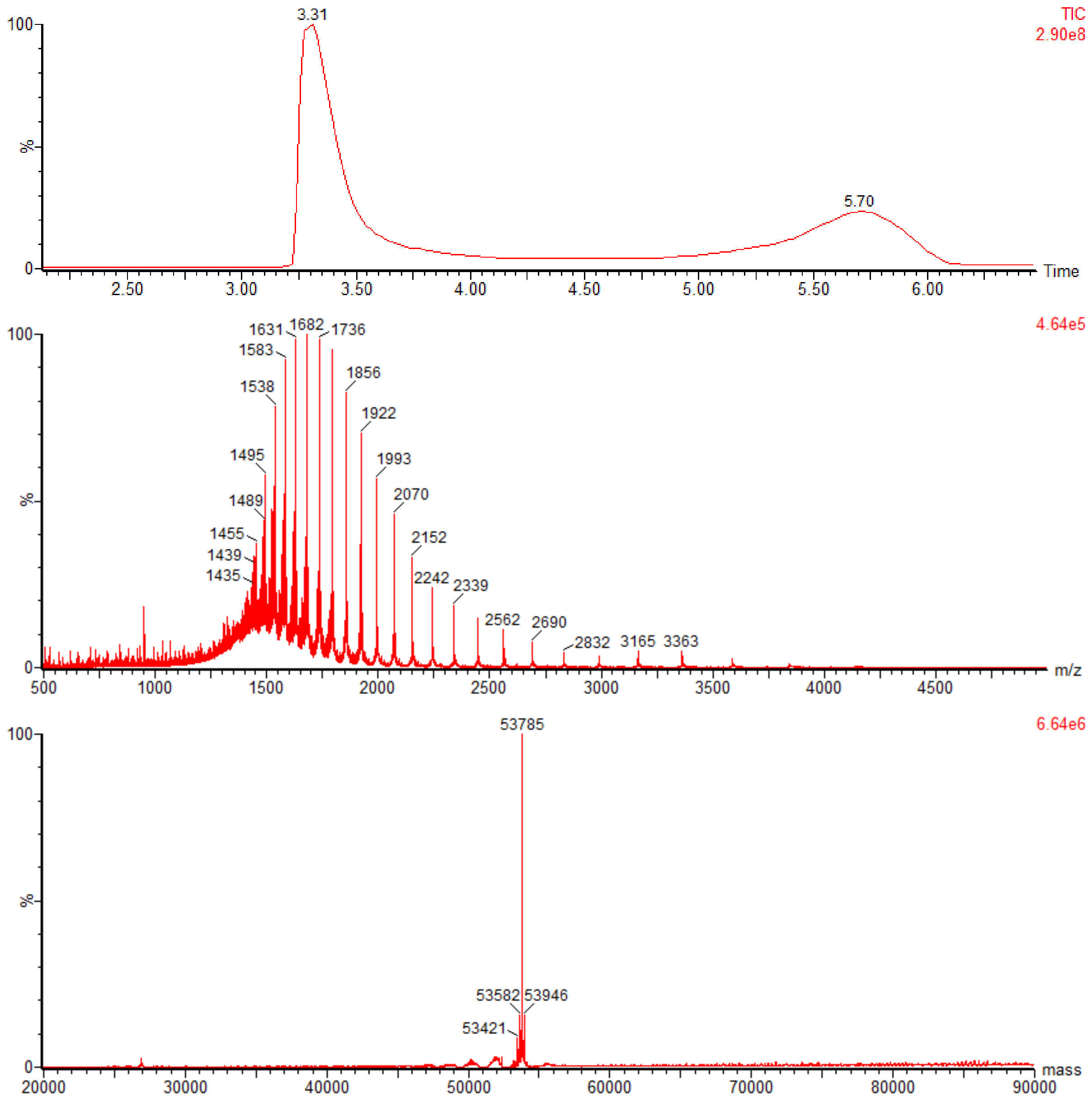

Figure S43: TIC trace (top), non-deconvoluted (middle) and deconvoluted (bottom) MS of recombinant Fc protein with glycosides present, major species found: 53785 Da.

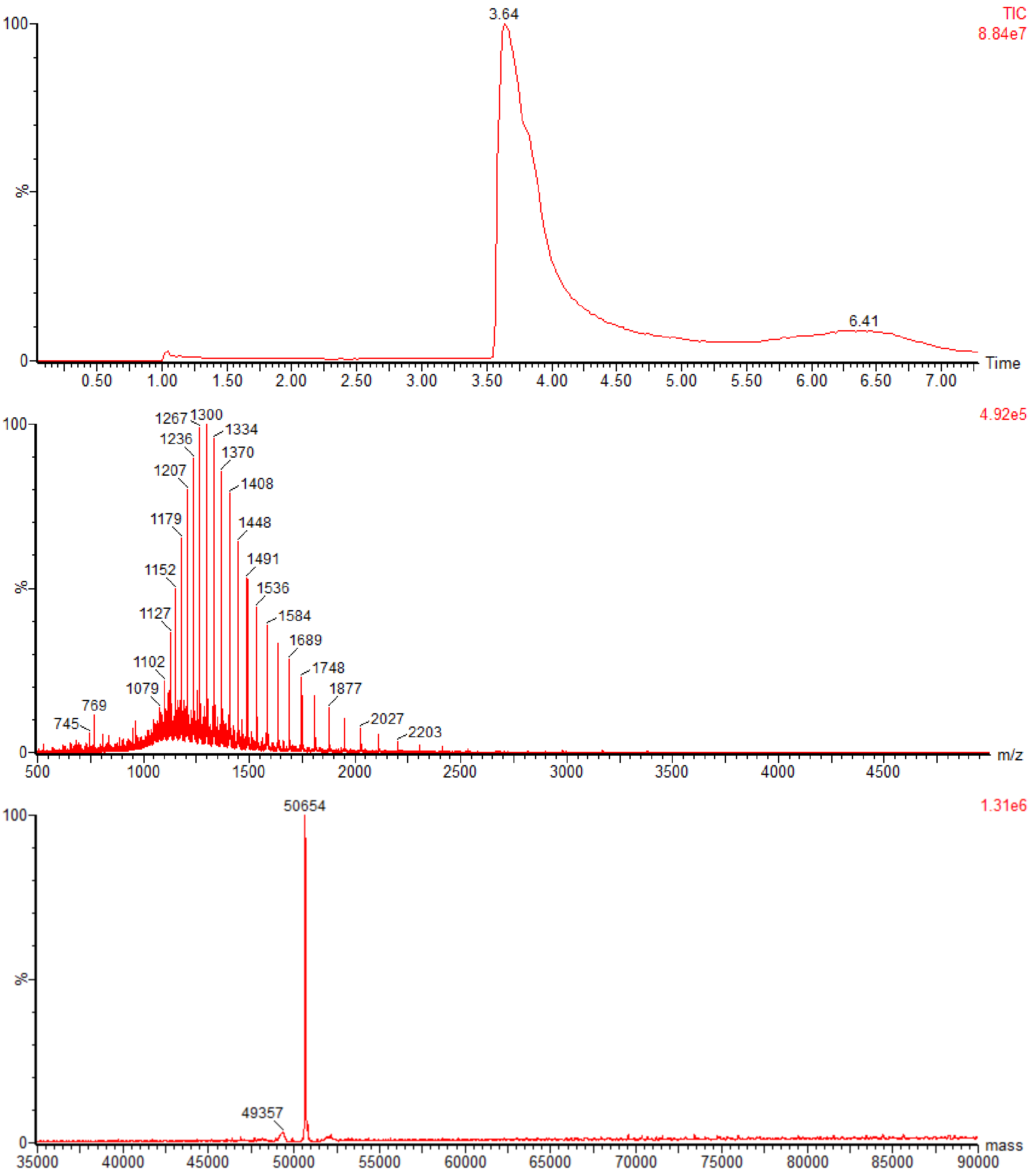

Figure S44: TIC trace (top), non-deconvoluted (middle) and deconvoluted (bottom) MS of bioconjugation reaction mixture after 5 equivalents of **BisDVP 1** to form **6**, 1 h, 37 °C, expected mass: 50649 Da; found: 50654 Da.

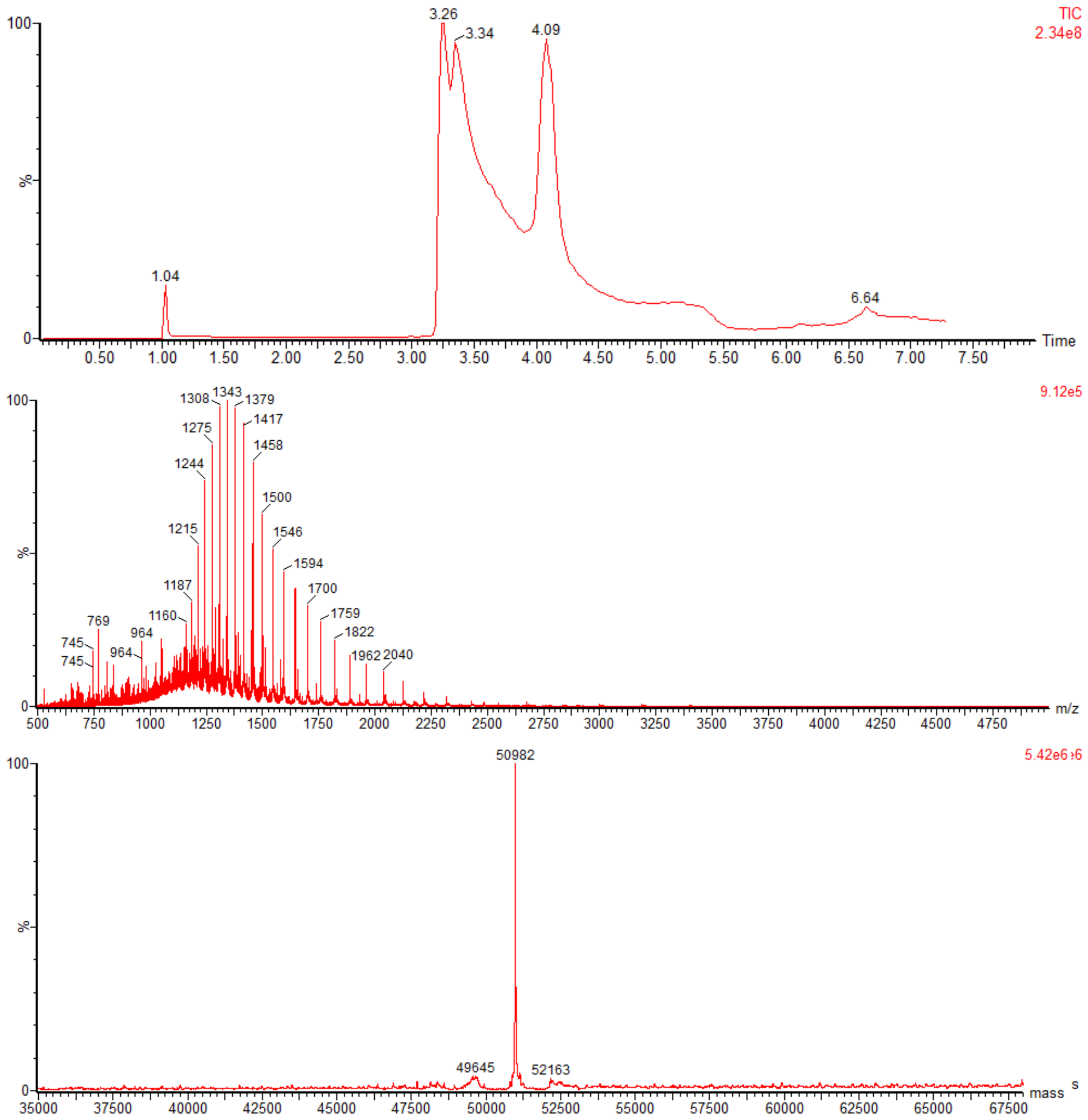

Figure S45: Bioconjugation of trastuzumab Fc with **BisDVP 2** to form **7.** TIC trace (top), non-deconvoluted (middle) and deconvoluted (bottom) MS, expected mass: 50977 Da; found: 50982 Da.

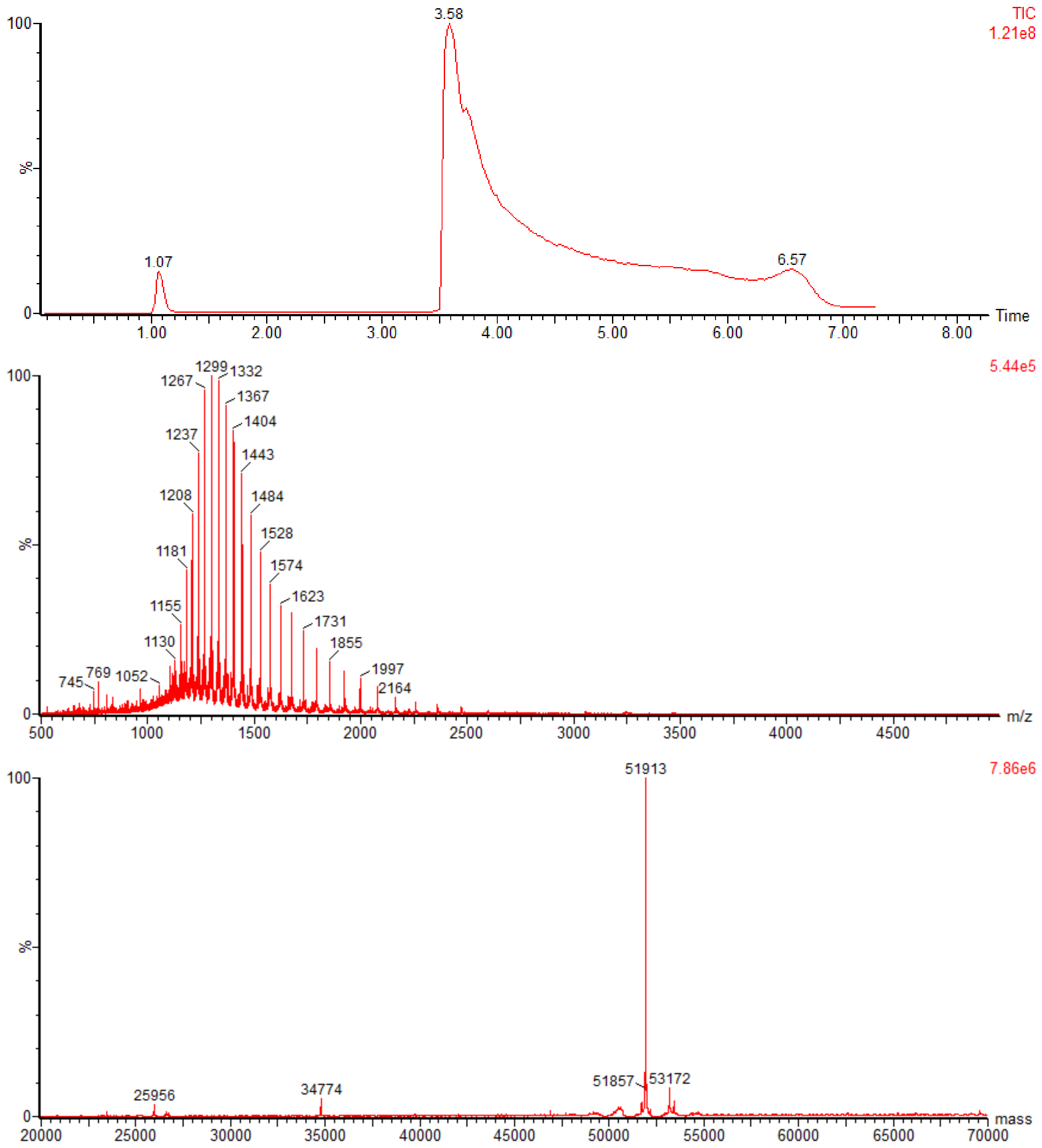

Figure S46: Analysis of Fc-BisDVP-DBCO **8.** TIC trace (top), non-deconvoluted (middle) and deconvoluted (bottom) MS, expected 51900 Da and observed 51913 Da.

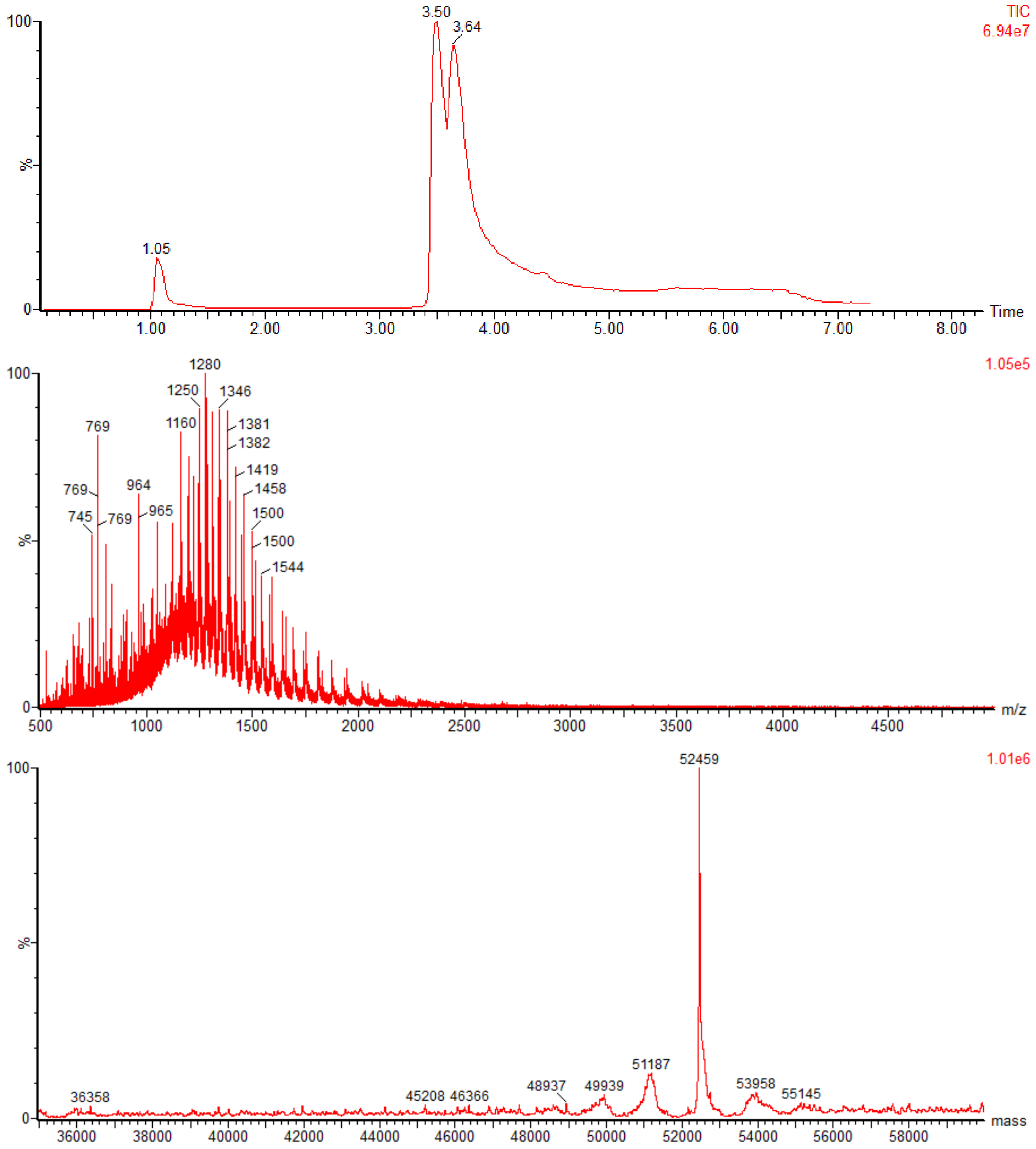

Figure S47: CuAAC of Fc-BisDVP-alkyne **6** with afamelanotide-N_3 to form Fc-Afamelanotide **10**. TIC trace (top), non-deconvoluted (middle) and deconvoluted (bottom) MS, expected 52449 Da and observed and 52453 Da.

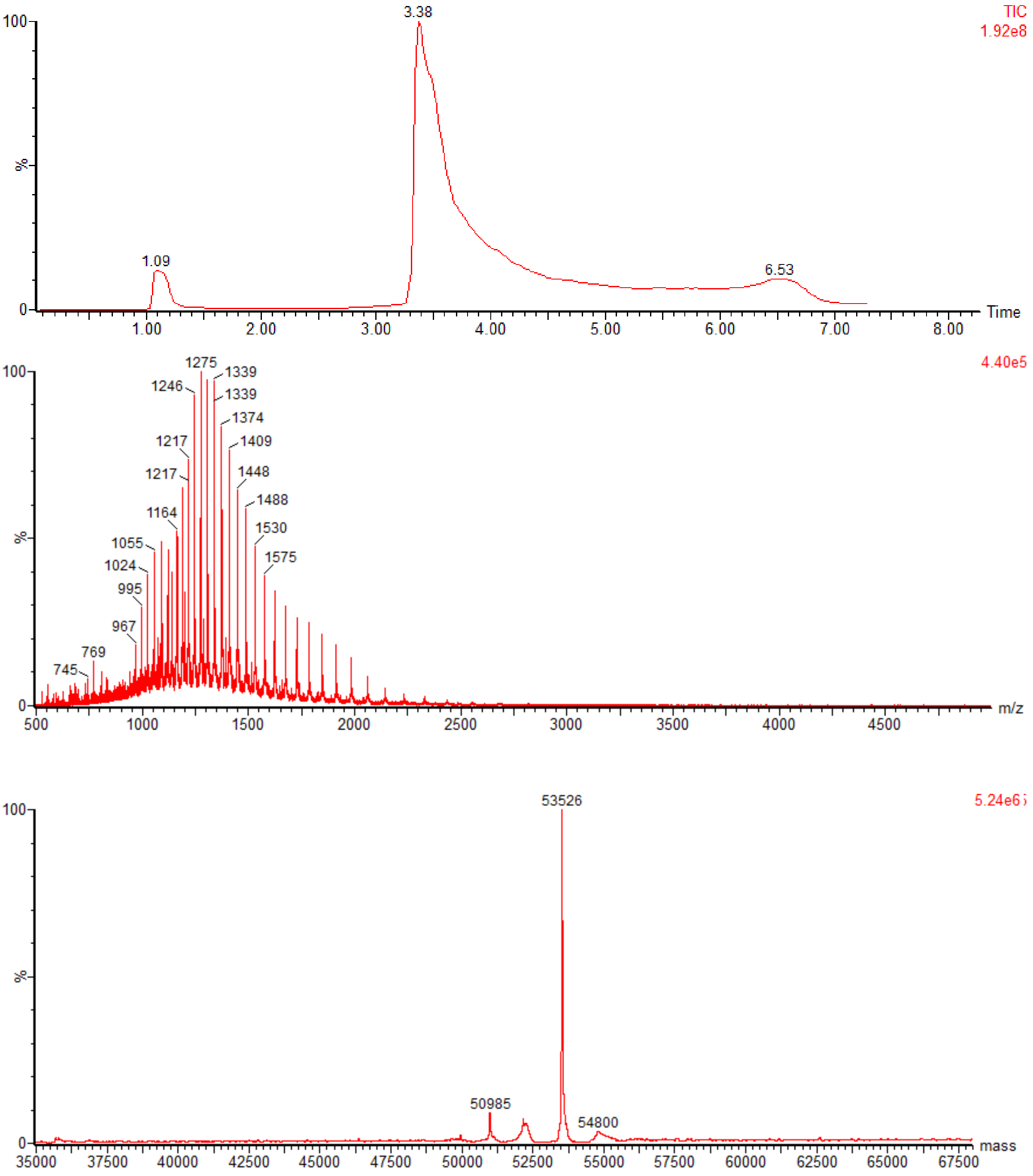

Figure S48: CuAAC of Fc-BisDVP-alkyne-azide with afamelanotide-DBCO to form **11.** TIC trace (top), non-deconvoluted (middle) and deconvoluted (bottom) MS, expected 53505 Da and observed 53526 Da.

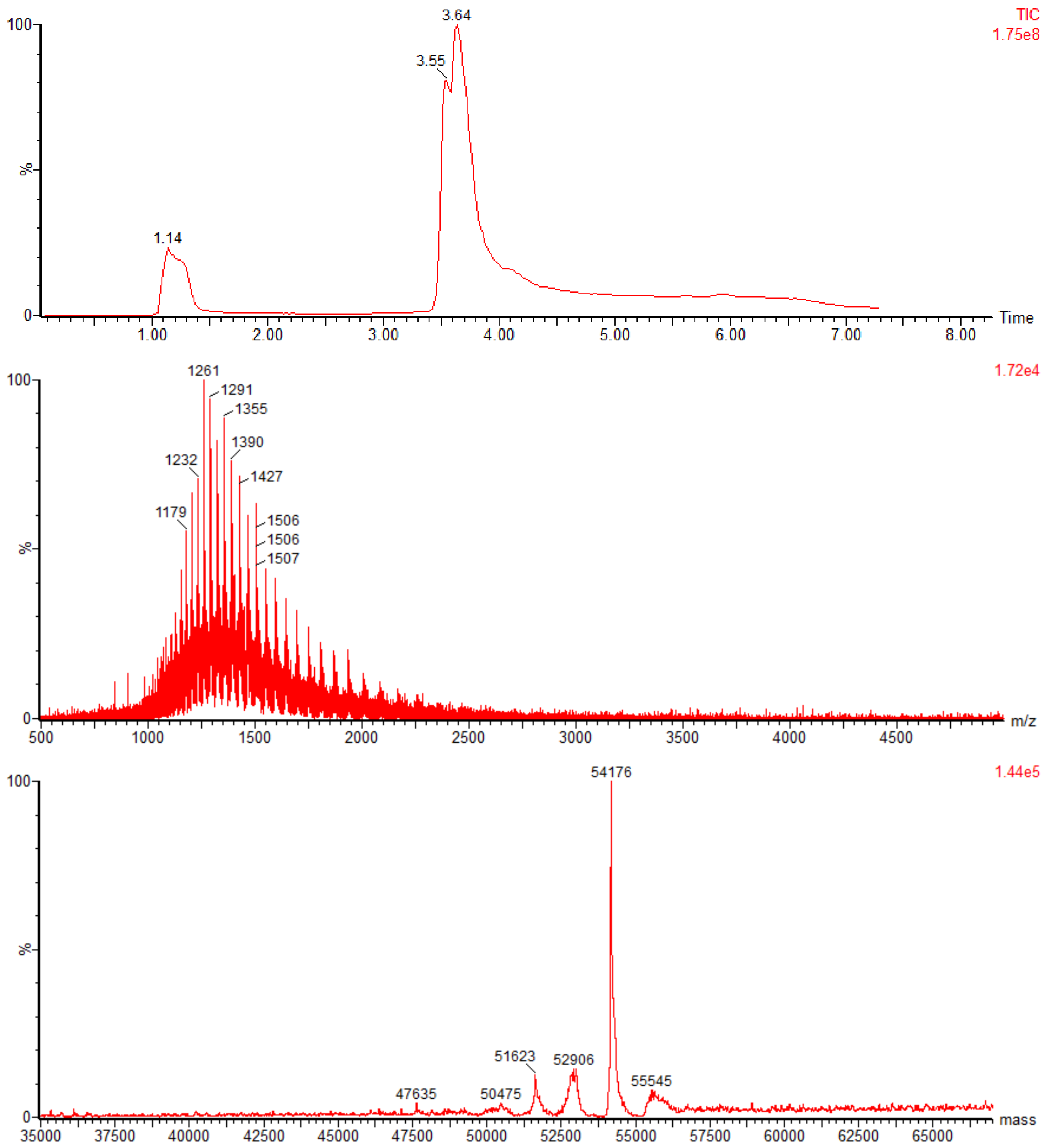

Figure S49: CuAAC of Fc-Afamelanotide-alkyne with AF488-azide to form **12**. TIC trace (top), non-deconvoluted (middle) and deconvoluted (bottom) MS, expected 54178 Da and observed 54176 Da.

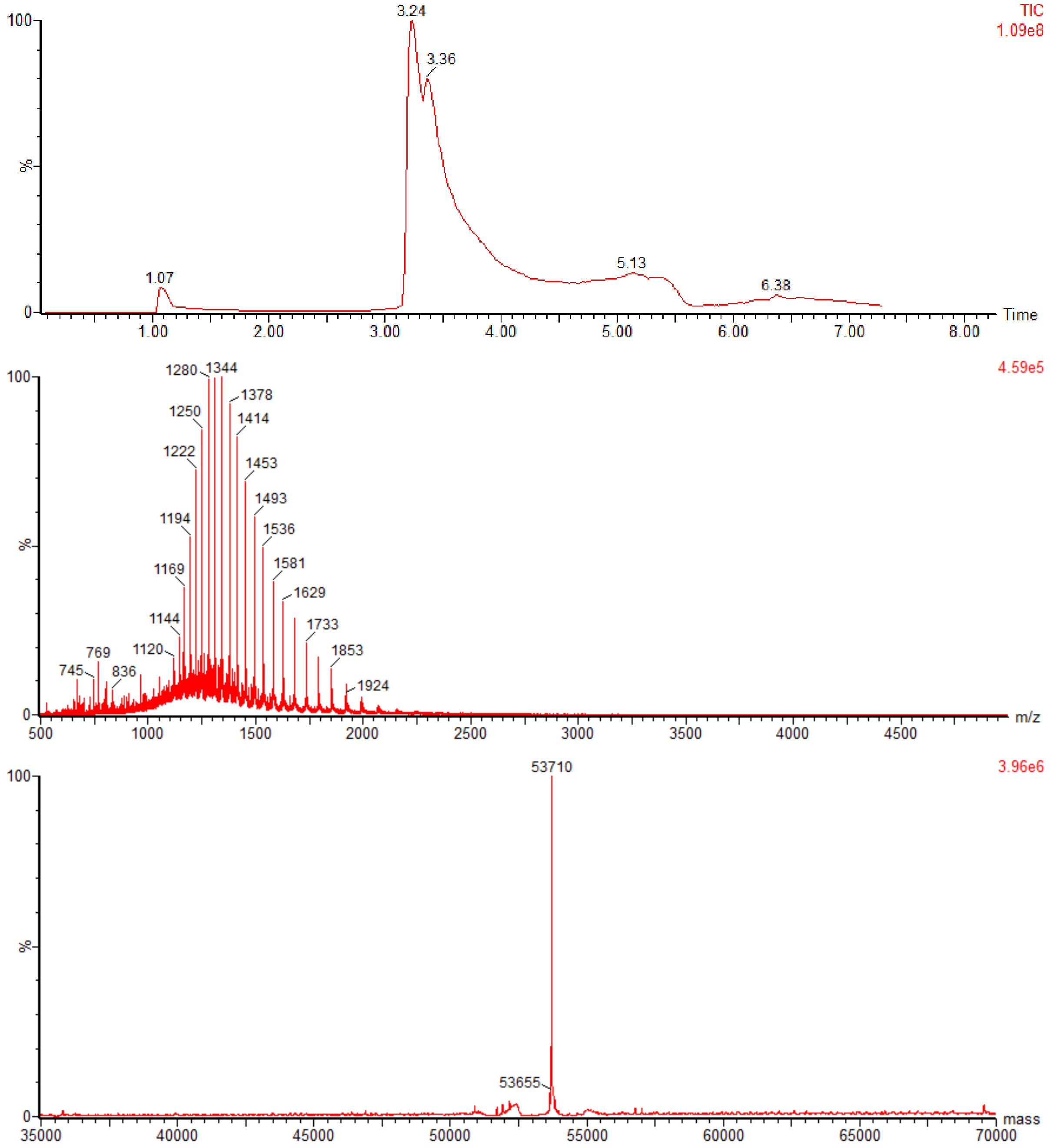

Figure S50: Fc-Afamelanotide (recombinant Fc) **13.** TIC trace (top), non-deconvoluted (middle) and deconvoluted (bottom) MS, expected 53709 Da and observed 53707 Da.

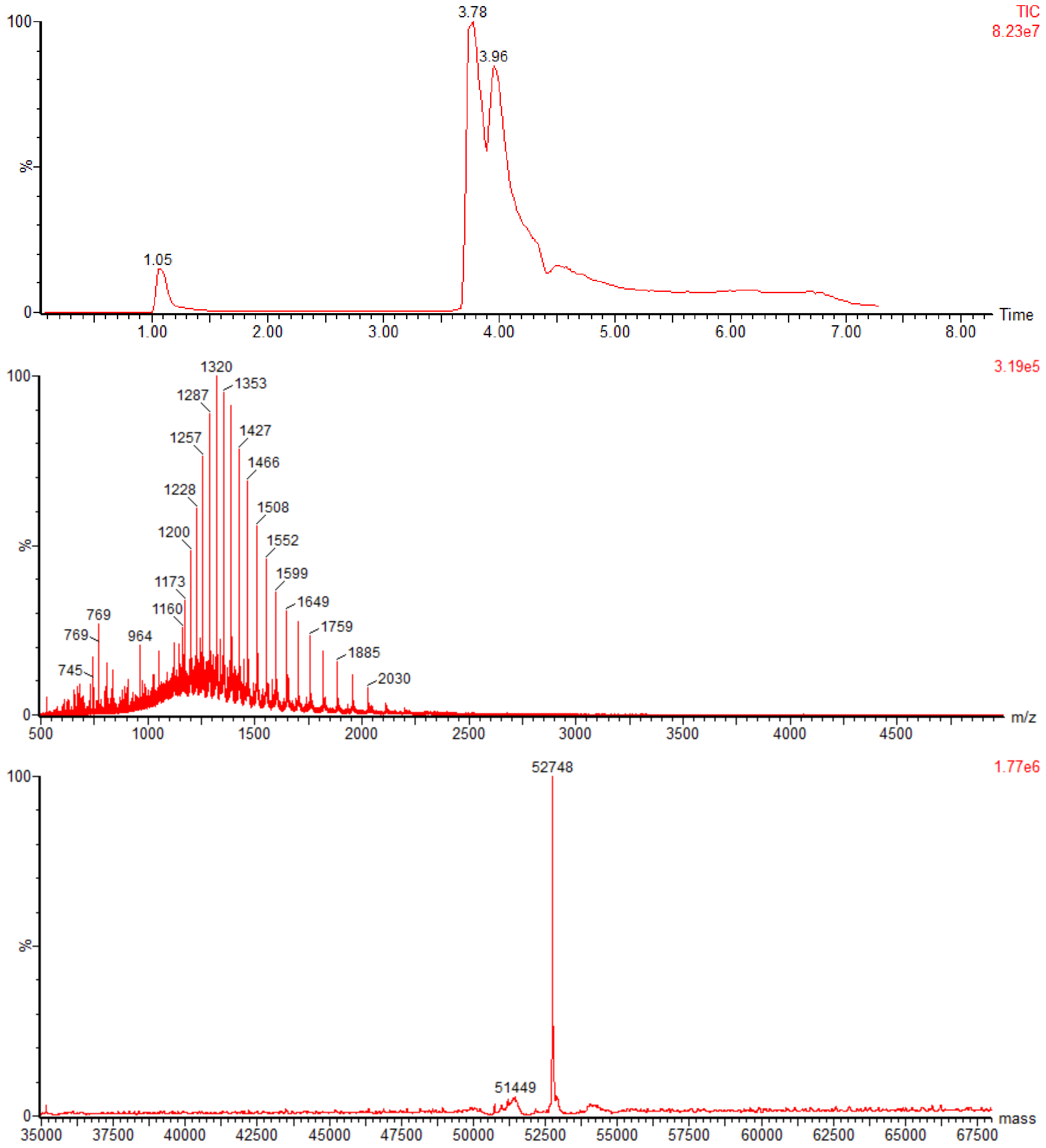

Figure S51: Fc-Afamelanotide (digest Fc) **13.** TIC trace (top), non-deconvoluted (middle) and deconvoluted (bottom) MS, expected 52750 Da and observed 52745 Da.

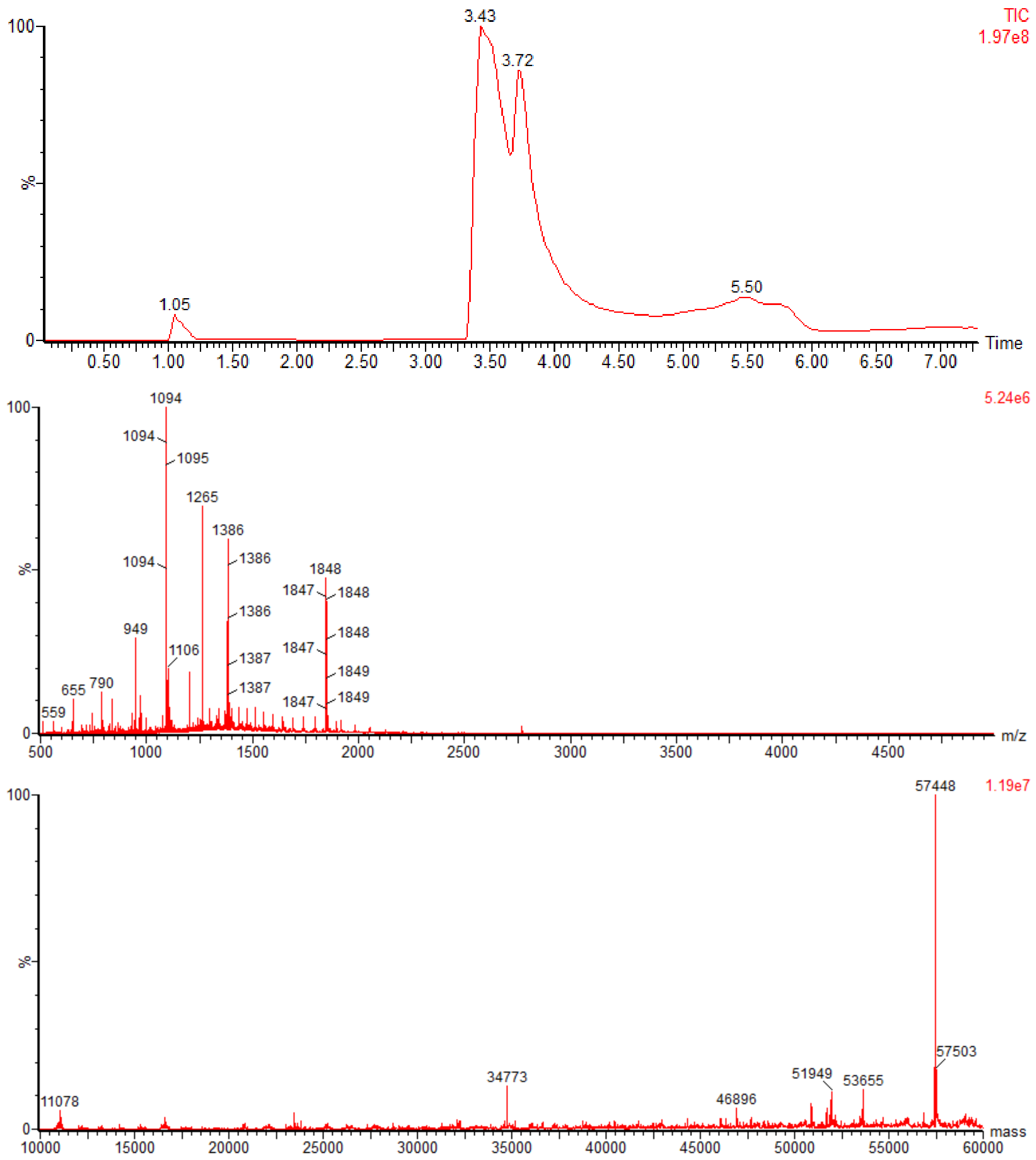

Figure S52: Fc-Exenatide **15** *before* protein A purification. TIC trace (top), non-deconvoluted (middle) and deconvoluted (bottom) MS.

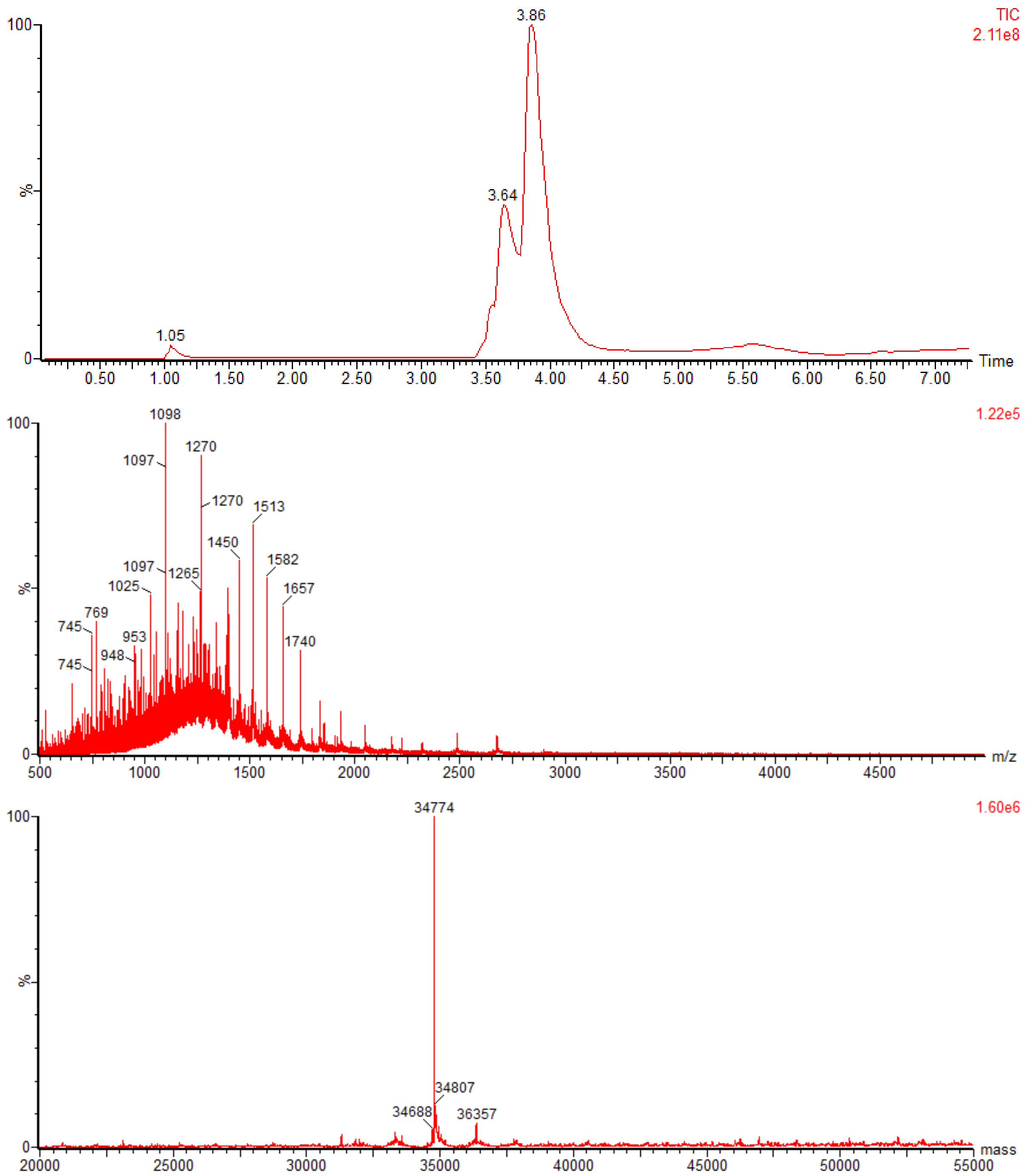

Figure S53: Analysis of 3.64 peak from LCMS of **15**, found 34774 Da = PNGase F. TIC trace (top), non-deconvoluted (middle) and deconvoluted (bottom) MS.

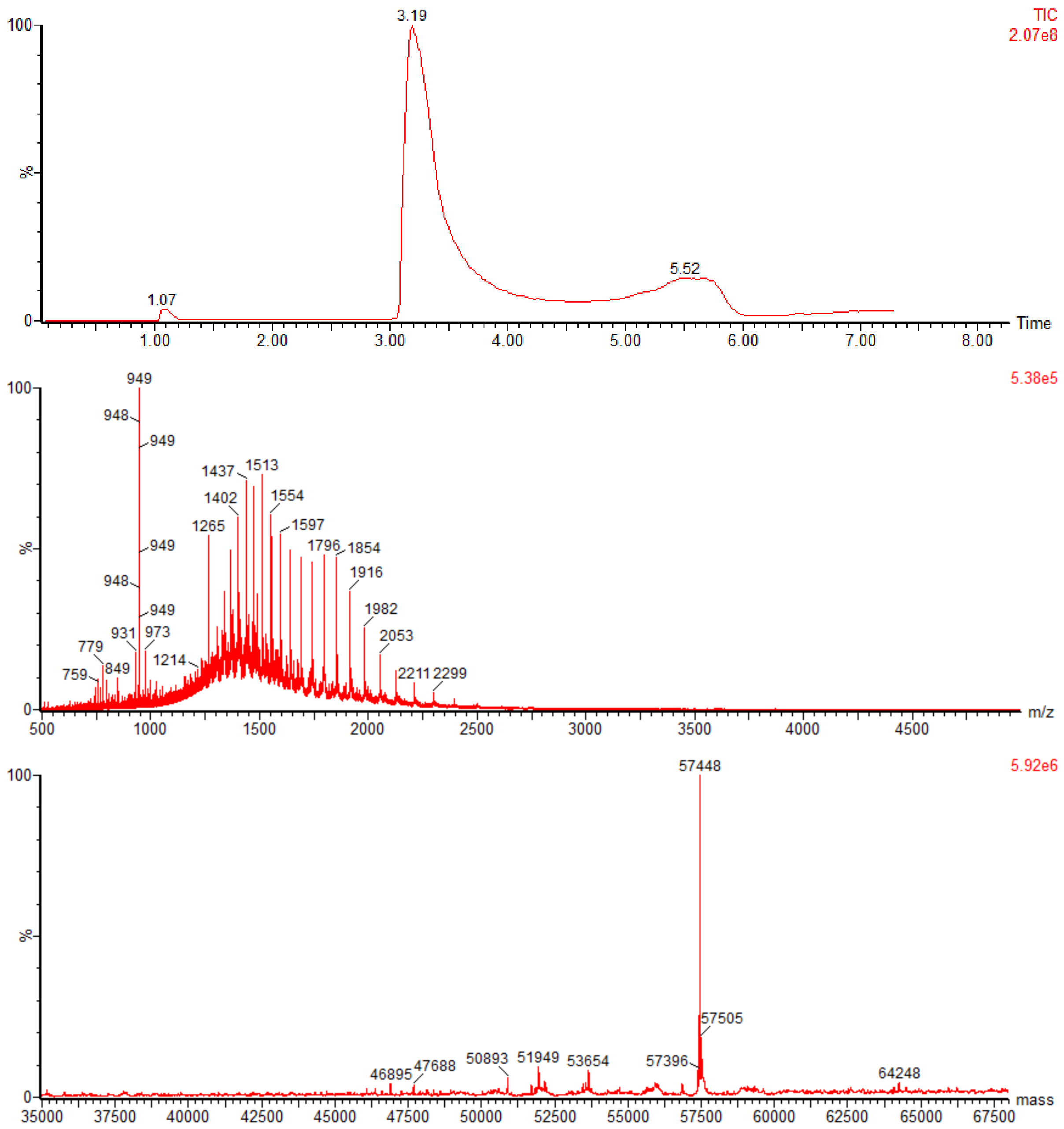

Figure S54: Fc-Exenatide **15** *after* protein A purification. TIC trace (top), non-deconvoluted (middle) and deconvoluted (bottom) MS, expected 57440 Da and observed 57446 Da.

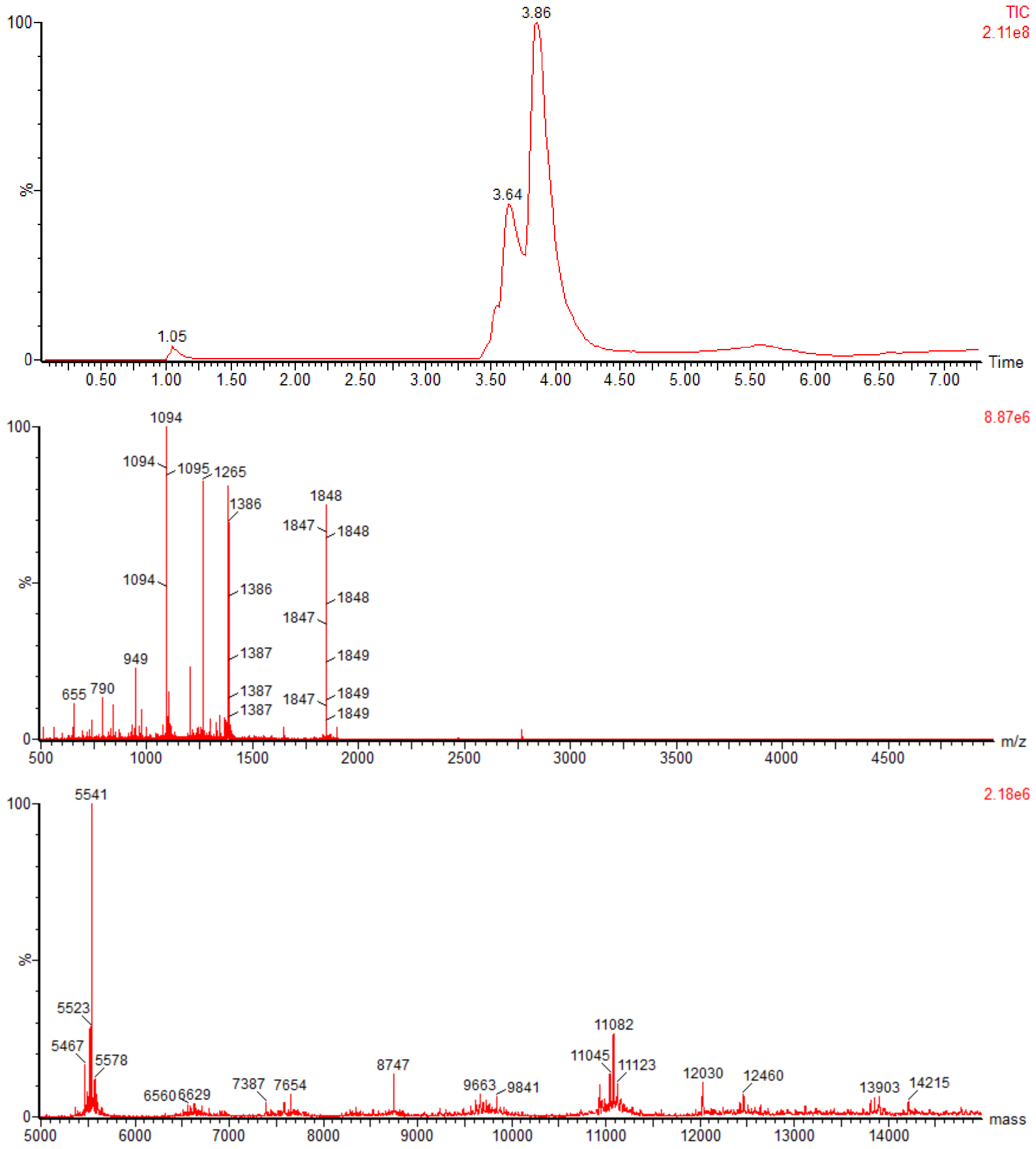

Figure S55: Washout from protein A purification of **15**. TIC trace (top), non-deconvoluted (middle) and deconvoluted (bottom) MS, expected 5540 Da and observed 5538 Da and 11079 Da (2M+H).

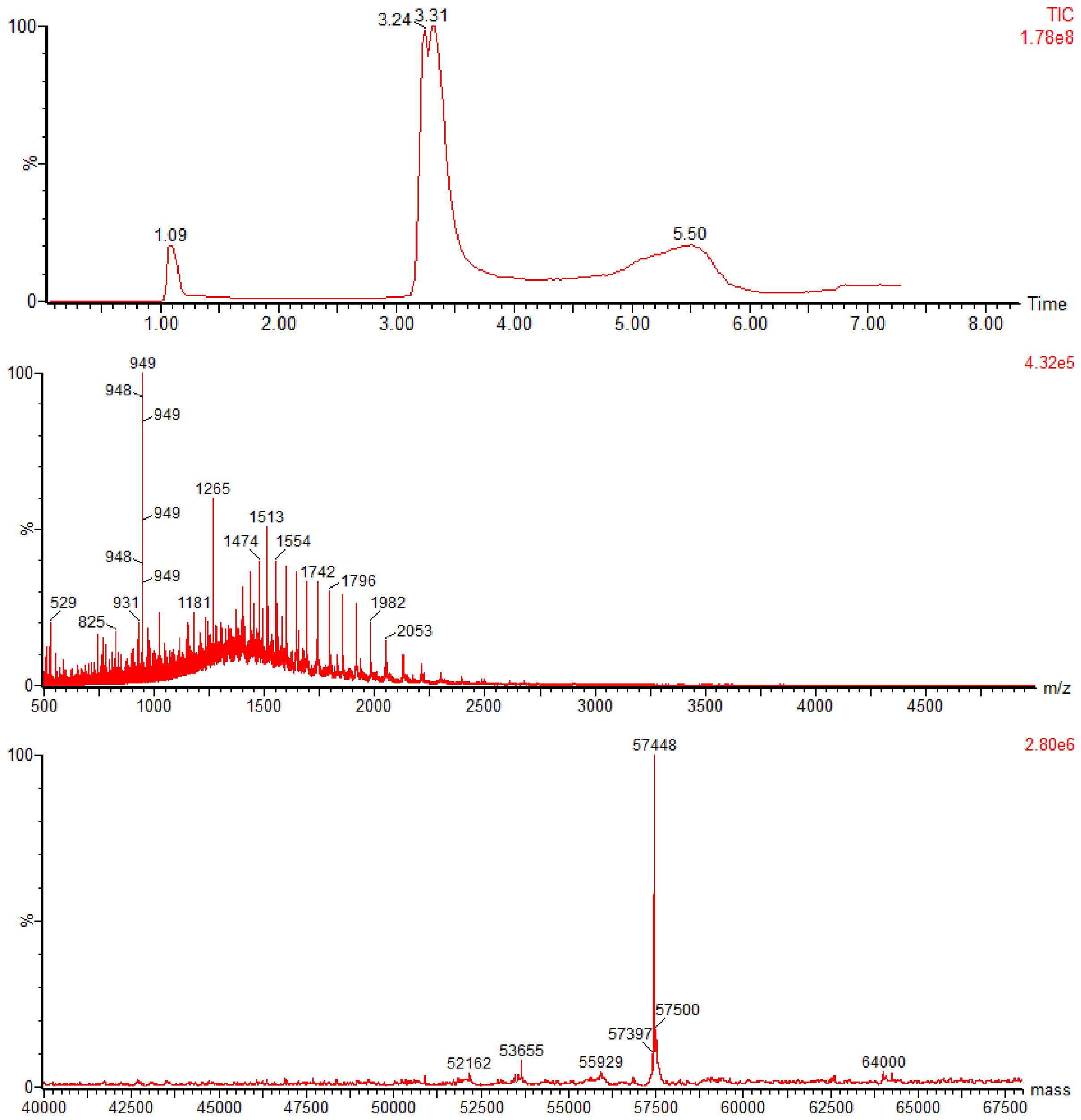

Figure S56: Fc-Exenatide **15** formed *via* room temperature bioconjugation and 4 °C SPAAC. TIC trace (top), non-deconvoluted (middle) and deconvoluted (bottom) MS, expected 57440 Da and observed 57448 Da and 64000 Da (Fc+2BisDVP+2Exenatide = 'over addition' species).

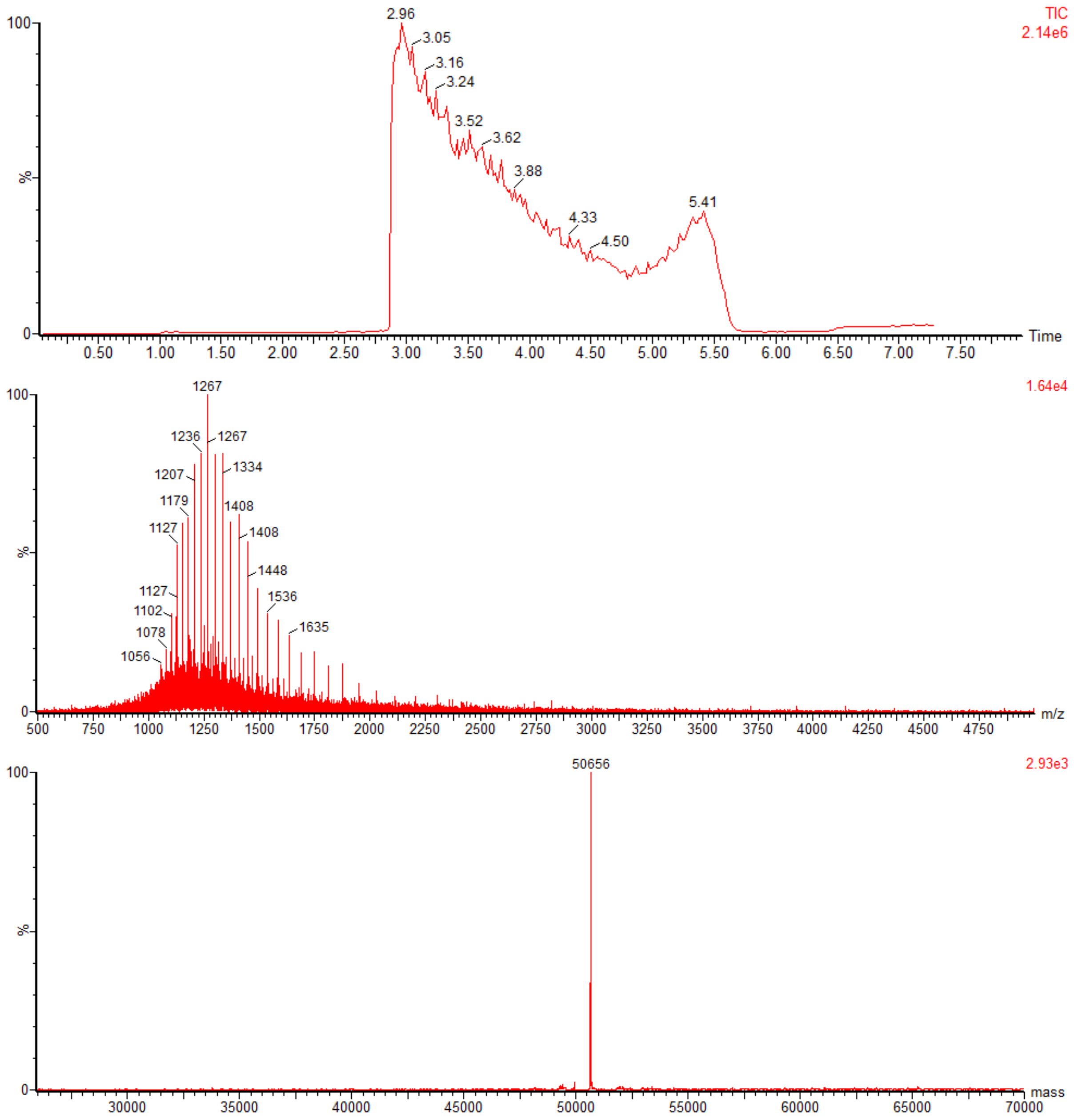

Figure S57: Bioconjugation of digest Fc-BisDVP quenched with N-(tert-Butoxycarbonyl)-L-cysteine methyl ester. TIC trace (top), non-deconvoluted (middle) and deconvoluted (bottom) MS, expected mass: 50649 Da, found: 50656 Da. Cropped data shown in **Figure S10**.

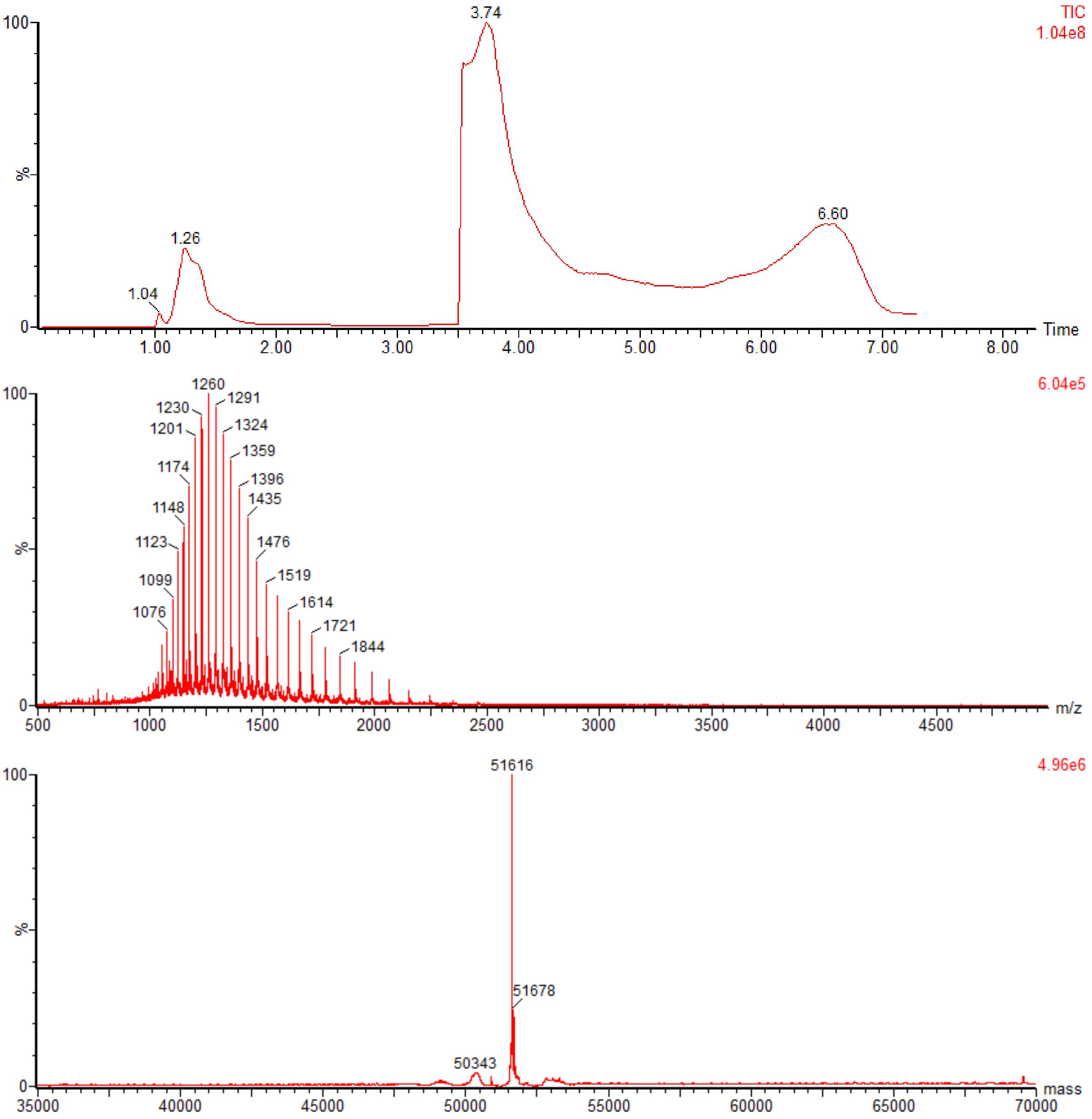

Figure S58: Bioconjugation of recombinant Fc-BisDVP quenched with N-(tert-Butoxycarbonyl)-L-cysteine methyl ester. TIC trace (top), non-deconvoluted (middle) and deconvoluted (bottom) MS, expected mass: 51606 Da, found: 51616 Da. Cropped data shown in **Figure S10**.

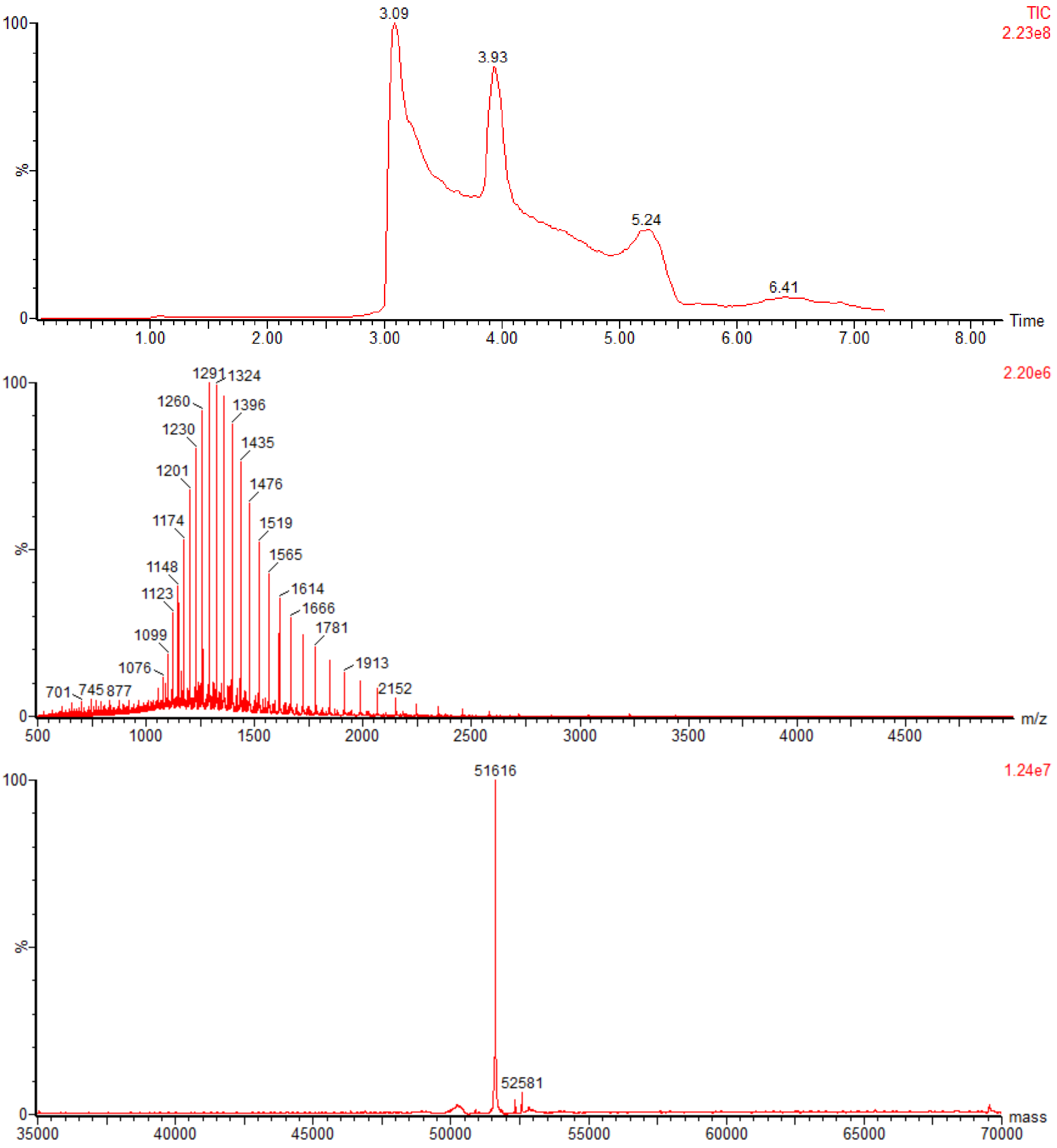

Figure xx: Bioconjugation of recombinant Fc **4** with BisDVP **1** to form **S22**. TIC trace (top), non-deconvoluted (middle) and deconvoluted (bottom) MS, expected mass: 51608 Da; found: 51615 Da.

NMR Spectra

Ethyl 4-((4,6-dichloropyrimidin-2-yl)amino)butanoate (S1)

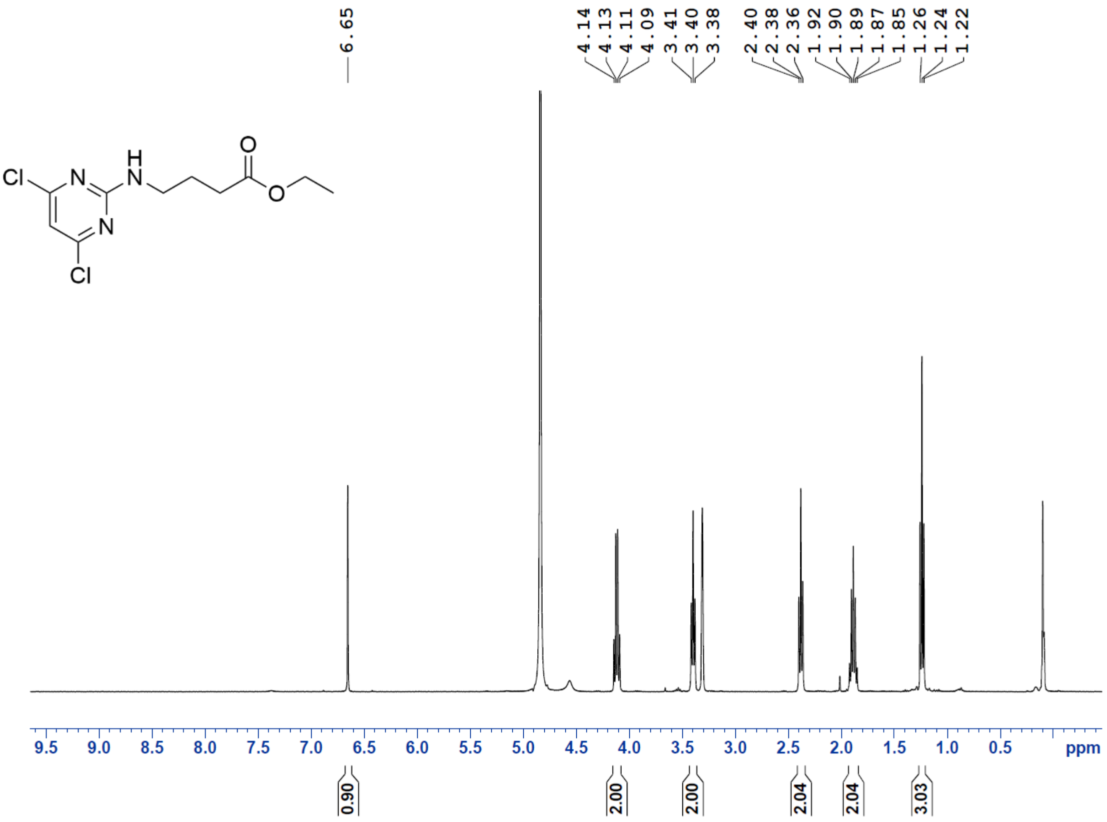

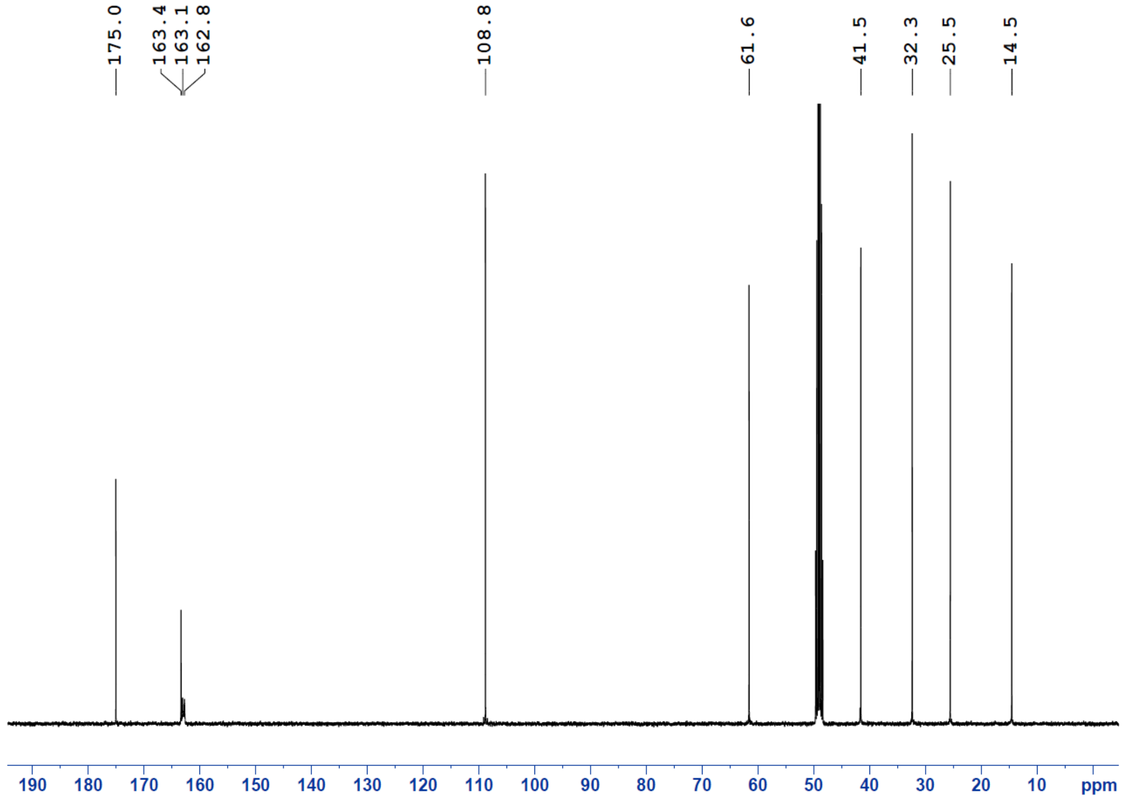

Ethyl 4-((4,6-divinylpyrimidin-2-yl)amino)butanoate (S2)

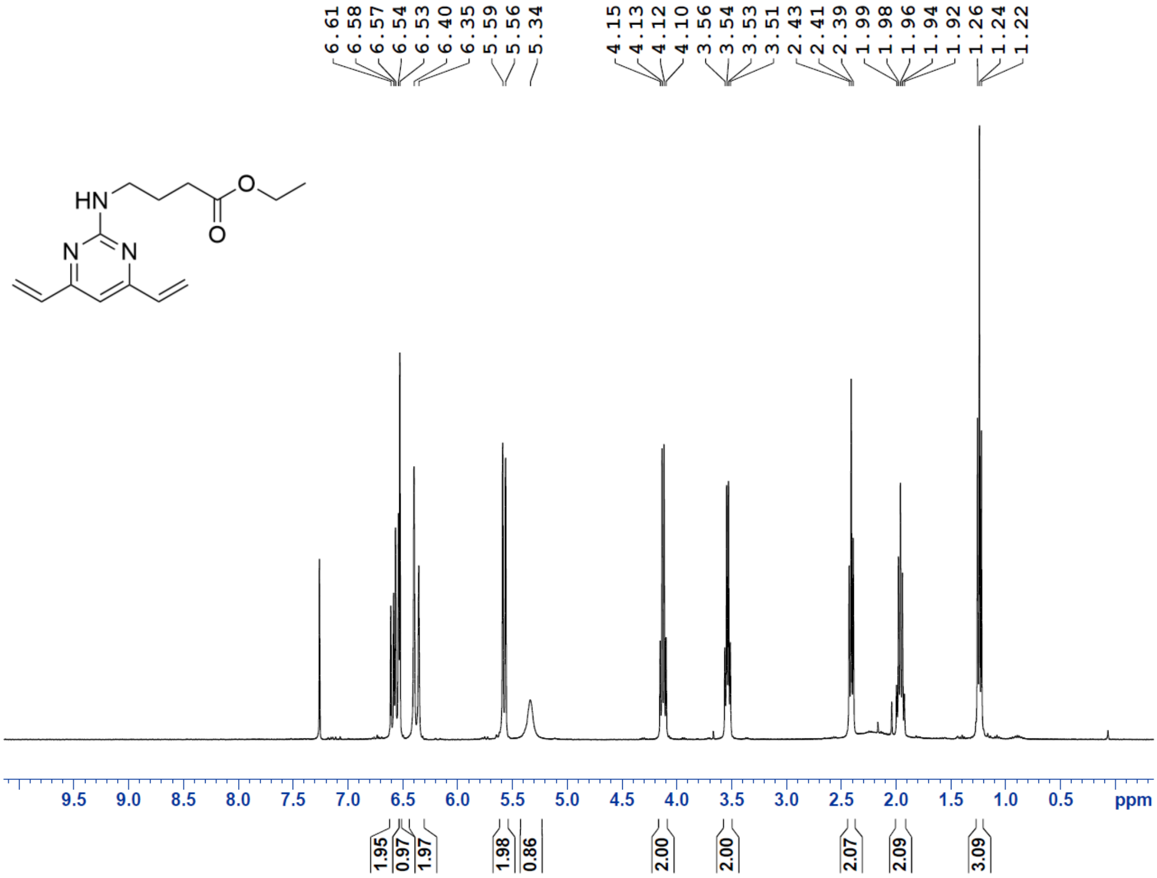

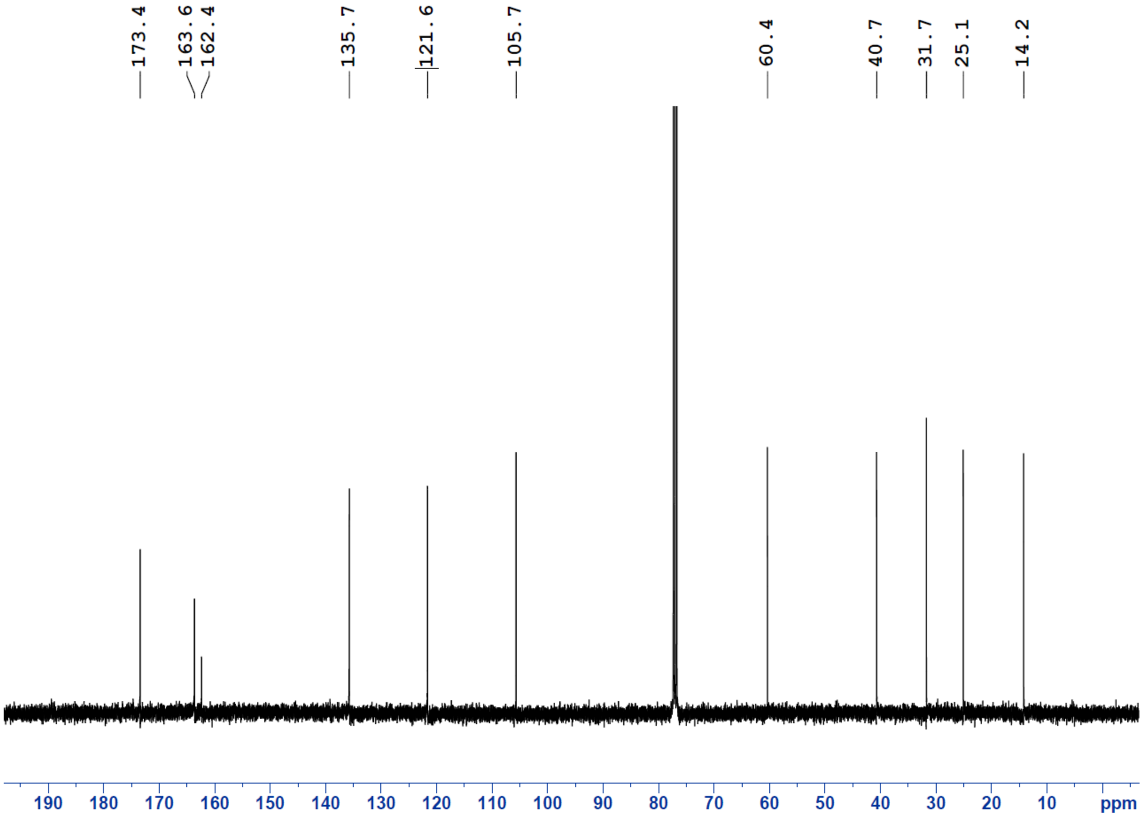

4-((4,6-divinylpyrimidin-2-yl)amino)butanoic acid (S3)

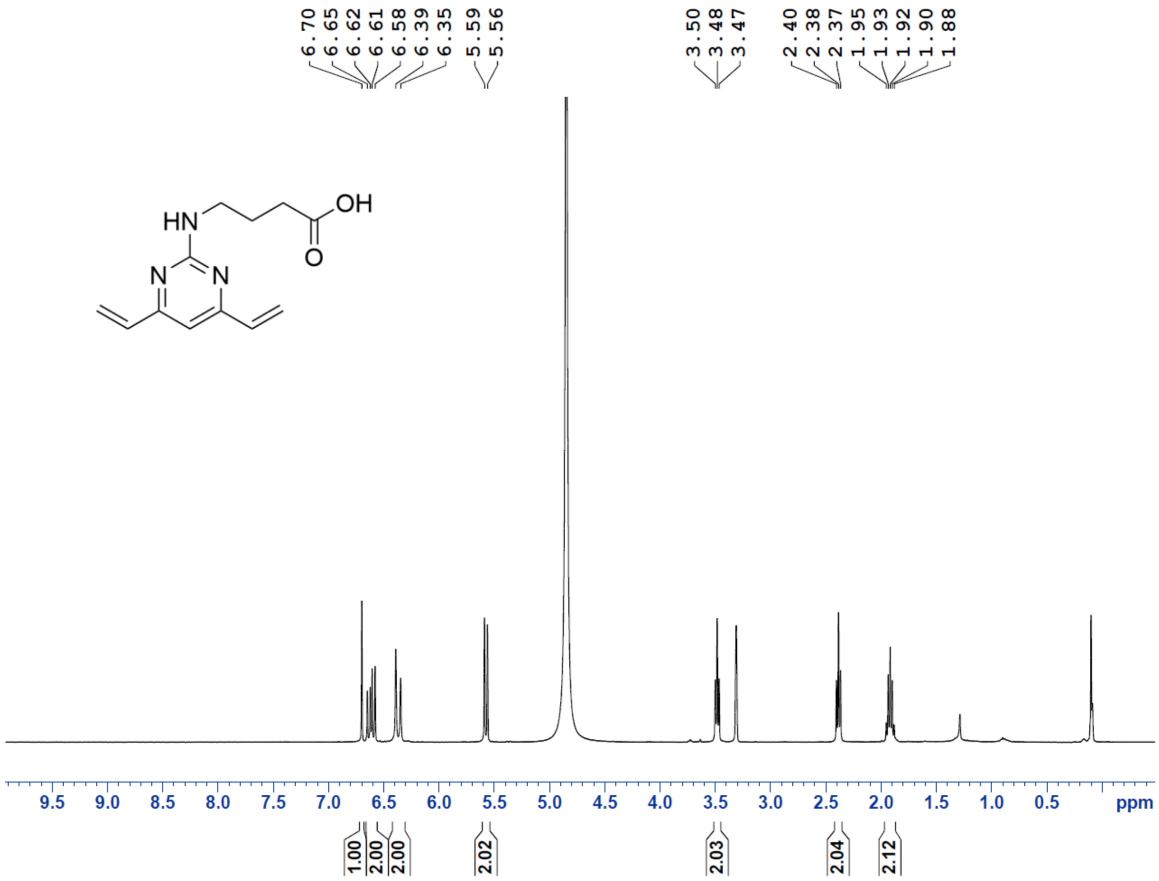

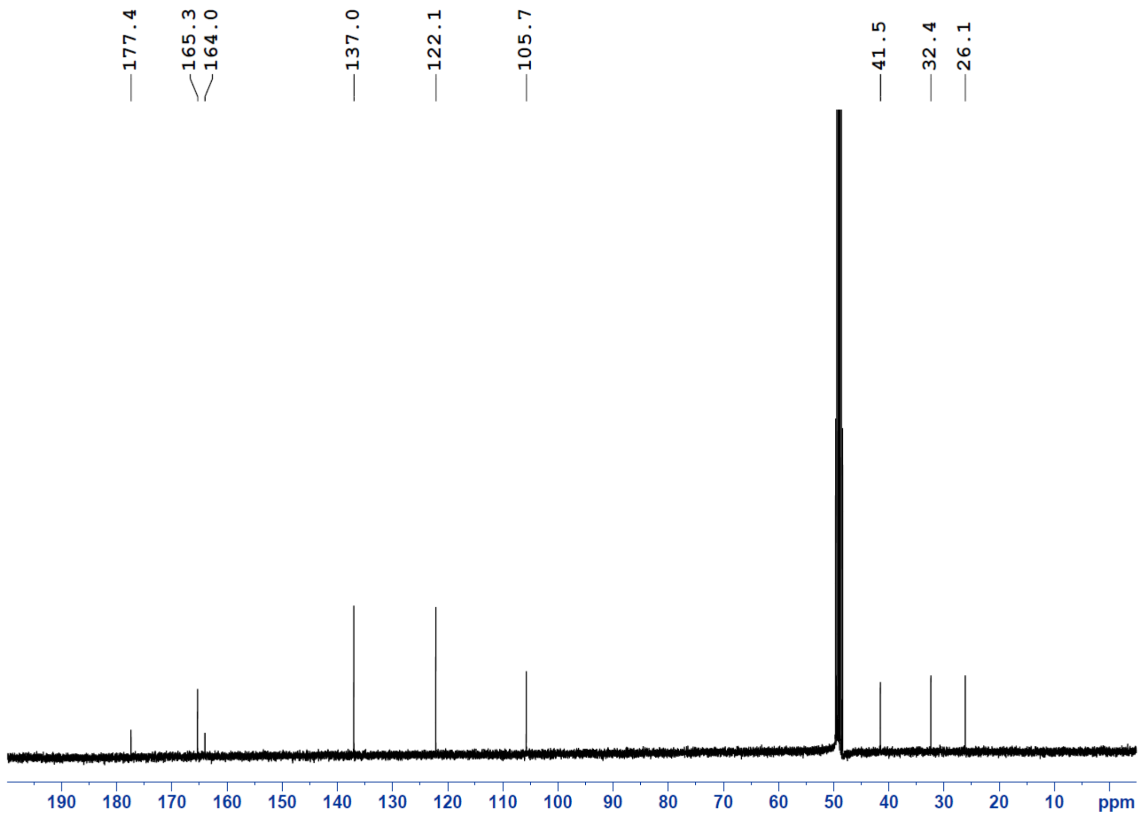

di-tert-butyl (azanediylbis(ethane-2,1-diyl))dicarbamate (S4)

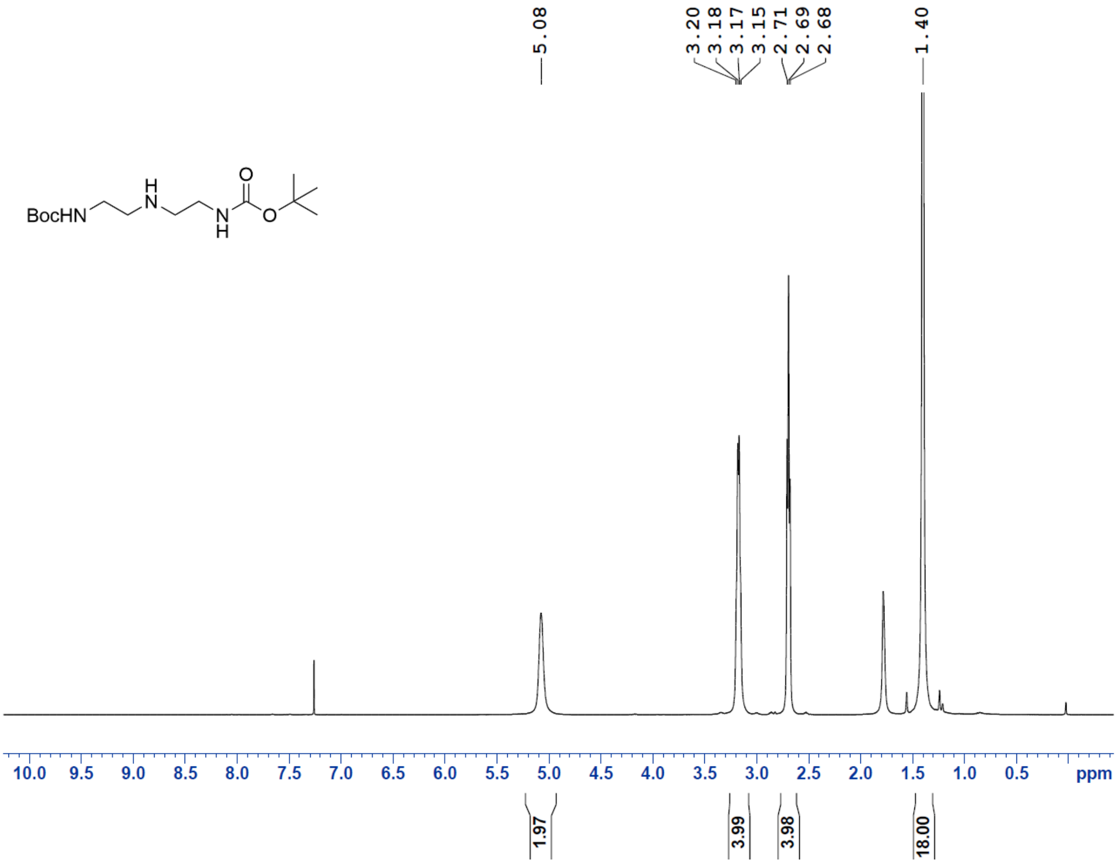

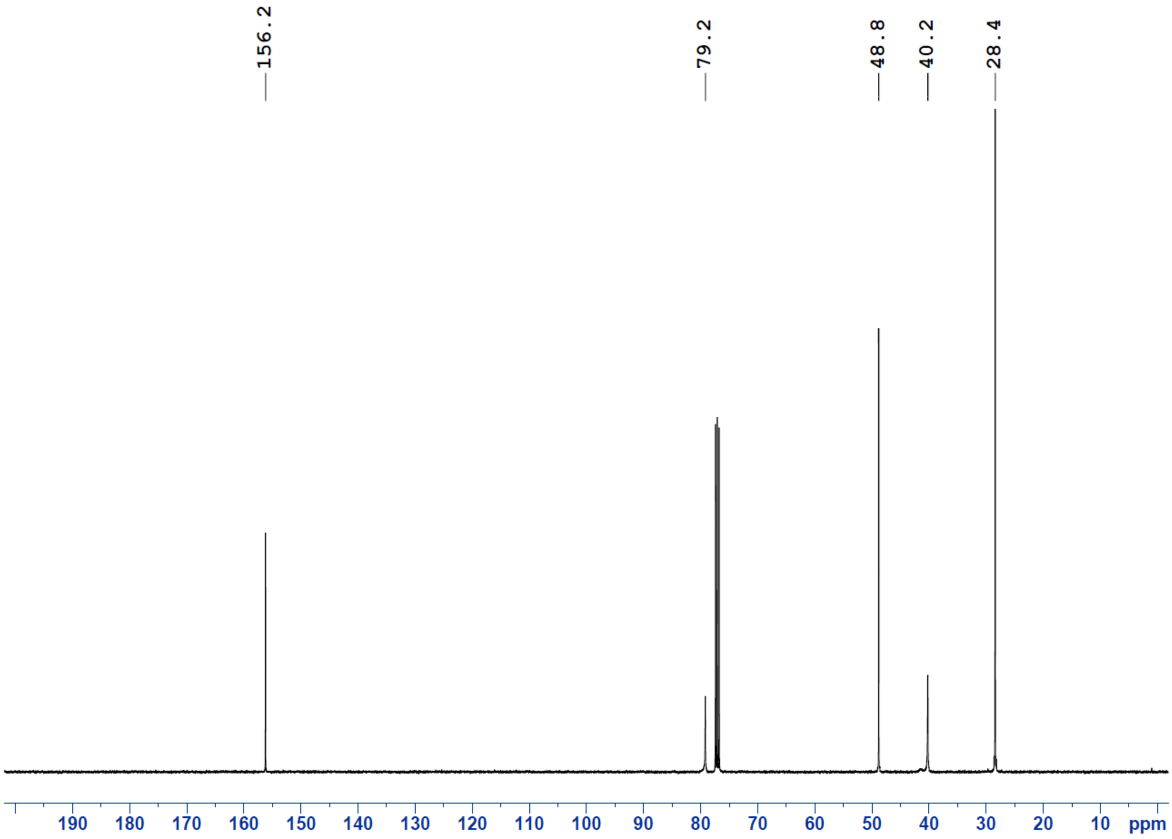

2-bromo-N-(2-(2-(prop-2-yn-1-yloxy)ethoxy)ethyl)acetamide (S5)

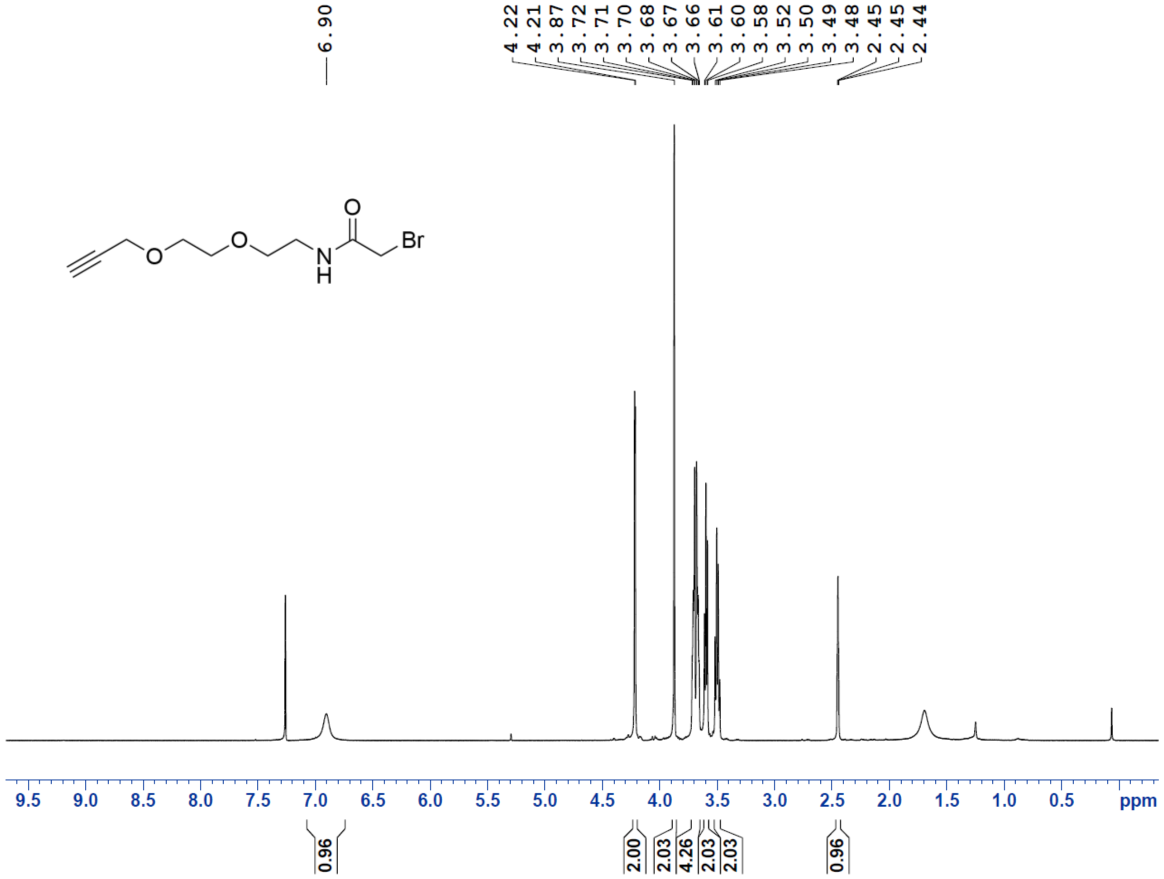

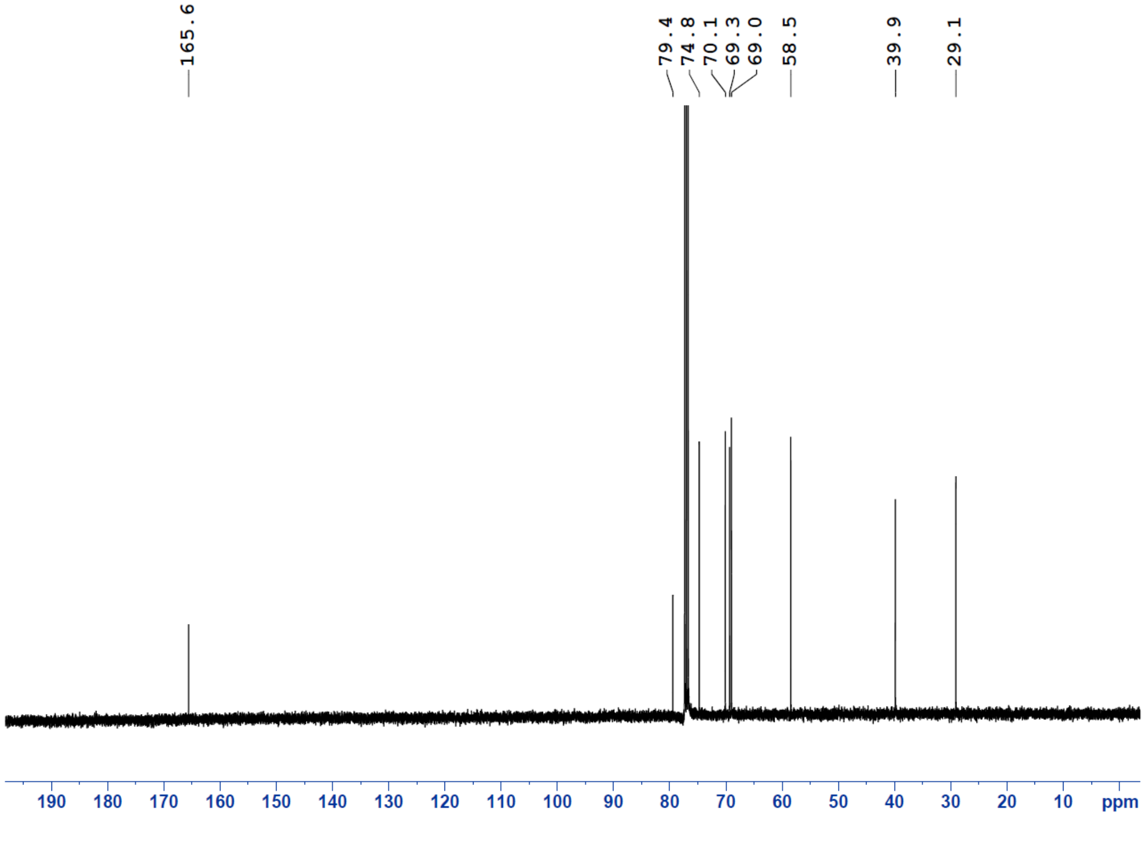

Bis-N-Boc-amine intermediate (PEG2-alkyne) (S6)

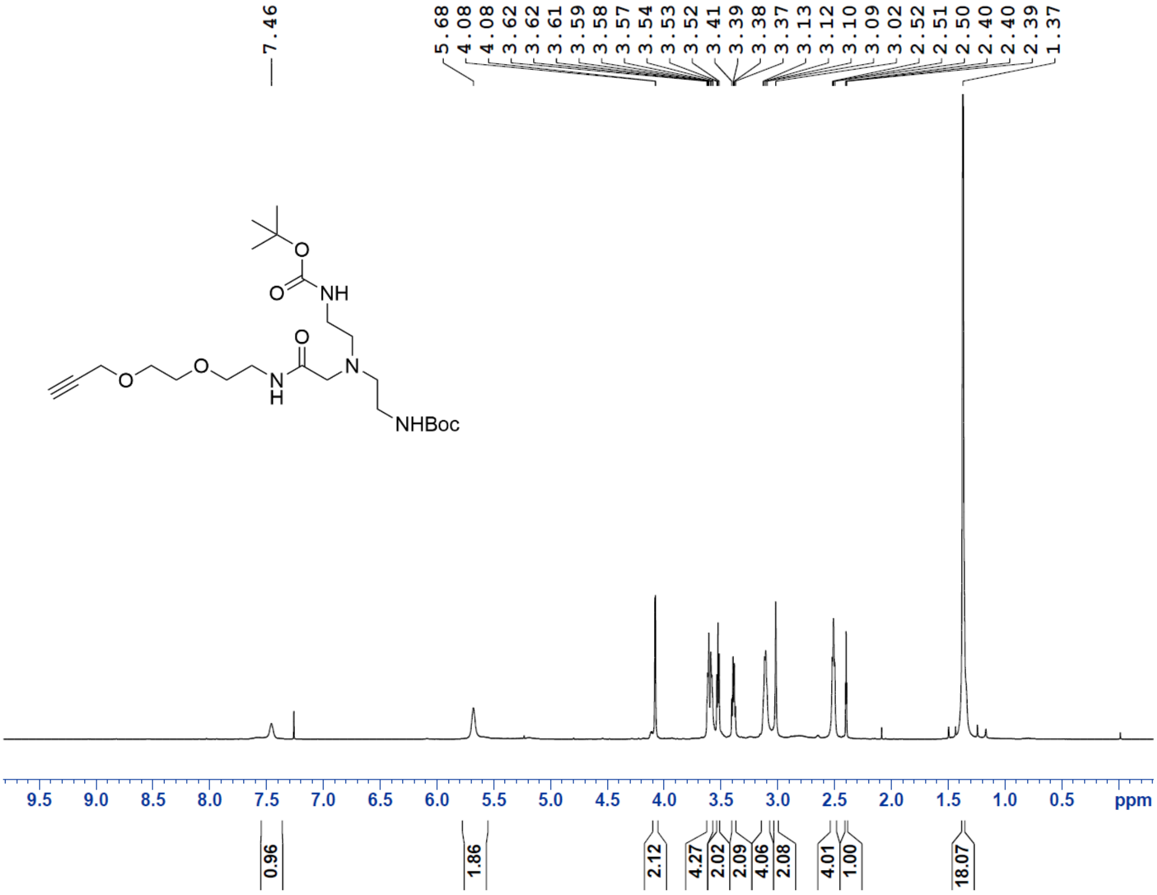

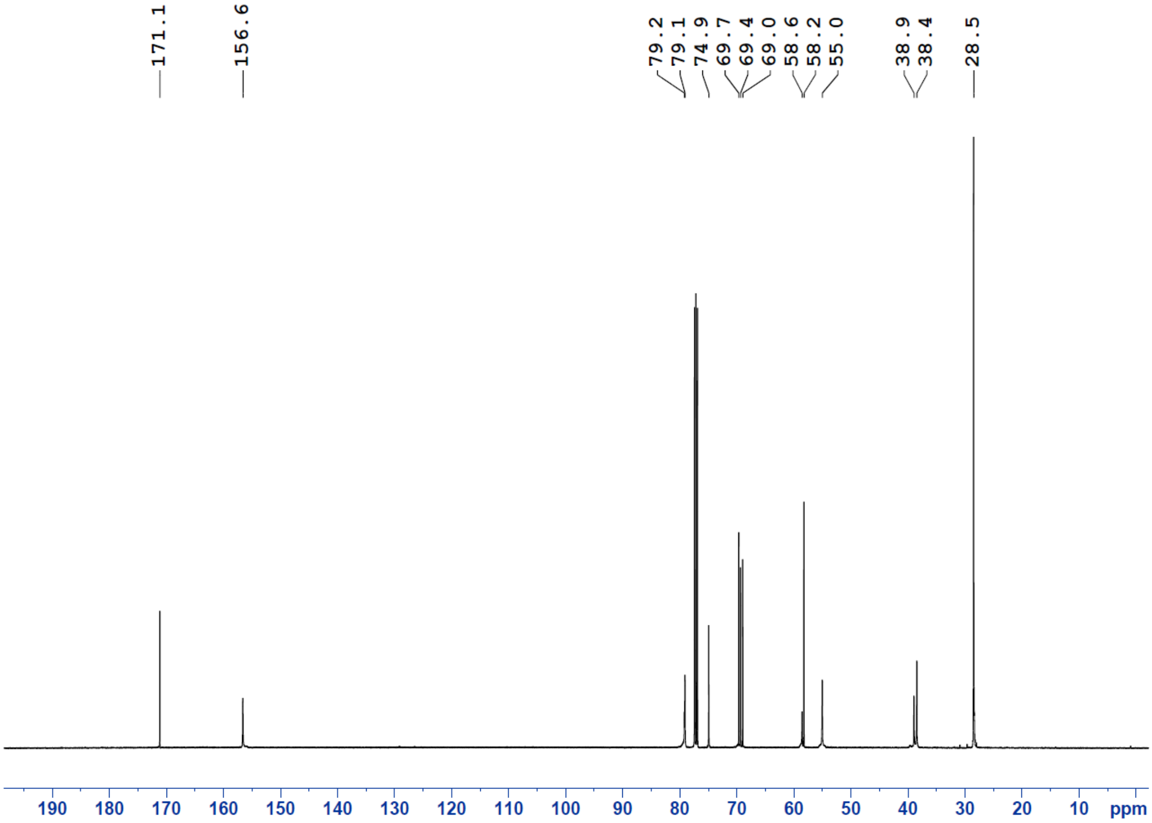

Bis-divinylpyrimidine Alkyne Linker (BisDVP 1)

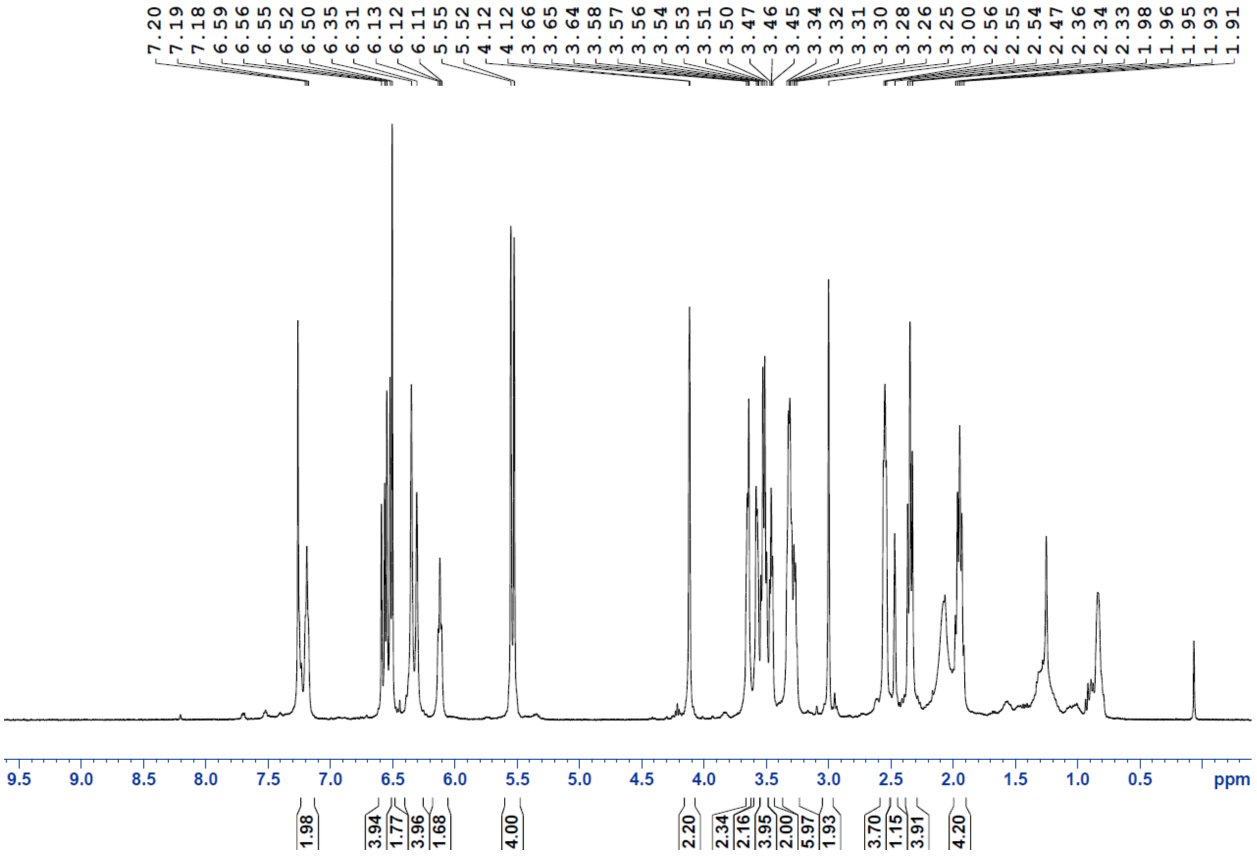

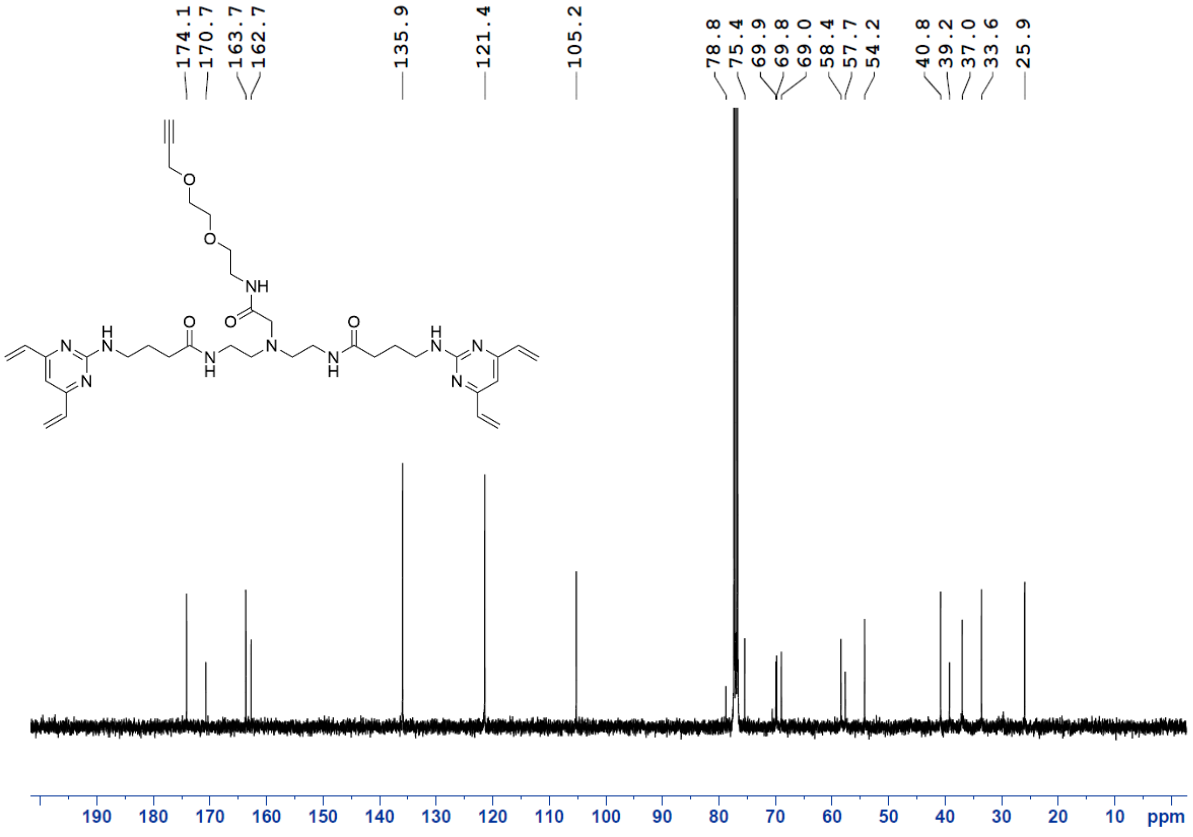

N-(2-(2-(2-(2-azidoethoxy)ethoxy)ethoxy)ethyl)oct-7-yn-1-amine (S8)

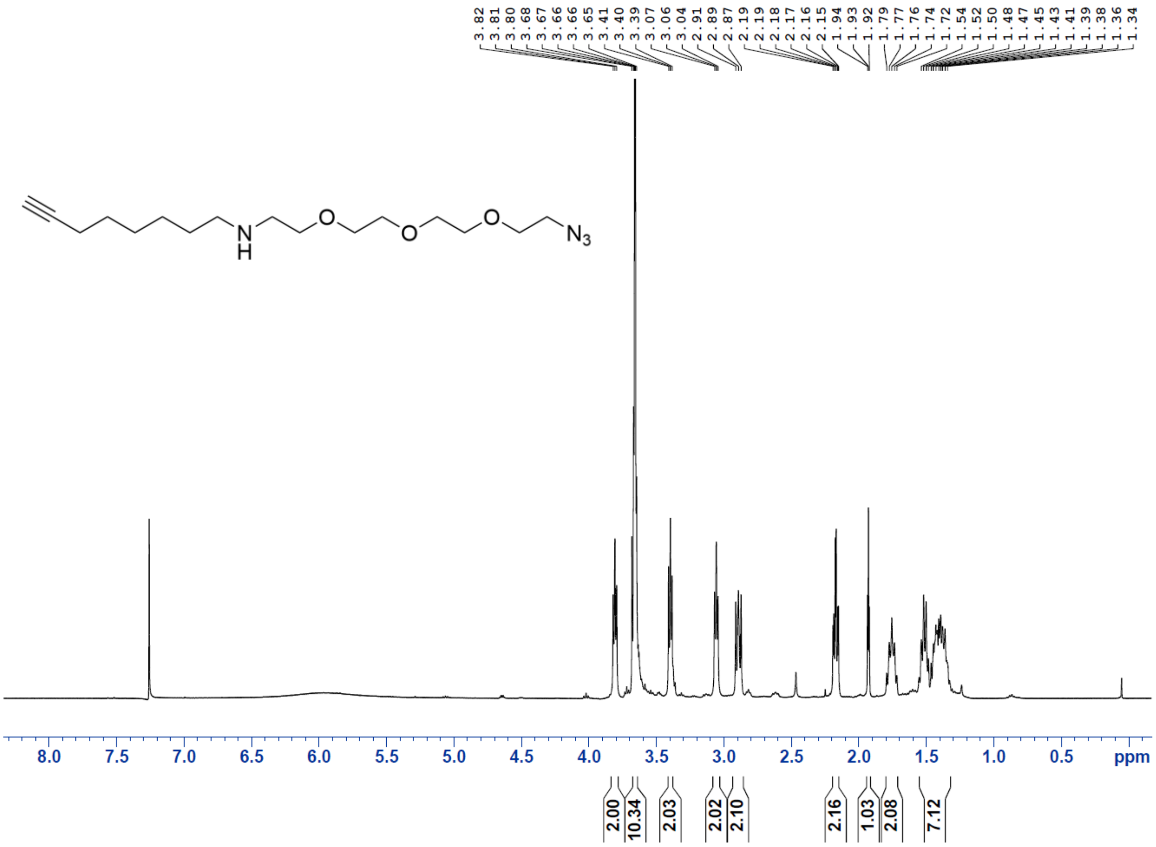

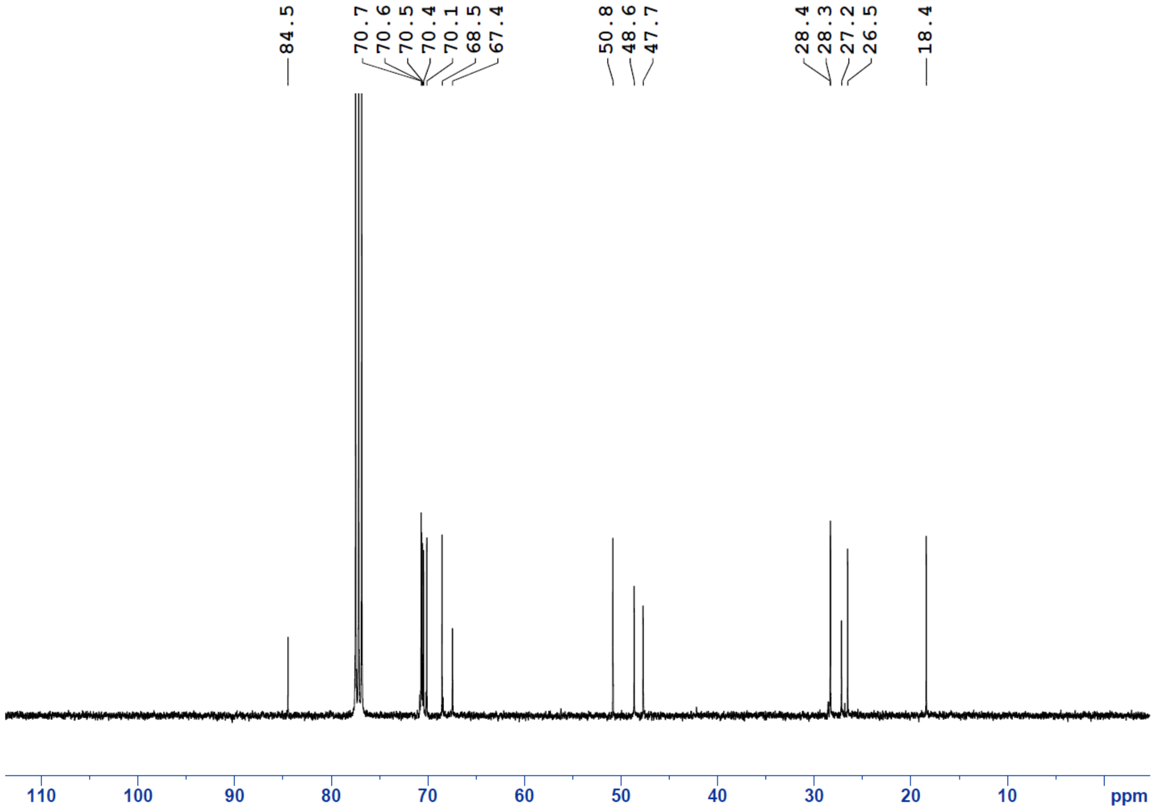

***tert*-Butyl (20-azido-9-(oct-7-yn-1-yl)-8-oxo-3,6,12,15,18-pentaoxa-9-azaicosyl)carbamate (S9)**

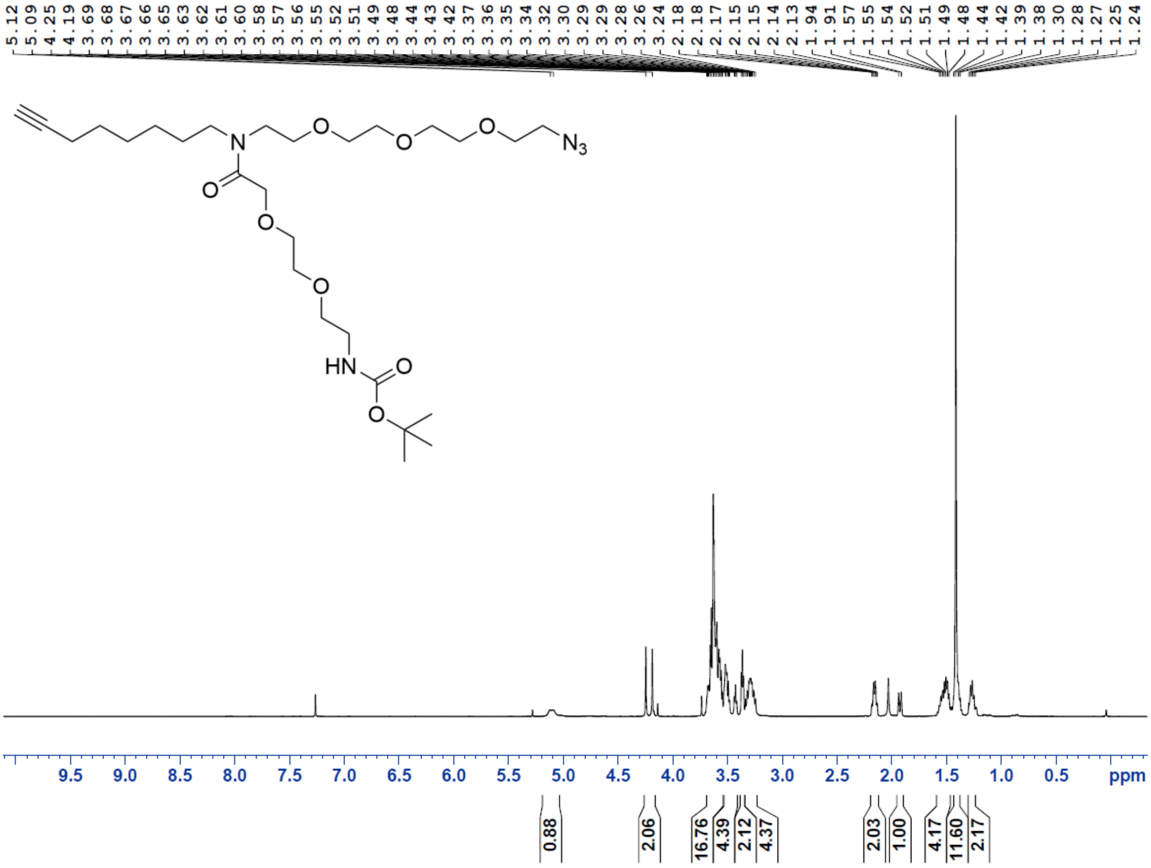

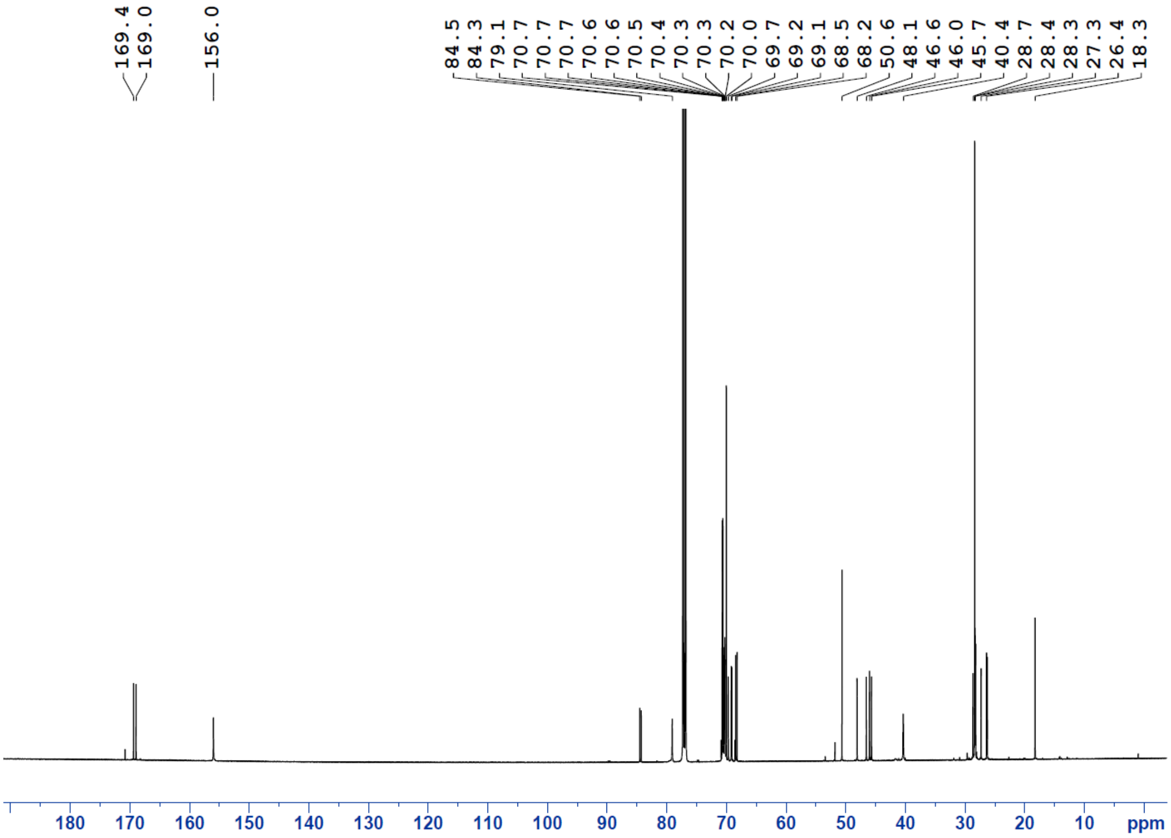

Methyl bis(2-((tert-butoxycarbonyl)amino)ethyl)glycinate (S10)

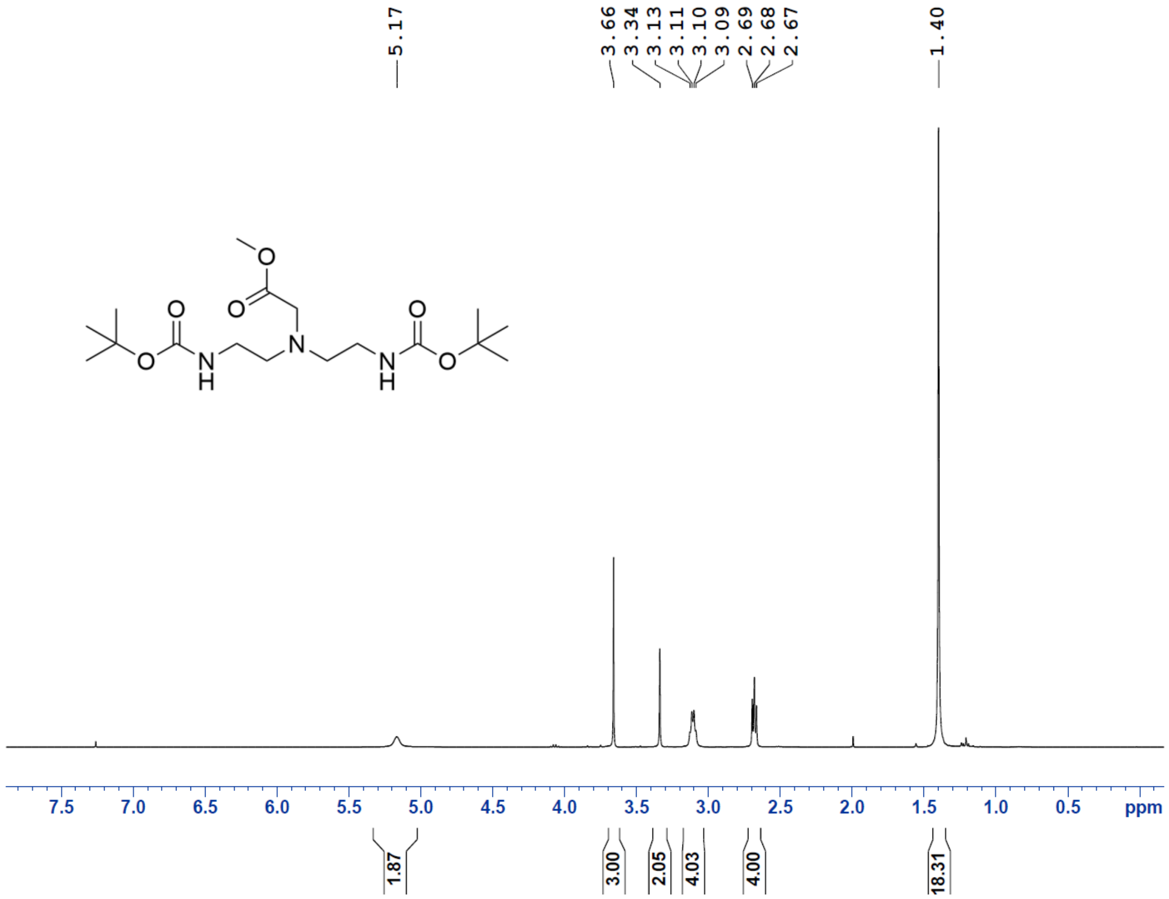

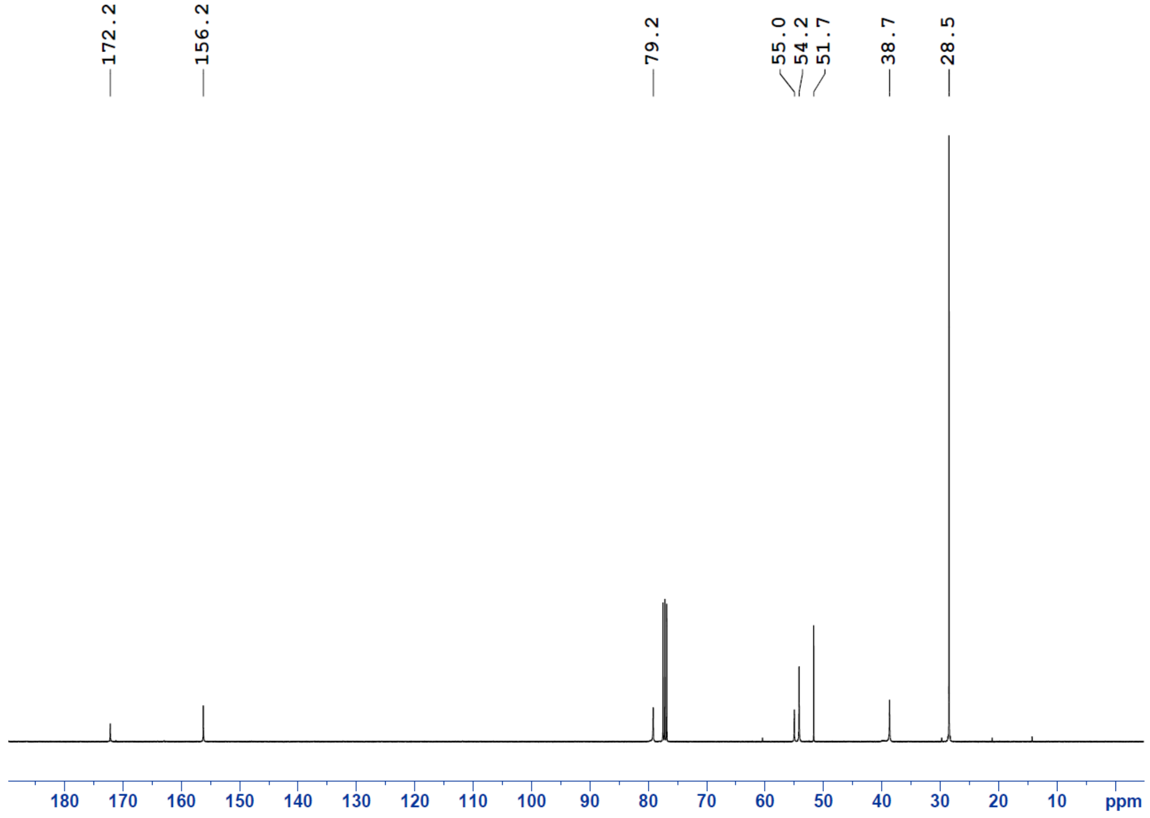

tert-butyl (20-azido-9-(oct-7-yn-1-yl)-8-oxo-3,6,12,15,18-pentaoxa-9-azaicosyl)carbamate (S13)

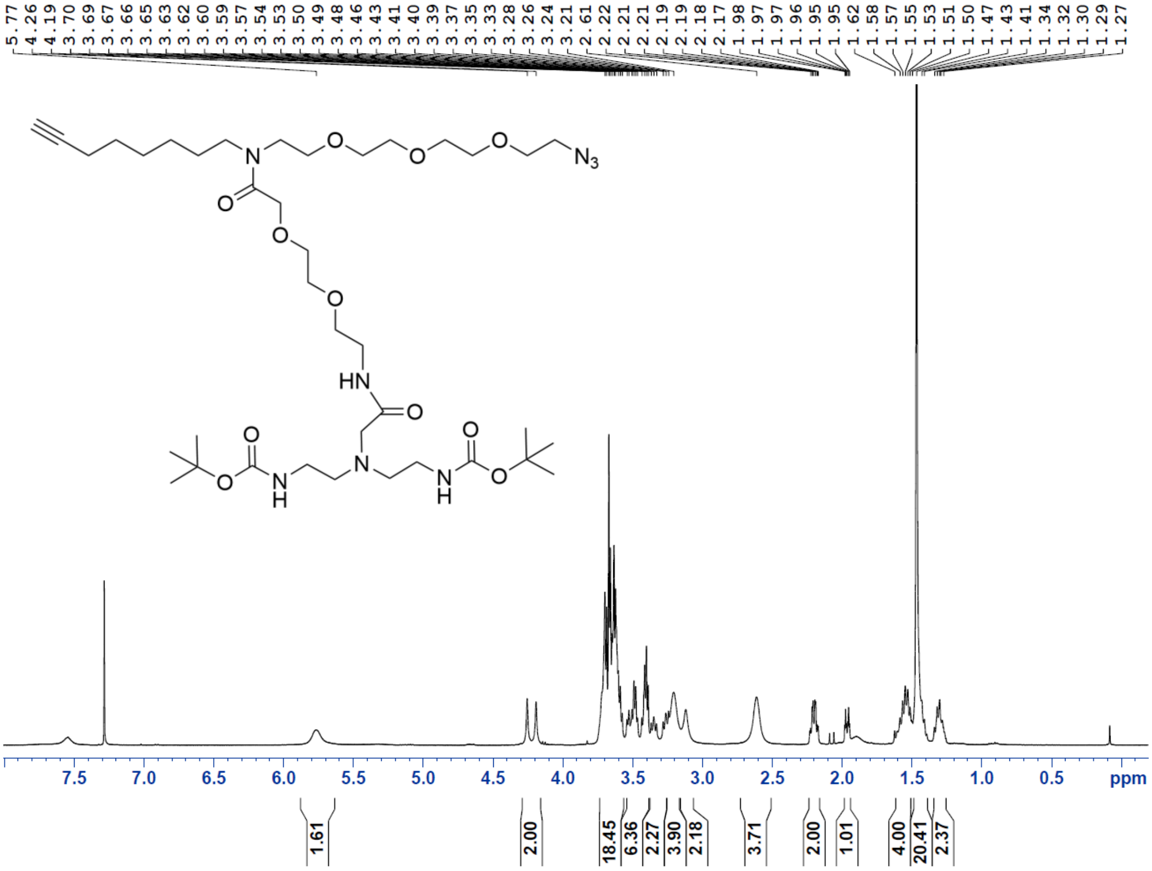

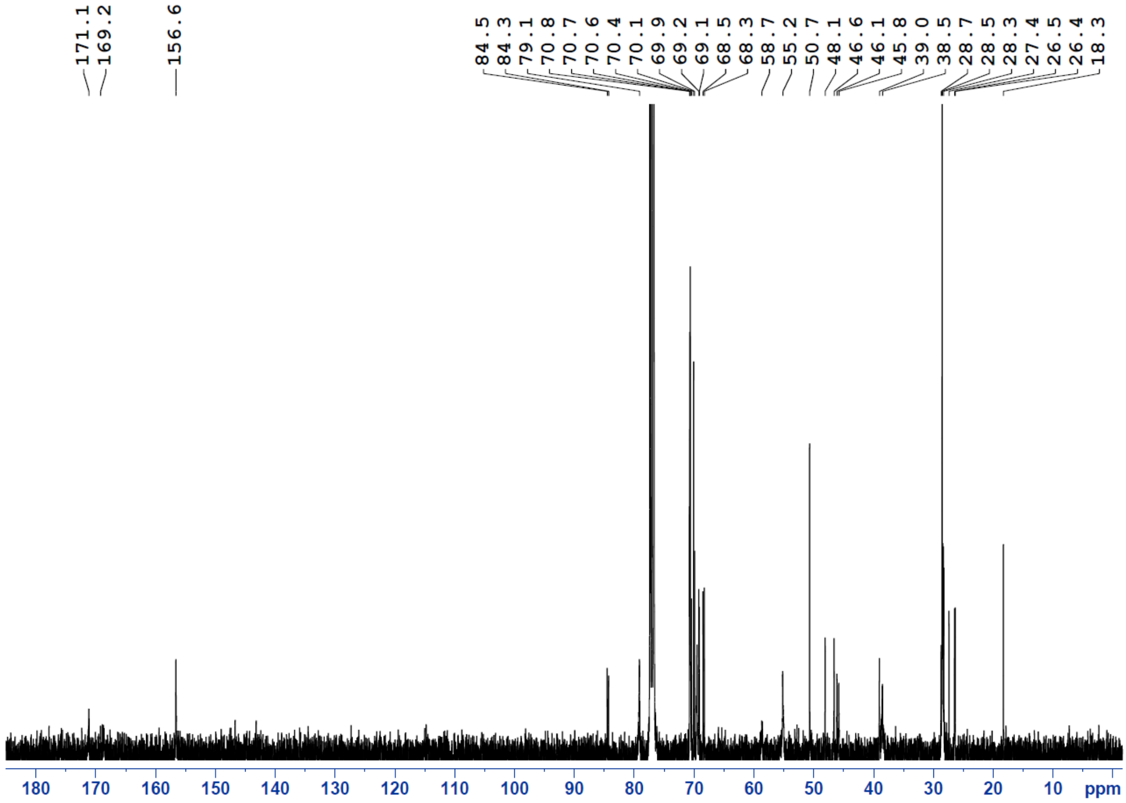

Bis-divinylpyrimidine Dual-functional alkyne-azide Linker (BisDVP 2)

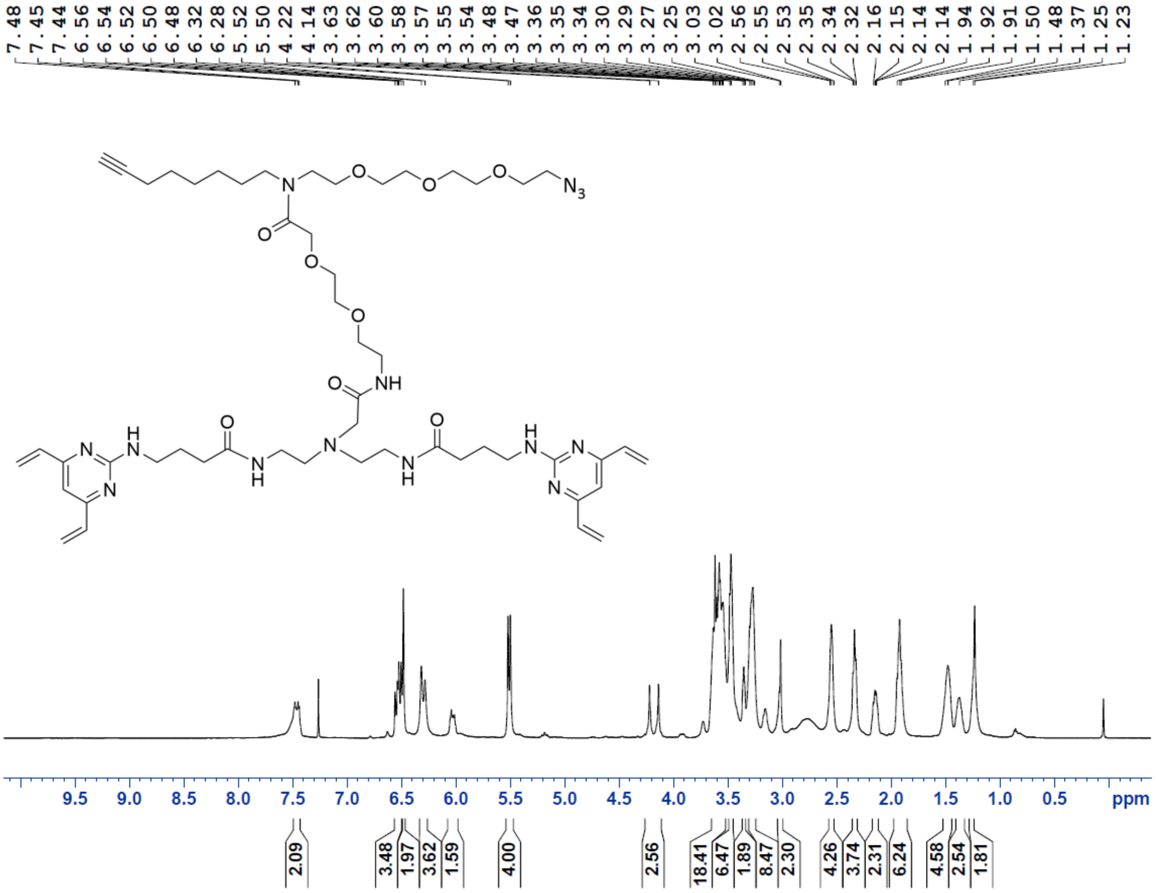

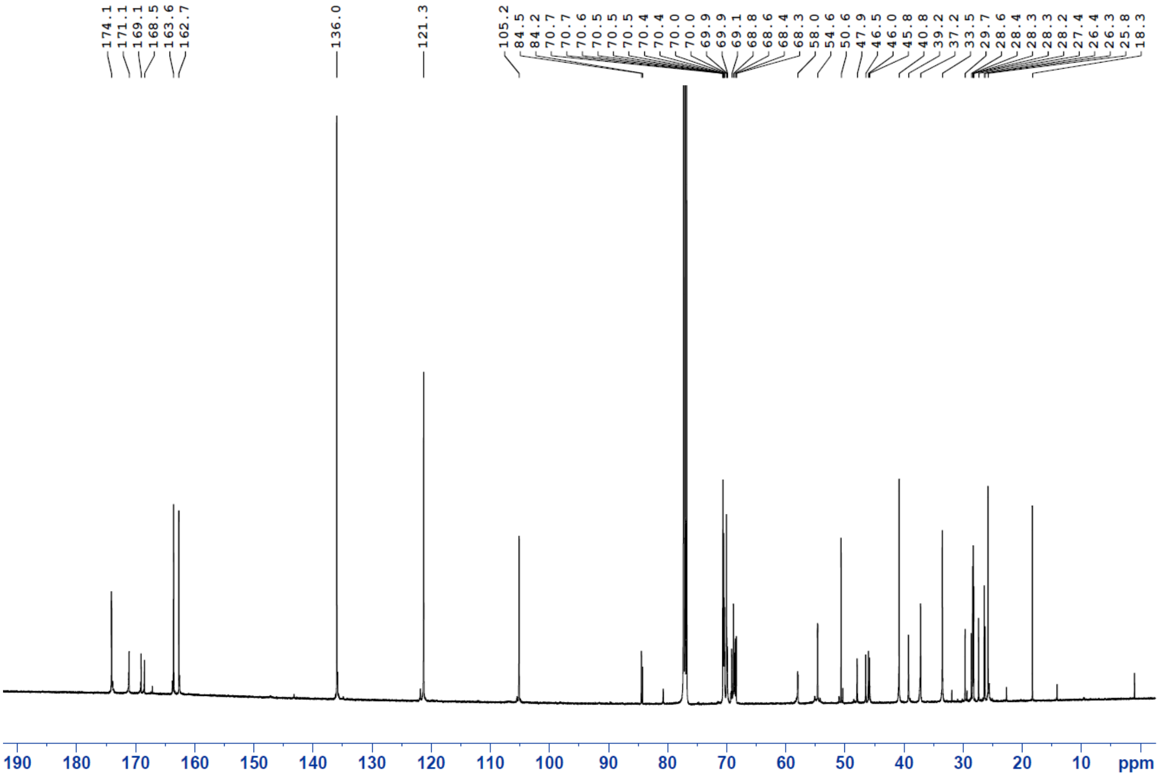

DBCO-COOH

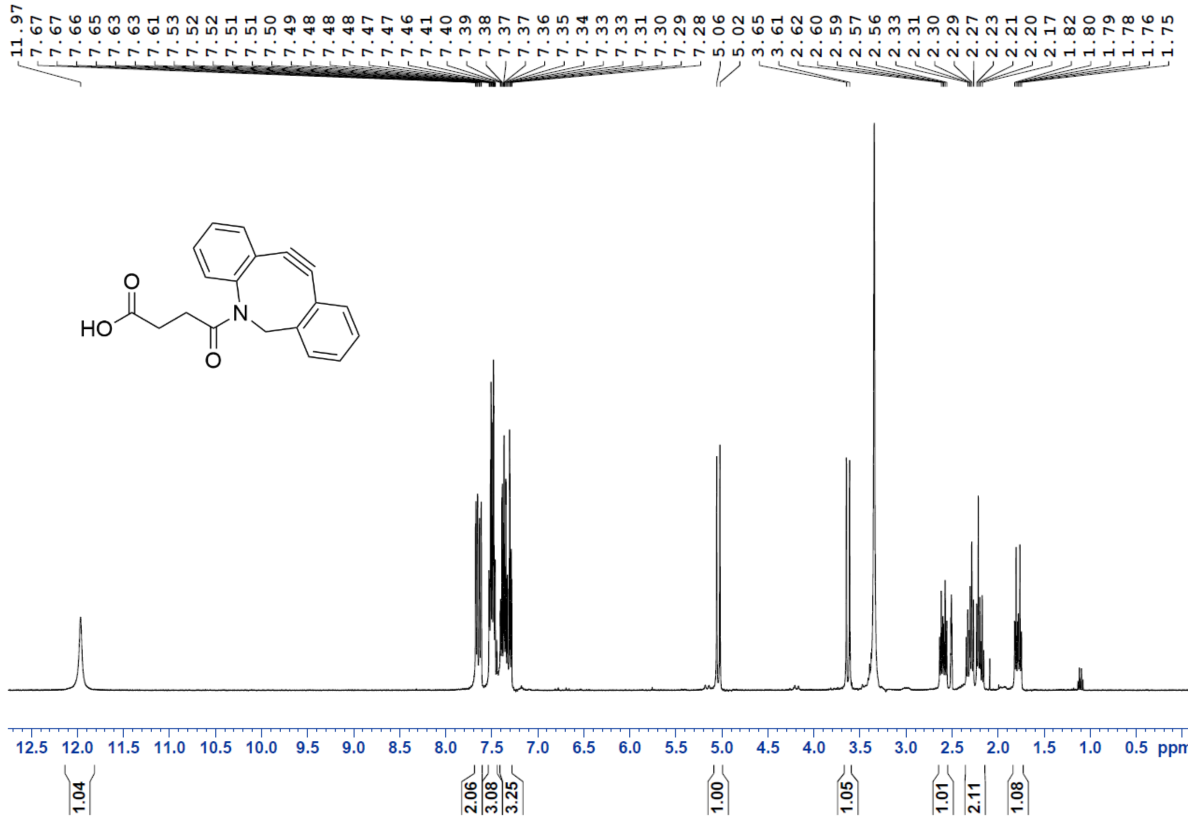
11.97
7.67
7.67
7.66
7.65
7.63
7.63
7.61
7.53
7.52
7.52
7.51
7.51
7.50
7.49
7.48
7.48
7.48
7.47
7.46
7.41
7.40
7.39
7.38
7.37
7.37
7.36
7.35
7.34
7.33
7.33
7.31
7.30
7.29
7.28
5.06
5.02
3.65
3.61
2.62
2.60
2.59
2.57
2.56
2.33
2.31
2.30
2.29
2.27
2.23
2.21
2.20
2.17
1.82
1.80
1.79
1.78
1.76
1.75
HO
O
N
O
12.5 12.0 11.5 11.0 10.5 10.0 9.5 9.0 8.5 8.0 7.5 7.0 6.5 6.0 5.5 5.0 4.5 4.0 3.5 3.0 2.5 2.0 1.5 1.0 0.5 ppm
1.04
2.06
3.08
3.25
1.00
1.05
1.01
2.11
1.08

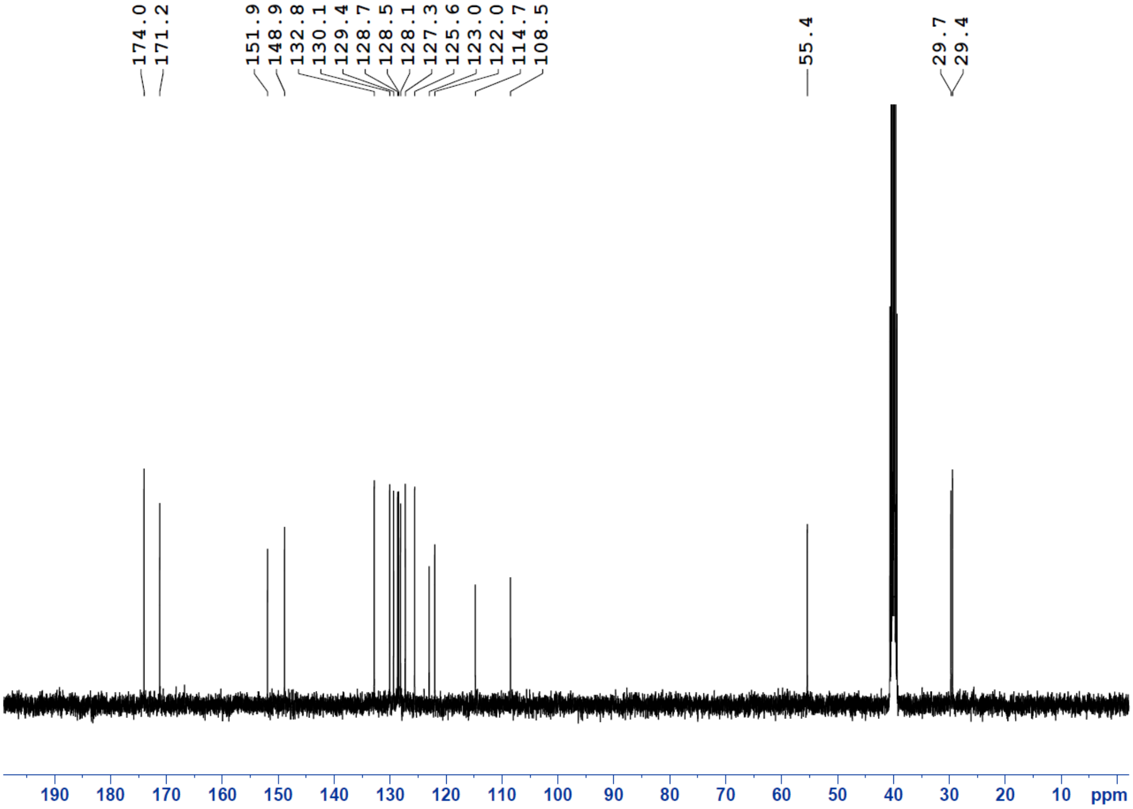
174.0
171.2
151.9
148.9
132.8
130.1
129.4
128.7
128.5
128.1
127.3
125.6
123.0
122.0
114.7
108.5
55.4
29.7
29.4
190 180 170 160 150 140 130 120 110 100 90 80 70 60 50 40 30 20 10 ppm

DBCO-NHS (S15)

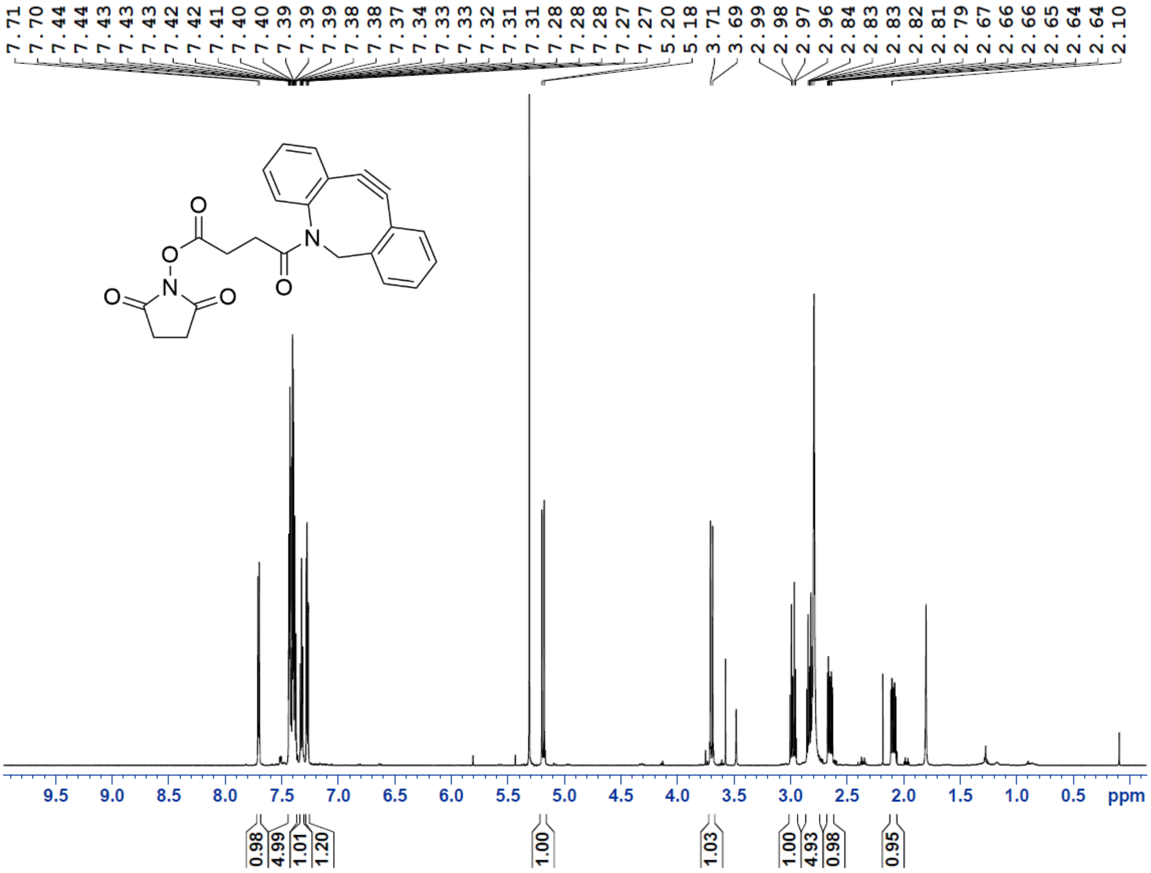
7.71 7.70 7.44 7.44 7.43 7.43 7.43 7.42 7.42 7.41 7.40 7.40 7.39 7.39 7.39 7.38 7.38 7.37 7.34 7.33 7.33 7.32 7.31 7.31 7.28 7.28 7.28 7.27 7.27 5.20 5.18 3.71 3.69 2.99 2.98 2.97 2.96 2.84 2.83 2.83 2.82 2.81 2.79 2.67 2.66 2.66 2.65 2.64 2.64 2.10
O
O
N
O
O
N
O
O
9.5 9.0 8.5 8.0 7.5 7.0 6.5 6.0 5.5 5.0 4.5 4.0 3.5 3.0 2.5 2.0 1.5 1.0 0.5 ppm
0.98 4.99 1.01 1.20 1.00 1.03 1.00 4.93 0.98 0.95

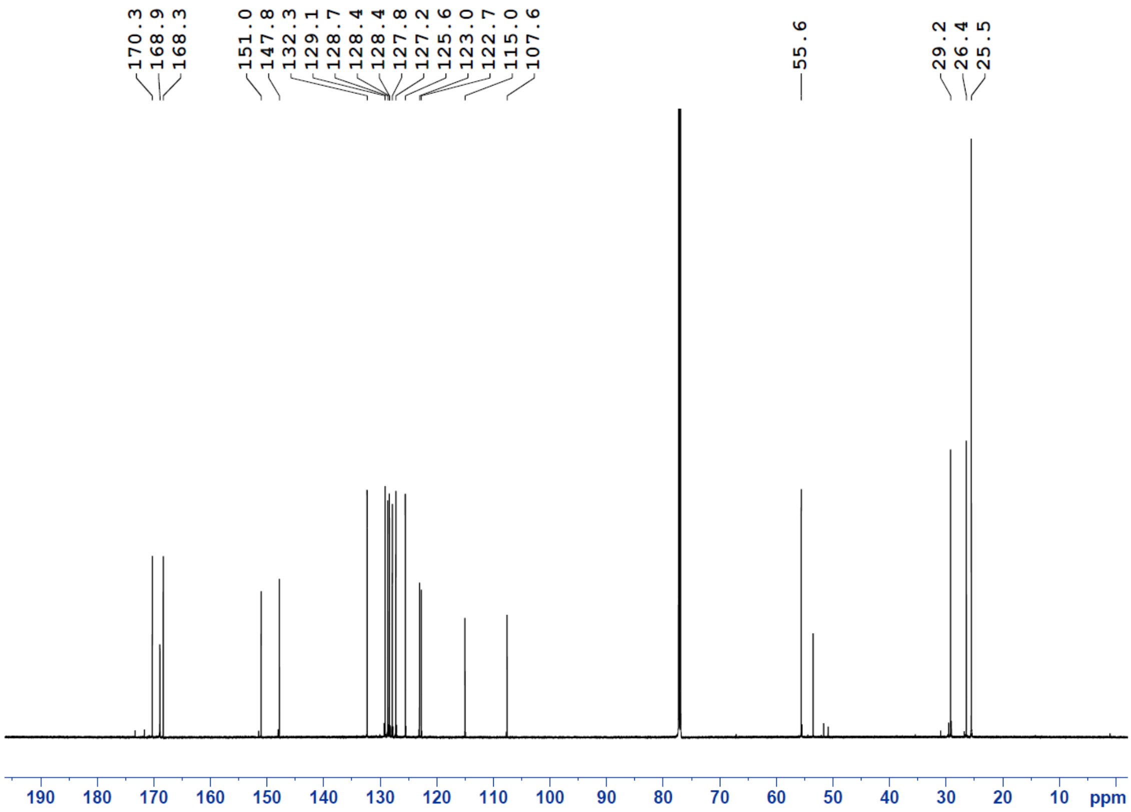
170.3 168.9 168.3 151.0 147.8 132.3 129.1 128.7 128.4 128.4 127.8 127.2 125.6 123.0 122.7 115.0 107.6 55.6 29.2 26.4 25.5
190 180 170 160 150 140 130 120 110 100 90 80 70 60 50 40 30 20 10 ppm

Amino-PEG2-DBCO (S16)

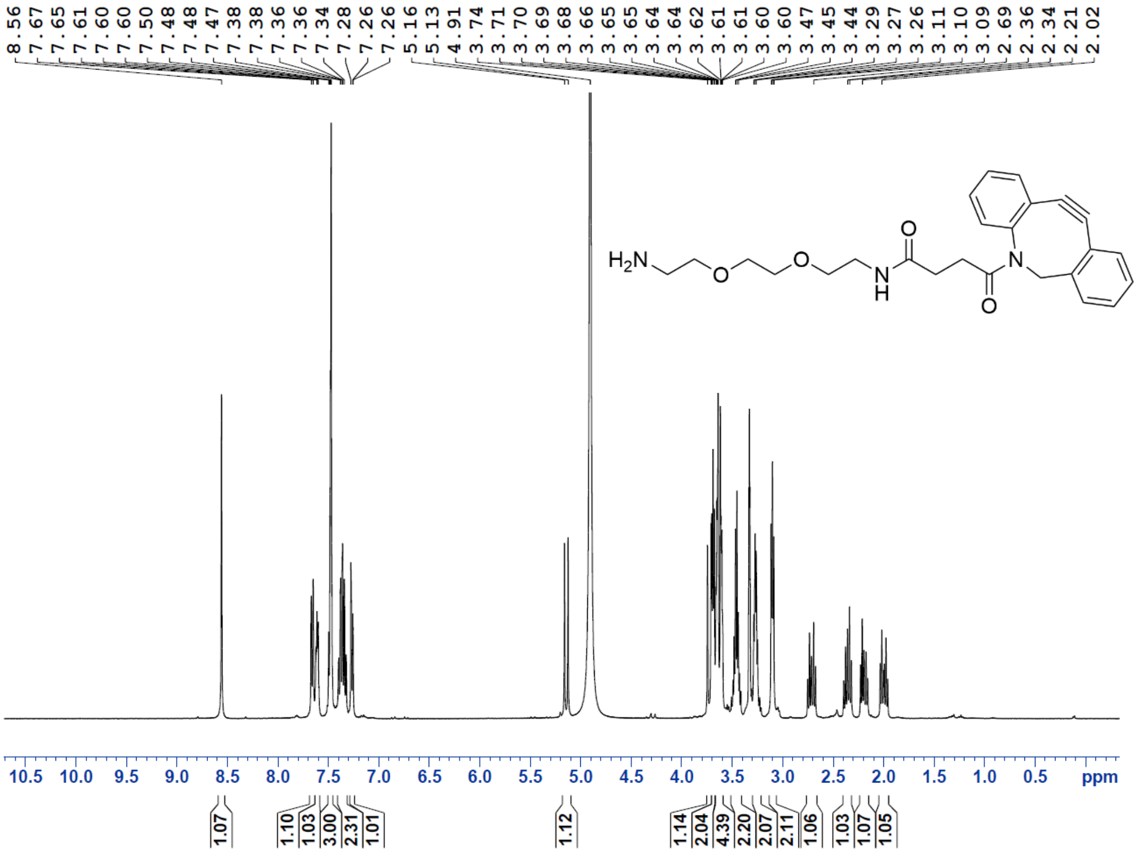

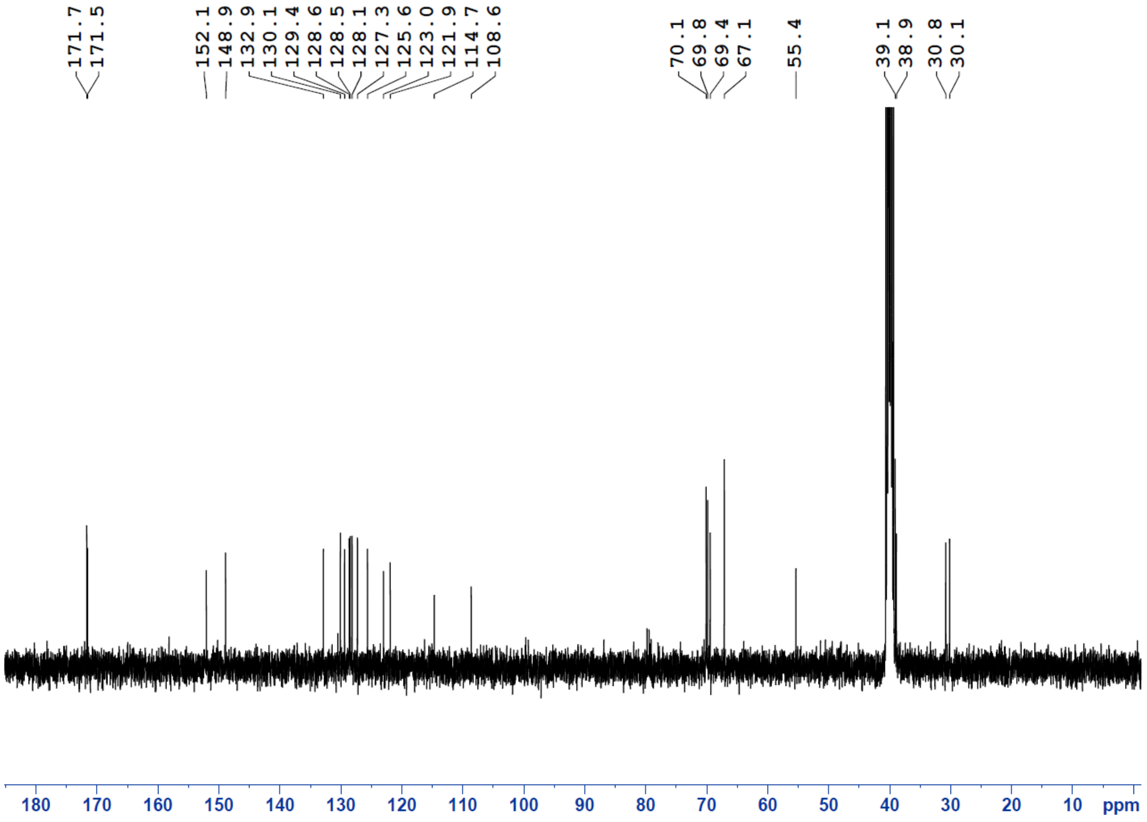

Boc-amino-PEG2-DBCO (S17)

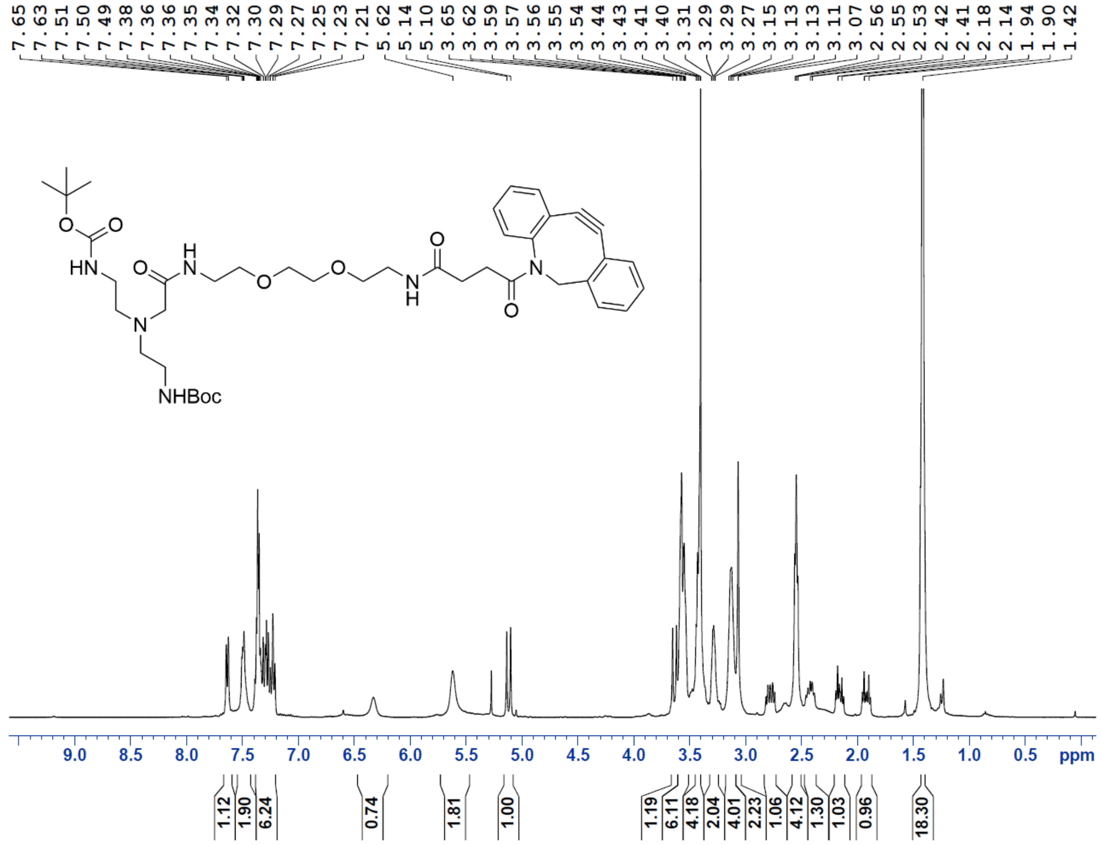

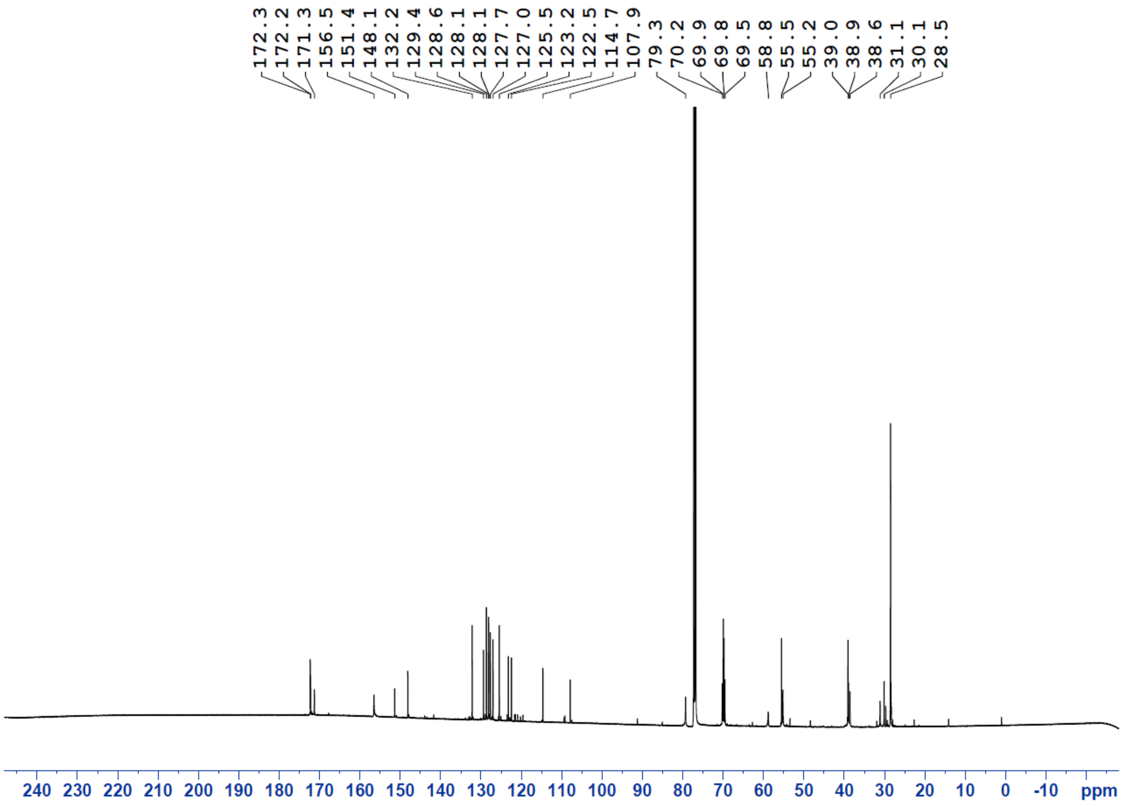

Bis-divinylpyrimidine-DBCO (BisDVP 3)

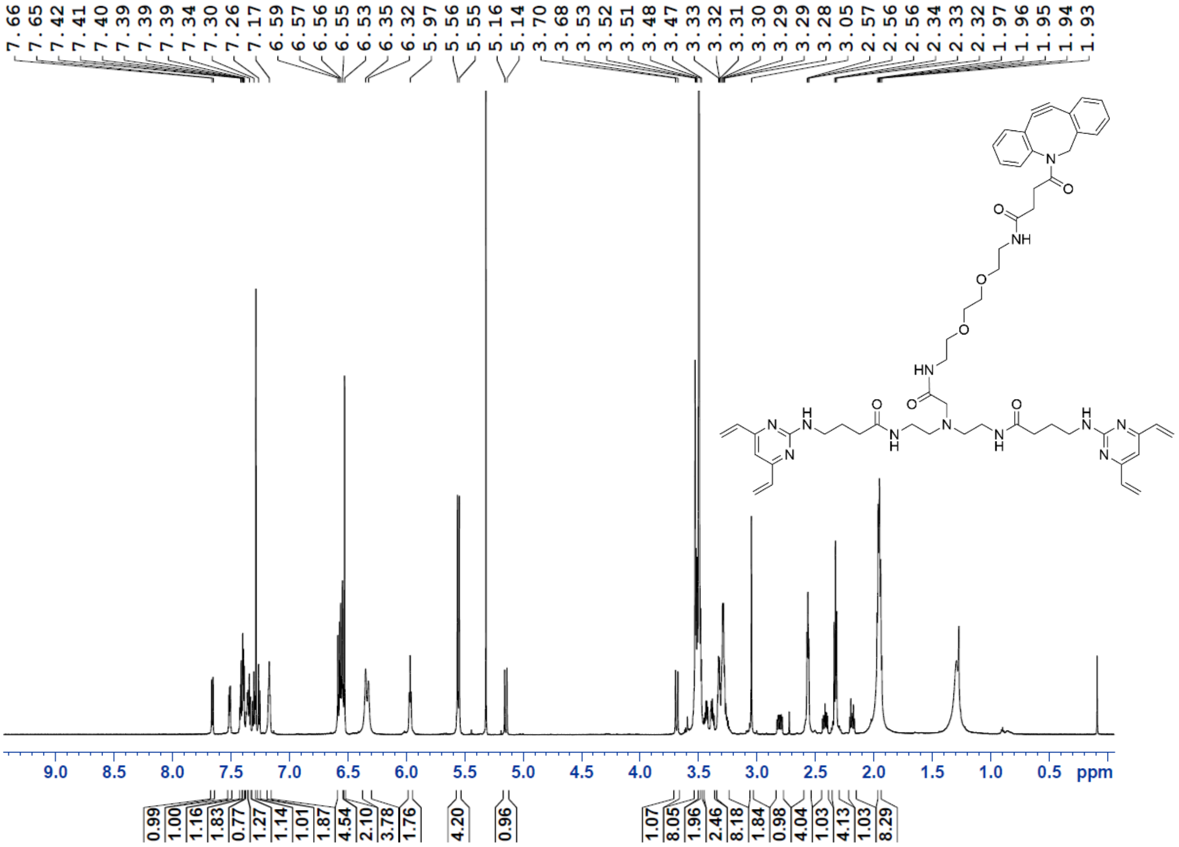

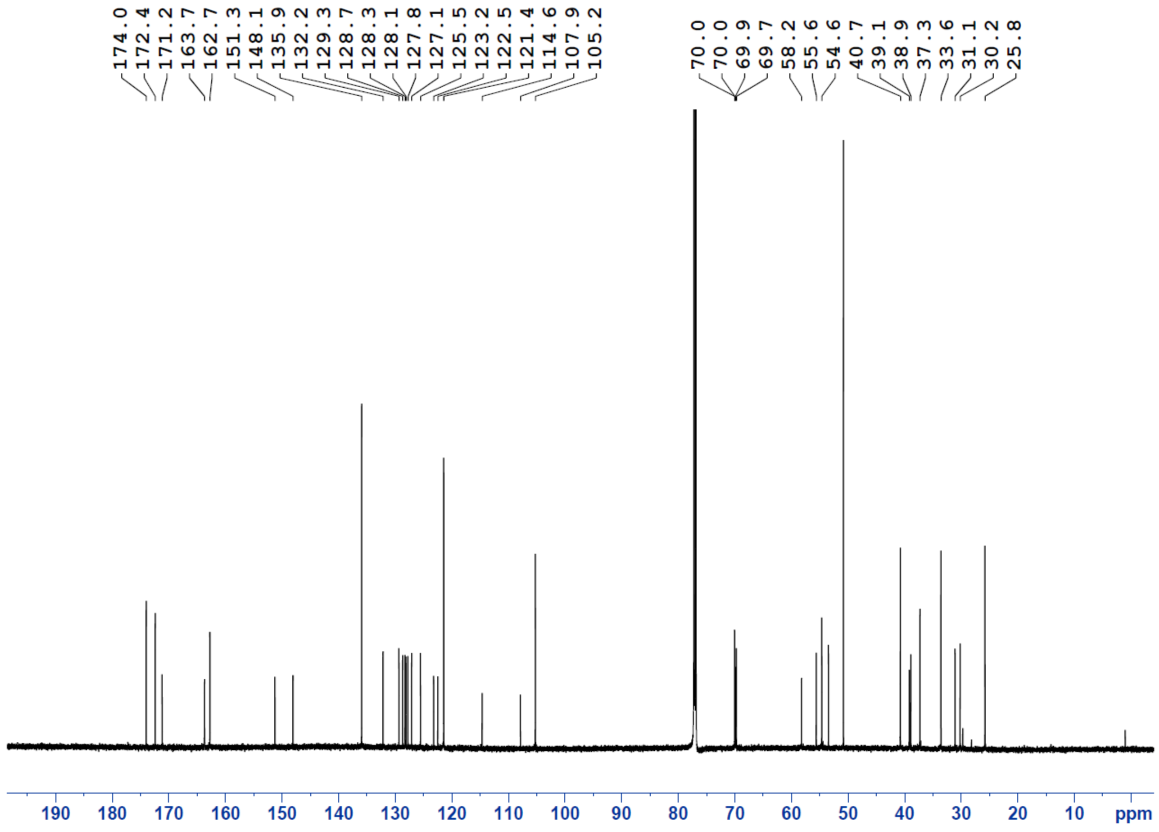

DBCO-PEG2-DBCO (S18)

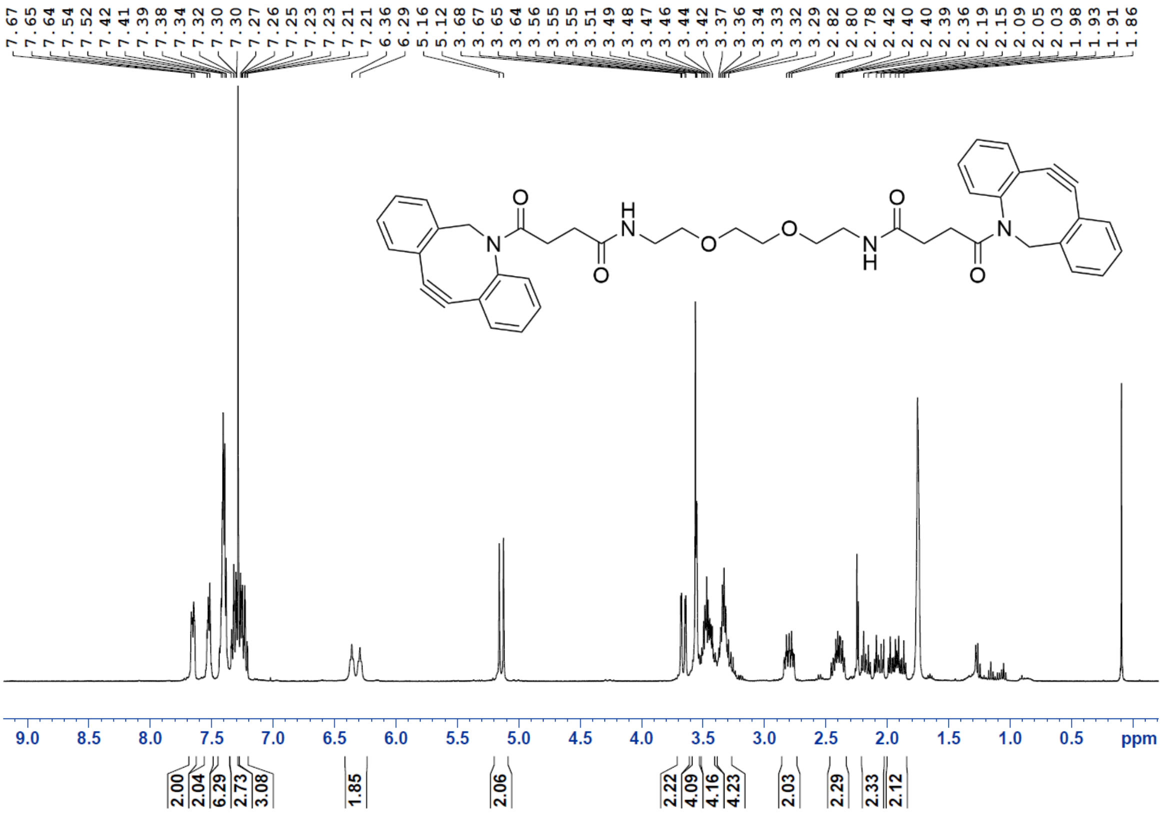

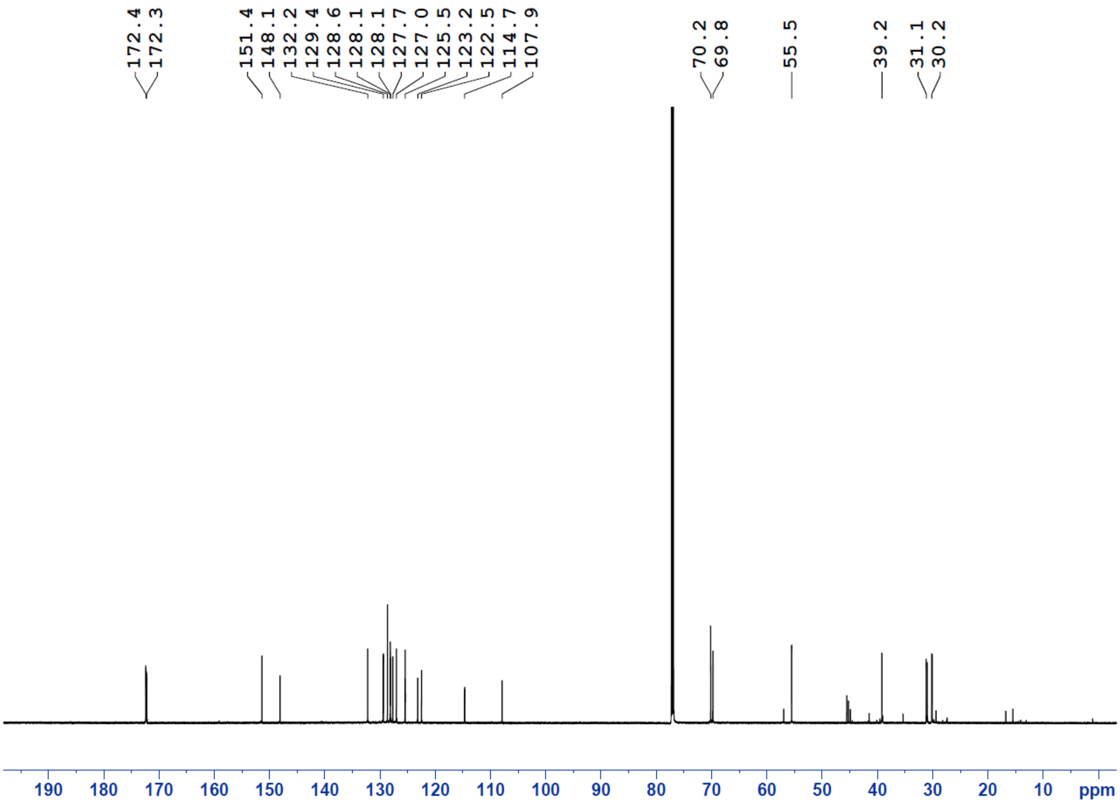

1-(2-(2-(prop-2-yn-1-yloxy)ethoxy)ethyl)-1H-pyrrole-2,5-dione (S19)

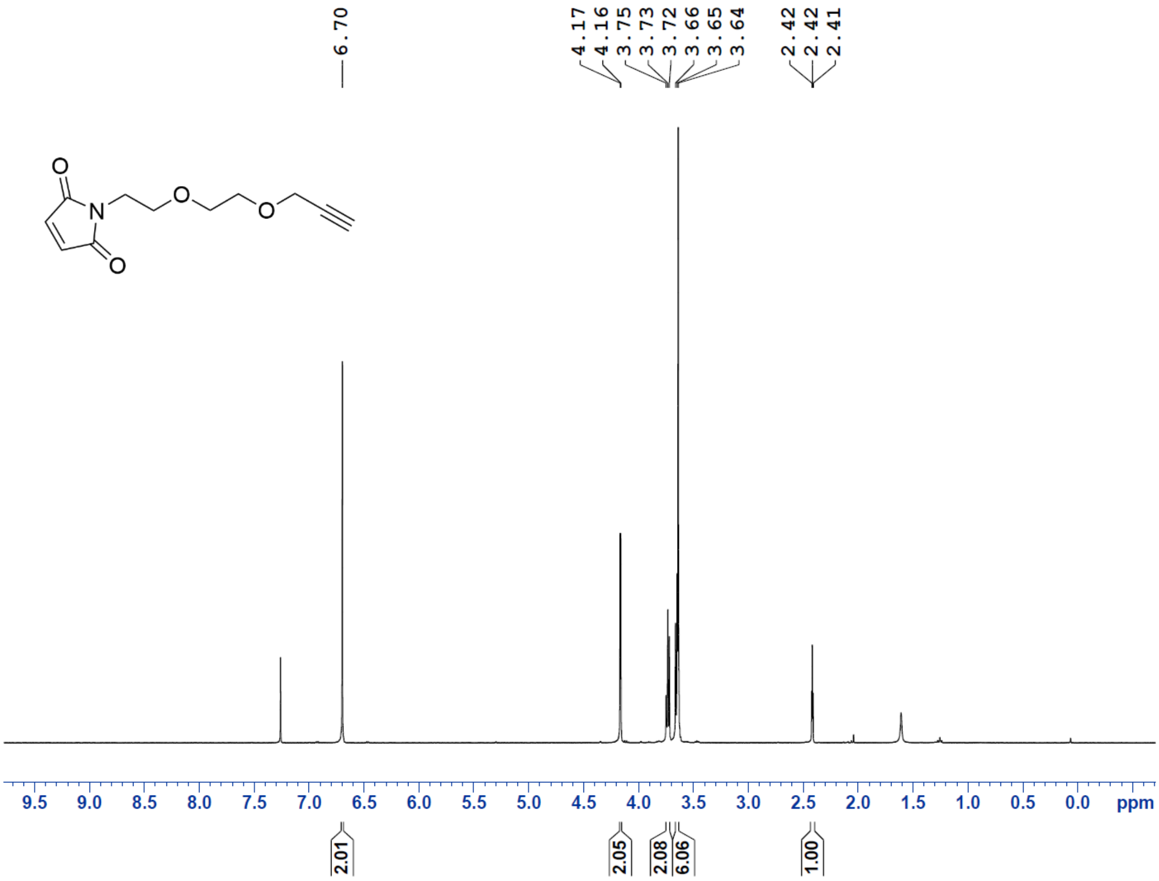

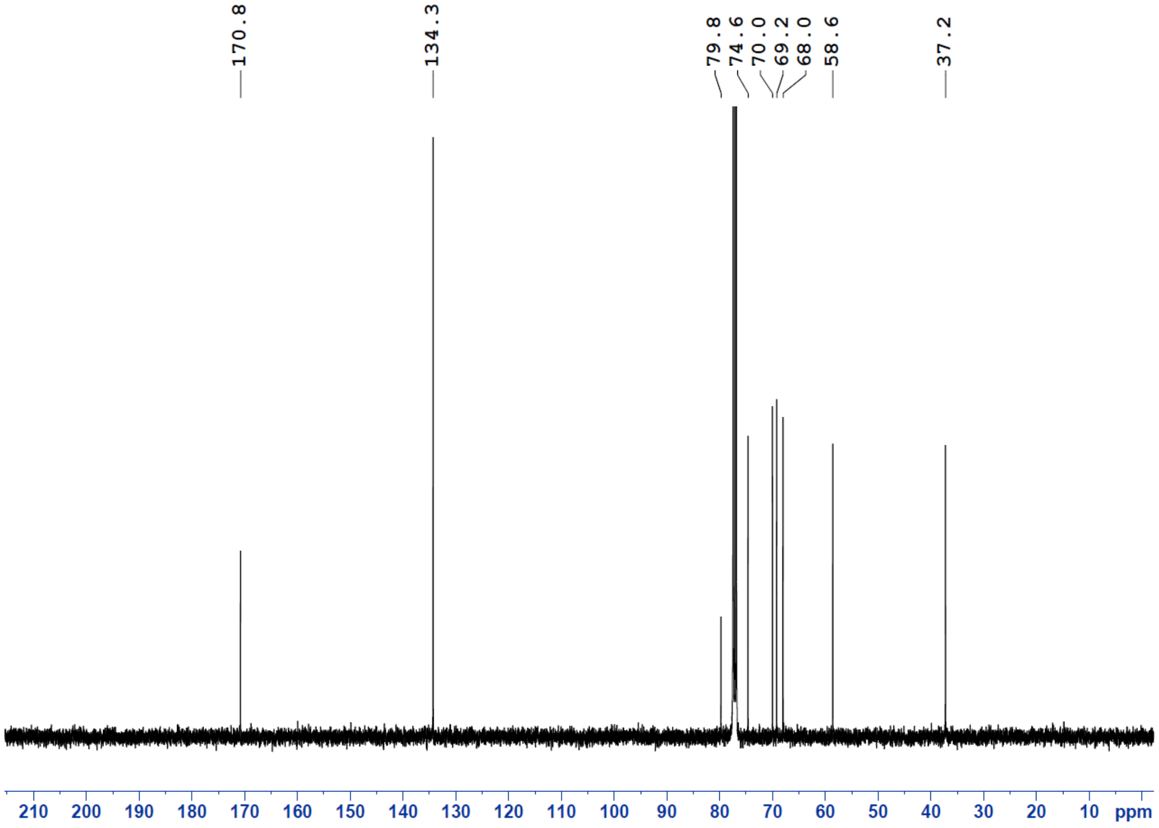

HPLC Traces

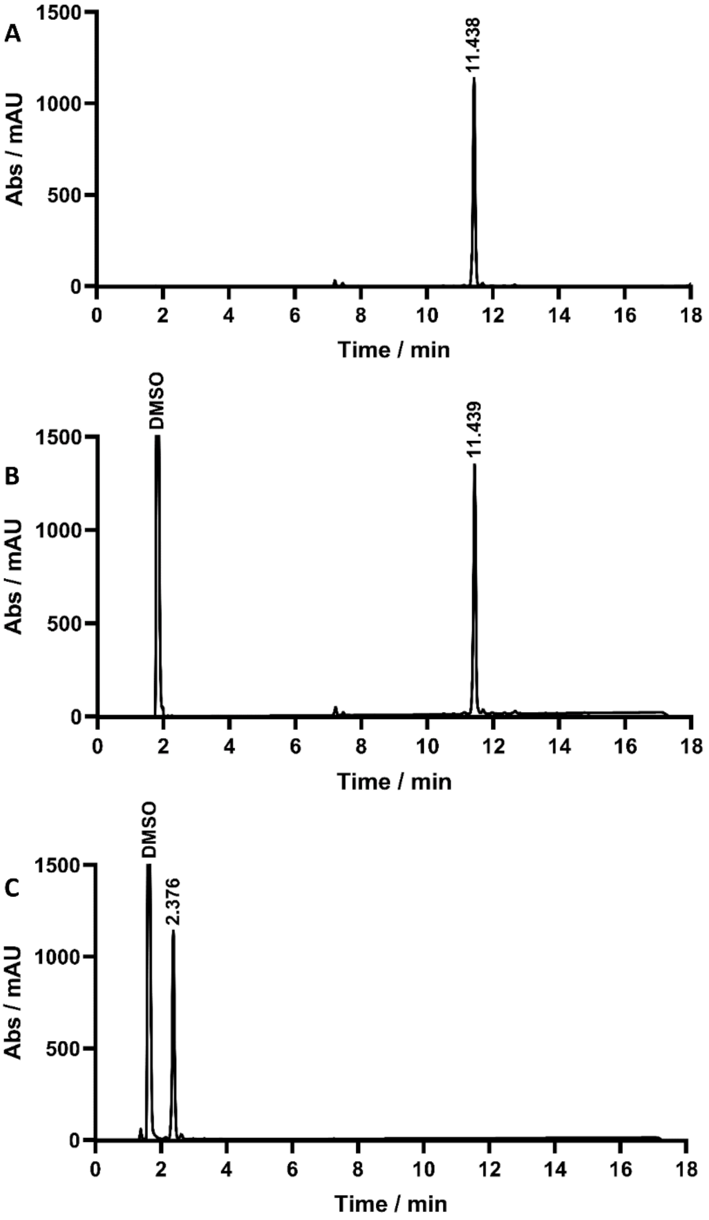

Figure S59: HPLC traces of BisDVP-DBCO **3** with a gradient of A) 10-60% B, Abs: 254 mAU, B) 10-60% B, Abs: 220 mAU and C) 40-60% B Abs: 220 mAU.

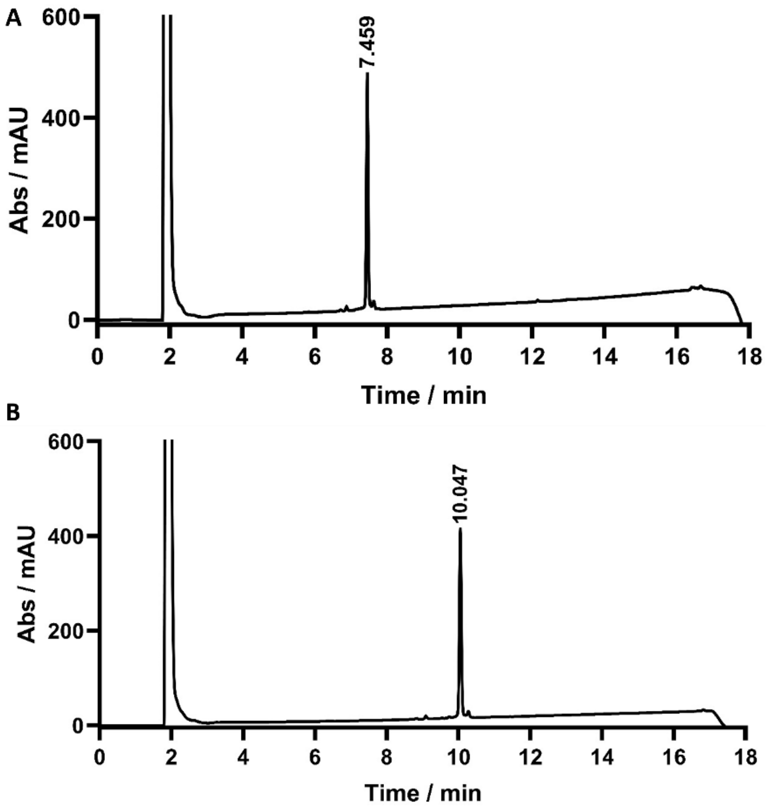

Figure S60: HPLC trace of Afamelanotide-N_3 with a gradient of A) 5-95% B and B) 20-60% B.

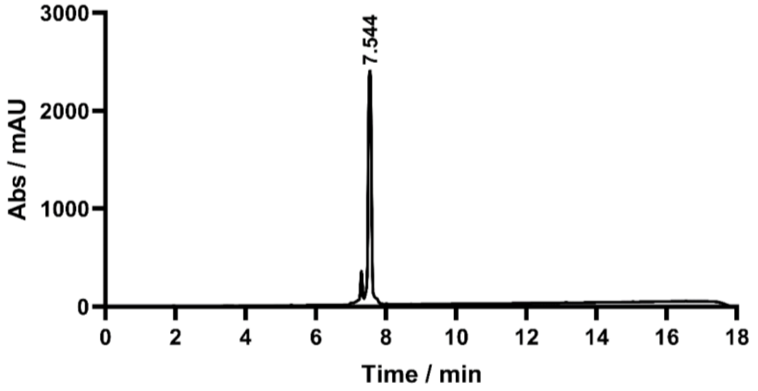

Figure S61: HPLC trace of Afamelanotide-DBCO via BisDBCO **S16** with a gradient of 5-95%.

References:

1. Walsh, S. J.; Iegre, J.; Seki, H.; Bargh, J. D.; Sore, H. F.; Parker, J. S.; Carroll, J. S.; Spring, D. R. *Org. Biomol. Chem.* 2020, **18**, 4224–4230.
2. Dannheim, F. M.; Walsh, S. J.; Orozco, C. T.; Hansen, A. H.; Bargh, J. D.; Jackson, S. E.; Bond, N. J.; Parker, J. S.; Carroll, J. S.; Spring, D. R. *Chem. Sci.,* 2022, **13***, 8781–8790.*
3. McNelles, S. A.; Pantaleo, J. L.; Adronov, A. *Org. Process Res. Dev.,* 2019, **23***, 2740–2745.*
4. Wang, J.; Cao, W.; Zhang, W.; Dou, B.; Ding, X.; Wang, M.; Ma, J.; Li, X. *J. Med. Chem*., 2024, **67**, 8296–8308.
5. Maisonial-Besset, A.; Witkowski, T.; Quintana, M.; Besse, S.; Gaumet, V.; Cordonnier, A.; Alliot, C.; Vidal, A.; Denevault-Sabourin, C.; Tarrit, S.; Levesque, S.; Miot-Noirault, E.; Chezal, J. M. *Molecules*. 2022, **28**, 75.
6. Daramola, O.; Stevenson, J.; Dean, G.; Hatton, D.; Pettman, G.; Holmes, W.; Field, R. *Biotechnol. Prog*., 2014, ***30***, 132–141.
